# Site-specific Umpolung amidation of carboxylic acids via triplet synergistic catalysis

**DOI:** 10.1038/s41467-021-24908-w

**Published:** 2021-07-30

**Authors:** Yunyun Ning, Shuaishuai Wang, Muzi Li, Jie Han, Chengjian Zhu, Jin Xie

**Affiliations:** 1 State Key Laboratory of Coordination Chemistry, Jiangsu Key Laboratory of Advanced Organic Materials, Chemistry and Biomedicine Innovation Center (ChemBIC), School of Chemistry and Chemical Engineering, Nanjing University, Nanjing, China; 2 College of Chemistry and Molecular Engineering, Zhengzhou University, Zhengzhou, China; 3 Advanced Catalytic Engineering Research Center of the Ministry of Education, Hunan University, Changsha, China

**Keywords:** Synthetic chemistry methodology, Photocatalysis

## Abstract

Development of catalytic amide bond-forming methods is important because they could potentially address the existing limitations of classical methods using superstoichiometric activating reagents. In this paper, we disclose an Umpolung amidation reaction of carboxylic acids with nitroarenes and nitroalkanes enabled by the triplet synergistic catalysis of FeI_2_, P(V)/P(III) and photoredox catalysis, which avoids the production of byproducts from stoichiometric coupling reagents. A wide range of carboxylic acids, including aliphatic, aromatic and alkenyl acids participate smoothly in such reactions, generating structurally diverse amides in good yields (86 examples, up to 97% yield). This Umpolung amidation strategy opens a method to address challenging regioselectivity issues between nucleophilic functional groups, and complements the functional group compatibility of the classical amidation protocols. The synthetic robustness of the reaction is demonstrated by late-stage modification of complex molecules and gram-scale applications.

## Abstract (web summary)

Catalytic amide bond-forming methods is important because they could potentially address the existing limitations of classical methods using superstoichiometric activating reagents. Here the authors show an Umpolung amidation reaction of carboxylic acids with nitroarenes and nitroalkanes enabled by FeI_2_, P(V)/P(III) and photoredox catalysis that avoids the production of byproducts.

## Introduction

**Fig. 1:**
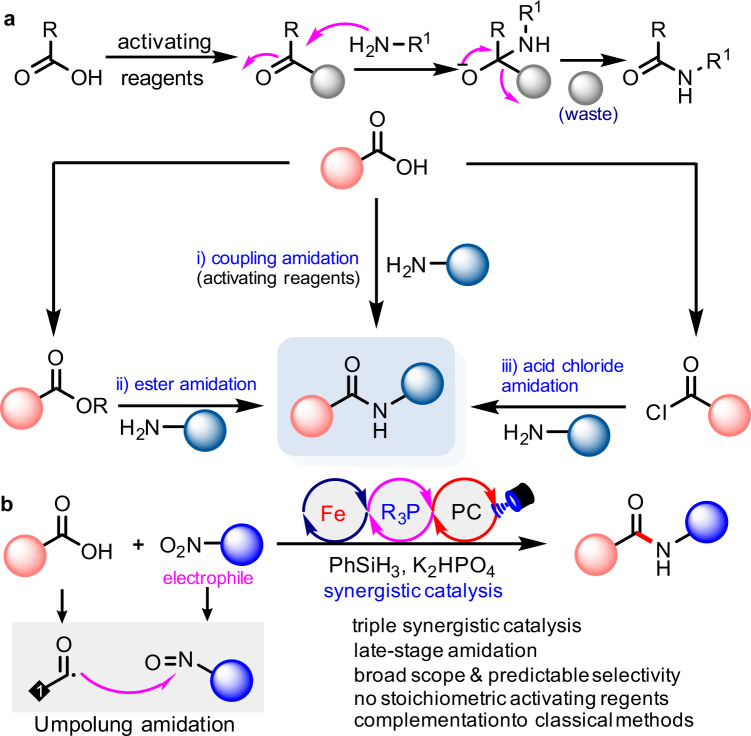
The state-of-the-art of amidation of carboxylic acids. **a** Well-established amidation protocols of carboxylic acids. **b** Iron/P(III)/Photoredox catalysis for Umpolung amidation. PC photocatalyst.

The amide functional group is an important moiety present in a broad spectrum of biologically active compounds, synthetic materials and building blocks^1,2^. About 25% of natural and synthetic drugs on the market contain at least one amide^3^ and consequently, the development of new synthetic strategy for the construction of amide bonds is pivotal in both organic synthesis and pharmaceutical production^4–6^. In addition to the biosynthetic routes^7^, classical synthetic routes from readily available carboxylic acids generally require either reagents which can activate carboxylic acids or preparation of reactive intermediates, such as esters, anhydrides or acyl chlorides for subsequent amidation with nucleophilic amines (Fig. 1a)^8–19^. Nitroarenes are readily available and cheap feedstock reagents^20–27^ and have recently been applied with reduction in situ as amination reagents in amidation^28–30^. For example, Ma et al. recently achieved an elegant one-pot stoichiometric amidation protocol of carboxylic acids with nitroarenes^30^. Although these typical amide bond formation strategies are powerful, the development of a catalytic amidation protocol of carboxylic acids is still being actively pursued in the context of sustainable synthetic chemistry^1,31^ as the carboxylic acid activating reagents not only result in some harmful byproducts but also would compromise the functional group tolerance.

Recently, our group developed a photoredox, Ph_3_P radical cation-mediated deoxygenation of aromatic acids, generating the corresponding acyl radical which participates in a series of novel organic transformations^32–34^. At the same time, Doyle and others also disclosed a similar activation mode of carboxylic acids^35–37^. However the use of stoichiometric amounts of Ph_3_P will diminish the total reaction economy, leading to issues in purification, and the aliphatic carboxylic acids remain challenging substrates with Ph_3_P as deoxygenation reagent under photoredox conditions due to the possibility of their decarboxylation^38–41^. Inspired by Radosevich’s recent seminal work on the P(III)/P(V) catalytic cycle^25,42–46^, we questioned if Umpolung amidation of carboxylic acids is possible with electrophilic amination reagents of nitroarenes. Such a reaction could proceed by means of synergistic photoredox and R_3_P/R_3_P = O catalysis, where the generated nucleophilic acyl radical undergoes radical addition to electrophilic amination reagents as shown in Fig. 1b. This kind of amide bond formation strategy would avoid the use of nucleophilic amine reagents and address the regioselectivity issue between different amine motifs, thus improving the functional group compatibility. A significant difficulty for the Umpolung amidation originates from the fact that the reaction rates between carboxylic C-O homolysis, R_3_P = O and nitroarenes reduction should be matched to support the concerted catalytic cycles. Herein we report an Umpolung amidation strategy of various carboxylic acids with commercially abundant nitroarenes and nitroalkanes by means of iron/R_3_P/photoredox multi-cooperative catalysis.

## Results

### Reaction optimization

Initially, we selected *n*-heptanoic acid (**1a**) and nitrobenzene (**2a**) as model reactants with which to investigate the Umpolung amidation conditions. As shown in Table 1, the standard conditions include synergistic catalysis by 15 mol% FeI_2_, 30 mol% organophosphine (**P-A**) and 1 mol% **PC-I** in the presence of PhSiH_3_ as the reductant (entry 1, also see Supplementary Information for details). Under the standard conditions, the desired amide (**3a**) is obtained in 95% isolated yield. The use of other organophosphine precatalysts ranging from **P-B** to **P-F** significantly decreased the reaction efficiency (entries 2-6). We presumed that **P-A** (R_3_P = O) undergoes rapid reduction rates in the presence of FeI_2_ and silanes to generate R_3_P at room temperature^47–49^. Replacement of FeI_2_ with other iron-based catalysts also led to sharply decreased yields (entries 7 and 8). It was speculated that iron-based catalysts would not only accelerate the reduction of R_3_P = O to R_3_P but would also favor the reduction of nitrobenzene to nitrosobenzene under mild conditions^26,50–52^. Notably, the use of other silanes in place of PhSiH_3_ did not improve the reaction yields (entries 9 and 10, and also see Supplementary Table 3 for details). Control experiments suggested that all the factors, FeI_2_, organophosphine precatalyst, photocatalyst and light irradiation were important for the successful Umpolung amidation (entries 11-14). The triplet catalytic systems should work in concert to meet the total reaction rate demand. A single faster or slower catalytic cycle will mismatch the synergistic effect, thus negatively influencing the reaction.

**Table 1:** Optimization of catalytic Umpolung amidation conditions^a^.


Entry	Variation of standard conditions	Yield(%)^b^
1	None	95
2	**P-B** instead of **P-A**	40
3	**P-C** instead of **P-A**	30
4	**P-D** instead of **P-A**	Trace
5	**P-E** instead of **P-A**	8
6	**P-F** instead of **P-A**	Trace
7	FeCl_2_ instead of FeI_2_	38
8	Fe(acac)_3_ instead of FeI_2_	Trace
9	Ph_2_SiH_2_ instead of PhSiH_3_	18
10	Et_3_SiH instead of PhSiH_3_	Trace
11	Without photocatalyst **PC-I**	ND
12	Without organocatalyst **P-A**	ND
13	Without FeI_2_	Trace
14	Without light irradiation	ND


ND not detected.

^a^Standard conditions: **1a** (0.1 mmol), **2a** (0.12 mmol), **PC-I** (1 mol%), **P-A** (30 mol%), FeI_2_ (15 mol%), K_2_HPO_4_ (0.5 equiv), PhSiH_3_ (0.5 mmol), MeCN (2 ml), blue LEDs, ambient temperature, 24 h.

^b^Isolated yield.

### Substrate scope

**Fig. 2:**
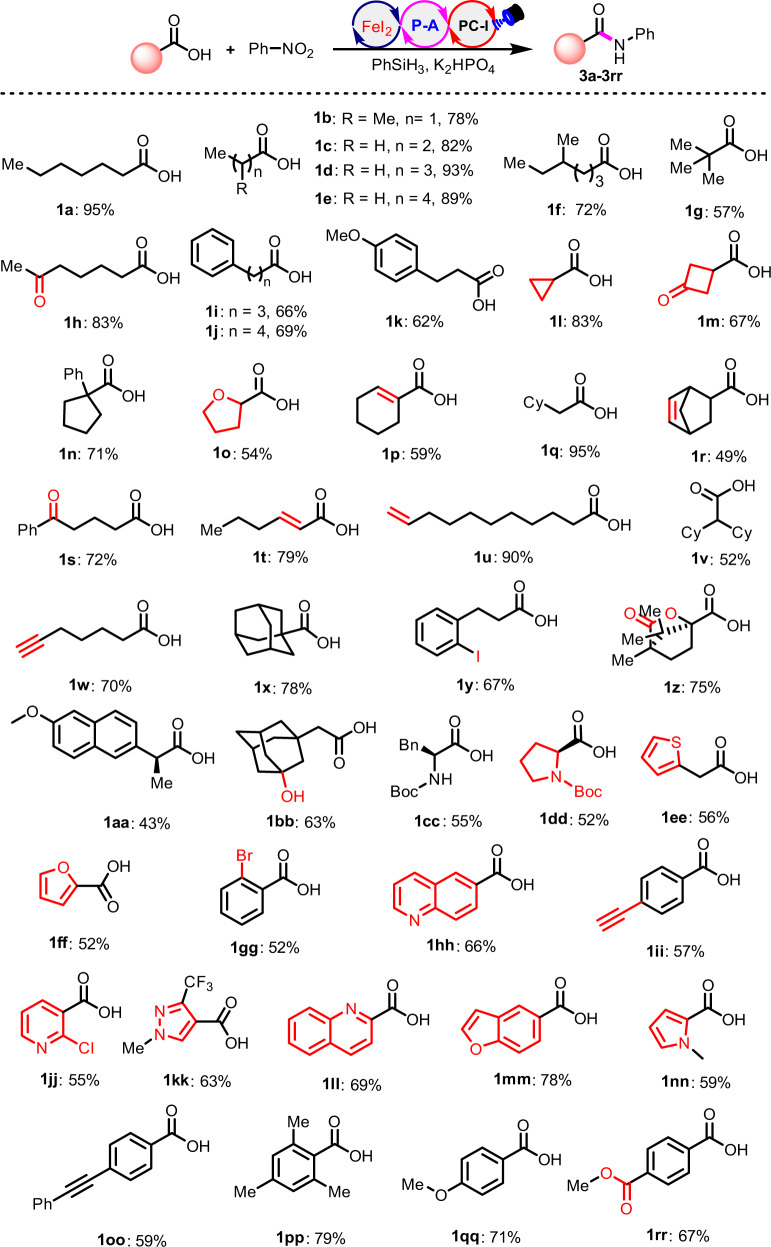
The scope of carboxylic acids. Standard conditions: carboxylic acids **1** (0.2 mmol), **2a** (1.2 equiv), **PC-I** (1 mol%), **P-A** (30 mol%), FeI_2_ (15 mol%), K_2_HPO_4_ (0.5 equiv), PhSiH_3_ (1 mmol), MeCN (4 ml), blue LEDs, ambient temperature, 24-40 h.

With the optimal conditions in hand, we explored the substrate scope of carboxylic acids. As can be seen in Fig. 2, this Umpolung amidation has a satisfactory functional group compatibility and a broad carboxylic acid substrate scope. A series of structurally diverse carboxylic acids (**1a-1rr**) are competent starting materials, affording the desired amides (**3a-3rr**) in moderate to good yields (44 examples, up to 95% yield). Primary, secondary and tertiary aliphatic acids (**1a-1o**, **1q-1s**, **1u-1ee**) all tolerate the conditions well and the protocol has excellent functional group tolerance. A great number of versatile functional groups, such as ketone (**1h**, **1m, 1s**), iodo (**1y**), bromo (**1gg**), cyclopropanyl (**1l**), alkene (**1p**, **1r**, **1u**), alkyne (**1w, 1ii**), ester (**1z**), heterocycle (**1o**, **1dd**-**1ff**, **1hh, 1jj-1nn**) remain intact. Besides aliphatic carboxylic acids, α,β-unsaturated acids (**1p** and **1t**) and aromatic acids (**1gg-1ii, 1oo-1rr**) as well as heteroaromatic acids (**1ff**, **1jj-1nn**) are also efficient coupling partners and can be directly employed for the construction of the corresponding amides. Interestingly, when the chiral amino acids (**1cc** and **1dd**) were subjected to this protocol, the chiral amides were obtained without racemization as determined by HPLC analysis. The use of organophosphine precatalyst (**P-A)** as the deoxygenation catalyst appears to complement Ph_3_P-mediated deoxygenative coupling, significantly expanding the carboxylic acid scope^32–34^.

**Fig. 3:**
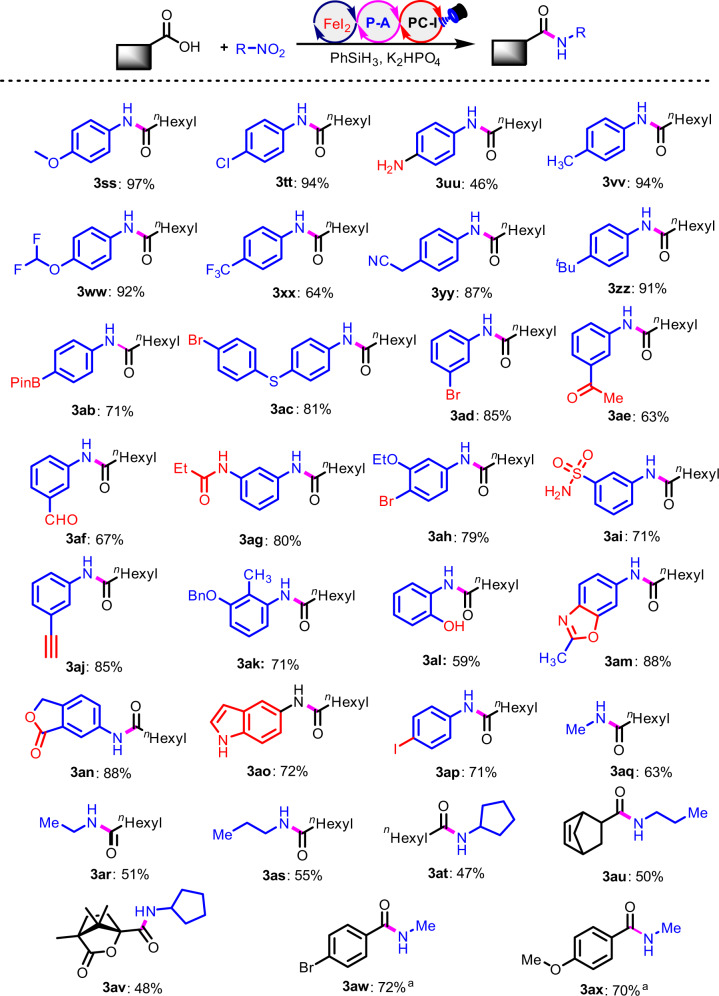
The scope of the organonitro compounds. Standard conditions: **1a** (0.2 mmol), R-NO_2_ **2** (0.24 mmol), **PC-I** (1 mol%), **P-A** (30 mol%), FeI_2_ (15 mol%), K_2_HPO_4_ (0.5 equiv), PhSiH_3_ (1 mmol), MeCN (4 ml), blue LEDs, ambient temperature, 24-36 h. ^*a*^ 2.0 equivalent nitromethane was used.

Subsequently, we studied the scope of the organonitro compounds (Fig. 3). A number of different nitroarenes can be successfully employed for selective formation of desired amides (**3ss-3ax**). Both electron-donating and withdrawing groups on the phenyl rings barely influence the reaction efficiency, delivering the desired products in yields of up to 97%. Importantly, when the nitroarenes bearing nucleophilic functional groups, such free amino (**3uu**), hydroxy (**3al**), NH-free indole (**3ao**), the amide bond formation between carboxylic acid and nitro groups is still predictable. These are cases which are challenging for classical amidation strategies with coupling reagents or transition metal-catalyzed aminocarbonylation^53–55^. There is also good functional group tolerance for nitroarenes. The aldehyde (**3af**), ketone (**3ae**), halogen (**3tt**, **3ac**, **3ad**, **3ah**, **3ap**), heteroarene (**3an**, **3ao**) and Bpin (**3ab**) are quite compatible. The use of nitroalkanes, such as nitromethane, nitroethane, nitropropane and nitrocyclopentane can successfully result in isolation of the desired amides **(3aq-3ax**) in acceptable yields of 47–72%.

### Synthetic application

**Fig. 4:**
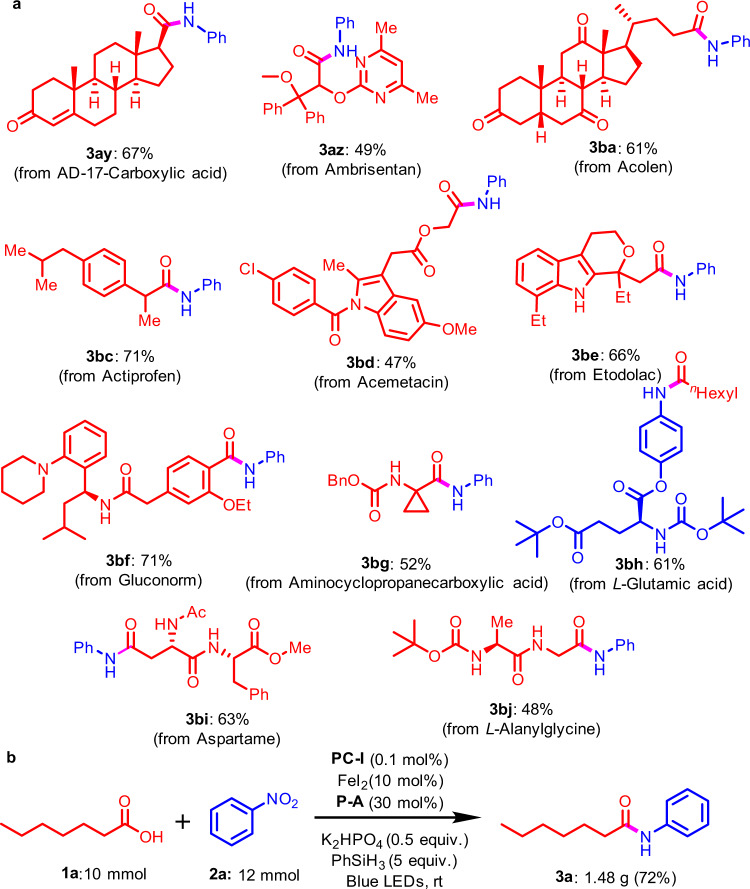
Synthetic application. **a** Late-stage Umpolung amidation of complex molecules. Standard conditions for late-stage modification: carboxylic acids **1** (0.2 mmol), nitroarenes **2** (0.24 mmol), **PC-I** (1 mol%), **P-A** (30 mol%), FeI_2_ (15 mol%), K_2_HPO_4_ (0.5 equiv), PhSiH_3_ (1 mmol), MeCN (4 ml), blue LEDs, 24-60 h. **b** Gram-scale test with 0.1 mol% photocatalyst. Standard conditions for gram-scale experiment: *n*-heptanoic acid **1a** (10 mmol), nitrobenzene **2a** (12 mmol), **PC-I** (0.1 mol%), **P-A** (30 mol%), FeI_2_ (10 mol%), K_2_HPO_4_ (0.5 equiv), PhSiH_3_ (5 equiv), MeCN (100 ml), blue LEDs, 48 h.

To further demonstrate the synthetic robustness of Umpolung amidation, we used this amide bond formation strategy with complex molecules (Fig. 4a). The excellent functional group tolerance and high selectivity of the reaction supports a predictable amide bond formation method. Complex carboxylic acids derived from AD-Acid (**3ay**), ambrisentan (**3az**), acolen (**3ba**), actiprofen (**3bc**), acemetacin (**3bd**), etodolac (**3be**), gluconorm (**3bf**) and aminocyclo-propanecarboxylic acid (**3bg**) are produced smoothly. Complex nitroarenes can also be successfully subjected to this Umpolung amidation reaction, affording for example, the desired product (**3bh**) in 61% yield. Importantly, it was found that this protocol can be compatible for the late-stage modification of some peptides. Under the standard conditions, the carboxylic acid group in dipeptides of aspartame and *L*-alanylglycine can be employed for construction of amide bond in moderate isolated yields (**3bi** and **3bj**). The success of these complex molecules suggests the potential application of the Umpolung amidation method in the late-stage modification of complex molecules. Furthermore, with modified standard conditions using only 0.1 mol% photocatalyst, a scaled-up experiment of 10 mmol can be conducted smoothly to afford the desired product in 72% yield (Fig. 4b).

### Mechanistic studies

**Fig. 5:**
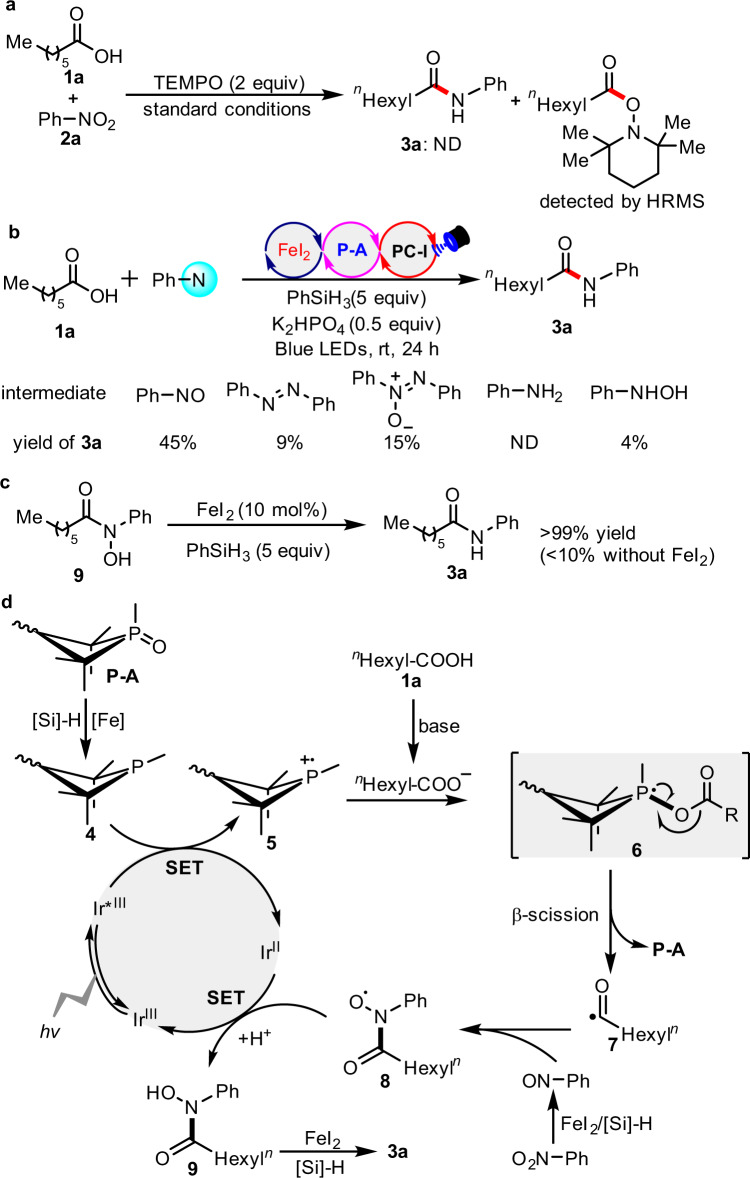
Mechanistic studies and proposed mechanism. **a** Radical inhibition experiment. **b** Potential N-based intermediate. **c** Reduction of **9** to product **3a**. **d** Proposed mechanism.

The following control experiments were performed to gain insight the mechanism of the reaction (Fig. 5). Under the standard conditions, upon addition of 2 equiv of TEMPO (2,2,6,6-tetramethylpiperidine-1-oxyl radical) into the reaction mixture, the reaction was completely inhibited and the corresponding acyl radical was trapped by TEMPO (Fig. 5a), giving a product which was identified by high resolution mass spectroscopy (HRMS). In general, with the use of nitroarenes as electrophilic reagents, there are several kinds of potential N-based intermediates^27,50,52,56,57^. Among the potential intermediates that were screened, we found that the use of nitrosobenzene under experimental conditions could afford the desired product (**3a**) in 45% yield, whereas the other electrophilic N-based intermediates such as 1,2-diphenyldiazene-1-oxide gave only a little product (Fig. 5b). This suggests that nitrosobenzene potentially is the intermediate which reacts with the nucleophilic acyl radical. The use of aniline failed to generate the product (**3a**) but *N*-phenylhydroxylamine gave 4% of the desired product (Fig. 5c). We envisioned that the photoredox conditions with [*Ir(dF(CF_3_)ppy)_2_(dtbbpy)]PF_6_ [^1/2^*E*_red_ (*Ir^III^/Ir^II^) = + 1.21 V]^58^ would slowly oxidize *N*-phenylhydroxylamine to the corresponding nitrosobenzene. In addition, under FeI_2_ catalysis together with PhSiH_3_, the prepared intermediate (**9**), which can also be detected by HRMS in the reaction mixture, can directly be reduced to the final amide (**3a**). In the light of previous work^32–37^, a plausible mechanism was proposed and is shown in Fig. 5d. After irradiation with blue LEDs, the excited photocatalyst [*Ir(dF(CF_3_)ppy)_2_(dtbbpy)]PF_6_ [^1/2^*E*_red_ (*Ir^III^/Ir^II^) = + 1.21 V]^58^ causes a single electron oxidation of electron-rich R_3_P (**4**), which can be formed by reduction in situ from the precatalyst R_3_P = O (**P-A**) in the presence of PhSiH_3_ and FeI_2_, generating the corresponding phosphine radical cation species (**5**). The Stern-Volmer quenching experiments demonstrated that the photoexcited [*Ir(dF(CF_3_)ppy)_2_(dtbbpy)]PF_6_ could be quenched by R_3_P rather than R_3_P = O (**P-A**), *n*-heptanoic acid (**1a**) or nitrobenzene (**2a**). Subsequently, this species (**5**) can recombine with the carboxylate anion to furnish the radical intermediate (**6**). Owing to the high affinity of P- and O-atoms, intermediate (**6**) prefers to undergo β-scission to generate nucleophilic acyl radical (**7**) and complete the organophosphine catalytic cycle. Under the reductive reaction conditions with FeI_2_, the nitrobenzene (**2a**) tends to form nitrosobenzene. Once the concentration of nitrosobenzene is relatively high, rapid nucleophilic acyl radical addition to nitrosobenzene occurs readily to give rise to N-centered radical intermediate (**8**). Donation of one electron to the Ir(II)-species would generate intermediate (**9**), completing the photoredox cycle. Finally, our control experiment showed that reduction of intermediate (**9**) with FeI_2_/PhSiH_3_ to amide (**3a**) at ambient temperature was almost quantitative. Less than 10% of amide (**3a**) was obtained under the identical conditions without the addition of catalytic amount of FeI_2_.

## Discussion

We have developed an unprecedented synergistic catalysis system of iron/P(V-III)/photoredox catalysis for Umpolung amidation of carboxylic acids and nitroarenes or nitroalkanes. A wide range of commercially abundant and inexpensive carboxylic acids and nitroarenes are competent coupling partners in this direct amidation, affording a rich library of structurally diverse amides in yields of up to 97%. This amidation strategy surpasses the classical amide bond-forming method via carboxylic acid activation and subsequent amidation with nucleophilic amines, thus creating promising synthetic robustness especially when the substrates already have several sensitive functional groups or competing nucleophilic substituents. This protocol is readily scaled-up with 0.1 mol% photocatalyst for preparative systems. The excellent functional group compatibility and reaction selectivity render it useful in future peptide modification and drug discovery.

## Methods

### General procedure for amidation

To an 8 mL transparent vial equipped with a stirring bar, **P-A** (10.4 mg, 30 mol%), **PC-I** (2.2 mg, 1 mol%), FeI_2_ (9.3 mg, 15 mol%), K_2_HPO_4_ (17.4 mg, 0.1 mmol) were added successively. Then the vial was carried into glovebox which was equipped with nitrogen. Then MeCN (4.0 ml), PhSiH_3_ (1 mmol), carboxylic acids **1** (0.2 mmol) and nitroarenes **2** (0.24 mmol) were added in sequence under N_2_ atmosphere. The reaction mixture was stirred under the irradiation of 45 W blue LEDs (distance app. 10.0 cm from the bulb) at ambient temperature for 24–60 h. When the reaction finished, the mixture was quenched with water and extracted with ethyl acetate (3 × 10 mL). The organic layers were combined and concentrated under vacuo. The product was purified by flash column chromatography on silica gel (eluent: *n-*hexane: ethyl acetate).

## Supplementary information

Supplementary Information

## Data Availability

We declare that all other data supporting the findings of this study are available within the article and Supplementary Information files, and also are available from the corresponding author upon reasonable request. The X-ray crystallographic data of product **3tt** in this study has been deposited in the Cambridge Crystallographic Data Centre under accession code CCDC 2055473.

## References

[1] Sabatini MT, Boulton LT, Sneddon HF, Sheppard TD (2019). A green chemistry perspective on catalytic amide bond formation. Nat. Catal..

[2] Wang X (2019). Challenges and outlook for catalytic direct amidation reactions. Nat. Catal..

[3] Roughley SD, Jordan AM (2011). The medicinal chemist’s toolbox: An analysis of reactions used in the pursuit of drug candidates. J. Med. Chem..

[4] Carey JS (2006). Analysis of the reactions used for the preparation of drug candidate molecules. Org. Biomol. Chem..

[5] Narendar Reddy T, Beatriz A, Jayathirtha Rao V, de Lima DP (2019). Carbonyl compounds’ journey to amide bond formation. Chem. Asian J..

[6] Massolo, E., Pirola, M. & Benaglia, M. Amide bond formation strategies: latest advances on a dateless transformation.* Eur. J. Org. Chem*. **30**, 4641–4651 (2020).

[7] Dorr BM, Fuerst DE (2018). Enzymatic amidation for industrial applications. Curr. Opin. Chem. Bio..

[8] Valeur E, Bradley M (2009). Amide bond formation: beyond the myth of coupling reagents. Chem. Soc. Rev..

[9] Hu L (2016). Ynamides as racemization-free coupling reagents for amide and peptide synthesis. J. Am. Chem. Soc..

[10] Dunetz JR, Magano J, Weisenburger GA (2016). Large-scale applications of amide coupling reagents for the synthesis of pharmaceuticals. Org. Process Res. Dev..

[11] Braddock DC (2018). Tetramethyl orthosilicate (TMOS) as a reagent for direct amidation of carboxylic acids. Org. Lett..

[12] Sayes M, Charette AB (2017). Diphenylsilane as a coupling reagent for amide bond formation. Green. Chem..

[13] Allen CL, Williams JMJ (2011). Metal-catalysed approaches to amide bond formation. Chem. Soc. Rev..

[14] Li G, Ji C, Hong X, Szostak M (2019). Highly chemoselective, transition-metal-free transamidation of unactivated amides and direct amidation of alkyl esters by N-C/O-C cleavage. J. Am. Chem. Soc..

[15] Ishihara K, Ohara S, Yamamoto H (1996). 3,4,5-Trifluorobenzeneboronic acid as an extremely active amidation catalyst. J. Org. Chem..

[16] Sawant DN (2018). Diboron-catalyzed dehydrative amidation of aromatic carboxylic acids with amines. Org. Lett..

[17] Du Y (2019). A solid-supported arylboronic acid catalyst for direct amidation. Chem. Commun..

[18] Movahed FS (2020). Tris(o-phenylenedioxy)cyclotriphosphazene as a promoter for the formation of amide bonds between aromatic acids and amines. Synthesis.

[19] Ramachandran PV, Hamann HJ (2021). Ammonia-borane as a catalyst for the direct amidation of carboxylic acids. Org. Lett..

[20] Gao Y, Yang S, Huo Y, Hu X‐Q (2020). Recent progress on reductive coupling of nitroarenes by using organosilanes as convenient reductants. Adv. Synth. Catal..

[21] Li, G. et al. Light-promoted C-N coupling of aryl halides with nitroarenes. *Angew. Chem. Int. Ed.* **60**, 5230–5234 (2021). 10.1002/anie.202012877 33184920

[22] Cheung CW, Ploeger ML, Hu X (2017). Direct amidation of esters with nitroarenes. Nat. Commun..

[23] Gui J (2015). Practical olefin hydroamination with nitroarenes. Science.

[24] Rauser M, Ascheberg C, Niggemann M (2017). Electrophilic amination with nitroarenes. Angew. Chem. Int. Ed..

[25] Li G (2020). An improved P(III)/P(V)=O catalyzed reductive C-N coupling of nitroaromatics and boronic acids by mechanistic differentiation of rate- and product-determining steps. J. Am. Chem. Soc..

[26] Song H, Yang Z, Tung C-H, Wang W (2019). Iron-catalyzed reductive coupling of nitroarenes with olefins: intermediate of iron–nitroso complex. ACS Catal..

[27] Xiao J, He Y, Ye F, Zhu S (2018). Remote sp^3^ C–H amination of alkenes with nitroarenes. Chem.

[28] Lee K, Kim J, Kim J (2002). One pot conversion of nitroarenes into N-arylamides. Bull. Korean Chem. Soc..

[29] Kumar V, Kumar M, Sharma S, Kumar N (2014). Highly selective direct reductive amidation of nitroarenes with carboxylic acids using cobalt(II) phthalocyanine/PMHS. RSC Adv..

[30] Wang S, Cheung CW, Ma J (2019). Direct amidation of carboxylic acids with nitroarenes. J. Org. Chem..

[31] Todorovic M, Perrin DM (2020). Recent developments in catalytic amide bond formation. Pept. Sci..

[32] Zhang M, Xie J, Zhu C (2018). A general deoxygenation approach for synthesis of ketones from aromatic carboxylic acids and alkenes. Nat. Commun..

[33] Ruzi R, Liu K, Zhu C, Xie J (2020). Upgrading ketone synthesis direct from carboxylic acids and organohalides. Nat. Commun..

[34] Zhang M, Yuan X-A, Zhu C, Xie J (2019). Deoxygenative deuteration of carboxylic acids with D_2_O. Angew. Chem. Int. Ed..

[35] Martinez Alvarado JI, Ertel AB, Stegner A, Stache EE, Doyle AG (2019). Direct use of carboxylic acids in the photocatalytic hydroacylation of styrenes to generate dialkyl ketones. Org. Lett..

[36] Stache EE, Ertel AB, Tomislav R, Doyle AG (2018). Generation of phosphoranyl radicals via photoredox catalysis enables voltage-independent activation of strong C-O bonds. ACS Catal..

[37] Zhang L (2019). Reductive C–C coupling by desulfurizing gold-catalyzed photoreactions. ACS Catal..

[38] Zhou Q (2015). Decarboxylative alkynylation and carbonylative alkynylation of carboxylic acids enabled by visible-light photoredox catalysis. Angew. Chem. Int. Ed..

[39] Patra T, Mukherjee S, Ma J, Strieth-Kalthoff F, Glorius F (2019). Visible-light-photosensitized aryl and alkyl decarboxylative functionalization reactions. Angew. Chem. Int. Ed..

[40] Shibatomi K (2017). Enantioselective decarboxylative chlorination of beta-ketocarboxylic acids. Nat. Commun..

[41] Liang Y, Zhang X, MacMillan DWC (2018). Decarboxylative sp^3^ C–N coupling via dual copper and photoredox catalysis. Nature.

[42] Li G, Grotenhuis C, Radosevich AT (2021). Reductive Csp^2^–N coupling by P^III^/P^V^=O catalysis. Trends Chem..

[43] Lecomte M, Lipshultz JM, Kim-Lee SH, Li G, Radosevich AT (2019). Driving recursive dehydration by P(III)/P(V) catalysis: Annulation of amines and carboxylic acids by sequential C-N and C-C bond formation. J. Am. Chem. Soc..

[44] Nykaza TV (2018). Intermolecular reductive C-N cross coupling of nitroarenes and boronic acids by P^III^/P^V^=O catalysis. J. Am. Chem. Soc..

[45] Li G, Qin Z, Radosevich AT (2020). P(III)/P(V)-catalyzed methylamination of arylboronic acids and esters: reductive C–N coupling with nitromethane as a methylamine surrogate. J. Am. Chem. Soc..

[46] Nykaza TV, Li G, Yang J, Luzung MR, Radosevich AT (2020). PIII/PV=O catalyzed cascade synthesis of N‐functionalized azaheterocycles. Angew. Chem. Int. Ed..

[47] O’Brien CJ (2009). Recycling the waste: the development of a catalytic Wittig reaction. Angew. Chem. Int. Ed..

[48] Marsi KL (1974). Phenylsilane reduction of phosphine oxides with complete stereospecificity. J. Org. Chem..

[49] Kirk AM, O’Brien CJ, Krenske EH (2020). Why do silanes reduce electron-rich phosphine oxides faster than electron-poor phosphine oxides?. Chem. Commun..

[50] Junge K, Wendt B, Shaikh N, Beller M (2010). Iron-catalyzed selective reduction of nitroarenes to anilines using organosilanes. Chem. Commun..

[51] Lo JC, Yabe Y, Baran PS (2014). A practical and catalytic reductive olefin coupling. J. Am. Chem. Soc..

[52] Zhu K, Shaver MP, Thomas SP (2016). Chemoselective nitro reduction and hydroamination using a single iron catalyst. Chem. Sci..

[53] Wang L, Neumann H, Beller M (2019). Palladium-catalyzed methylation of nitroarenes with methanol. Angew. Chem. Int. Ed..

[54] Zheng Y-L, Newman SG (2019). Methyl esters as cross-coupling electrophiles: Direct synthesis of amide bonds. ACS Catal..

[55] Yuan Y (2020). Copper-catalyzed carbonylative hydroamidation of styrenes to branched amides. Angew. Chem. Int. Ed..

[56] Cheung CW, Leendert Ploeger M, Hu X (2018). Amide synthesis via nickel-catalysed reductive aminocarbonylation of aryl halides with nitroarenes. Chem. Sci..

[57] Yang X-J, Chen B, Zheng L-Q, Wu L-Z, Tung C-H (2014). Highly efficient and selective photocatalytic hydrogenation of functionalized nitrobenzenes. Green. Chem..

[58] Prier CK, Rankic DA, MacMillan DWC (2013). Visible light photoredox catalysis with transition metal complexes: applications in organic synthesis. Chem. Rev..

