# Supplementary material for: Site-specific Umpolung amidation of carboxylic acids via triplet synergistic catalysis
Source: Nat Commun. 2021 Jul 30;12:4637. doi: 10.1038/s41467-021-24908-w (PMC8324892; doi:10.1038/s41467-021-24908-w)
Supplement: Supplementary file 1 — Supplementary Information [file 41467_2021_24908_MOESM1_ESM.pdf]

SUPPLEMENTARY INFORMATION

Site-Specific Umpolung Amidation of Carboxylic Acids via Triplet Synergistic Catalysis

Ning et al.

Supplementary Methods

1 General information

All the reactions were conducted in transparent vials under Argon atmosphere unless otherwise noted. All solvents were obtained from commercial suppliers and used without further purification. Anhydrous MeCN was purified from MeCN (≥99.9%, HPLC) by Solvent Purification System. Reagents were purchased from Energy Chemical, Adamas-beta, and etc. Flash column chromatographic purification of products was accomplished using forced-flow chromatography on Silica Gel (300-400 mesh).

^{1}H NMR, ^{13}C NMR spectra were recorded on a 400 or 500 MHz spectrometer in $CDCl_3$ (δH = 0.0 ppm, δC = 77.02 ppm as standard). Data for ^{1}H NMR are reported as follows: chemical shift (ppm, scale), multiplicity, coupling constant (Hz), and integration. Data for ^{13}C NMR are reported in terms of chemical shift (ppm, scale), multiplicity, and coupling constant (Hz). The following abbreviations were used for ^{1}H NMR spectra to indicate the signal multiplicity: s (singlet); brs (broad singlet), d (doublet), t (triplet), q (quartet), quint (quintet), sext (sextet), sept (septet) and m (multiplet) as well as combinations of them. Gas chromatographic (GC) analyses were performed on a GC equipped with a flameionization detector and an Rtx@-65 (30 m × 0.32 mm ID × 0.25 μm df) column. GC-MS analyses were performed on a GC-MS with an EI mode. High-resolution mass spectra were obtained by ESI on a TOF mass analyzer. And the 45 W blue LEDs light was purchased from Kessil (A360NE/WE). The organophsophine (**P-A**) was synthesized according to literature.[1, 2]

2 Optimization of reaction conditions of umpolung amidation

Supplementary Table 1. Screening of photocatalysts.

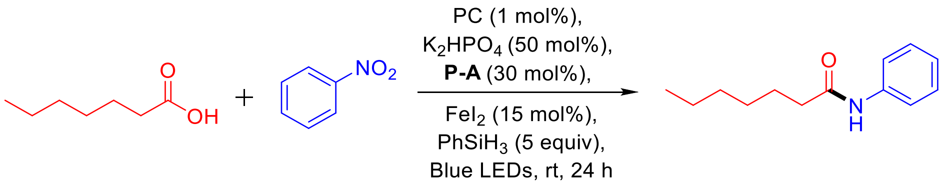

Entry	Photocatalyst	Yield (%)
1	Ir[dFCF$_3$(ppy)]$_2$(dtbbpy)PF$_6$	95
2	Ir(ppy)$_2$(dtbbpy)PF$_6$	74

3	Ir(ppy)$_3$	trace
4	Mes-Acr$^+$ClO$_4$	trace
5	Ir[dFMe(ppy)]$_2$(dtbbpy)	40
6	EoSin Y	trace
7	PTH	trace
8	Ru(bpy)$_3$Cl$_2$ 6H$_2$O	26
9	Ru(bpy)$_3$Cl$_2$	trace
10	4CZIPN	trace

Reaction conditions: **1a** (0.1 mmol), **2a** (0.12 mmol), **PC** (1 mol%), **P-A** (30 mol%), FeI$_2$ (15 mol%), K$_2$HPO$_4$ (0.5 equiv), PhSiH$_3$ (0.5 mmol), MeCN (2 mL), Blue LEDs, ambient temperature, 24 h.

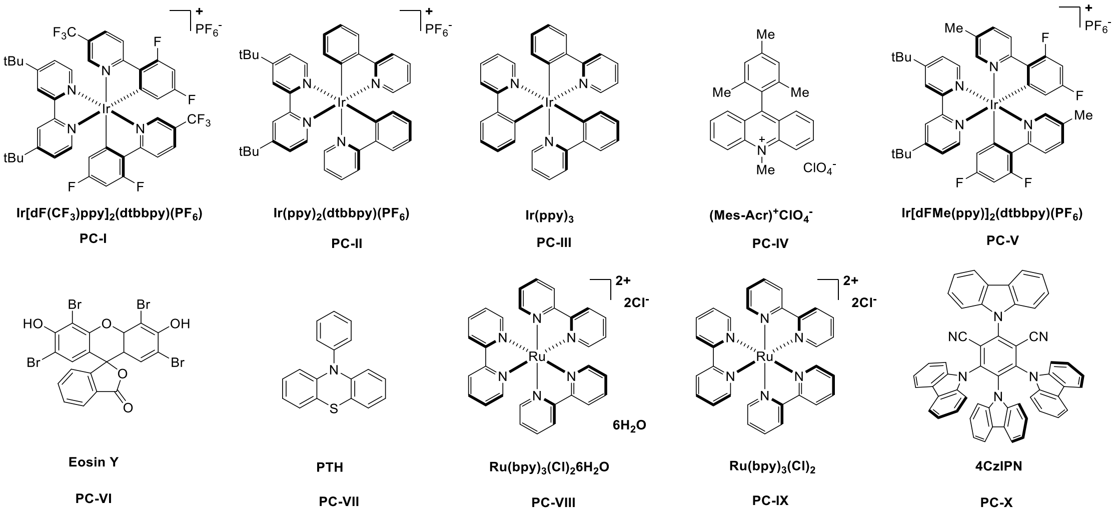

Supplementary Table 2. Screening of iron catalyst.

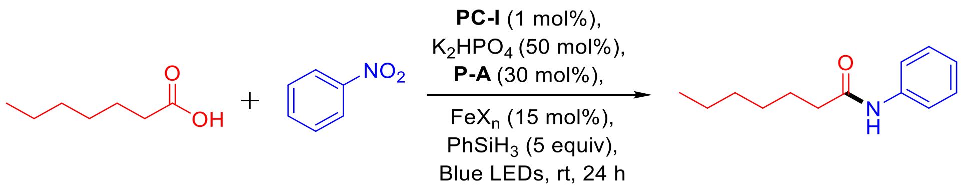

**Entry**	**Iron**	**Yield (%)**
1	FeF$_2$	trace
2	FeF$_3$	trace
3	FeCl$_2$	38
4	FeCl$_3$	trace

5	$FeCl_2 4H_2O$	36
6	$FeSO_4 7H_2O$	trace
7	$Fe(acac)_3$	trace
8	FeI_2	95
9	$Fe(OAc)_2$	trace
10	none	trace

Reaction conditions: **1a** (0.1 mmol), **2a** (0.12 mmol), **PC-I** (1 mol%), **P-A** (30 mol%), FeX_n (15 mol%), K_2HPO_4 (0.5 equiv), $PhSiH_3$ (0.5 mmol), MeCN (2 mL), Blue LEDs, ambient temperature, 24 h.

Supplementary Table 3. Screening of silanes.

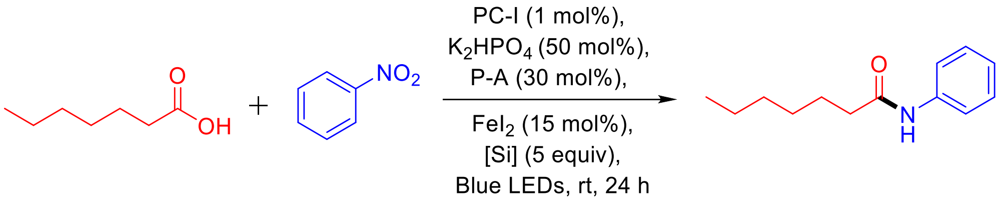

Entry	Silane	Yield (%)
1	$PhSiH_3$	95
2	$(CH_3CH_2)_2SiH_2$	10
3	$CH_3(CH_3CH_2O)_2SiH$	18
4	$CH_3(CH_3CH_2)_2SiH$	17
5	$(CH_3CH_2O)_3SiH$	17
6	Ph_2SiH_2	18
7	$(CH_3CH_2)_3SiH$	trace
8	none	n. r.

Reaction conditions: **1a** (0.1 mmol), **2a** (0.12 mmol), **PC-I** (1 mol%), **P-A** (30 mol%), FeI_2 (15 mol%), K_2HPO_4 (0.5 equiv), [Si]-H (0.5 mmol), MeCN (2 mL), Blue LEDs, ambient temperature, 24 h.

Supplementary Table 4. Screen of phosphines.

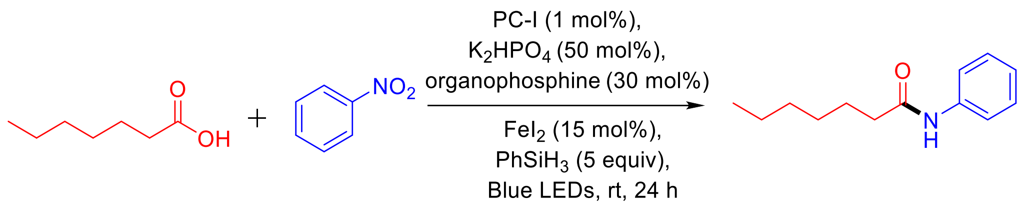

Entry	Phosphine	Yield (%)
**1**	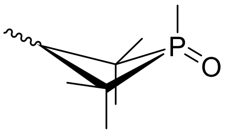 **P-A**	95
**2**	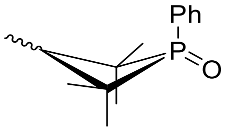 **P-B**	40
**3**	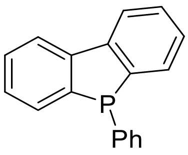 **P-C**	30
**4**	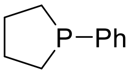 **P-D**	trace
**5**	PPh_3 **P-E**	8
**6**	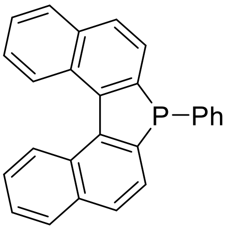 **P-F**	trace
**7**	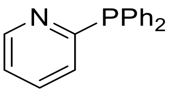 **P-G**	trace
**8**	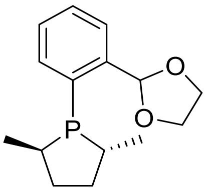 **P-H**	n. r.
**9**	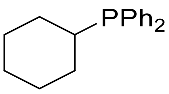 **P-I**	30

**10**	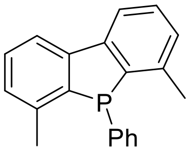 **P-J**	trace

Reaction conditions: **1a** (0.1 mmol), **2a** (0.12 mmol), **PC-I** (1 mol%), **P** (30 mol%), FeI_2 (15 mol%), K_2HPO_4 (0.5 equiv), $PhSiH_3$ (0.5 mmol), MeCN (2 mL), Blue LEDs, ambient temperature, 24 h.

3 General procedure for umpolung amidation

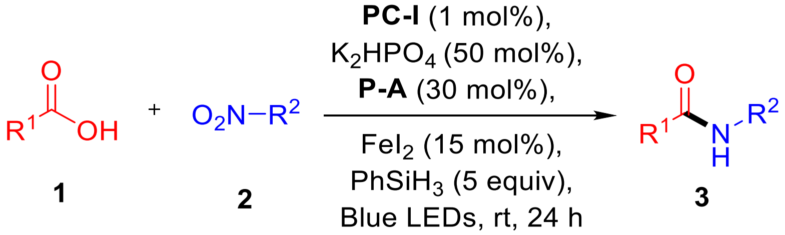

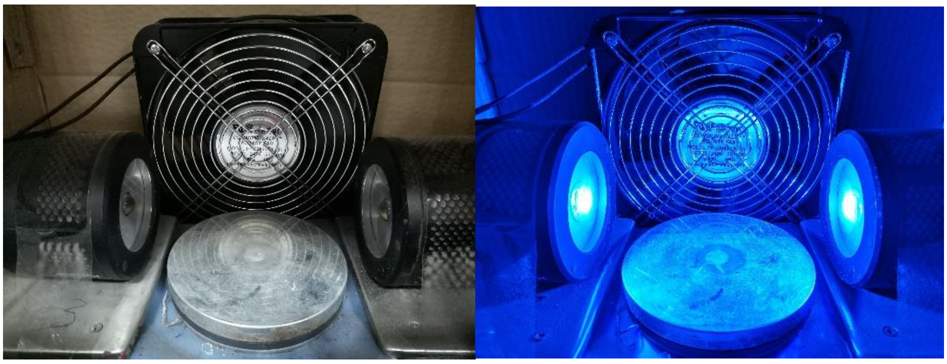

Supplementary Figure 1. Reaction set-up

0.2 mmol scale: To an 8 mL transparent vial equipped with a stirring bar, **P-A** (10.4 mg, 30 mol%), **PC-I** (2.2 mg, 1 mol%), FeI_2 (9.3 mg, 15 mol%), K_2HPO_4 (17.4 mg, 0.1 mmol) were added successively. Then the vial was carried into glovebox which was equipped with nitrogen. Then MeCN (4.0 ml), $PhSiH_3$ (1 mmol), carboxylic acids **1** (0.2 mmol) and nitroarenes **2** (0.24 mmol) were added in sequence under N_2 atmosphere. The reaction mixture was stirred under the irradiation of two 45 W blue LEDs (distance app. 10.0 cm from the bulb) at ambient temperature for 24 h. When the reaction finished, the mixture was quenched with water and extracted with ethyl acetate (3 x 10 mL). The organic layers were combined and concentrated under vacuo. The product was purified by flash column chromatography on silica gel (eluent: *n*-hexane: ethyl acetate).

Gram-scale with 0.1 mol% photocatalyst:

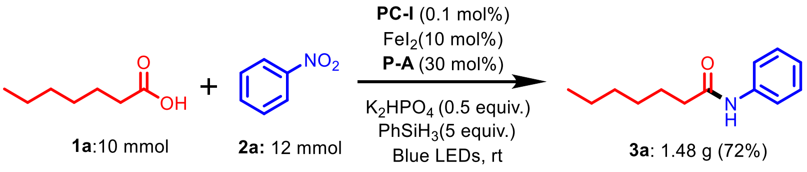

To a 250 mL round bottom flask equipped with a stirring bar, **P-A** (520 mg, 30 mol%), **PC-I** (11 mg, 0.1 mol%), FeI_2 (309 mg, 10 mol%), K_2HPO_4 (870 mg, 5 mmol) were added successively. Then the flask was carried into glovebox which was equipped with nitrogen. Then MeCN (100 ml), $PhSiH_3$ (50 mmol), carboxylic acids **1a** (10 mmol) and nitroarenes **2a** (12 mmol) were added in sequence under N_2 atmosphere. The reaction mixture was stirred under the irradiation of two 45 W blue LEDs (distance app. 10.0 cm from the bulb) at ambient temperature for 48 h. When the reaction finished, the mixture was quenched with water and extracted with ethyl acetate (3 x 10 mL). The organic layers were combined and concentrated under vacuo. The product was purified by flash column chromatography on silica gel (eluent: *n*-hexane: ethyl acetate = 5:1) to afford the product (**3a**) as 1.48 g (72%).

4. Investigation of the reaction mechanism

4.1 Radical inhibition experiments

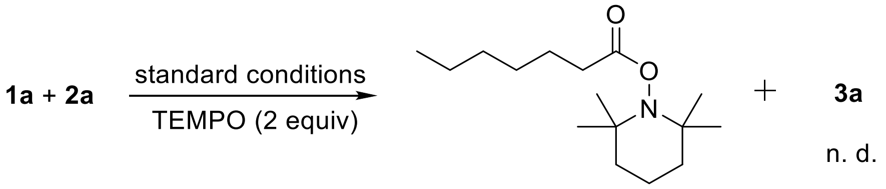

Chemical Formula (+Na+): $C_{16}H_{31}NO_2Na^+$
calculated: 292.2247
found: 292.2239

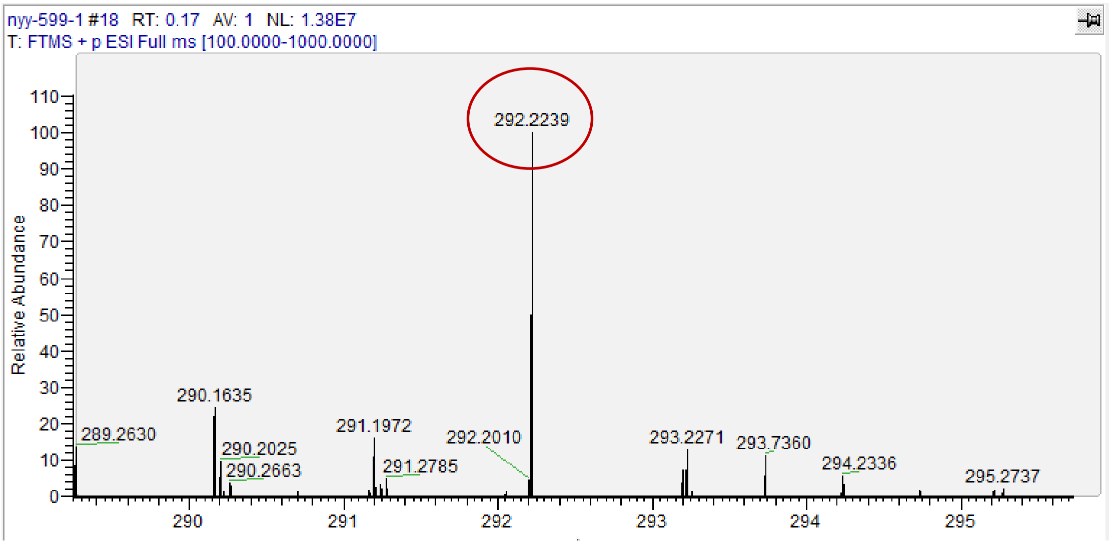

Supplementary Figure 2. The HRMS -ESI spectra

4.2 Reaction intermediate

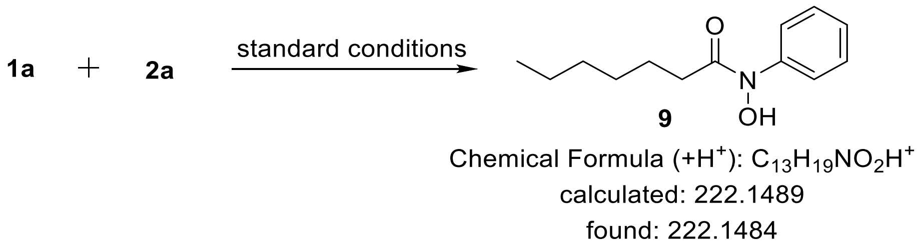

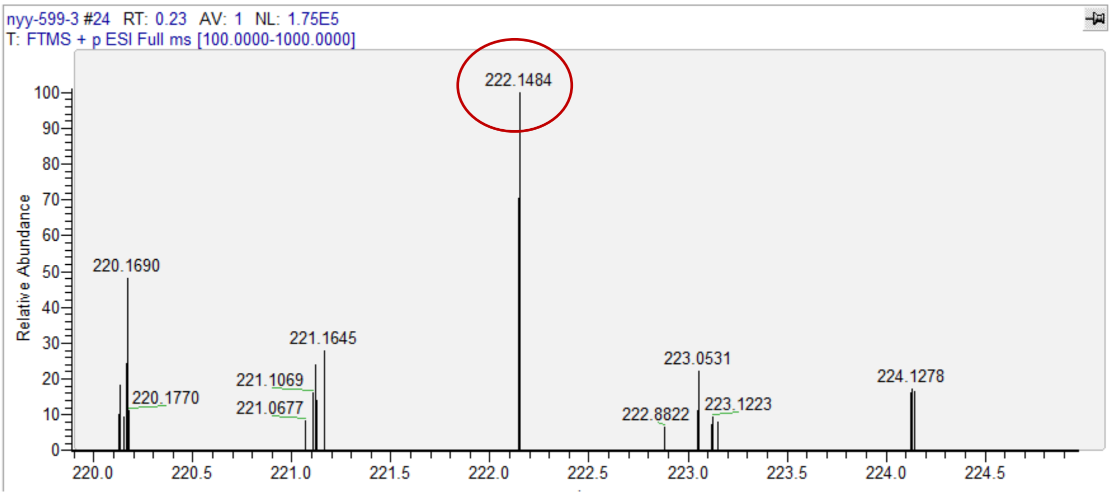

Supplementary Figure 3. The HRMS -ESI spectra

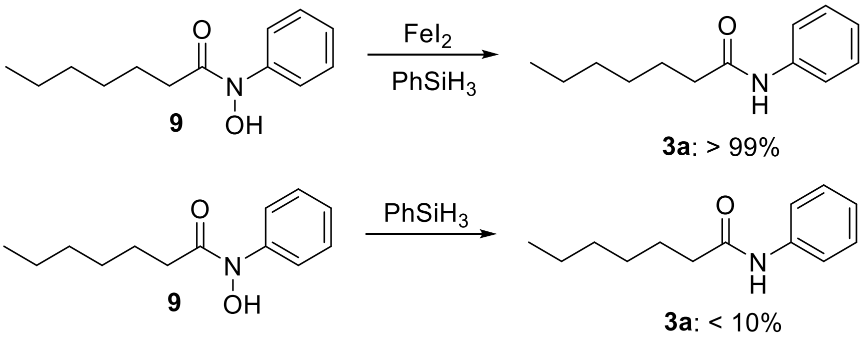

To a 4 mL transparent vial equipped with a stirring bar, **9** (22.1 mg, 0.1 mmol), FeI_2 (3.1 mg, 10 mol%) were added. Then the vial was carried into glovebox which was equipped with nitrogen. Then MeCN (2.0 ml), $PhSiH_3$ (0.5 mmol) were added in sequence under N_2 atmosphere. The reaction mixture was stirred at ambient temperature for 24 h. When the reaction finished, the mixture was quenched with water and extracted with ethyl acetate (3 x 10 mL). The organic layers were combined and concentrated under vacuo. The product was purified by flash column chromatography on silica gel (eluent: *n*-hexane: ethyl acetate) to give rise to product **3a** in quantitative yield. Meanwhile, under the same conditions but in the absence of FeI_2, the yield of product **3a** was less

than 10%, showing that iron plays an important role in product formation.

4.3 Quantum yield measurement

The quantum yield (ф) was determined by the known ferrioxalate actinometry method. A ferrioxalate actinometry solution was prepared by following the Hammond variation of the Hatchard and Parker procedure outlined in Handbook of Photochemistry. The actinometry solutions (1 mL) were irradiated with two 45 W blue LEDs for specified time intervals (0 s, 10 s, 30 s, 60 s, and 80 s). The UV-Vis spectra are shown in Fig 1. Based on the data, we got the graph (Fig 1) between the number of moles of products (y axis) and time (x axis). Then, the irradiated light intensity was estimated to 1.712 x 10^{-9} einstein S^{-1} by using $K_3[Fe(C_2O_4)_3]$ as an actinometer. For five clean tubes, according to the general procedure, the 0.1 mmol scale model reaction solution was irradiated with two 45 W blue LEDs for specified time intervals (30 min, 60 min, 90 min, 120 min and 180 min). The moles of products formed were determined by GC yield with acetophenone as reference standard. The number of moles of products (y axis) per unit time is related to the number of photons (x axis, calculated from the light intensity). The slope gives the quantum yield (Φ) of the photoreaction, 0.1389. A radical chain pathway can be basically excluded at present.

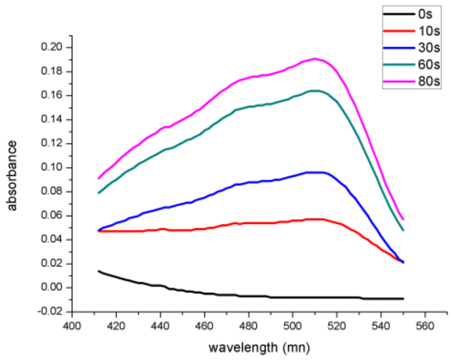

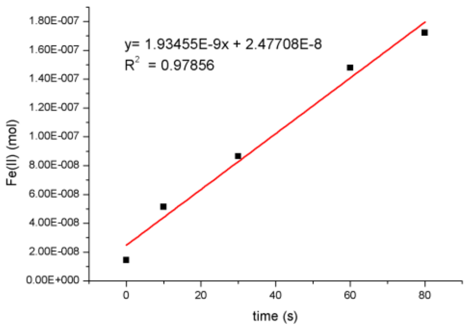

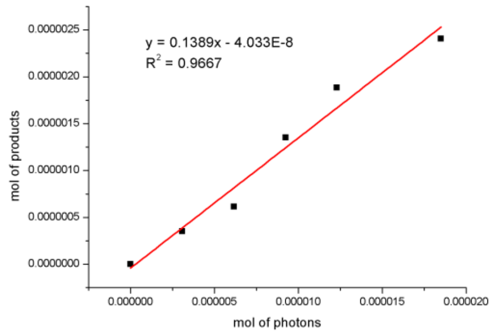

Supplementary Figure 4. The UV-Vis spectra and data of quantum yield measurement.

4.4 Luminescence quenching experiments

The luminescence quenching experiment was taken using an F-7000 FL Spectrophotometer (Hitachi, Japan). The experiments were carried out in 1 x 10^{-5} mol/L of Ir[dFCF$_3$(ppy)]$_2$(dtbbpy)PF$_6$ in CH_3CN at 25 °C. The emission intensity was collected at 475 nm. The concentrations of quencher (1a, 2a, P-A, the reduction of P-A) in CH_3CN were 1 mmol/L, 3 mmol/L, 6 mmol/L, 10 mmol/L, 20 mmol/L. Based on the data shown (Fig 2 and Fig 3), photoexcited Ir[dFCF$_3$(ppy)]$_2$(dtbbpy)PF$_6$ can be quenched by the reduction form of **P-A**.

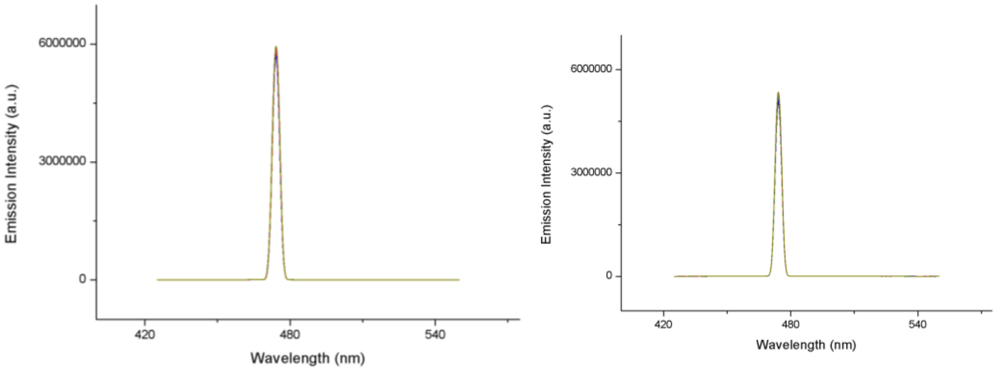

Supplementary Figure 5. The data of fluorescence quenching of Ir[dFCF$_3$(ppy)]$_2$(dtbbpy)PF$_6$ quenched by starting material **1a** (left) and **2a** (right) respectively

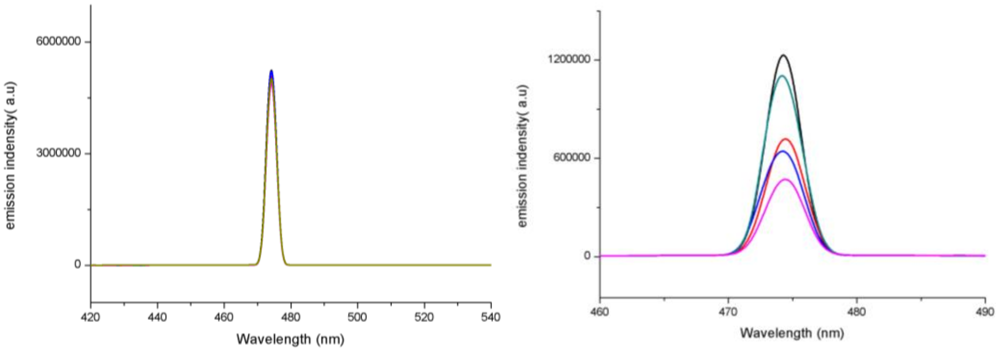

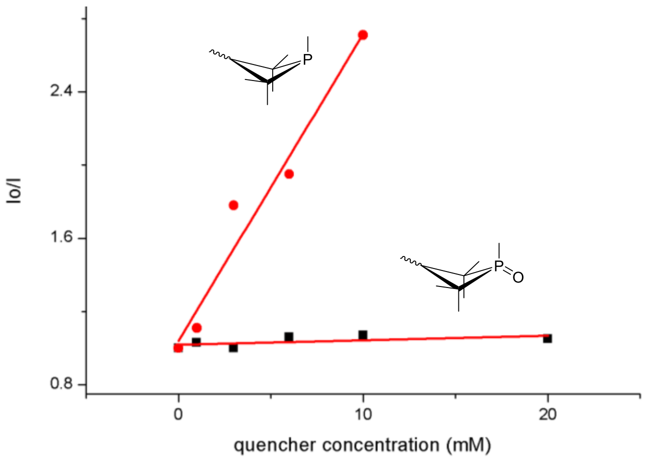

Supplementary Figure 6. The data of fluorescence quenching of $Ir[dFCF_3(ppy)]_2(dtbbpy)PF_6$ quenched by $R_3P{=}O$ (left) and R_3P (reduction in situ, right) respectively.

Footnote: 1,2,2,3,4,4-Hexamethylphosphetane prepared according to the literature procedure[3]. Because the phosphine is unstable, luminescence quenching experiment was taken immediately after in-situ synthesis in an inert gas environment.

5 Characterization of products

N-phenylheptanamide (**3a**)[4]

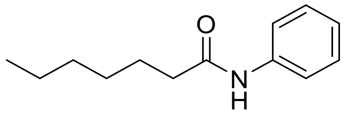

According to the general procedure in 0.2 mmol scale using 1.2 equiv. nitrobenzene with reaction time of 24 h; purified by flash chromatography (*n*-hexane / ethyl acetate = 5:1), 38.9 mg, 95% yield, white solid, m.p. = 62 – 65 °C; R_f = 0.4 (*n*-hexane / ethyl acetate = 5:1). ^1H NMR (400 MHz, Chloroform-*d*) δ 7.51 (d, *J* = 7.9 Hz, 2H), 7.31 (t, *J* = 7.9 Hz, 2H), 7.18 (s, 1H), 7.10 (t, *J* = 7.4 Hz, 1H), 2.35 (t, *J* = 7.6 Hz, 2H), 1.73 (quint, *J* = 7.5 Hz, 2H), 1.43 – 1.26 (m, 6H), 0.89 (t, *J* = 6.7 Hz, 3H). ^{13}C NMR (101 MHz, Chloroform-*d*) δ 177.0, 129.0, 124.2, 119.7, 37.9, 31.6, 28.9, 25.6, 22.5, 14.0. IR (ATR): ν = 3300, 2952, 2930, 2856, 1665, 1501, 759, 689 cm^{-1}. HRMS m/z (ESI) calcd for $C_{13}H_{20}NO$ $(M + H)^+$: 206.1539; found: 206.1538.

N-phenylisobutyramide (**3b**)[4]

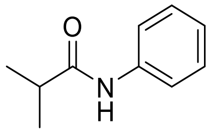

According to the general procedure in 0.2 mmol scale using 1.2 equiv. nitrobenzene with reaction

time of 24 h; purified by flash chromatography (*n*-hexane / ethyl acetate = 5:1), 23.4 mg, 72% yield, white solid, m.p. = 100 – 105 ºC; R_f = 0.6 (*n*-hexane / ethyl acetate = 5:1). ^{1}H NMR (400 MHz, Chloroform-*d*) δ 7.53 (d, *J* = 7.3 Hz, 2H), 7.44 (s, 1H), 7.30 (t, *J* = 7.4 Hz, 2H), 7.09 (t, *J* = 7.4 Hz, 1H), 2.51 (sept, *J* = 6.8 Hz, 1H), 1.24 (d, *J* = 6.9 Hz, 6H). ^{13}C NMR (101 MHz, Chloroform-*d*) δ 175.4, 138.1, 128.9, 124.1, 119.8, 36.6, 19.6. IR (ATR): ν = 3299, 2967, 2931, 2873, 2850, 1660, 1599, 753, 693 cm^{-1}. HRMS m/z (ESI) calcd for $C_{10}H_{14}NO$ $(M + H)^+$: 164.1070; found: 164.1069.

N-phenylbutyramide (**3c**)[4]

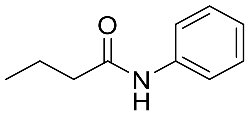

According to the general procedure in 0.2 mmol scale using 1.2 equiv. nitrobenzene with reaction time of 24 h; purified by flash chromatography (*n*-hexane / ethyl acetate = 5:1), 26.5 mg, 82% yield, white solid, m.p. = 95 – 97 ºC; R_f = 0.3 (*n*-hexane / ethyl acetate = 5:1). ^{1}H NMR (400 MHz, Chloroform-*d*) δ 7.61 (s, 1H), 7.52 (d, *J* = 7.4 Hz, 2H), 7.29 (t, *J* = 7.9 Hz, 2H), 7.08 (t, *J* = 7.4 Hz, 1H), 2.32 (t, *J* = 7.5 Hz, 2H), 1.74 (sext, *J* = 7.4 Hz, 2H), 0.98 (t, *J* = 7.4 Hz, 3H). ^{13}C NMR (101 MHz, Chloroform-*d*) δ 171.6, 138.0, 128.9, 124.1, 119.9, 39.6, 19.1, 13.7. IR (ATR): ν = 3284, 2962, 2929, 2872, 1656, 744, 691 cm^{-1}. HRMS m/z (ESI) calcd for $C_{10}H_{14}NO$ $(M + H)^+$: 164.1070; found: 164.1070.

N-phenylpentanamide (**3d**)[4]

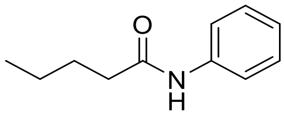

According to the general procedure in 0.2 mmol scale using 1.2 equiv. nitrobenzene with reaction time of 24 h; purified by flash chromatography (*n*-hexane / ethyl acetate = 5:1), 32.7 mg, 92% yield, white solid, m.p. = 56 – 59 ºC; R_f = 0.4 (*n*-hexane / ethyl acetate = 5:1). ^{1}H NMR (400 MHz, Chloroform-*d*) δ 7.67 (s, 1H), 7.52 (d, *J* = 7.2 Hz, 2H), 7.28 (t, *J* = 7.9 Hz, 2H), 7.08 (t, *J* = 7.9 Hz, 1H), 2.34 (t, *J* = 7.6 Hz, 2H), 1.69 (quint, *J* = 7.5 Hz, 2H), 1.38 (sext, *J* = 7.4 Hz, 2H), 0.92 (t, *J* = 7.3 Hz, 3H). ^{13}C NMR (101 MHz, Chloroform-*d*) δ 171.8, 138.0, 128.9, 124.1, 119.9, 37.4, 27.7, 22.3, 13.8. IR (ATR): ν = 3300, 2953, 2924, 2869, 1667, 1599, 752, 687 cm^{-1}. HRMS m/z (ESI) calcd for $C_{11}H_{16}NO$ $(M + H)^+$: 178.1226; found: 178.1226.

N-phenylhexanamide (**3e**)[4]

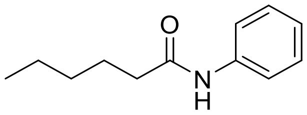

According to the general procedure in 0.2 mmol scale using 1.2 equiv. nitrobenzene with reaction time of 24 h; purified by flash chromatography (*n*-hexane / ethyl acetate = 5:1), 33.7 mg, 89% yield, white solid, m.p. = 95 – 98 ºC; R_f = 0.5 (*n*-hexane / ethyl acetate = 5:1). ^{1}H NMR (400 MHz, Chloroform-*d*) δ 7.68 (s, 1H), 7.52 (d, *J* = 7.3 Hz, 2H), 7.28 (t, *J* = 7.9 Hz, 2H), 7.08 (t, *J* = 7.4 Hz, 1H), 2.33 (t, *J* = 7.6 Hz, 2H), 1.71 (quint, *J* = 7.4 Hz, 2H), 1.37 – 1.28 (m, 4H), 0.89 (t, *J* = 7.8 Hz, 3H). ^{13}C NMR (101 MHz, Chloroform-*d*) δ 171.8, 138.0, 128.9, 124.1, 119.9, 37.7, 31.4, 25.3, 22.4, 13.9. IR (ATR): ν = 3302, 2955, 2930, 2856, 1665, 752, 689 cm^{-1}. HRMS m/z (ESI) calcd for

$C_{12}H_{18}NO$ (M + H)$^+$: 192.1383; found: 192.1381.

2-ethyl-N-phenylhexanamide (**3f**)

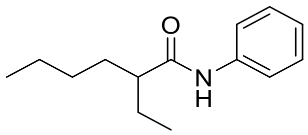

According to the general procedure in 0.2 mmol scale using 1.2 equiv. nitrobenzene with reaction time of 24 h; purified by flash chromatography (*n*-hexane / ethyl acetate = 5:1), 34.2 mg, 78% yield, white solid, m.p. = 87 – 89 °C; R_f = 0.4 (*n*-hexane / ethyl acetate = 5:1). ^{1}H NMR (400 MHz, Chloroform-*d*) δ 7.55 (d, *J* = 7.6 Hz, 2H), 7.45 (s, 1H), 7.30 (t, *J* = 7.9 Hz, 2H), 7.09 (t, *J* = 7.4 Hz, 1H), 2.16 – 2.06 (m, 1H), 1.75 – 1.65 (m, 2H), 1.60 – 1.43 (m, 2H), 1.37 – 1.25 (m, 4H), 0.95 (t, *J* = 7.4 Hz, 3H), 0.87 (t, *J* = 6.0 Hz, 3H). ^{13}C NMR (101 MHz, Chloroform-*d*) δ 174.6, 138.0, 128.9, 124.2, 120.0, 50.7, 32.6, 29.9, 26.2, 22.8, 14.0, 12.1. IR (ATR): ν = 3294, 2959, 2927, 2872, 2857, 1656, 1598, 746, 689 cm^{-1}. HRMS m/z (ESI) calcd for $C_{13}H_{20}NO$ (M + H)$^+$: 220.1696; found: 220.1693.

N-phenylpivalamide (**3g**)[4]

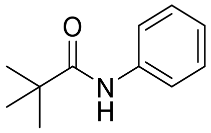

According to the general procedure in 0.2 mmol scale using 1.2 equiv. nitrobenzene with reaction time of 24 h; purified by flash chromatography (*n*-hexane / ethyl acetate = 5:1), 20.1 mg, 57% yield, white solid, m.p. = 133 – 135 °C; R_f = 0.5 (*n*-hexane / ethyl acetate = 5:1). ^{1}H NMR (400 MHz, Chloroform-*d*) δ 7.52 (d, *J* = 7.7 Hz, 2H), 7.31 (t, *J* = 7.9 Hz, 3H), 7.10 (t, *J* = 7.4 Hz, 1H), 1.31 (s, 9H). ^{13}C NMR (101 MHz, Chloroform-*d*) δ 176.6, 138.0, 128.9, 124.2, 120.0, 39.6, 27.6. IR (ATR): ν = 3311, 2965, 2871, 1654, 752, 695 cm^{-1}. HRMS m/z (ESI) calcd for $C_{11}H_{16}NO$ (M + H)$^+$: 178.1226; found: 178.1226.

6-oxo-N-phenylheptanamide (**3h**)

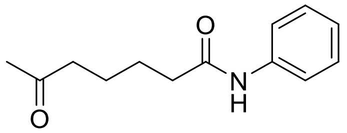

According to the general procedure in 0.2 mmol scale using 1.2 equiv. nitrobenzene with reaction time of 24 h; purified by flash chromatography (*n*-hexane / ethyl acetate = 3:1), 36.3 mg, 83% yield, white solid, m.p. = 80 – 82 °C; R_f = 0.3 (*n*-hexane / ethyl acetate = 3:1). ^{1}H NMR (400 MHz, Chloroform-*d*) δ 8.00 (s, 1H), 7.55 (d, *J* = 7.3 Hz, 2H), 7.29 (t, *J* = 8.0 Hz, 2H), 7.08 (t, *J* = 7.4 Hz, 1H), 2.48 (t, *J* = 6.7 Hz, 2H), 2.36 (t, *J* = 6.9 Hz, 2H), 2.14 (s, 3H), 1.72 – 1.58 (m, 4H). ^{13}C NMR (101 MHz, Chloroform-*d*) δ 209.3, 171.2, 138.1, 128.8, 124.1, 119.9, 43.2, 37.2, 30.0, 24.8, 23.0. IR (ATR): ν = 3337, 2944, 2921, 2870, 1704, 1662, 1598, 734, 693 cm^{-1}. HRMS m/z (ESI) calcd for $C_{13}H_{18}NO_2$ (M + H)$^+$: 220.1332; found: 220.1329.

N,4-diphenylbutanamide (**3i**)

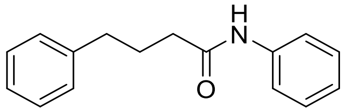

According to the general procedure in 0.2 mmol scale using 1.2 equiv. nitrobenzene with reaction time of 24 h; purified by flash chromatography (*n*-hexane / ethyl acetate = 5:1), 30.5 mg, 64% yield, white solid, m.p. = 95 – 98 °C; R_f = 0.4 (*n*-hexane / ethyl acetate = 5:1). ^{1}H NMR (400 MHz, Chloroform-*d*) δ 7.49 (d, *J* = 7.6 Hz, 2H), 7.39 (s, 1H), 7.33 – 7.23 (m, 4H), 7.20 (d, *J* = 9.3 Hz, 3H), 7.08 (t, *J* = 7.4 Hz, 1H), 2.68 (t, *J* = 7.5 Hz, 2H), 2.32 (t, *J* = 7.5 Hz, 2H), 2.04 (quint, *J* = 7.5 Hz, 2H). ^{13}C NMR (101 MHz, Chloroform-*d*) δ 171.1, 141.3, 137.9, 128.9, 128.5, 128.4, 126.0, 124.2, 119.8, 36.7, 35.0, 26.8. IR (ATR): ν = 3297, 2937, 2916, 1663, 754, 687 cm^{-1}. HRMS m/z (ESI) calcd for $C_{16}H_{17}NO$ $(M + Na)^+$: 262.1202; found: 262.1194.

N,5-diphenylpentanamide (**3j**)

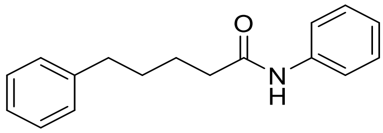

According to the general procedure in 0.2 mmol scale using 1.2 equiv. nitrobenzene with reaction time of 24 h; purified by flash chromatography (*n*-hexane / ethyl acetate = 5:1), 34.9 mg, 69% yield, white solid, m.p. = 90 – 93 °C; R_f = 0.4 (*n*-hexane / ethyl acetate = 5:1). ^{1}H NMR (400 MHz, Chloroform-*d*) δ 7.49 (d, *J* = 7.9 Hz, 2H), 7.41 (s, 1H), 7.27 (q, *J* = 7.6 Hz, 4H), 7.22 – 7.12 (m, 3H), 7.08 (t, *J* = 7.4 Hz, 1H), 2.63 (t, *J* = 7.3 Hz, 2H), 2.34 (t, *J* = 7.2 Hz, 2H), 1.79 – 1.64 (m, 4H). ^{13}C NMR (101 MHz, Chloroform-*d*) δ 171.3, 142.1, 137.9, 128.9, 128.4, 128.3, 125.8, 124.2, 119.8, 37.5, 35.6, 31.0, 25.2. IR (ATR): ν = 3294, 2983, 2932, 2858, 1658, 1599, 733, 696 cm^{-1}. HRMS m/z (ESI) calcd for $C_{17}H_{20}NO$ $(M + H)^+$: 254.1539; found: 254.1536.

3-(4-methoxyphenyl)-N-phenylpropanamide (**3k**)

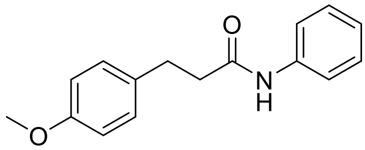

According to the general procedure in 0.2 mmol scale using 1.2 equiv. nitrobenzene with reaction time of 24 h; purified by flash chromatography (*n*-hexane / ethyl acetate = 5:1), 31.2 mg, 62% yield, white solid, m.p. = 112 – 115 °C; R_f = 0.3 (*n*-hexane / ethyl acetate = 5:1). ^{1}H NMR (400 MHz, Chloroform-*d*) δ 7.43 (d, *J* = 7.9 Hz, 2H), 7.28 (t, *J* = 7.8 Hz, 3H), 7.13 (d, *J* = 8.3 Hz, 2H), 7.08 (t, *J* = 7.4 Hz, 1H), 6.82 (d, *J* = 8.6 Hz, 2H), 3.77 (s, 3H), 2.97 (t, *J* = 7.5 Hz, 2H), 2.60 (t, *J* = 7.5 Hz, 2H). ^{13}C NMR (101 MHz, Chloroform-*d*) δ 170.6, 137.7, 134.1, 129.3, 128.9, 127.7, 124.3, 120.0, 114.0, 55.3, 39.7, 30.7. IR (ATR): ν = 3316, 2951, 2927, 2858, 2834, 1649, 1597, 814, 749, 694 cm^{-1}. HRMS m/z (ESI) calcd for $C_{16}H_{18}NO_2$ $(M + H)^+$: 256.1332; found: 256.1327.

N-phenylcyclopropanecarboxamide (**3l**)[4]

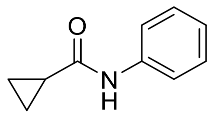

According to the general procedure in 0.2 mmol scale using 1.2 equiv. nitrobenzene with reaction time of 24 h; purified by flash chromatography (*n*-hexane / ethyl acetate = 5:1), 26.7 mg, 83% yield, white solid, m.p. = 109 – 111 ºC; R_f = 0.3 (*n*-hexane / ethyl acetate = 5:1). ^{1}H NMR (400 MHz, Chloroform-*d*) δ 7.62 (s, 1H), 7.50 (d, *J* = 7.9 Hz, 2H), 7.29 (t, *J* = 7.7 Hz, 2H), 7.08 (t, *J* = 7.4 Hz, 1H), 1.56 – 1.47 (m, 1H), 1.11 – 1.04 (m, 2H), 0.85 – 0.77 (m, 2H). ^{13}C NMR (101 MHz, Chloroform-*d*) δ 172.2, 138.1, 128.9, 124.0, 119.8, 15.6, 7.9. IR (ATR): ν = 3283, 2922, 2850, 1655, 745, 694 cm^{-1}. HRMS m/z (ESI) calcd for $C_{10}H_{12}NO$ $(M + H)^+$: 162.0913; found: 162.0911.

3-oxo-N-phenylcyclobutane-1-carboxamide (**3m**)

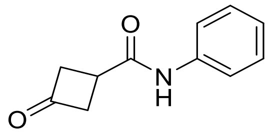

According to the general procedure in 0.2 mmol scale using 1.2 equiv. nitrobenzene with reaction time of 24 h; purified by flash chromatography (*n*-hexane / ethyl acetate = 5:1), 25.3 mg, 67% yield, white solid, m.p. = 148 – 150 ºC; R_f = 0.1 (*n*-hexane / ethyl acetate = 5:1). ^{1}H NMR (400 MHz, Chloroform-*d*) δ 7.81 (s, 1H), 7.53 (d, *J* = 7.6 Hz, 2H), 7.33 (t, *J* = 7.9 Hz, 2H), 7.13 (t, *J* = 7.4 Hz, 1H), 3.67 – 3.42 (m, 2H), 3.37 – 3.08 (m, 3H). ^{13}C NMR (101 MHz, Chloroform-*d*) δ 203.9, 171.5, 137.5, 129.1, 124.8, 120.1, 51.7, 29.6. IR (ATR): ν = 3234, 2984, 2922, 1684, 1599, 752, 691 cm^{-1}. HRMS m/z (ESI) calcd for $C_{11}H_{12}NO_2$ $(M + H)^+$: 190.0863; found: 190.0858.

N,1-diphenylcyclopentane-1-carboxamide (**3n**)

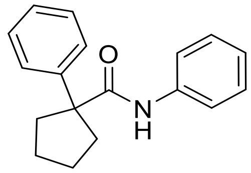

According to the general procedure in 0.2 mmol scale using 1.2 equiv. nitrobenzene with reaction time of 24 h; purified by flash chromatography (*n*-hexane / ethyl acetate = 5:1), 37.6 mg, 71% yield, white solid, m.p. = 90 – 92 ºC; R_f = 0.7 (*n*-hexane / ethyl acetate = 5:1). ^{1}H NMR (400 MHz, Chloroform-*d*) δ 7.42 (d, *J* = 7.1 Hz, 2H), 7.38 (t, *J* = 7.6 Hz, 2H), 7.33 (d, *J* = 7.6 Hz, 2H), 7.29 (t, *J* = 7.1 Hz, 1H), 7.23 (t, *J* = 7.9 Hz, 2H), 7.03 (t, *J* = 7.4 Hz, 1H), 6.84 (s, 1H), 2.62 – 2.50 (m, 2H), 2.15 – 2.05 (m, 2H), 1.95 – 1.82 (m, 2H), 1.78 – 1.66 (m, 2H). ^{13}C NMR (101 MHz, Chloroform-*d*) δ 174.5, 143.6, 138.0, 128.9, 128.7, 127.2, 126.8, 123.9, 119.5, 60.1, 36.8, 24.0. IR (ATR): ν = 3413, 2965, 2946, 2864, 1668, 1596, 745, 696 cm^{-1}. HRMS m/z (ESI) calcd for $C_{18}H_{20}NO$ $(M + H)^+$: 266.1539; found: 266.1536.

N-phenyltetrahydrofuran-2-carboxamide (**3o**)

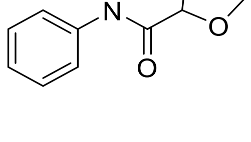

According to the general procedure in 0.2 mmol scale using 1.2 equiv. nitrobenzene with reaction time of 24 h; purified by flash chromatography (*n*-hexane / ethyl acetate = 5:1), 20.7 mg, 54% yield, white solid, m.p. = 133 – 135 ºC; R_f = 0.2 (*n*-hexane / ethyl acetate = 5:1). ^{1}H NMR (400 MHz, Chloroform-*d*) δ 8.47 (s, 1H), 7.58 (d, *J* = 7.4 Hz, 2H), 7.33 (t, *J* = 7.5 Hz, 2H), 7.12 (t, *J* = 7.5 Hz, 1H), 4.55 – 4.41 (m, 1H), 4.09 – 4.01 (m, 1H), 4.00 – 3.91 (m, 1H), 2.44 – 2.29 (m, 1H), 2.25 – 2.12 (m, 1H), 2.00 – 1.90 (m, 2H). ^{13}C NMR (101 MHz, Chloroform-*d*) δ 171.3, 137.2, 129.0, 124.4, 119.6, 78.6, 69.7, 30.2, 25.6. IR (ATR): ν = 3311, 2979, 2953, 2875, 1669, 1597, 1237, 752, 692 cm^{-1}. HRMS m/z (ESI) calcd for $C_{11}H_{14}NO_2$ $(M + H)^+$: 192.1019; found: 192.1018.

N-phenylcyclohex-1-ene-1-carboxamide (**3p**)

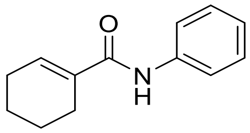

According to the general procedure in 0.2 mmol scale using 1.2 equiv. nitrobenzene with reaction time of 24 h; purified by flash chromatography (*n*-hexane / ethyl acetate = 5:1), 23.7 mg, 59% yield, white solid, m.p. = 109 – 112 ºC; R_f = 0.6 (*n*-hexane / ethyl acetate = 5:1). ^{1}H NMR (400 MHz, Chloroform-*d*) δ 7.55 (d, *J* = 7.3 Hz, 2H), 7.43 (s, 1H), 7.30 (t, *J* = 8.0, 2H), 7.09 (t, *J* = 8.0, 1H), 6.75 – 6.69 (m 1H), 2.25 – 2.19 (m, 2H), 2.38 – 2.32 (m, 2H), 1.77 – 1.70 (m, 2H), 1.67 – 1.61 (m, 2H). ^{13}C NMR (101 MHz, Chloroform-*d*) δ 166.8, 142.0, 138.1, 134.2, 129.0, 124.1, 120.0, 25.5, 24.4, 22.1, 21.5. IR (ATR): ν = 3246, 2924, 2856, 1629, 1595, 848, 800 cm^{-1}. HRMS m/z (ESI) calcd for $C_{13}H_{16}NO$ $(M + H)^+$: 202.1226; found: 202.1226.

2-cyclohexyl-N-phenylacetamide (**3q**)

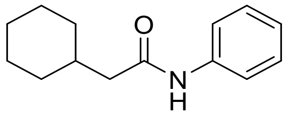

According to the general procedure in 0.2 mmol scale using 1.2 equiv. nitrobenzene with reaction time of 24 h; purified by flash chromatography (*n*-hexane / ethyl acetate = 5:1), 41.4 mg, 95% yield, white solid, m.p. = 64 – 66 ºC; R_f = 0.4 (*n*-hexane / ethyl acetate = 5:1). ^{1}H NMR (400 MHz, Chloroform-*d*) δ 7.58 (s, 1H), 7.53 (d, *J* = 8.0 Hz, 2H), 7.29 (t, *J* = 7.8 Hz, 2H), 7.08 (t, *J* = 7.4 Hz, 1H), 2.21 (d, *J* = 7.1 Hz, 2H), 1.94 – 1.83 (m, 1H), 1.82 – 1.74 (m, 2H), 1.73 – 1.61 (m, 3H), 1.33 – 1.20 (m, 2H), 1.19 – 1.06 (m, 1H), 1.05 – 0.92 (m, 2H). ^{13}C NMR (101 MHz, Chloroform-*d*) δ 171.0, 138.0, 128.9, 124.1, 119.9, 45.8, 35.5, 33.1, 26.1, 26.0. IR (ATR): ν = 3239, 2918, 2849, 1649, 1595, 756, 694 cm^{-1}. HRMS m/z (ESI) calcd for $C_{14}H_{20}NO$ $(M + H)^+$: 218.1539; found: 218.1535.

N-phenylbicyclo[2.2.1]hept-5-ene-2-carboxamide (**3r**)

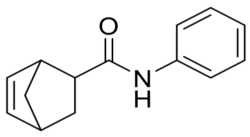

According to the general procedure in 0.2 mmol scale using 1.2 equiv. nitrobenzene with reaction time of 24 h; purified by flash chromatography (*n*-hexane / ethyl acetate = 5:1), 21 mg, 49% yield,

white solid, m.p. = 135 – 138 °C; R_f = 0.4 (*n*-hexane / ethyl acetate = 5:1). ^{1}H NMR (400 MHz, Chloroform-*d*) δ 7.53 (d, *J* = 7.5 Hz, 2H), 7.31 (t, *J* = 7.9 Hz, 2H), 7.25 (s, 1H), 7.09 (t, *J* = 7.4 Hz, 1H), 6.19 (dd, *J* = 5.7, 2.9 Hz, 1H), 6.14 (dd, *J* = 5.7, 3.0 Hz, 1H), 3.05 (s, 1H), 2.97 (s, 1H), 2.20 – 2.12 (m, 1H), 2.08 – 2.00 (m, 1H), 1.78 (d, *J* = 8.3 Hz, 1H), 1.44 – 1.36 (m, 2H). ^{13}C NMR (101 MHz, Chloroform-*d*) δ 173.9, 138.6, 138.2, 135.9, 129.0, 124.0, 119.6, 47.3, 46.3, 45.9, 41.6, 30.6. IR (ATR): ν = 3297, 2973, 2943, 1658, 1540, 756, 692 cm^{-1}. HRMS m/z (ESI) calcd for $C_{14}H_{16}NO$ $(M + H)^+$: 214.1226; found: 214.1211.

5-oxo-N,5-diphenylpentanamide (**3s**)

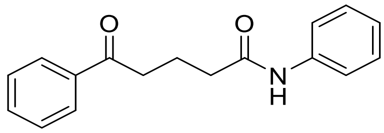

According to the general procedure in 0.2 mmol scale using 1.2 equiv. nitrobenzene with reaction time of 24 h; purified by flash chromatography (*n*-hexane / ethyl acetate = 5:1), 38.5 mg, 72% yield, white solid, m.p. = 118 – 120 °C; R_f = 0.1 (*n*-hexane / ethyl acetate = 5:1). ^{1}H NMR (400 MHz, Chloroform-*d*) δ 7.96 (d, *J* = 7.0 Hz, 2H), 7.79 (s, 1H), 7.59 – 7.49 (m, 3H), 7.45 (t, *J* = 7.6 Hz, 2H), 7.30 (t, *J* = 7.9 Hz, 2H), 7.08 (t, *J* = 7.4 Hz, 1H), 3.11 (t, *J* = 6.8 Hz, 2H), 2.47 (t, *J* = 7.2 Hz, 2H), 2.16 (quint, *J* = 7.0 Hz, 2H). ^{13}C NMR (101 MHz, Chloroform-*d*) δ 200.2, 171.0, 137.9, 136.6, 133.3, 128.9, 128.6, 128.1, 124.2, 119.8, 37.3, 36.4, 20.1. IR (ATR): ν = 3364, 2918, 2850, 1673, 1596, 752, 689 cm^{-1}. HRMS m/z (ESI) calcd for $C_{17}H_{18}NO_2$ $(M + H)^+$: 268.1332; found: 268.1325.

(E)-N-phenylhex-2-enamide (**3t**)

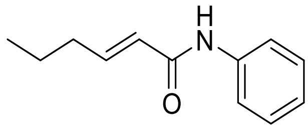

According to the general procedure in 0.2 mmol scale using 1.2 equiv. nitrobenzene with reaction time of 24 h; purified by flash chromatography (*n*-hexane / ethyl acetate = 5:1), 30.1 mg, 79% yield (*E/Z* = 4.6:1), white solid, m.p. = 112 – 115 °C; R_f = 0.3 (*n*-hexane / ethyl acetate = 5:1). ^{1}H NMR (400 MHz, Chloroform-*d*) δ 7.56 (d, *J* = 8.0 Hz, 2H), 7.37 (s, 1H), 7.30 (t, *J* = 8.0 Hz, 2H), 7.10 (t, *J* = 7.8 Hz, 1H), 7.06 – 6.93 (m, 1H), 5.97 – 5.80 (m, 1H), 2.25 – 2.15 (m, 2H), 1.56 – 1.44 (m, 2H), 0.94 (t, *J* = 7.4 Hz, 3H). ^{13}C NMR (101 MHz, Chloroform-*d*) δ 170.5, 151.7, 146.4, 138.0, 129.0, 124.3, 124.0, 120.7, 119.9, 34.2, 21.5, 13.7. IR (ATR): ν = 3261, 3080, 2958, 2929, 2872, 1666, 1600, 753, 688 cm^{-1}. HRMS m/z (ESI) calcd for $C_{12}H_{16}NO$ $(M + H)^+$: 190.1226; found: 190.1225.

N-phenylundec-10-enamide (**3u**)

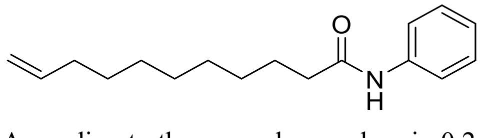

According to the general procedure in 0.2 mmol scale using 1.2 equiv. nitrobenzene with reaction time of 24 h; purified by flash chromatography (*n*-hexane / ethyl acetate = 5:1), 46.9 mg, 90% yield, white solid, m.p. = 64 – 65 °C; R_f = 0.5 (*n*-hexane / ethyl acetate = 5:1). ^{1}H NMR (400 MHz, Chloroform-*d*) δ 7.51 (d, *J* = 7.9 Hz, 2H), 7.34 (s, 1H), 7.30 (t, *J* = 7.8 Hz, 2H), 7.09 (t, *J* = 7.4 Hz,

1H), 5.88 – 5.73(m, 1H), 5.04 – 4.89 (m, 2H), 2.34 (t, *J* = 7.6 Hz, 2H), 2.03 (q, *J* = 7.0 Hz, 2H), 1.74 – 1.69 (m, 2H), 1.38 – 1.26 (m, 10H). ^{13}C NMR (101 MHz, Chloroform-*d*) δ 171.5, 139.1, 138.0, 128.9, 124.1, 119.8, 114.1, 37.8, 33.8, 29.3, 29.3, 29.2, 29.1, 28.9, 25.6. IR (ATR): ν = 3304, 3075, 2961, 2918, 2849, 1662, 1603, 742, 690 cm^{-1}. HRMS m/z (ESI) calcd for $C_{17}H_{26}NO$ (M + H)$^+$: 260.2009; found: 260.2007.

2,2-dicyclohexyl-N-phenylacetamide (**3v**)

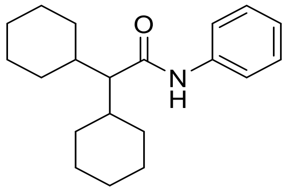

According to the general procedure in 0.2 mmol scale using 1.2 equiv. nitrobenzene with reaction time of 24 h; purified by flash chromatography (*n*-hexane / ethyl acetate = 5:1), 31.2 mg, 53% yield, white solid, m.p. = 99 – 101 °C; R_f = 0.6 (*n*-hexane / ethyl acetate = 5:1). ^{1}H NMR (400 MHz, Chloroform-*d*) δ 7.52 (d, *J* = 7.5 Hz, 2H), 7.31 (t, *J* = 8.0 Hz, 2H), 7.13 (s, 1H), 7.09 (t, *J* = 7.4 Hz, 1H), 1.80 – 1.60 (m, 12H), 1.35-1.10 (m, 9H), 1.05-0.97 (m, 2H). ^{13}C NMR (101 MHz, Chloroform-*d*) δ 172.9, 137.8, 128.9, 124.1, 120.0, 60.6, 36.8, 31.6, 29.6, 26.5. IR (ATR): ν = 3328, 2927, 2847, 1653, 750, 691 cm^{-1}. HRMS m/z (ESI) calcd for $C_{20}H_{30}NO$ (M + H)$^+$: 300.2322; found: 300.2315.

N-phenylhept-6-ynamide (**3w**)

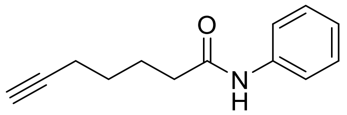

According to the general procedure in 0.2 mmol scale using 1.2 equiv. nitrobenzene with reaction time of 24 h; purified by flash chromatography (*n*-hexane / ethyl acetate = 5:1), 28.3 mg, 70% yield, white solid, m.p. = 70 – 72 °C; R_f = 0.4 (*n*-hexane / ethyl acetate = 5:1). ^{1}H NMR (400 MHz, Chloroform-*d*) δ 7.51 (d, *J* = 7.7 Hz, 3H), 7.30 (t, *J* = 7.9 Hz, 2H), 7.09 (t, *J* = 7.4 Hz, 1H), 2.37 (t, *J* = 7.5 Hz, 2H), 2.27 – 2.18 (m, 2H), 1.97 (t, *J* = 2.6 Hz, 1H), 1.89 – 1.78 (m, 2H), 1.60 (quint, *J* = 7.1 Hz, 2H). ^{13}C NMR (101 MHz, Chloroform-*d*) δ 171.1, 137.9, 128.9, 124.2, 119.9, 84.0, 68.7, 37.0, 27.8, 24.6, 18.2. IR (ATR): ν = 3309, 3278, 2950, 2858, 1659, 1597, 742, 692 cm^{-1}. HRMS m/z (ESI) calcd for $C_{13}H_{16}NO$ (M + H)$^+$: 202.1226; found: 202.1225.

1-Adamantanecarboxylic acid (**3x**)[4]

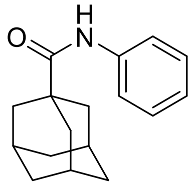

According to the general procedure in 0.2 mmol scale using 1.2 equiv. nitrobenzene with reaction time of 24 h; purified by flash chromatography (*n*-hexane / ethyl acetate = 5:1), 39.8 mg, 78% yield, white solid, m.p. = 194 – 196 °C; R_f = 0.7 (*n*-hexane / ethyl acetate = 5:1). ^{1}H NMR (400 MHz, Chloroform-*d*) δ 7.53 (d, *J* = 7.6 Hz, 2H), 7.31 (t, *J* = 7.9 Hz, 3H), 7.09 (t, *J* = 7.4 Hz, 1H), 2.13 – 2.06 (m, 3H), 1.97 (d, *J* = 2.8 Hz, 6H), 1.80 – 1.72 (m, 6H). ^{13}C NMR (101 MHz, Chloroform-*d*) δ

176.1, 138.0, 128.9, 124.1, 119.9, 41.5, 40.4, 39.3, 38.6, 36.4, 28.1. IR (ATR): ν = 3276, 2898, 2849, 1643, 1596, 755, 694 cm^{-1}. HRMS m/z (ESI) calcd for $C_{17}H_{22}NO$ $(M + H)^+$: 256.1696; found: 256.1691.

3-(2-iodophenyl)-N-phenylpropanamide (**3y**)

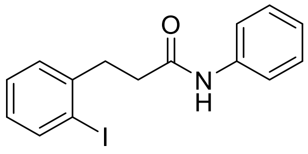

According to the general procedure in 0.2 mmol scale using 1.2 equiv. nitrobenzene with reaction time of 24 h; purified by flash chromatography (*n*-hexane / ethyl acetate = 5:1), 47.5 mg, 67% yield, white solid, m.p. = 101 – 103 °C; R_f = 0.4 (*n*-hexane / ethyl acetate = 5:1). ^{1}H NMR (400 MHz, Chloroform-*d*) δ 7.80 (d, *J* = 7.8 Hz, 1H), 7.46 (d, *J* = 8.0 Hz, 2H), 7.41 (s, 1H), 7.33 – 7.23 (m, 4H), 7.08 (t, *J* = 7.4 Hz, 1H), 6.94 – 6.84 (m, 1H), 3.13 (t, *J* = 7.7 Hz, 2H), 2.62 (t, *J* = 7.7 Hz, 2H). ^{13}C NMR (101 MHz, Chloroform-*d*) δ 170.0, 143.0, 139.5, 137.7, 129.8, 128.9, 128.6, 128.3, 124.3, 120.0, 100.3, 37.7, 36.5. IR (ATR): ν = 3282, 2962, 2931, 2852, 1644, 1496, 753, 694 cm^{-1}. HRMS m/z (ESI) calcd for $C_{15}H_{15}INO$ $(M + H)^+$: 352.0193; found: 352.0188.

(1S,4R)-4,7,7-trimethyl-3-oxo-N-phenyl-2-oxabicyclo[2.2.1]heptane-1-carboxamide (**3z**)

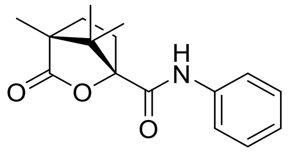

According to the general procedure in 0.2 mmol scale using 1.2 equiv. nitrobenzene with reaction time of 24 h; purified by flash chromatography (*n*-hexane / ethyl acetate = 5:1), 41 mg, 75% yield, white solid, m.p. = 98 – 100 °C; R_f = 0.4 (*n*-hexane / ethyl acetate = 5:1). ^{1}H NMR (400 MHz, Chloroform-*d*) δ 8.15 (s, 1H), 7.58 (d, *J* = 7.6 Hz, 2H), 7.35 (t, *J* = 7.8 Hz, 2H), 7.16 (t, *J* = 7.4 Hz, 1H), 2.67 – 2.56 (m, 1H), 2.05 – 1.95 (m, 2H), 1.79 – 1.69 (m, 1H), 1.17 (s, 3H), 1.15 (s, 3H), 0.99 (s, 3H). ^{13}C NMR (101 MHz, Chloroform-*d*) δ 178.0, 165.2, 136.7, 129.1, 125.0, 120.0, 92.4, 55.5, 54.4, 30.5, 29.1, 16.7, 16.6, 9.7. IR (ATR): ν = 3285, 2966, 2937, 1783, 1667, 1597, 758, 693 cm^{-1}. HRMS m/z (ESI) calcd for $C_{16}H_{20}NO_3$ $(M + H)^+$: 274.1438; found: 274.1432.

(S)-2-(6-methoxynaphthalen-2-yl)-N-phenylpropanamide (**3aa**)

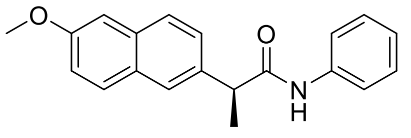

According to the general procedure in 0.2 mmol scale using 1.2 equiv. nitrobenzene with reaction time of 40 h; purified by flash chromatography (*n*-hexane / ethyl acetate = 3:1), 28.7 mg, 47% yield, white solid, m.p. = 130 – 133 °C; R_f = 0.5 (*n*-hexane / ethyl acetate = 3:1). ^{1}H NMR (400 MHz, Chloroform-*d*) δ 7.73 (t, *J* = 9.5 Hz, 3H), 7.46 – 7.41 (m, 1H), 7.42 – 7.36 (m, 2H), 7.28 – 7.20 (m, 2H), 7.21 – 7.09 (m, 3H), 7.04 (t, *J* = 7.4 Hz, 1H), 3.92 (s, 3H), 3.85 (q, *J* = 7.1 Hz, 1H), 1.67 (d, *J* = 7.1 Hz, 3H). ^{13}C NMR (101 MHz, Chloroform-*d*) δ 172.5, 157.9, 137.8, 135.9, 133.9, 129.3, 129.0, 128.9, 127.9, 126.3, 126.1, 124.2, 119.7, 119.3, 105.7, 55.3, 48.1, 18.5. IR (ATR): ν = 3304, 2996,

2963, 2928, 2852, 1656, 1599, 731, 689 cm^{-1}. HRMS m/z (ESI) calcd for $C_{20}H_{20}NO_2$ $(M + H)^+$: 306.1489; found: 306.1482.

2-((1r,3s,5R,7S)-3-hydroxyadamantan-1-yl)-N-phenylacetamide (**3bb**)

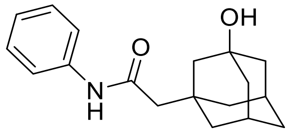

According to the general procedure in 0.2 mmol scale using 1.2 equiv. nitrobenzene with reaction time of 24 h; purified by flash chromatography (*n*-hexane / ethyl acetate = 5:1), 35.7 mg, 63% yield, white solid, m.p. = 164 – 166 °C; R_f = 0.4 (*n*-hexane / ethyl acetate = 5:1). ^{1}H NMR (400 MHz, Methanol-*d*$_4$) δ 7.53 (d, *J* = 8.3 Hz, 2H), 7.29 (t, *J* = 8.0 Hz, 2H), 7.08 (t, *J* = 7.4 Hz, 1H), 4.87 (s, 2H), 2.18 (s, 2H), 2.17 (s, 1H), 1.71 – 1.55 (m, 12H). ^{13}C NMR (101 MHz, Methanol-*d*$_4$) δ 172.1, 139.7, 129.7, 125.2, 121.4, 69.2, 51.5, 50.8, 45.2, 42.4, 37.5, 36.4, 32.1. IR (ATR): ν = 3353, 2908, 2850, 1647, 752, 692 cm^{-1}. HRMS m/z (ESI) calcd for $C_{18}H_{24}NO_2$ $(M + H)^+$: 286.1802; found: 286.1801.

tert-butyl (S)-(1-oxo-3-phenyl-1-(phenylamino)propan-2-yl)carbamate (**3cc**)

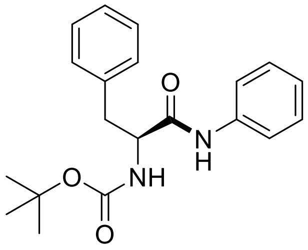

According to the general procedure in 0.2 mmol scale using 1.5 equiv. nitrobenzene with reaction time of 48 h; purified by flash chromatography (*n*-hexane / ethyl acetate = 3:1), 37.4 mg, 55% yield, white solid, m.p. = 140 – 142 °C; R_f = 0.4 (*n*-hexane / ethyl acetate = 3:1). ^{1}H NMR (400 MHz, Chloroform-*d*) δ 7.74 (s, 1H), 7.40 – 7.33 (m, 2H), 7.32 – 7.28 (m, 3H), 7.28 – 7.22 (m, 4H), 7.13 – 7.06 (m, 1H), 5.17 (s, 1H), 4.51 – 4.40 (m, 1H), 3.14 (d, *J* = 7.1 Hz, 2H), 1.42 (s, 9H). ^{13}C NMR (101 MHz, Chloroform-*d*) δ 169.6, 155.8, 137.3, 136.6, 129.3, 128.9, 128.7, 127.0, 124.5, 120.1, 80.5, 56.6, 38.5, 28.3. IR (ATR): ν = 3261, 2977, 2927, 2855, 1658, 1603, 749, 692 cm^{-1}. HRMS m/z (ESI) calcd for $C_{20}H_{25}N_2O_3$ $(M + H)^+$: 341.1860; found: 341.1852.

tert-butyl (R)-2-(phenylcarbamoyl)pyrrolidine-1-carboxylate (**3dd**)

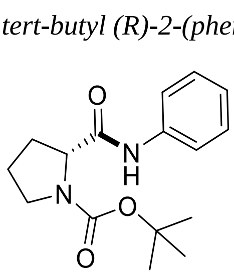

According to the general procedure in 0.2 mmol scale using 1.2 equiv. nitrobenzene with reaction time of 24 h; purified by flash chromatography (*n*-hexane / ethyl acetate = 3:1), 30.2 mg, 52% yield, white solid, m.p. = 188 – 190 °C; R_f = 0.4 (*n*-hexane / ethyl acetate = 3:1). ^{1}H NMR (400 MHz,

Chloroform-*d*) δ 9.47 (s, 1H), 7.52 (d, *J* = 7.7 Hz, 2H), 7.30 (t, *J* = 7.5 Hz, 2H), 7.16 – 6.99 (m, 1H), 4.47 (s, 1H), 3.80 – 3.15 (m, 2H), 2.40 – 1.70 (m, 4H), 1.49 (s, 9H). ^{13}C NMR (101 MHz, Chloroform-*d*) δ 170.0, 156.6, 138.4, 128.9, 123.9, 119.7, 80.9, 60.5, 47.2, 32.7, 28.4, 27.1. IR (ATR): ν = 3288, 2975, 2930, 2877, 1695, 1668, 1601, 755, 693 cm^{-1}. HRMS m/z (ESI) calcd for $C_{16}H_{22}N_2NaO_3$ (M + Na)$^+$: 313.1523; found: 313.1510.

N-phenyl-2-(thiophen-2-yl)acetamide (**3ee**)

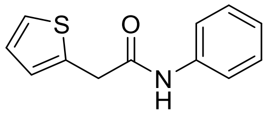

According to the general procedure in 0.2 mmol scale using 1.2 equiv. nitrobenzene with reaction time of 24 h; purified by flash chromatography (*n*-hexane / ethyl acetate = 5:1), 24.3 mg, 56% yield, white solid, m.p. = 117 – 120 °C; R_f = 0.3 (*n*-hexane / ethyl acetate = 5:1). ^{1}H NMR (400 MHz, Chloroform-*d*) δ 7.43 (d, *J* = 7.7 Hz, 2H), 7.38 (s, 1H), 7.30 (d, *J* = 7.0 Hz, 2H), 7.26 (d, *J* = 6.4 Hz, 1H), 7.10 (t, *J* = 7.4 Hz, 1H), 7.04 (d, *J* = 4.8 Hz, 2H), 3.94 (s, 2H). ^{13}C NMR (101 MHz, Chloroform-*d*) δ 167.9, 137.4, 134.1, 129.0, 127.8, 127.6, 126.1, 124.6, 119.9, 38.5. IR (ATR): ν = 3258, 2921, 1704, 1485, 757, 690 cm^{-1}. HRMS m/z (ESI) calcd for $C_{12}H_{12}NOS$ (M + H)$^+$: 218.0634; found: 218.0631.

N-phenylfuran-2-carboxamide (**3ff**)[4]

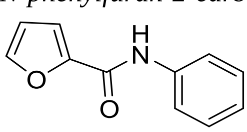

According to the general procedure in 0.2 mmol scale using 1.2 equiv. nitrobenzene with reaction time of 24 h; purified by flash chromatography (*n*-hexane / ethyl acetate = 5:1), 19.2 mg, 52% yield, white solid, m.p. = 120 – 123 °C; R_f = 0.4 (*n*-hexane / ethyl acetate = 5:1). ^{1}H NMR (400 MHz, Chloroform-*d*) δ 8.12 (s, 1H), 7.65 (dd, *J* = 8.6, 1.1 Hz, 2H), 7.50 (dd, *J* = 1.7, 0.8 Hz, 1H), 7.6 (t, *J* = 7.5 Hz, 2H), 7.23 (dd, *J* = 3.5, 0.8 Hz, 1H), 7.14 (*J* = 7.5 Hz, 1.1 Hz, 1H), 6.55 (dd, *J* = 3.5, 1.8 Hz, 1H). ^{113}C NMR (101 MHz, Chloroform-*d*) δ 156.1, 147.8, 144.2, 137.3, 129.1, 124.5, 119.9, 115.2, 112.6. IR (ATR): ν = 3278, 1651, 1596, 1226, 748, 691 cm^{-1}. HRMS m/z (ESI) calcd for $C_{11}H_{10}NO_2$ (M + H)$^+$: 188.0706; found: 188.0706.

2-bromo-N-phenylbenzamide (**3gg**)

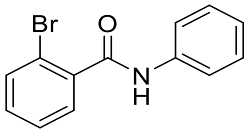

According to the general procedure in 0.2 mmol scale using 1.2 equiv. nitrobenzene with reaction time of 24 h; purified by flash chromatography (*n*-hexane / ethyl acetate = 5:1), 28.7 mg, 52% yield, white solid, m.p. = 125 – 127 °C; R_f = 0.5 (*n*-hexane / ethyl acetate = 5:1). ^{1}H NMR (400 MHz, Chloroform-*d*) δ 7.78 (s, 1H), 7.67 – 7.58 (m, 4H), 7.38 (q, *J* = 8.2, 7.3 Hz, 3H), 7.31 (td, *J* = 7.7, 1.8 Hz, 1H), 7.17 (t, *J* = 7.4 Hz, 1H). ^{13}C NMR (101 MHz, Chloroform-*d*) δ 165.5, 137.8, 137.5, 133.5, 131.6, 129.8, 129.1, 127.7, 124.9, 120.1, 119.2. IR (ATR): ν = 3252, 1656, 1597, 745, 687, 587 cm^{-1}. HRMS m/z (ESI) calcd for $C_{13}H_{11}BrNO$ (M + H)$^+$: 276.0019; found: 276.0016.

N-phenylquinoline-6-carboxamide (**3hh**)

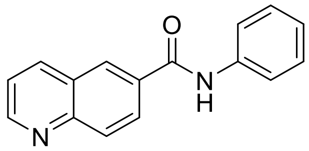

According to the general procedure in 0.2 mmol scale using 1.2 equiv. nitrobenzene with reaction time of 24 h; purified by flash chromatography (*n*-hexane / ethyl acetate = 5:1), 32.7 mg, 66% yield, white solid, m.p. = 156 – 158 °C; R_f = 0.4 (*n*-hexane / ethyl acetate = 5:1). ^{1}H NMR (400 MHz, Chloroform-*d*) δ 8.96 (dd, *J* = 4.2, 1.7 Hz, 1H), 8.40 (s, 1H), 8.33 (s, 1H), 8.16 (dd, *J* = 8.3, 1.7 Hz, 1H), 8.10 (d, *J* = 1.8 Hz, 2H), 7.70 (d, *J* = 7.3 Hz, 2H), 7.46 – 7.41 (m, 1H), 7.37 (t, *J* = 7.3 Hz, 2H), 7.17 (t, *J* = 7.4 Hz, 1H). ^{13}C NMR (101 MHz, Chloroform-*d*) δ 165.4, 152.0, 149.2, 137.9, 137.1, 132.9, 130.0, 129.1, 127.9, 127.5, 127.2, 124.8, 122.0, 120.5. IR (ATR): ν = 3201, 1690, 1633, 1601, 1571, 749, 687 cm^{-1}. HRMS m/z (ESI) calcd for $C_{16}H_{13}N_2O$ (M + H)$^+$: 249.1022; found: 249.1018.

4-ethynyl-N-phenylbenzamide (**3ii**)

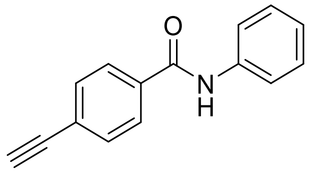

According to the general procedure in 0.2 mmol scale using 1.2 equiv. nitrobenzene with reaction time of 24 h; purified by flash chromatography (*n*-hexane / ethyl acetate = 5:1), 25.3 mg, 57% yield, white solid, m.p. = 158 – 160 °C; R_f = 0.6 (*n*-hexane / ethyl acetate = 5:1). ^{1}H NMR (400 MHz, Chloroform-*d*) δ 7.91 (s, 1H), 7.82 (d, *J* = 8.4 Hz, 2H), 7.63 (d, *J* = 7.4 Hz, 2H), 7.58 (d, *J* = 8.3 Hz, 2H), 7.37 (t, *J* = 8.4 Hz, 2H), 7.16 (t, *J* = 7.4 Hz, 1H), 3.23 (s, 1H). ^{13}C NMR (101 MHz, Chloroform-*d*) δ 164.9, 137.7, 134.9, 132.4, 129.1, 127.0, 125.7, 124.8, 120.3, 82.6, 79.8. IR (ATR): ν = 3336, 3289, 1650, 1596, 855, 743, 689 cm^{-1}. HRMS m/z (ESI) calcd for $C_{15}H_{12}NO$ (M + H)$^+$: 222.0913; found: 222.0912.

2-chloro-N-phenylnicotinamide (**3jj**)

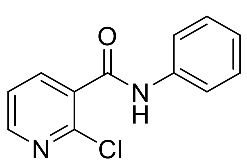

According to the general procedure in 0.2 mmol scale using 1.2 equiv. nitrobenzene with reaction time of 24 h; purified by flash chromatography (*n*-hexane/ethyl acetate = 3:1), 32.0 mg, 69%, white solid, m. p. = 125 - 126 °C; R_f= 0.3 (*n*-hexane/ethyl acetate = 3:1). ^{1}H NMR (400 MHz, Chloroform-*d*) δ 8.48 (dd, *J* = 4.8, 2.0 Hz, 1H), 8.28 (s, 1H), 8.14 (dd, *J* = 7.7, 2.0 Hz, 1H), 7.64 (d, *J* = 8.0 Hz, 2H), 7.44 – 7.37 (m, 2H), 7.36 (d, *J* = 5.4 Hz, 1H), 7.20 (t, *J* = 7.4 Hz, 1H). ^{13}C NMR (101 MHz, Chloroform-*d*) δ 162.8, 151.1, 147.0, 139.7, 137.1, 131.6, 129.2, 125.3, 122.8, 120.3. IR (ATR): ν = 3258, 1657, 1601, 754, 691 cm^{-1}. HRMS m/z (ESI) calcd for $C_{12}H_{10}ClN_2O$ (M + H)$^+$: 233.0476; found: 223.0474.

1-methyl-N-phenyl-3-(trifluoromethyl)-1H-pyrazole-4-carboxamide (**3kk**)

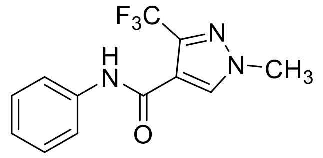

According to the general procedure in 0.2 mmol scale using 1.2 equiv. nitrobenzene with reaction time of 24 h; purified by flash chromatography (*n*-hexane/ethyl acetate = 3:1), 33.9 mg, 63%, white solid, m. p. = 135 - 136 ºC; R_f = 0.2 (*n*-hexane/ethyl acetate = 3:1). ^{1}H NMR (400 MHz, Chloroform-*d*) δ 7.98 (s, 1H), 7.84 (s, 1H), 7.56 (d, *J* = 7.9 Hz, 2H), 7.35 (t, *J* = 7.8 Hz, 2H), 7.15 (t, *J* = 7.4 Hz, 1H), 3.94 (s, 3H). ^{13}C NMR (101 MHz, Chloroform-*d*) δ 158.6, 138.4 (q, J = 37.4 Hz), 137.4, 135.5, 129.1, 124.9, 121.0 (q, J = 270.7 Hz), 120.3, 117.6, 39.8. IR (ATR): ν = 3387, 2924, 2853, 1632, 1598, 734, 695 cm^{-1}. HRMS m/z (ESI) calcd for $C_{12}H_{11}F_3N_3O$ $(M + H)^+$: 270.0849; found: 270.0841.

N-phenylquinoline-2-carboxamide (**3ll**)

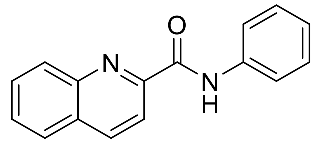

According to the general procedure in 0.2 mmol scale using 1.2 equiv. nitrobenzene with reaction time of 24 h; purified by flash chromatography (*n*-hexane/ethyl acetate = 3:1), 27.5 mg, 55%, white solid, m. p. = 161 - 162 ºC; R_f = 0.7 (*n*-hexane/ethyl acetate = 3:1). ^{1}H NMR (400 MHz, Chloroform-*d*) δ 10.24 (s, 1H), 8.41 (d, *J* = 8.5 Hz, 1H), 8.37 (d, *J* = 8.5 Hz, 1H), 8.20 (d, *J* = 8.1 Hz, 1H), 7.92 (d, *J* = 9.1 Hz, 1H), 7.86 (dd, *J* = 8.6, 1.0 Hz, 2H), 7.84 – 7.78 (m, 1H), 7.69 – 7.63 (m, 1H), 7.47 – 7.39 (m, 2H), 7.18 (t, *J* = 7.4 Hz, 1H). ^{13}C NMR (101 MHz, Chloroform-*d*) δ 162.2, 149.7, 146.3, 146.1, 137.9, 130.3, 129.7, 129.4, 129.1, 128.2, 127.8, 124.4, 119.8, 118.8. IR (ATR): ν = 3342, 1680, 1597, 754, 735, 692 cm^{-1}. HRMS m/z (ESI) calcd for $C_{16}H_{13}N_2O$ $(M + H)^+$: 249.1022; found: 249.1017.

N-phenylbenzofuran-5-carboxamide (**3mm**)

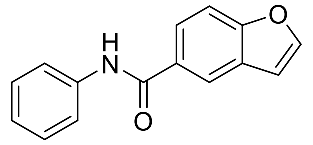

According to the general procedure in 0.2 mmol scale using 1.2 equiv. nitrobenzene with reaction time of 24 h; purified by flash chromatography (*n*-hexane/ethyl acetate = 3:1), 37.0 mg, 78%, white solid, m. p. = 158 - 159 ºC; R_f = 0.5 (*n*-hexane/ethyl acetate = 3:1). ^{1}H NMR (400 MHz, DMSO-d_6) δ 10.29 (s, 1H), 8.32 (d, *J* = 1.7 Hz, 1H), 8.12 (d, *J* = 2.2 Hz, 1H), 7.94 (dd, *J* = 8.7, 1.9 Hz, 1H), 7.81 (d, *J* = 8.3 Hz, 2H), 7.74 (d, *J* = 8.6 Hz, 1H), 7.36 (t, *J* = 7.8 Hz, 2H), 7.15 – 7.06 (m, 2H). ^{13}C NMR (101 MHz, DMSO-d_6) δ 165.6, 155.9, 147.3, 139.3, 130.1, 128.6, 127.0, 124.3, 123.5, 121.3, 120.3, 111.1, 107.2. IR (ATR): ν = 3367, 1656, 1597, 865, 831 cm^{-1}. HRMS m/z (ESI) calcd for $C_{15}H_{12}NO_2$ $(M + H)^+$: 238.0863; found: 238.0856.

1-methyl-N-phenyl-1H-pyrrole-2-carboxamide (**3nn**)

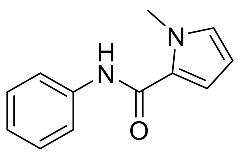

According to the general procedure in 0.2 mmol scale using 1.2 equiv. nitrobenzene with reaction time of 24 h; purified by flash chromatography (*n*-hexane/ethyl acetate = 3:1), 23.6 mg, 59%, white solid, m. p. = 116 - 117 °C; R_f = 0.6 (*n*-hexane/ethyl acetate = 3:1). ^{1}H NMR (400 MHz, Chloroform-*d*) δ 7.58 (s, 1H), 7.55 (d, *J* = 8.5 Hz, 3H), 7.35 (t, *J* = 7.7 Hz, 2H), 7.11 (t, *J* = 7.9 Hz, 1H), 6.79 (s, 1H), 6.70 (d, *J* = 3.8 Hz, 1H), 6.15 (t, *J* = 3.5 Hz, 1H), 3.98 (s, 3H). ^{13}C NMR (101 MHz, Chloroform-*d*) δ 165.2, 138.0, 129.1, 128.8, 124.0, 121.9, 120.0, 112.1, 107.5, 36.9. IR (ATR): ν = 3311, 2928, 2852, 1644, 1596, 752, 692 cm^{-1}. HRMS m/z (ESI) calcd for $C_{12}H_{13}N_2O$ $(M + H)^+$: 201.1022; found: 201.1021.

N-phenyl-4-(phenylethynyl)benzamide (**3oo**)

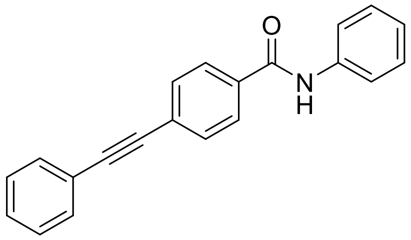

According to the general procedure in 0.2 mmol scale using 1.2 equiv. nitrobenzene with reaction time of 24 h; purified by flash chromatography (*n*-hexane/ethyl acetate = 5:1), 35.0 mg, 59%, white solid, m. p. = 165 - 166 °C; R_f = 0.6 (*n*-hexane/ethyl acetate = 5:1). ^{1}H NMR (400 MHz, DMSO-d_6) δ 10.35 (s, 1H), 8.03 (d, *J* = 8.2 Hz, 2H), 7.80 (d, *J* = 8.1 Hz, 2H), 7.73 (d, *J* = 8.2 Hz, 2H), 7.61 (dd, *J* = 6.5, 3.0 Hz, 2H), 7.50 – 7.44 (m, 3H), 7.37 (t, *J* = 7.8 Hz, 2H), 7.12 (t, *J* = 7.4 Hz, 1H). ^{13}C NMR (101 MHz, DMSO-d_6) δ 164.6, 139.0, 134.6, 131.5, 131.3, 129.1, 128.8, 128.6, 128.0, 125.3, 123.8, 121.8, 120.4, 91.4, 88.7. IR (ATR): ν = 3340, 2121, 1646, 1597, 746, 686 cm^{-1}. HRMS m/z (ESI) calcd for $C_{21}H_{16}NO$ $(M + H)^+$: 298.1226; found: 298.1217.

2,4,6-trimethyl-N-phenylbenzamide (**3pp**)[4]

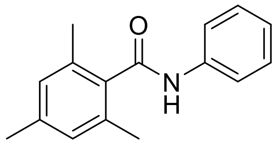

According to the general procedure in 0.2 mmol scale using 1.2 equiv. nitrobenzene with reaction time of 24 h; purified by flash chromatography (*n*-hexane/ethyl acetate = 5:1),37.8 mg, 79%, white solid, m. p. = 166 - 167 °C; R_f = 0.5 (*n*-hexane/ethyl acetate = 5:1). ^{1}H NMR (400 MHz, Chloroform-*d*) δ 7.59 (d, *J* = 8.4 Hz, 2H), 7.50 (s, 1H), 7.34 (t, *J* = 7.7 Hz, 2H), 7.14 (t, *J* = 7.4 Hz, 1H), 6.86 (s, 2H), 2.32 (s, 6H), 2.29 (s, 3H). ^{13}C NMR (101 MHz, Chloroform-*d*) δ 168.8, 138.8, 137.9, 134.9, 134.2, 129.1, 128.3, 124.5, 119.8, 21.1, 19.1. IR (ATR): ν = 3279, 2952, 2918, 2857, 1656, 1594, 752, 689 cm^{-1}. HRMS m/z (ESI) calcd for $C_{16}H_{18}NO$ $(M + H)^+$: 240.1383; found: 240.1378.

4-methoxy-N-phenylbenzamide (**3qq**)[4]

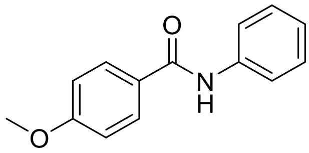

According to the general procedure in 0.2 mmol scale using 1.2 equiv. nitrobenzene with reaction time of 24 h; purified by flash chromatography (*n*-hexane/ethyl acetate = 5:1), 32.2 mg, 71%, white solid, m. p. = 169 - 170 °C; R_f = 0.5 (*n*-hexane/ethyl acetate = 5:1). ^{1}H NMR (400 MHz, Chloroform-*d*) δ 7.85 (d, *J* = 8.8 Hz, 2H), 7.75 (s, 1H), 7.63 (d, *J* = 7.6 Hz, 2H), 7.41 – 7.33 (m, 2H), 7.14 (t, *J* = 7.4 Hz, 1H), 6.98 (d, *J* = 8.8 Hz, 2H), 3.88 (s, 3H). ^{13}C NMR (101 MHz, Chloroform-*d*) δ 162.5, 138.1, 129.4, 129.1, 128.9, 124.4, 120.1, 119.8, 114.0, 55.5. IR (ATR): ν = 3337, 2959, 2933, 2839, 1653, 1595, 846 cm^{-1}. HRMS m/z (ESI) calcd for $C_{14}H_{14}NO_2$ $(M + H)^+$: 228.1019; found: 228.1015.

methyl 4-(phenylcarbamoyl)benzoate (**3rr**)

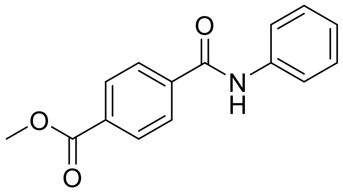

According to the general procedure in 0.2 mmol scale using 1.2 equiv. nitrobenzene with reaction time of 24 h; purified by flash chromatography (*n*-hexane/ethyl acetate = 5:1), 34.2 mg, 67%, white solid, m. p. = 192 - 193 °C; R_f = 0.5 (*n*-hexane/ethyl acetate = 5:1). ^{1}H NMR (400 MHz, Chloroform-*d*) δ 8.15 (d, *J* = 8.3 Hz, 2H), 7.93 (d, *J* = 8.2 Hz, 2H), 7.86 (s, 1H), 7.65 (d, *J* = 8.0 Hz, 2H), 7.39 (t, *J* = 7.9 Hz, 2H), 7.18 (t, *J* = 7.0 Hz, 1H), 3.96 (s, 3H). ^{13}C NMR (101 MHz, Chloroform-*d*) δ 166.2, 164.8, 138.9, 137.6, 133.1, 130.1, 129.2, 127.1, 124.9, 120.3, 52.5. IR (ATR): ν = 3368, 2952, 2923, 2851, 1658, 1599, 815, 754, 689 cm^{-1}. HRMS m/z (ESI) calcd for $C_{15}H_{14}NO_3$ $(M + H)^+$: 256.0968; found: 256.0961.

N-(4-methoxyphenyl)heptanamide (**3ss**)[6]

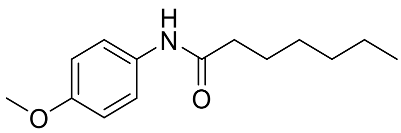

According to the general procedure in 0.2 mmol scale using 1.2 equiv. 1-methoxy-4-nitrobenzene with reaction time of 24 h; purified by flash chromatography (*n*-hexane / ethyl acetate = 5:1), 45.6 mg, 97% yield, white solid, m.p. = 103 – 105 °C; R_f = 0.4 (*n*-hexane / ethyl acetate = 5:1). ^{1}H NMR (400 MHz, Chloroform-*d*) δ 7.75 (s, 1H), 7.40 (d, *J* = 9.0 Hz, 2H), 6.81 (d, *J* = 9.0 Hz, 2H), 3.76 (s, 3H), 2.30 (t, *J* = 7.8 Hz, 2H), 1.68 (quint, *J* = 7.5 Hz, 2H), 1.37 – 1.24 (m, 6H), 0.87 (t, *J* = 7.6 Hz, 3H). ^{13}C NMR (101 MHz, Chloroform-*d*) δ 171.7, 156.2, 131.2, 121.9, 113.9, 55.4, 37.5, 31.5, 28.9, 25.7, 22.5, 14.0. IR (ATR): ν = 3317, 2955, 2920, 2871, 2849, 1650, 1243, 823 cm^{-1}. HRMS m/z (ESI) calcd for $C_{14}H_{22}NO_2$ $(M + H)^+$: 236.1645; found: 236.1639.

N-(4-chlorophenyl)heptanamide (**3tt**)[6]

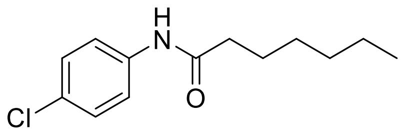

According to the general procedure in 0.2 mmol scale using 1.2 equiv. 1-chloro-4-nitrobenzene with reaction time of 24 h; purified by flash chromatography (*n*-hexane / ethyl acetate = 5:1), 45.0 mg, 94% yield, white solid, m.p. = 82 – 85 °C; R_f = 0.5 (*n*-hexane / ethyl acetate = 5:1). ^{1}H NMR (400 MHz, Chloroform-*d*) δ 7.50 (s, 1H), 7.47 (d, *J* = 8.8 Hz, 2H), 7.25 (d, *J* = 8.8 Hz, 2H), 2.33 (t, *J* = 8.0 Hz, 2H), 1.70 (quint, *J* = 7.5 Hz, 2H), 1.37 – 1.25 (m, 6H), 0.88 (t, *J* = 8.0 Hz, 3H). ^{13}C NMR (101 MHz, Chloroform-*d*) δ 171.6, 136.5, 129.1, 128.9, 121.1, 37.7, 31.5, 28.9, 25.5, 22.5, 14.0. IR (ATR): ν = 3305, 2958, 2931, 2871, 2852, 1656, 817, 696 cm^{-1}. HRMS m/z (ESI) calcd for $C_{13}H_{19}ClNO$ (M + H)$^+$: 240.1150; found: 240.1147.

N-(4-aminophenyl)heptanamide (**3uu**)

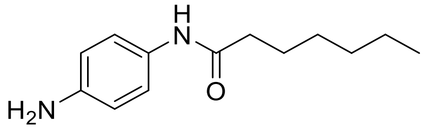

According to the general procedure in 0.2 mmol scale using 1.2 equiv. 4-nitroaniline with reaction time of 24 h; purified by flash chromatography (*n*-hexane / ethyl acetate = 3:1), 20.2 mg, 46% yield, white solid, m.p. = 75 – 77 °C; R_f = 0.2 (*n*-hexane / ethyl acetate = 3:1). ^{1}H NMR (400 MHz, Chloroform-*d*) δ 7.26 (d, *J* = 8.7 Hz, 2H), 7.07 (s, 1H), 6.64 (d, *J* = 8.7 Hz, 2H), 3.66 (s, 2H), 2.30 (t, *J* = 7.6 Hz, 2H), 1.70 (quint, *J* = 7.5 Hz, 2H), 1.39 – 1.27 (m, 6H), 0.89 (t, *J* = 6.8 Hz, 3H). ^{13}C NMR (101 MHz, Chloroform-*d*) δ 171.3, 143.2, 129.3, 122.0, 115.4, 37.6, 31.6, 29.0, 25.7, 22.5, 14.0. IR (ATR): ν = 3393, 3278, 2953, 2930, 2870, 2855, 1642, 824 cm^{-1}. HRMS m/z (ESI) calcd for $C_{13}H_{21}N_2O$ (M + H)$^+$: 221.1648; found: 221.1642.

N-(p-tolyl)heptanamide (**3vv**)[6]

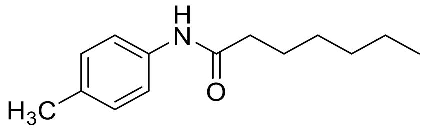

According to the general procedure in 0.2 mmol scale using 1.2 equiv. 1-methyl-4-nitrobenzene with reaction time of 24 h; purified by flash chromatography (*n*-hexane / ethyl acetate = 5:1), 41.0 mg, 94% yield, white solid, m.p. = 78 – 80 °C; R_f = 0.5 (*n*-hexane / ethyl acetate = 5:1). ^{1}H NMR (400 MHz, Chloroform-*d*) δ 7.60 (s, 1H), 7.39 (d, *J* = 8.4 Hz, 2H), 7.08 (d, *J* = 8.2 Hz, 2H), 2.30 (d, *J* = 7.8 Hz, 5H), 1.69 (quint, *J* = 7.5 Hz, 2H), 1.39 – 1.22 (m, 6H), 0.87 (t, *J* = 6.8 Hz, 3H). ^{13}C NMR (101 MHz, Chloroform-*d*) δ 171.6, 135.5, 133.6, 129.3, 120.0, 37.7, 31.5, 28.9, 25.7, 22.5, 20.8, 14.0. IR (ATR): ν = 3315, 2951, 2931, 2917, 2867, 2855, 1657, 814 cm^{-1}. HRMS m/z (ESI) calcd for $C_{13}H_{22}NO$ (M + H)$^+$: 220.1696; found: 220.1693.

N-(4-(difluoromethoxy)phenyl)heptanamide (**3ww**)

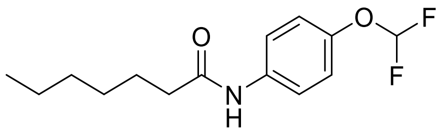

According to the general procedure in 0.2 mmol scale using 1.2 equiv. 1-(difluoromethoxy)-4-nitrobenzene with reaction time of 24 h; purified by flash chromatography (*n*-hexane / ethyl acetate = 5:1), 49.8 mg, 92% yield, white solid, m.p. = 71 – 72 °C; R_f = 0.6 (*n*-hexane / ethyl acetate = 5:1). ^{1}H NMR (400 MHz, Chloroform-*d*) δ 7.63 (s, 1H), 7.50 (d, *J* = 8.5 Hz, 2H), 7.05 (d, *J* = 8.4 Hz, 2H), 6.45 (t, *J* = 74.0 Hz, 1H), 2.34 (t, *J* = 7.6 Hz, 2H), 1.70 (quint, *J* = 7.5 Hz, 2H), 1.39 – 1.23 (m, 6H), 0.88 (t, *J* = 6.6 Hz, 3H). ^{13}C NMR (101 MHz, Chloroform-*d*) δ 171.8, 147.2, 135.5, 121.4, 120.3, 116.0 (t, *J* = 260.0 Hz), 37.6, 31.5, 28.9, 25.6, 22.5, 14.0. IR (ATR): ν = 3302, 2960, 2929, 2857, 1655, 1222, 1128, 839 cm^{-1}. HRMS m/z (ESI) calcd for $C_{14}H_{20}F_2NO_2$ (M + H)$^+$: 272.1457; found: 272.1451.

N-(4-(trifluoromethyl)phenyl)heptanamide (**3xx**)

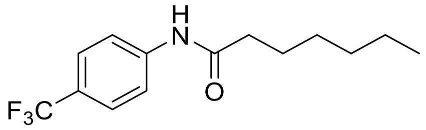

According to the general procedure in 0.2 mmol scale using 1.2 equiv. 1-nitro-4-(trifluoromethyl)benzene with reaction time of 24 h; purified by flash chromatography (*n*-hexane / ethyl acetate = 5:1), 35.0 mg, 64% yield, white solid, m.p. = 97 – 99 °C; R_f = 0.5 (*n*-hexane / ethyl acetate = 5:1). ^{1}H NMR (400 MHz, Chloroform-*d*) δ 7.65 (d, *J* = 8.5 Hz, 2H), 7.56 (d, *J* = 8.5 Hz, 2H), 7.38 (s, 1H), 2.38 (t, *J* = 7.6 Hz, 2H), 1.73 (quint, *J* = 7.5 Hz, 2H), 1.41 – 1.27 (m, 6H), 0.89 (t, *J* = 8.0 Hz, 3H). ^{13}C NMR (101 MHz, Chloroform-*d*) δ 171.7, 141.0, 126.3 (q, *J* = 3.8 Hz), 125.9 (q, *J* = 33.1 Hz), 124.1 (q, *J* = 271.6 Hz), 119.3, 37.8, 31.5, 28.9, 25.4, 22.5, 14.0. IR (ATR): ν = 3309, 2947, 2916, 2873, 1679, 1156, 856 cm^{-1}. HRMS m/z (ESI) calcd for $C_{14}H_{19}F_3NO$ (M + H)$^+$: 274.1413; found: 274.1405.

N-(4-(cyanomethyl)phenyl)heptanamide (**3yy**)

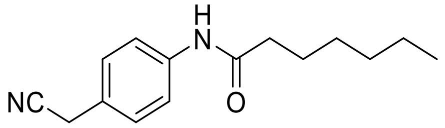

According to the general procedure in 0.2 mmol scale using 1.2 equiv. 2-(4-nitrophenyl)acetonitrile with reaction time of 24 h; purified by flash chromatography (*n*-hexane / ethyl acetate = 5:1), 42.7 mg, 87% yield, white solid, m.p. = 80 – 82 °C; R_f = 0.2 (*n*-hexane / ethyl acetate = 5:1). ^{1}H NMR (400 MHz, Chloroform-*d*) δ 7.64 (s, 1H), 7.54 (d, *J* = 8.4 Hz, 2H), 7.24 (d, *J* = 8.5 Hz, 2H), 3.71 (s, 2H), 2.35 (t, *J* = 7.6 Hz, 2H), 1.71 (quint, *J* = 7.5 Hz, 2H), 1.41 – 1.23 (m, 6H), 0.88 (t, *J* = 8.6 Hz, 3H). ^{13}C NMR (101 MHz, Chloroform-*d*) δ 171.8, 137.9, 128.5, 125.2, 120.3, 118.0, 37.7, 31.5, 28.9, 25.5, 23.0, 22.5, 14.0. IR (ATR): ν = 3288, 2953, 2918, 2869, 2847, 2114, 1656, 809 cm^{-1}. HRMS m/z (ESI) calcd for $C_{15}H_{21}N_2O$ (M + H)$^+$: 245.1648; found: 245.1644.

N-(4-(tert-butyl)phenyl)heptanamide (**3zz**)[4]

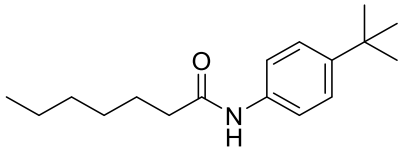

According to the general procedure in 0.2 mmol scale using 1.2 equiv. 1-(tert-butyl)-4-nitrobenzene with reaction time of 24 h; purified by flash chromatography (*n*-hexane / ethyl acetate = 5:1), 47.5 mg, 91% yield, white solid, m.p. = 77 – 79 °C; R_f = 0.5 (*n*-hexane / ethyl acetate = 5:1). ^{1}H NMR (400 MHz, Chloroform-*d*) δ 8.05 (s, 1H), 7.46 (d, *J* = 8.7 Hz, 2H), 7.28 (d, *J* = 8.7 Hz, 2H), 2.33 (t, *J* = 7.6 Hz, 2H), 1.68 (quint, *J* = 7.4 Hz, 2H), 1.36 – 1.25 (m, 15H), 0.87 (t, *J* = 6.7 Hz, 3H). ^{13}C NMR (101 MHz, Chloroform-*d*) δ 171.9, 146.9, 135.5, 125.5, 119.8, 37.6, 34.2, 31.5, 31.3, 28.9, 25.7, 22.4, 14.0. IR (ATR): ν = 3294, 2958, 2929, 2862, 1659, 835 cm^{-1}. HRMS m/z (ESI) calcd for $C_{17}H_{28}NO$ $(M + H)^+$: 262.2165; found: 262.2160.

N-(4-(4,4,5,5-tetramethyl-1,3,2-dioxaborolan-2-yl)phenyl)heptanamide (**3ab**)

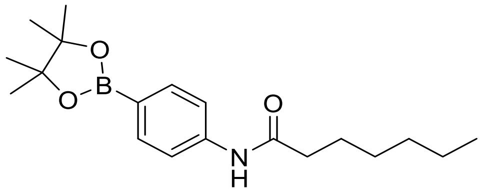

According to the general procedure in 0.2 mmol scale using 1.2 equiv. 4,4,5,5-tetramethyl-2-(4-nitrophenyl)-1,3,2-dioxaborolane with reaction time of 24 h; purified by flash chromatography (*n*-hexane / ethyl acetate = 3:1), 47.0 mg, 71% yield, white solid, m.p. = 102 – 105 °C; R_f = 0.4 (*n*-hexane / ethyl acetate = 3:1). ^{1}H NMR (400 MHz, Chloroform-*d*) δ 7.75 (d, *J* = 8.4 Hz, 2H), 7.54 (d, *J* = 8.0 Hz, 2H), 7.51 (s, 1H), 2.34 (t, *J* = 7.6 Hz, 2H), 1.70 (quint, *J* = 7.4 Hz, 2H), 1.43 – 1.26 (m, 18H), 0.88 (t, *J* = 6.6 Hz, 3H). ^{13}C NMR (101 MHz, Chloroform-*d*) δ 171.6, 140.7, 135.7, 118.5, 83.7, 37.8, 31.5, 28.9, 25.5, 24.8, 22.5, 14.0. IR (ATR): ν = 3296, 2961, 2927, 2860, 1659, 1593, 1467, 830 cm^{-1}. HRMS m/z (ESI) calcd for $C_{19}H_{31}BNO_3$ $(M + H)^+$: 332.2392; found: 332.2387.

N-(4-((4-bromophenyl)thio)phenyl)heptanamide (**3ac**)

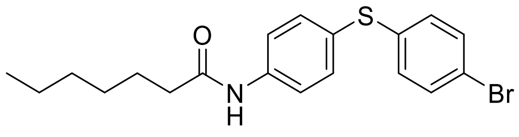

According to the general procedure in 0.2 mmol scale using 1.2 equiv. (4-bromophenyl)(4-nitrophenyl)sulfane with reaction time of 24 h; purified by flash chromatography (*n*-hexane / ethyl acetate = 5:1), 63.5 mg, 81% yield, white solid, m.p. = 113 – 115 °C; R_f = 0.5 (*n*-hexane / ethyl acetate = 5:1). ^{1}H NMR (400 MHz, Chloroform-*d*) δ 7.52 (d, *J* = 8.4 Hz, 2H), 7.42 (s, 1H), 7.39 – 7.31 (m, 4H), 7.08 (d, *J* = 8.5 Hz, 2H), 2.35 (t, *J* = 7.6 Hz, 2H), 1.71 (quint, *J* = 7.5 Hz, 2H), 1.40 – 1.25 (m, 6H), 0.88 (t, *J* = 6.7 Hz, 3H). ^{13}C NMR (101 MHz, Chloroform-*d*) δ 171.6, 138.0, 136.6, 133.6, 132.1, 130.8, 128.6, 120.6, 120.2, 37.8, 31.5, 28.9, 25.5, 22.5, 14.0. IR (ATR): ν = 3295, 2954, 2923, 2854, 1658, 1592, 810 cm^{-1}. HRMS m/z (ESI) calcd for $C_{19}H_{23}B_rNOS$ $(M + H)^+$: 392.0678; found: 392.0670.

N-(3-bromophenyl)heptanamide (**3ad**)

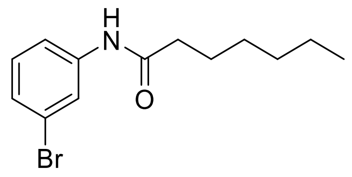

According to the general procedure in 0.2 mmol scale using 1.2 equiv. 1-bromo-3-nitrobenzene with reaction time of 24 h; purified by flash chromatography (*n*-hexane / ethyl acetate = 5:1), 48.3 mg, 85% yield, white solid, m.p. = 87 – 89 °C; R_f = 0.6 (*n*-hexane / ethyl acetate = 5:1). ^{1}H NMR (400 MHz, Chloroform-*d*) δ 7.78 (s, 1H), 7.52 (s, 1H), 7.42 (d, *J* = 7.9 Hz, 1H), 7.21 (d, *J* = 8.3 Hz, 1H), 7.15 (t, *J* = 8.0 Hz, 1H), 2.34 (t, *J* = 7.6 Hz, 2H), 1.70 (quint, *J* = 7.3 Hz, 2H), 1.38 – 1.26 (m, 6H), 0.88 (t, *J* = 8.0 Hz, 3H). ^{13}C NMR (101 MHz, Chloroform-*d*) δ 171.7, 139.2, 130.2, 127.1, 122.8, 118.3, 37.7, 31.5, 28.9, 25.5, 22.5, 14.0. IR (ATR): ν = 3297, 2955, 2927, 2857, 1662, 872, 775, 726 cm^{-1}. HRMS m/z (ESI) calcd for $C_{13}H_{19}BrNO$ (M + H)$^+$: 284.0645; found: 284.0640.

N-(3-acetylphenyl)heptanamide (**3ae**)

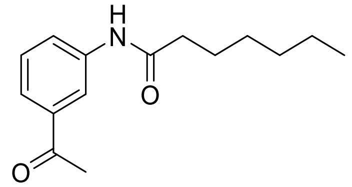

According to the general procedure in 0.2 mmol scale using 1.2 equiv. 1-(3-nitrophenyl)ethan-1-one with reaction time of 24 h; purified by flash chromatography (*n*-hexane / ethyl acetate = 5:1), 31.0 mg, 63% yield, white solid, m.p. = 72 – 74 °C; R_f = 0.3 (*n*-hexane / ethyl acetate = 5:1). ^{1}H NMR (400 MHz, Chloroform-*d*) δ 8.20 (s, 1H), 8.09 (s, 1H), 7.96 (d, *J* = 8.0 Hz, 1H), 7.66 (d, *J* = 7.8 Hz, 1H), 7.40 (t, *J* = 7.9 Hz, 1H), 2.59 (s, 3H), 2.40 (t, *J* = 7.6 Hz, 2H), 1.73 (quint, *J* = 7.5 Hz, 2H), 1.39 – 1.26 (m, 6H), 0.87 (t, *J* = 6.8 Hz, 3H). ^{13}C NMR (101 MHz, Chloroform-*d*) δ 198.3, 172.2, 138.8, 137.5, 129.2, 124.5, 123.9, 119.2, 37.6, 31.5, 28.9, 26.7, 25.5, 22.5, 14.0. IR (ATR): ν = 3283, 2953, 2930, 2867, 2850, 1671, 1656, 887, 799, 698 cm^{-1}. HRMS m/z (ESI) calcd for $C_{15}H_{22}NO_2$ (M + H)$^+$: 248.1645; found: 248.1641.

N-(3-formylphenyl)heptanamide (**3af**)

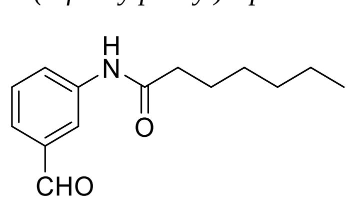

According to the general procedure in 0.2 mmol scale using 1.2 equiv. 3-nitrobenzaldehyde with reaction time of 24 h; purified by flash chromatography (*n*-hexane / ethyl acetate = 5:1), 31.0 mg, 67% yield, white solid, m.p. = 80 – 81 °C; R_f = 0.4 (*n*-hexane / ethyl acetate = 5:1). ^{1}H NMR (400 MHz, Chloroform-*d*) δ 9.96 (s, 1H), 8.02 (s, 1H), 7.89 (d, *J* = 8.0 Hz, 1H), 7.79 (s, 1H), 7.60 (d, *J* = 7.5 Hz, 1H), 7.46 (t, *J* = 7.8 Hz, 1H), 2.39 (t, *J* = 7.6 Hz, 2H), 1.72 (quint, *J* = 7.5 Hz, 2H), 1.39 – 1.25 (m, 6H), 0.87 (t, *J* = 7.8 Hz, 3H). ^{13}C NMR (101 MHz, Chloroform-*d*) δ 192.1, 139.0, 137.0, 129.7, 125.6, 125.4, 120.4, 37.7, 31.5, 28.9, 25.5, 22.5, 14.0. IR (ATR): ν = 3305, 2955, 2927, 2856, 2726, 1665, 891, 789, 727 cm^{-1}. HRMS m/z (ESI) calcd for $C_{14}H_{20}NO_2$ (M + H)$^+$: 234.1489; found:

234.1485.

N-(3-propionamidophenyl)heptanamide (**3ag**)

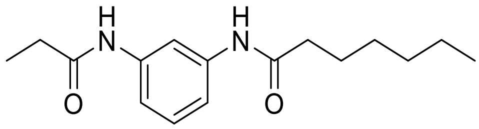

According to the general procedure in 0.2 mmol scale using 1.2 equiv. N-(3-nitrophenyl)propionamide with reaction time of 24 h; purified by flash chromatography (*n*-hexane / ethyl acetate = 5:1), 44.0 mg, 80% yield, white solid, m.p. = 141 – 143 °C; R_f = 0.4 (*n*-hexane / ethyl acetate = 5:1). ^{1}H NMR (400 MHz, Methanol-d_4) δ 7.75 (s, 1H), 7.22 – 7.16 (m, 2H), 7.15 – 7.07 (m, 1H), 4.77 (s, 2H), 2.33 – 2.19 (m, 4H), 1.58 (quint, *J* = 7.5 Hz, 2H), 1.31 – 1.21 (m, 6H), 1.09 (t, *J* = 7.6 Hz, 3H), 0.81 (t, *J* = 6.6 Hz, 3H). ^{13}C NMR (101 MHz, Methanol-d_4) δ 175.4, 174.8, 140.3, 130.0, 116.9, 113.2, 38.0, 32.8, 31.0, 30.1, 26.9, 23.6, 14.4, 10.3. IR (ATR): ν = 3292, 3209, 2959, 2925, 2854, 1653, 1607, 876, 778, 721 cm^{-1}. HRMS m/z (ESI) calcd for $C_{16}H_{25}N_2O_2$ (M + H)$^+$: 277.1911; found: 277.1904.

N-(4-bromo-3-ethoxyphenyl)heptanamide (**3ah**)

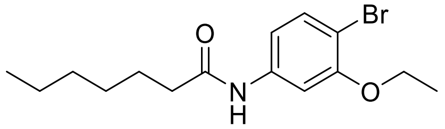

According to the general procedure in 0.2 mmol scale using 1.2 equiv. 1-bromo-2-ethoxy-4-nitrobenzene with reaction time of 24 h; purified by flash chromatography (*n*-hexane / ethyl acetate = 5:1), 52.1 mg, 79% yield, white solid, m.p. = 98 – 99 °C; R_f = 0.4 (*n*-hexane / ethyl acetate = 5:1). ^{1}H NMR (400 MHz, Chloroform-*d*) δ 7.53 (d, *J* = 2.4 Hz, 1H), 7.44 (s, 1H), 7.40 (d, *J* = 8.5 Hz, 1H), 6.73 (dd, *J* = 8.5, 2.4 Hz, 1H), 4.07 (q, *J* = 7.0 Hz, 2H), 2.33 (t, *J* = 7.6 Hz, 2H), 1.70 (quint, *J* = 7.5 Hz, 2H), 1.44 (t, *J* = 7.0 Hz, 3H), 1.40 – 1.22 (m, 6H), 0.88 (t, *J* = 6.7 Hz, 3H). ^{13}C NMR (101 MHz, Chloroform-*d*) δ 171.7, 155.6, 138.5, 132.9, 112.3, 106.3, 105.3, 64.8, 37.8, 31.5, 28.9, 25.4, 22.5, 14.6, 14.0. IR (ATR): ν = 3304, 2971, 2950, 2857, 1658, 853, 806 cm^{-1}. HRMS m/z (ESI) calcd for $C_{15}H_{23}BrNO_2$ (M + H)$^+$: 328.0907; found: 328.0903.

N-(3-sulfamoylphenyl)heptanamide (**3ai**)

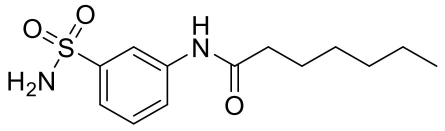

According to the general procedure in 0.2 mmol scale using 1.2 equiv. 3-nitrobenzenesulfonamide with reaction time of 24 h; purified by flash chromatography (*n*-hexane / ethyl acetate = 1:1), 40.2 mg, 71% yield, white solid, m.p. = 126 – 128 °C; R_f = 0.3 (*n*-hexane / ethyl acetate = 1:1). ^{1}H NMR (400 MHz, Acetonitrile-d_3) δ 8.56 (s, 1H), 8.25 (s, 1H), 7.66 (d, *J* = 8.0 Hz, 1H), 7.54 (d, *J* = 9.0 Hz, 1H), 7.46 (t, *J* = 7.9 Hz, 1H), 5.75 (s, 2H), 2.33 (t, *J* = 7.5 Hz, 2H), 1.64 (quint, *J* = 7.5 Hz, 2H), 1.33 (d, *J* = 12.2 Hz, 6H), 0.89 (t, *J* = 6.6 Hz, 3H). ^{13}C NMR (101 MHz, Acetonitrile-d_3) δ 173.2, 144.7, 140.6, 130.5, 123.5, 121.5, 117.4, 37.7, 32.2, 29.5, 26.0, 23.2, 14.3. IR (ATR): ν = 3325,

3296, 3189, 2957, 2929, 2860, 1674, 895, 798, 746 cm^{-1}. HRMS m/z (ESI) calcd for $C_{13}H_{21}N_2O_3S$ $(M + H)^+$: 285.1267; found: 285.1261.

N-(3-ethynylphenyl)heptanamide (**3aj**)

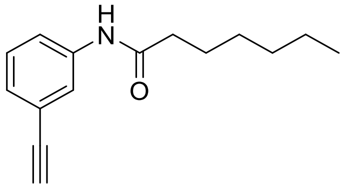

According to the general procedure in 0.2 mmol scale using 1.2 equiv. 1-ethynyl-3-nitrobenzene with reaction time of 24 h; 38.9 mg, purified by flash chromatography (*n*-hexane / ethyl acetate = 5:1), 85% yield, white solid, m.p. = 79 – 81 °C; R_f = 0.7 (*n*-hexane / ethyl acetate = 5:1). ^{1}H NMR (400 MHz, Chloroform-*d*) δ 7.63 (s, 1H), 7.56 (d, *J* = 7.9 Hz, 1H), 7.36 (s, 1H), 7.26 (d, *J* = 7.9 Hz, 1H), 7.24 – 7.18 (m, 1H), 3.05 (s, 1H), 2.34 (t, *J* = 7.6 Hz, 2H), 1.71 (quint, *J* = 7.3 Hz, 2H), 1.38 – 1.24 (m, 6H), 0.88 (t, *J* = 6.0 Hz, 3H). ^{13}C NMR (101 MHz, Chloroform-*d*) δ 171.6, 138.0, 129.0, 127.9, 123.2, 122.8, 120.4, 83.2, 77.4, 37.8, 31.5, 28.9, 25.5, 22.5, 14.0. IR (ATR): ν = 3275, 2955, 2927, 2857, 1660, 886, 780, 726 cm^{-1}. HRMS m/z (ESI) calcd for $C_{15}H_{20}NO$ $(M + H)^+$: 230.1539; found: 230.1539.

N-(3-(benzyloxy)-2-methylphenyl)heptanamide (**3ak**)

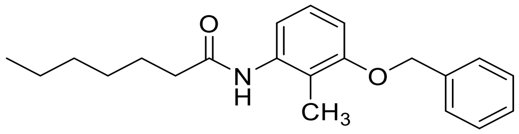

According to the general procedure in 0.2 mmol scale using 1.2 equiv. 1-(benzyloxy)-2-methyl-3-nitrobenzene with reaction time of 24 h; purified by flash chromatography (*n*-hexane / ethyl acetate = 5:1), 46.1 mg, 71% yield, white solid, m.p. = 88 – 89 °C; R_f = 0.3 (*n*-hexane / ethyl acetate = 5:1). ^{1}H NMR (400 MHz, Chloroform-*d*) δ 7.45 – 7.28 (m, 5H), 7.11 (t, *J* = 8.2 Hz, 2H), 6.74 (d, *J* = 8.2 Hz, 1H), 5.04 (s, 2H), 2.36 (t, *J* = 7.6 Hz, 2H), 2.16 (s, 3H), 1.72 (t, *J* = 7.5 Hz, 2H), 1.34 (m, 6H), 0.89 (t, *J* = 6.0 Hz, 3H). ^{13}C NMR (101 MHz, Chloroform-*d*) δ 171.6, 156.9, 137.2, 136.5, 128.5, 127.8, 127.1, 126.4, 119.0, 116.6, 108.8, 70.3, 37.5, 31.5, 28.9, 25.8, 22.5, 14.0, 10.2. IR (ATR): ν = 3275, 2954, 2933, 2855, 1717, 1645, 757, 725 cm^{-1}. HRMS m/z (ESI) calcd for $C_{21}H_{28}NO_2$ $(M + H)^+$: 326.2115; found: 326.2105.

N-(2-hydroxyphenyl)heptanamide (**3al**)

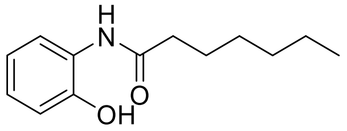

According to the general procedure in 0.2 mmol scale using 1.2 equiv. 2-nitrophenol with reaction time of 24 h; purified by flash chromatography (*n*-hexane / ethyl acetate = 5:1), 26.2 mg, 59% yield, white solid, m.p. = 80 – 83 °C; R_f = 0.4 (*n*-hexane / ethyl acetate = 5:1). ^{1}H NMR (400 MHz, Chloroform-*d*) δ 8.89 (s, 1H), 7.66 (s, 1H), 7.11 (t, *J* = 7.7 Hz, 1H), 7.02 (dd, *J* = 11.8, 8.0 Hz, 2H), 6.85 (t, *J* = 7.6 Hz, 1H), 2.44 (t, *J* = 7.6 Hz, 2H), 1.73 (quint, *J* = 7.5 Hz, 2H), 1.41 – 1.24 (m, 6H),

0.89 (t, J = 7.6 Hz, 3H). ^{13}C NMR (101 MHz, Chloroform-d) δ 173.7, 148.6, 127.0, 125.6, 122.1, 120.4, 119.7, 37.0, 31.5, 28.8, 25.7, 22.5, 14.0. IR (ATR): ν = 3629, 3285, 2956, 2929, 2858, 1654, 750 cm^{-1}. HRMS m/z (ESI) calcd for $C_{13}H_{20}NO_2$ (M + H)$^+$: 222.1489; found: 222.1486.

N-(2-methylbenzo[d]oxazol-6-yl)heptanamide (**3am**)

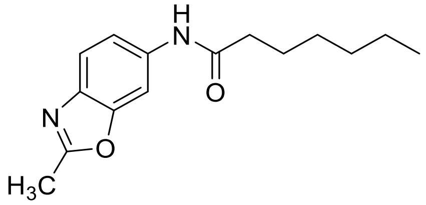

According to the general procedure in 0.2 mmol scale using 1.2 equiv. 2-methyl-6-nitrobenzo[d]oxazole with reaction time of 24 h; purified by flash chromatography (*n*-hexane / ethyl acetate = 1:1), 45.7 mg, 88% yield, white solid, m.p. = 95 – 97 °C; R_f = 0.5 (*n*-hexane / ethyl acetate = 1:1). ^{1}H NMR (400 MHz, Chloroform-d) δ 8.13 (d, J = 1.7 Hz, 1H), 7.60 (s, 1H), 7.51 (d, J = 8.5 Hz, 1H), 7.09 (dd, J = 8.5, 2.0 Hz, 1H), 2.61 (s, 3H), 2.38 (t, J = 7.6 Hz, 2H), 1.74 (quint, J = 7.5 Hz, 2H), 1.43 – 1.26 (m, 6H), 0.88 (t, J = 6.6 Hz,, 3H). ^{13}C NMR (101 MHz, Chloroform-d) δ 171.6, 164.0, 151.2, 137.8, 135.0, 119.0, 116.3, 102.8, 37.8, 31.5, 28.9, 25.6, 22.5, 14.5, 14.0. IR (ATR): ν = 3311, 2959, 2853, 1660, 854, 824, 807 cm^{-1}. HRMS m/z (ESI) calcd for $C_{15}H_{21}N_2O_2$ (M + H)$^+$: 261.1598; found: 261.1584.

N-(3-oxo-1,3-dihydroisobenzofuran-5-yl)heptanamide (**3an**)

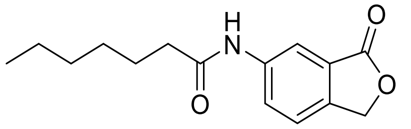

According to the general procedure in 0.2 mmol scale using 1.2 equiv. 6-nitroisobenzofuran-1(3H)-one with reaction time of 24 h; purified by flash chromatography (*n*-hexane / ethyl acetate = 1:1), 46.1 mg, 88% yield, white solid, m.p. = 135 – 137 °C; R_f = 0.6 (*n*-hexane / ethyl acetate = 1:1). ^{1}H NMR (400 MHz, Chloroform-d) δ 8.31 (s, 1H), 8.20 (d, J = 8.3 Hz, 1H), 7.96 (s, 1H), 7.44 (d, J = 8.3 Hz, 1H), 5.30 (s, 2H), 2.44 (t, J = 7.6 Hz, 2H), 1.74 (quint, J = 7.5 Hz, 2H), 1.43 – 1.25 (m, 6H), 0.88 (t, J = 6.7 Hz, 3H). ^{13}C NMR (101 MHz, Chloroform-d) δ 172.3, 171.3, 141.5, 139.6, 126.3, 126.1, 122.6, 115.9, 69.8, 37.6, 31.5, 28.9, 25.5, 22.5, 14.0. IR (ATR): ν = 3336, 2955, 2923, 2852, 1739, 1685, 886, 847 cm^{-1}. HRMS m/z (ESI) calcd for $C_{15}H_{20}NO_3$ (M + H)$^+$: 262.1438; found: 262.1432.

N-(1H-indol-5-yl)heptanamide (**3ao**)

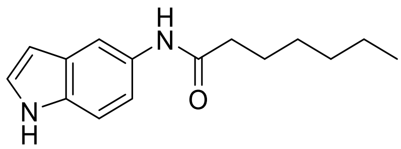

According to the general procedure in 0.2 mmol scale using 1.5 equiv. 5-nitro-1H-indole with reaction time of 60 h; purified by flash chromatography (*n*-hexane / ethyl acetate = 3:1), 35.4 mg, 72% yield, white solid, m.p. = 128 – 129 °C; R_f= 0.3 (*n*-hexane / ethyl acetate = 3:1). ^{1}H NMR (400

MHz, Chloroform-*d*) δ 8.27 (s, 1H), 7.82 (s, 1H), 7.28 (d, *J* = 8.6 Hz, 1H), 7.25 (d, *J* = 6.6 Hz, 1H), 7.19 (d, *J* = 9.3 Hz, 2H), 6.49 (s, 1H), 2.36 (t, *J* = 7.6 Hz, 2H), 1.75 (quint, *J* = 7.5 Hz, 2H), 1.45 – 1.27 (m, 6H), 0.89 (t, *J* = 6.7 Hz, 3H). ^{13}C NMR (101 MHz, Chloroform-*d*) δ 171.5, 133.2, 130.4, 128.0, 125.1, 116.4, 112.7, 111.1, 102.7, 37.8, 31.6, 29.0, 25.8, 22.5, 14.1. IR (ATR): ν = 3245, 2956, 2926, 2855, 1632, 878, 796 cm^{-1}. HRMS m/z (ESI) calcd for $C_{15}H_{21}N_2O$ (M + H)$^+$: 245.1648 ; found: 245.1647.

N-(4-iodophenyl)heptanamide (**3ap**)

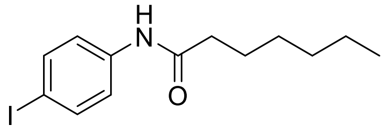

According to the general procedure in 0.2 mmol scale using 1.2 equiv. 1-iodo-4-nitrobenzene with reaction time of 24 h; purified by flash chromatography (*n*-hexane / ethyl acetate = 5:1), 47.0 mg, 71% yield, white solid, m.p. = 120 – 121 °C; R_f = 0.5 (*n*-hexane / ethyl acetate = 5:1). ^{1}H NMR (400 MHz, Chloroform-*d*) δ 7.59 (d, *J* = 8.7 Hz, 2H), 7.51 (s, 1H), 7.30 (d, *J* = 8.7 Hz, 2H), 2.33 (t, *J* = 7.6 Hz, 2H), 1.69 (quint, *J* = 7.5 Hz, 2H), 1.41 – 1.23 (m, 6H), 0.88 (t, *J* = 6.7 Hz, 3H). ^{13}C NMR (101 MHz, Chloroform-*d*) δ 171.7, 137.8, 137.7, 121.7, 87.3, 37.8, 31.5, 28.9, 25.5, 22.5, 14.0. IR (ATR): ν = 3281, 2984, 2930, 1734, 1237, 826, 729 cm^{-1}. HRMS m/z (ESI) calcd for $C_{13}H_{19}INO$ (M + H)$^+$: 332.0506; found: 332.0501.

N-methylheptanamide (**3aq**)

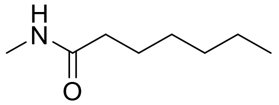

According to the general procedure in 0.2 mmol scale using 1.2 equiv. nitromethane with reaction time of 36 h; purified by flash chromatography (*n*-hexane/ethyl acetate = 5:1), 18.0 mg, 63%, white solid, m. p. = 94 - 95 °C; R_f = 0.2 (*n*-hexane/ethyl acetate = 5:1). ^{1}H NMR (400 MHz, Chloroform-*d*) δ 5.47 (s, 1H), 2.81 (d, *J* = 4.9 Hz, 3H), 2.16 (t, *J* = 7.9 Hz, 2H), 1.70 – 1.55(m, 2H), 1.34 – 1.26 (m, 6H), 0.88 (t, *J* = 7.9 Hz, 3H). ^{13}C NMR (101 MHz, Chloroform-*d*) δ 173.8, 36.8, 31.6, 29.0, 26.3, 25.8, 22.5, 14.0. IR (ATR): ν = 3292, 2955, 2927, 2858, 1643 cm^{-1}. HRMS m/z (ESI) calcd for $C_8H_{18}NO$ (M + H)$^+$: 144.1383; found: 144.1381.

N-ethylheptanamide (**3ar**)

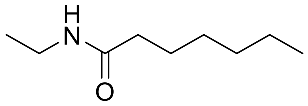

According to the general procedure in 0.2 mmol scale using 1.2 equiv. nitroethane with reaction time of 36 h; purified by flash chromatography (*n*-hexane/ethyl acetate = 3:1), 16.0 mg, 51%, white solid, m. p. = 101 - 102 °C; R_f = 0.2 (*n*-hexane/ethyl acetate = 3:1). ^{1}H NMR (400 MHz, Chloroform-*d*) δ 5.38 (s, 1H), 3.35 – 3.24 (m, 2H), 2.20 – 2.08 (m, 2H), 1.63 (d, *J* = 6.7 Hz, 2H), 1.35 – 1.26 (m, 6H), 1.13 (t, *J* = 7.3 Hz, 3H), 0.88 (t, *J* = 6.8 Hz, 3H). ^{13}C NMR (101 MHz, Chloroform-*d*) δ 173.0, 36.9, 34.3, 31.5, 29.0, 25.8, 22.5, 14.9, 14.0. IR (ATR): ν = 3291, 2957, 2928, 2858, 1644 cm^{-1}. HRMS m/z (ESI) calcd for $C_9H_{20}NO$ (M + H)$^+$: 158.1539; found: 158.1538.

N-propylheptanamide (**3as**)

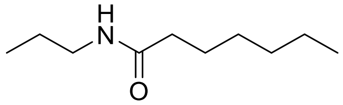

According to the general procedure in 0.2 mmol scale using 1.2 equiv. nitropropane with reaction time of 36 h; purified by flash chromatography (*n*-hexane/ethyl acetate = 5:1), 18.8 mg, 55%, white solid, m. p. = 110 - 111 °C; R_f = 0.2 (*n*-hexane/ethyl acetate = 5:1). ^{1}H NMR (400 MHz, Chloroform-*d*) δ 5.46 (s, 1H), 3.26 – 3.16 (m, 2H), 2.15 (t, *J* = 7.4 Hz, 2H), 1.67 –1.57 (m, 2H), 1.56 – 1.44 (m, 2H), 1.33 – 1.24 (m, 6H), 0.92 (t, *J* = 7.4 Hz, 3H), 0.88 (t, *J* = 7.6 Hz, 3H). ^{13}C NMR (101 MHz, Chloroform-*d*) δ 173.1, 41.2, 37.0, 31.6, 29.0, 25.8, 22.9, 22.5, 14.0, 11.4. IR (ATR): ν = 3291, 2959, 2928, 2873, 2859,1644 cm^{-1}. HRMS m/z (ESI) calcd for $C_{10}H_{22}NO$ (M + H)$^+$: 172.1696; found: 172.1696.

N-cyclopentylheptanamide (**3at**)[5]

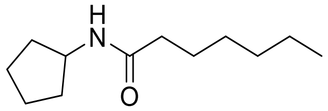

According to the general procedure in 0.2 mmol scale using 1.2 equiv. nitrocyclopentane with reaction time of 24 h; purified by flash chromatography (*n*-hexane / ethyl acetate = 5:1), 18.5 mg, 47% yield, white solid, m. p. = 83 - 85 °C; R_f = 0.3 (*n*-hexane/ethyl acetate = 5:1). ^{1}H NMR (500 MHz, Chloroform-*d*) δ 5.37 (s, 1H), 4.25 – 4.15 (m, 1H), 2.17 – 2.08 (m, 2H), 2.03 – 1.92 (m, 2H), 1.71 – 1.52 (m, 6H), 1.38 – 1.23 (m, 8H), 0.87 (t, *J* = 6.7 Hz, 3H). ^{13}C NMR (126 MHz, Chloroform-*d*) δ 172.7, 51.0, 37.0, 33.2, 31.5, 29.0, 25.8, 23.7, 22.5, 14.0. HRMS m/z (ESI) calcd for $C_{12}H_{24}NO$ (M + H)$^+$: 198.1852, found: 198.1850. IR (ATR): ν = 3302, 2964, 2921, 2872, 2855, 1638 cm^{-1}. HRMS m/z (ESI) calcd for $C_{13}H_{19}NO$ (M + H)$^+$: 332.0506; found: 332.0501.

N-propylbicyclo[2.2.1]hept-5-ene-2-carboxamide (**3au**)

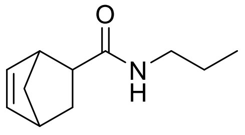

According to the general procedure in 0.2 mmol scale using 1.2 equiv. nitropropane with reaction time of 36 h; purified by flash chromatography (*n*-hexane/ethyl acetate = 3:1), 17.9 mg, 50%, white solid, m. p. = 91 - 92 °C; R_f= 0.3 (*n*-hexane/ethyl acetate = 3:1). ^{1}H NMR (400 MHz, Chloroform-*d*) δ 6.24 (dd, *J* = 5.7, 3.1 Hz, 1H), 5.98 (dd, *J* = 5.7, 2.9 Hz, 1H), 5.36 (s, 1H), 3.23 – 3.09 (m, 3H), 2.95 – 2.90 (m, 1H), 2.89 – 2.83 (m, 1H), 1.90 – 1.97 (m, 1H), 1.54 – 1.46 (m, 2H), 1.46 – 1.43 (m, 1H), 1.31 – 1.25 (m, 2H), 0.90 (t, *J* = 7.4 Hz, 3H). ^{13}C NMR (101 MHz, Chloroform-d) δ 173.1, 137.7, 132.3, 50.0, 46.2, 44.9, 42.7, 41.1, 30.0, 22.9, 11.4. IR (ATR): ν = 3305, 2965, 2923, 2872, 2853, 1648 cm^{-1}. HRMS m/z (ESI) calcd for $C_{11}H_{18}NO$ (M + H)$^+$: 180.1383; found: 180.1380.

(1S,4R)-N-cyclopentyl-4,7,7-trimethyl-3-oxo-2-oxabicyclo[2.2.1]heptane-1-carboxamide (**3av**)

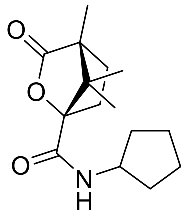

According to the general procedure in 0.2 mmol scale using 1.2 equiv. nitrocyclopentane with reaction time of 36 h; purified by flash chromatography (*n*-hexane/ethyl acetate = 3:1), 24.0 mg, 48%, white solid, m. p. = 123 - 124 °C; R_f = 0.4 (*n*-hexane/ethyl acetate = 3:1). ^{1}H NMR (400 MHz, Chloroform-*d*) δ 6.39 (s, 1H), 4.32 – 4.17 (m, 1H), 2.59 – 2.45 (m, 1H), 2.05 – 1.95 (m, 2H), 1.95 – 1.82 (m, 2H), 1.76 – 1.53 (m, 5H), 1.45 – 1.35 (m, 2H), 1.11 (s, 6H), 0.90 (s, 3H). ^{13}C NMR (101 MHz, Chloroform-*d*) δ 178.5, 166.3, 92.6, 55.3, 53.9, 50.8, 33.5, 32.8, 30.2, 29.1, 23.8, 16.8, 16.5, 9.7, 8.6. IR (ATR): ν = 3360, 2963, 2872, 1787, 1663 cm^{-1}. HRMS m/z (ESI) calcd for $C_{15}H_{24}NO_3$ $(M + H)^+$: 266.1751; found: 266.1742

4-bromo-N-methylbenzamide (**3aw**)

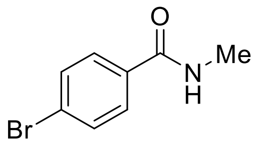

According to the general procedure in 0.2 mmol scale using 2.0 equiv. nitromethane with reaction time of 30 h; purified by flash chromatography (*n*-hexane / ethyl acetate = 5:1), 30.3 mg, 72% yield, white solid, m.p. = 173 – 174 °C; R_f = 0.2 (*n*-hexane / ethyl acetate = 5:1). ^{1}H NMR (400 MHz, Chloroform-*d*) δ 7.65 – 7.58 (m, 2H), 7.57 – 7.49 (m, 2H), 6.37 (s, 1H), 2.98 (d, *J* = 4.9 Hz, 3H). ^{13}C NMR (101 MHz, $CDCl_3$) δ = 167.28, 133.37, 131.72, 128.45, 125.97, 26.86. IR (ATR): ν = 3340, 2923, 2853, 1636, 836 cm^{-1}. HRMS m/z (ESI) calcd for C_8H_9BrNO $(M + H)^+$: 213.9862; found: 213.9857.

4-methoxy-N-methylbenzamide (**3ax**)

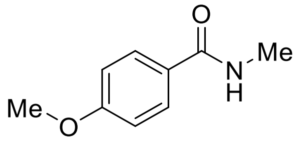

According to the general procedure in 0.2 mmol scale using 2.0 equiv. nitromethane with reaction time of 30 h; purified by flash chromatography (*n*-hexane / ethyl acetate = 5:1), 23.1 mg, 70% yield, white solid, m.p. = 115 – 116 °C; R_f = 0.1 (*n*-hexane / ethyl acetate = 5:1). ^{1}H NMR (400 MHz, Chloroform-*d*) δ 7.83 – 7.57 (m, 2H), 7.06 – 6.74 (m, 2H), 3.83 (s, 3H), 2.98 (d, *J* = 4.8 Hz, 3H). ^{13}C NMR (101 MHz, $CDCl_3$) δ = 167.76, 162.00, 128.57, 126.89, 113.67, 55.34, 26.75. IR (ATR): ν = 3349, 2923, 2852, 1624, 845 cm^{-1}. HRMS m/z (ESI) calcd for $C_9H_{12}NO_2$ $(M + H)^+$: 166.0863; found: 166.0863.

(8S,9S,10R,13S,14S,17S)-10,13-dimethyl-3-oxo-N-phenyl-2,3,6,7,8,9,10,11,12,13,14,15,16,17-tetradecahydro-1H-cyclopenta[a]phenanthrene-17-carboxamide (**3ay**)

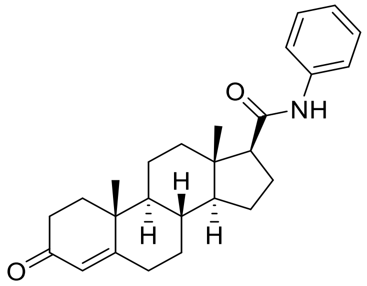

According to the general procedure in 0.2 mmol scale using 1.5 equiv. nitrobenzene with reaction time of 24 h; purified by flash chromatography (*n*-hexane / ethyl acetate = 3:1), 52.4 mg, 67% yield, white solid, m.p. = 204 – 206 ºC; R_f = 0.3 (*n*-hexane / ethyl acetate = 3:1). ^{1}H NMR (400 MHz, Chloroform-*d*) δ 7.58 (d, *J* = 7.3 Hz, 3H), 7.30 (t, *J* = 7.8 Hz, 2H), 7.08 (t, *J* = 7.4 Hz, 1H), 5.75 (s, 1H), 2.52 – 2.22 (m, 6H), 2.08 – 1.98 (m, 2H), 1.94 – 1.53 (m, 6H), 1.51 – 1.23 (m, 3H), 1.19 (s, 3H), 1.16 – 0.89 (m, 3H), 0.80 (s, 3H). ^{13}C NMR (101 MHz, Chloroform-*d*) δ 199.8, 171.4, 171.1, 138.3, 128.9, 124.0, 123.9, 119.9, 57.8, 55.7, 54.0, 44.3, 38.7, 38.2, 35.8, 35.6, 34.0, 32.8, 32.0, 24.4, 23.7, 20.9, 17.3, 13.4. IR (ATR): ν = 3418, 2981, 2943, 2926, 2910, 2899, 2854, 1731, 1666, 1598, 761, 693 cm^{-1}. HRMS m/z (ESI) calcd for $C_{26}H_{34}NO_2$ (M + H)$^+$: 392.2584; found: 392.2575.

2-((4,6-dimethylpyrimidin-2-yl)oxy)-3-methoxy-N,3,3-triphenylpropanamide (**3az**)

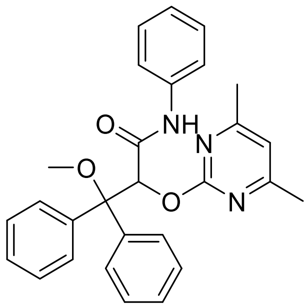

According to the general procedure in 0.2 mmol scale using 2.0 equiv. nitrobenzene with reaction time of 46 h; purified by flash chromatography (*n*-hexane / ethyl acetate = 1:1), 44.2 mg, 49% yield, white solid, m.p. = 98 – 99 ºC; R_f = 0.3 (*n*-hexane / ethyl acetate = 1:1). ^{1}H NMR (400 MHz, Chloroform-*d*) δ 7.64 – 7.57 (m, 2H), 7.40 – 7.37 (m, 1H), 7.35 – 7.31 (m, 3H), 7.29 (dd, *J* = 5.2, 2.1 Hz, 3H), 7.21 – 7.17 (m, 2H), 7.15 (d, *J* = 5.1 Hz, 2H), 7.12 – 7.06 (m, 2H), 7.03 – 6.96 (m, 1H), 6.71 (s, 1H), 6.60 (s, 1H), 3.26 (s, 3H), 2.41 (s, 6H). ^{13}C NMR (101 MHz, Chloroform-*d*) δ 169.7, 166.2, 163.4, 140.3, 140.2, 137.1, 129.0, 128.9, 128.6, 128.0, 127.9, 127.7, 127.6, 124.0, 120.0, 115.4, 84.4, 77.9, 52.5, 23.8. IR (ATR): ν = 3402, 2984, 2931, 2827, 1689, 1597, 755, 692 cm^{-1}. HRMS m/z (ESI) calcd for $C_{28}H_{28}N_3O_3$ (M + H)$^+$: 454.2125; found: 454.2116.

(R)-4-((5S,8R,9S,10S,13R,14S,17R)-10,13-dimethyl-3,7,12-trioxohexadecahydro-1H-cyclopenta[a]phenanthren-17-yl)-N-phenylpentanamide (**3ba**)

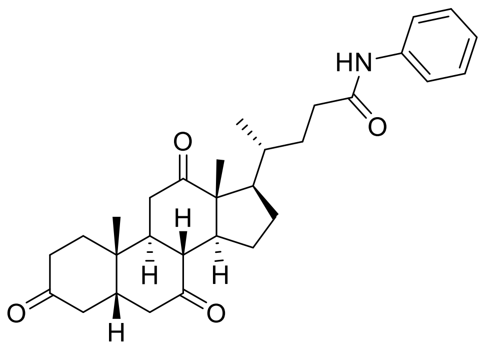

According to the general procedure in 0.2 mmol scale using 1.5 equiv. nitrobenzene with reaction time of 40 h; purified by flash chromatography (*n*-hexane / ethyl acetate = 3:1), 58.2 mg, 61% yield, white solid, m.p. = 248 – 249 °C; R_f = 0.2 (*n*-hexane / ethyl acetate = 3:1). ^{1}H NMR (400 MHz, Chloroform-*d*) δ 7.55 (s, 1H), 7.51 (d, *J* = 8.0 Hz, 2H), 7.30 (t, *J* = 7.8 Hz, 2H), 7.08 (t, *J* = 7.4 Hz, 1H), 2.99 – 2.78 (m, 3H), 2.51 – 2.25 (m, 6H), 2.25 – 2.09 (m, 4H), 2.06 – 1.99 (m, 3H), 1.99 – 1.91 (m, 2H), 1.90 – 1.71 (m, 2H), 1.68 – 1.46 (m, 2H), 1.40 (s, 3H), 1.32 – 1.21 (m, 2H), 1.07 (s, 3H), 0.87 (d, *J* = 6.7 Hz, 3H). ^{13}C NMR (101 MHz, Chloroform-*d*) δ 212.2, 209.2, 208.9, 171.6, 138.1, 128.9, 124.1, 119.7, 56.9, 51.8, 49.0, 46.8, 45.5, 45.4, 45.0, 42.8, 38.6, 36.4, 36.0, 35.3, 35.2, 34.3, 30.8, 27.6, 25.1, 21.9, 18.7, 11.9. IR (ATR): ν = 3360, 2957, 2934, 2875, 1721, 1710, 1696, 1599, 732, 694 cm^{-1}. HRMS m/z (ESI) calcd for $C_{30}H_{40}NO_4$ (M + H)$^+$: 478.2952; found: 478.2941.

2-(4-isobutylphenyl)-N-phenylpropanamide (**3bc**)

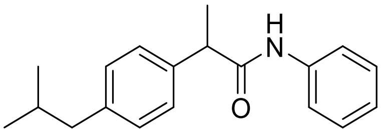

According to the general procedure in 0.2 mmol scale using 1.2 equiv. nitrobenzene with reaction time of 24 h; purified by flash chromatography (*n*-hexane / ethyl acetate = 5:1), 39.9 mg, 71% yield, white solid, m.p. = 130 – 131 °C; R_f = 0.7 (*n*-hexane / ethyl acetate = 5:1). ^{1}H NMR (400 MHz, Chloroform-*d*) δ 7.41 (d, *J* = 7.3 Hz, 2H), 7.29 – 7.23 (m, 4H), 7.15 (d, *J* = 8.0 Hz, 2H), 7.05 (t, *J* = 7.4 Hz, 1H), 3.69 (q, *J* = 7.2 Hz, 1H), 2.47 (d, *J* = 7.2 Hz, 2H), 1.80 – 1.92 (m, 1H), 1.58 (d, *J* = 7.1 Hz, 3H), 0.91 (d, *J* = 6.6 Hz, 6H). ^{13}C NMR (101 MHz, Chloroform-*d*) δ 172.6, 141.1, 138.0, 137.9, 129.8, 128.9, 127.4, 124.1, 119.6, 47.7, 45.0, 30.2, 22.4, 18.5. IR (ATR): ν = 3287, 2949, 2919, 2865, 2850, 1657, 1596, 844, 753, 694 cm^{-1}. HRMS m/z (ESI) calcd for $C_{19}H_{24}NO$ (M + H)$^+$: 282.1852; found: 282.1847.

2-oxo-2-(phenylamino)ethyl 2-(1-(4-chlorobenzoyl)-5-methoxy-2-methyl-1H-indol-3-yl)acetate (**3bd**)

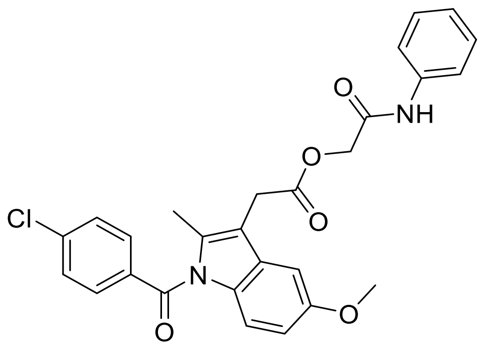

According to the general procedure in 0.2 mmol scale using 1.5 equiv. nitrobenzene with reaction time of 60 h; purified by flash chromatography (*n*-hexane / ethyl acetate = 1:1), 46.2 mg, 47% yield, white solid, m.p. = 148 – 150 °C; R_f = 0.2 (*n*-hexane / ethyl acetate = 1:1). ^{1}H NMR (400 MHz, Chloroform-*d*) δ 7.59 (d, *J* = 8.5 Hz, 2H), 7.42 (d, *J* = 8.5 Hz, 2H), 7.29 (s, 1H), 7.21 (t, *J* = 7.8 Hz, 2H), 7.08 (t, *J* = 7.4 Hz, 1H), 7.05 (d, *J* = 2.4 Hz, 1H), 6.97 (d, *J* = 7.7 Hz, 2H), 6.91 (d, *J* = 9.0 Hz, 1H), 6.74 (dd, *J* = 9.0, 2.5 Hz, 1H), 4.72 (s, 2H), 3.84 (s, 2H), 3.76 (s, 3H), 2.45 (s, 3H). ^{13}C NMR (101 MHz, Chloroform-*d*) δ 168.8, 168.2, 164.6, 156.5, 139.6, 136.6, 136.4, 133.4, 131.2, 130.9, 130.1, 129.2, 128.9, 124.8, 119.8, 115.4, 111.9, 111.3, 101.0, 63.2, 55.7, 30.4, 13.3. IR (ATR): ν = 3307, 2939, 2835, 1726, 1714, 1645, 1606, 829, 734, 691 cm^{-1}. HRMS m/z (ESI) calcd for $C_{27}H_{23}ClN_2O_5Na$ (M + Na)$^+$: 513.1188; found: 513.1171.

2-(1,8-diethyl-1,3,4,9-tetrahydropyrano[3,4-b]indol-1-yl)-N-phenylacetamide (**3be**)

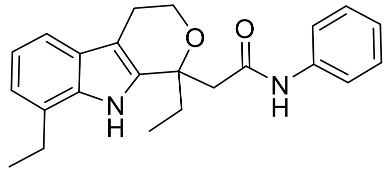

According to the general procedure in 0.2 mmol scale using 1.2 equiv. nitrobenzene with reaction time of 24 h; purified by flash chromatography (*n*-hexane / ethyl acetate = 5:1), 47.8 mg, 66% yield, white solid, m.p. = 113 – 115 °C; R_f = 0.3 (*n*-hexane / ethyl acetate = 5:1). ^{1}H NMR (400 MHz, Chloroform-*d*) δ 9.47 (s, 1H), 8.85 (s, 1H), 7.46 (d, *J* = 7.1 Hz, 2H), 7.31 (d, *J* = 7.8 Hz, 1H), 7.26 (d, *J* = 15.8 Hz, 2H), 7.06 (t, *J* = 7.4 Hz, 1H), 7.01 (t, *J* = 7.5 Hz, 1H), 6.86 (d, *J* = 7.2 Hz, 1H), 4.11 (t, *J* = 7.5 Hz, 2H), 3.27 – 3.01 (m, 2H), 2.99 – 2.80 (m, 2H), 2.66 – 2.53 (m, 1H), 2.50 – 2.36 (m, 1H), 2.27 – 2.13 (m, 1H), 2.11 – 2.00 (m, 1H), 1.00 (t, *J* = 7.5 Hz, 3H), 0.89 (t, *J* = 7.7 Hz, 3H). ^{13}C NMR (101 MHz, Chloroform-*d*) δ 169.7, 137.7, 135.1, 135.0, 129.3, 129.0, 127.2, 126.1, 124.3, 120.1, 119.5, 115.6, 107.8, 76.5, 60.8, 45.3, 31.1, 23.6, 22.4, 13.3, 7.9. IR (ATR): ν = 3301, 2964, 2931, 2876, 1673, 1599, 744, 715, 690 cm^{-1}. HRMS m/z (ESI) calcd for $C_{23}H_{27}N_2O_2$ (M + H)$^+$: 363.2067; found: 363.2060.

(S)-2-ethoxy-4-(2-((3-methyl-1-(2-(piperidin-1-yl)phenyl)butyl)amino)-2-oxoethyl)-N-phenylbenzamide (**3bf**)

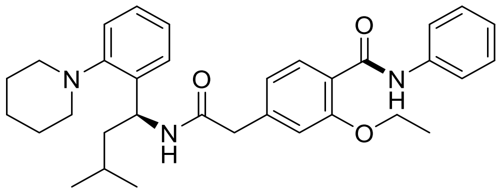

According to the general procedure in 0.2 mmol scale using 1.2 equiv. nitrobenzene with reaction time of 24 h; purified by flash chromatography (*n*-hexane / ethyl acetate = 1:1), 73.2 mg, 71% yield, white solid, m.p. = 115 – 117 °C; R_f = 0.2 (*n*-hexane / ethyl acetate = 1:1). ^{1}H NMR (400 MHz, Chloroform-*d*) δ 10.07 (s, 1H), 8.21 (d, *J* = 7.9 Hz, 1H), 7.66 (d, *J* = 7.3 Hz, 2H), 7.40 – 7.30 (m, 2H), 7.24 – 7.15 (m, 2H), 7.14 – 7.08 (m, 2H), 7.07 – 7.01 (m, 1H), 6.99 – 6.87 (m, 3H), 5.39 (td, *J* = 8.7, 6.4 Hz, 1H), 4.26 – 4.01 (m, 3H), 3.54 (s, 2H), 2.95 (d, *J* = 11.2 Hz, 2H), 2.61 (t, *J* = 9.3 Hz, 2H), 2.04 (s, 1H), 1.81 – 1.68 (m, 2H), 1.56 (t, *J* = 6.9 Hz, 5H), 1.51 – 1.37 (m, 2H), 1.25 (t, *J* = 7.1 Hz, 1H), 1.25 (t, *J* = 7.1 Hz, 1H), 0.95 – 0.89 (m, 6H). ^{13}C NMR (101 MHz, Chloroform-*d*) δ 168.7, 162.9, 156.8, 152.5, 141.1, 138.7, 138.5, 132.7, 129.0, 127.8, 127.7, 125.0, 124.0, 122.7, 122.1, 119.9, 113.0, 65.0, 60.3, 55.0, 49.8, 46.6, 44.0, 26.7, 25.3, 24.1, 22.8, 22.5, 21.0, 14.8. IR (ATR): ν = 3343, 2933, 2867, 1733, 1641, 1598, 859, 825, 753, 690 cm^{-1}. HRMS m/z (ESI) calcd for $C_{32}H_{40}N_3O_3$ (M + H)$^+$: 514.3064; found: 514.3061.

benzyl (1-(phenylcarbamoyl)cyclopropyl)carbamate (**3bg**)

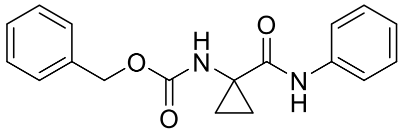

According to the general procedure in 0.2 mmol scale using 1.5 equiv. nitrobenzene with reaction time of 24 h; purified by flash chromatography (*n*-hexane / ethyl acetate = 5:1), 32.2 mg, 52% yield, white solid, m.p. = 135 – 137 °C; R_f = 0.1 (*n*-hexane / ethyl acetate = 5:1). ^{1}H NMR (400 MHz, Chloroform-*d*) δ 8.35 (s, 1H), 7.46 – 7.40 (m, 2H), 7.33 (d, *J* = 9.3 Hz, 3H), 7.31 – 7.23 (m, 3H), 7.09 (t, *J* = 7.4 Hz, 1H), 5.57 (s, 1H), 5.15 (s, 2H), 1.69 – 1.62 (m, 2H), 1.12 – 1.03 (m, 2H). ^{13}C NMR (101 MHz, Chloroform-*d*) δ 169.9, 156.6, 137.6, 135.8, 133.7, 128.9, 128.6, 128.4, 124.3, 120.0, 67.5, 36.4, 29.7, 17.9. IR (ATR): ν = 3308, 2958, 1713, 1665, 1599, 755, 694 cm^{-1}. HRMS m/z (ESI) calcd for $C_{18}H_{19}N_2O_3$ (M + H)$^+$: 311.1390; found: 311.1381.

5-(tert-butyl) 1-(4-heptanamidophenyl) (tert-butoxycarbonyl)-L-glutamate (**3bh**)

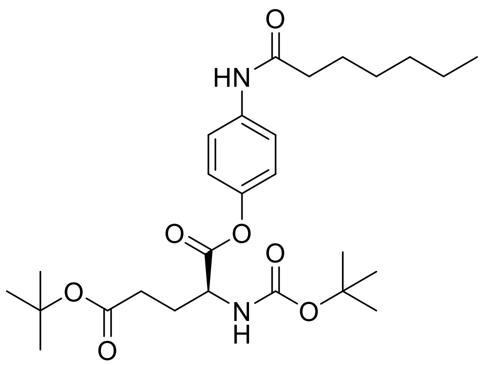

According to the general procedure in 0.2 mmol scale using 1.2 equiv. 5-(tert-butyl) 1-(4-nitrophenyl) (tert-butoxycarbonyl)-L-glutamate with reaction time of 36 h; purified by flash

chromatography (DCM / methanol 30:1), 61.7 mg, 61% yield, white solid, m.p. = 105 – 107 °C; R_f = 0.4 (DCM / methanol 30:1). ^{1}H NMR (400 MHz, Chloroform-*d*) δ 7.52 (d, *J* = 8.5 Hz, 2H), 7.38 (s, 1H), 7.04 (d, *J* = 8.9 Hz, 2H), 5.21 (d, *J* = 8.4 Hz, 1H), 4.52 (dt, *J* = 13.4, 6.4 Hz, 1H), 2.42 (q, *J* = 7.0 Hz, 2H), 2.34 (t, *J* = 7.5 Hz, 2H), 2.30 – 2.19 (m, 1H), 2.15 – 2.01 (m, 1H), 1.76 – 1.67 (m, 2H), 1.46 (s, 18H), 1.40 – 1.28 (m, 6H), 0.89 (t, *J* = 6.7 Hz, 3H). ^{13}C NMR (101 MHz, Chloroform-*d*) δ 172.0, 171.4, 171.2, 155.4, 146.4, 136.0, 121.7, 120.7, 81.0, 80.2, 53.3, 37.7, 31.6, 31.5, 28.9, 28.3, 28.1, 27.4, 25.5, 22.5, 14.0. IR (ATR): ν = 3320, 2959, 2930, 2859, 1762, 1717, 1669, 846 cm^{-1}. HRMS m/z (ESI) calcd for $C_{27}H_{42}N_2NaO_7$ (M + Na)$^+$: 529.2884; found: 529.2880.

methyl N2-acetyl-N4-phenyl-L-asparaginyl-L-phenylalaninate (**3bi**)

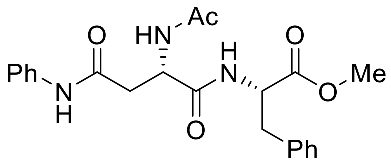

According to the general procedure in 0.2 mmol scale using 1.2 equiv. nitrobenzene with reaction time of 48 h; 51.8 mg, 63%, purified by flash chromatography (dichloromethane/methanol 50:1), white solid, m. p. = 267 - 268 °C; R_f = 0.5 (dichloromethane/methanol 50:1). ^{1}H NMR (400 MHz, DMSO-d_6) δ 9.91 (s, 1H), 8.19 (d, *J* = 7.5 Hz, 1H), 8.13 (d, *J* = 8.0 Hz, 1H), 7.58 (d, *J* = 7.6 Hz, 2H), 7.31 – 7.25 (m, 4H), 7.24 – 7.18 (m, 3H), 7.03 (t, *J* = 7.4 Hz, 1H), 4.73 (td, *J* = 8.1, 5.5 Hz, 1H), 4.47 (td, *J* = 8.0, 5.8 Hz, 1H), 3.57 (s, 3H), 3.10 – 2.90 (m, 2H), 2.82 – 2.72 (m, 1H), 2.62 – 2.52 (m, 1H), 1.82 (s, 3H). ^{13}C NMR (101 MHz, DMSO-d_6) δ 172.1, 171.7, 169.8, 168.5, 139.6, 137.4, 129.6, 129.1, 128.7, 127.0, 123.5, 119.5, 54.1, 52.3, 49.7, 38.6, 37.0, 23.0. IR (ATR): ν = 3293, 3256, 2998, 2953, 1737, 1714, 1649, 1597, 749, 697 cm^{-1}. HRMS m/z (ESI) calcd for $C_{22}H_{26}N_3O_5$ (M + H)$^+$: 412.1867; found: 412.1855.

tert-butyl (S)-(1-oxo-1-((2-oxo-2-(phenylamino)ethyl)amino)propan-2-yl)carbamate (**3bj**)

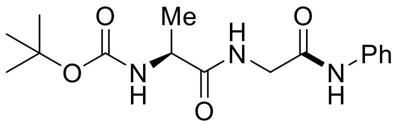

According to the general procedure in 0.2 mmol scale using 1.2 equiv. nitrobenzene with reaction time of 48 h; purified by flash chromatography (dichloromethane/methanol 50:1), 30.8 mg, 48%, white solid, m. p. = 233 - 234 °C; R_f = 0.5 (dichloromethane/methanol 50:1). ^{1}H NMR (400 MHz, Chloroform-*d*) δ 8.70 (s, 1H), 7.61 (d, *J* = 7.9 Hz, 2H), 7.29 (t, *J* = 7.9 Hz, 2H), 7.09 (t, *J* = 7.4 Hz, 2H), 5.16 (s, 1H), 4.20 – 4.13 (m, 1H), 4.11 (t, *J* = 5.7 Hz, 2H), 1.42 (s, 9H), 1.39 (d, *J* = 6.4 Hz, 3H). ^{13}C NMR (101 MHz, Chloroform-*d*) δ 173.5, 167.2, 156.0, 137.7, 128.8, 124.4, 120.2, 80.9, 51.1, 44.0, 29.7, 28.3. IR (ATR): ν = 3252, 2982, 2935, 1738, 1685, 1656, 1599, 755, 694 cm^{-1}. HRMS m/z (ESI) calcd for $C_{16}H_{23}N_3NaO_4$ (M + Na)$^+$: 344.1581; found: 344.1575.

6 NMR spectra of products

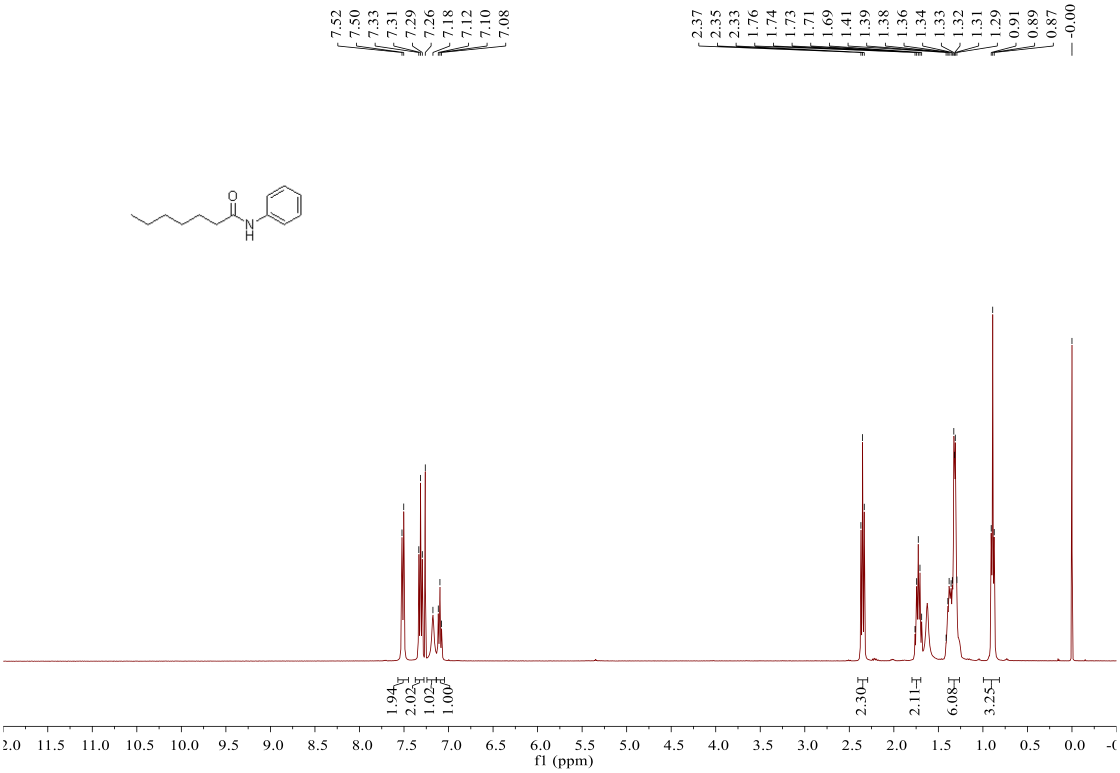

Supplementary Figure 7. ^{1}H NMR spectrum for compound **3a**

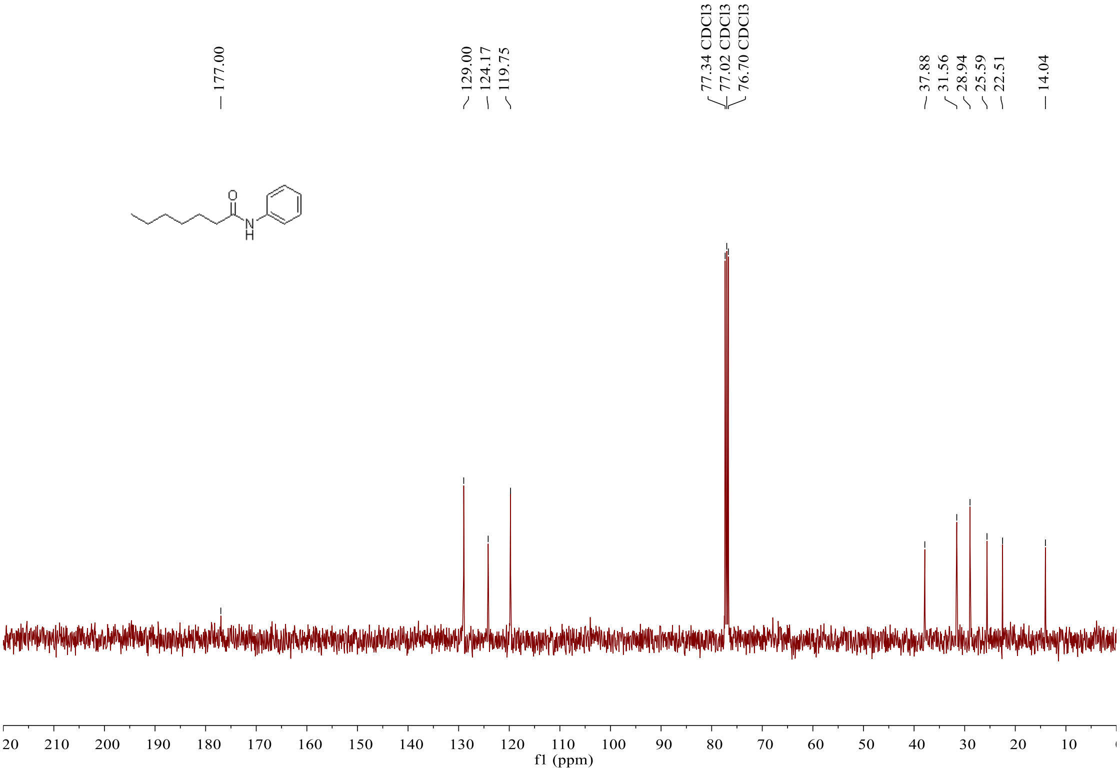

Supplementary Figure 8. ^{13}C NMR spectrum for compound **3a**

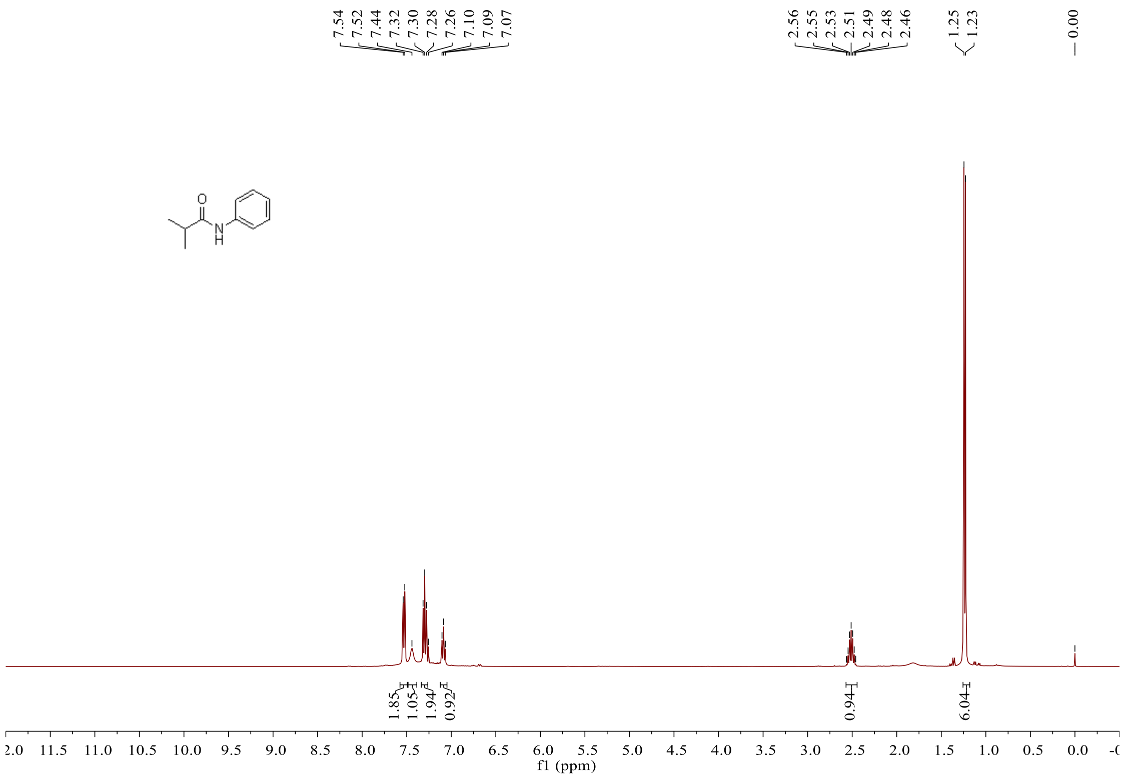

Supplementary Figure 9. ^{1}H NMR spectrum for compound **3b**

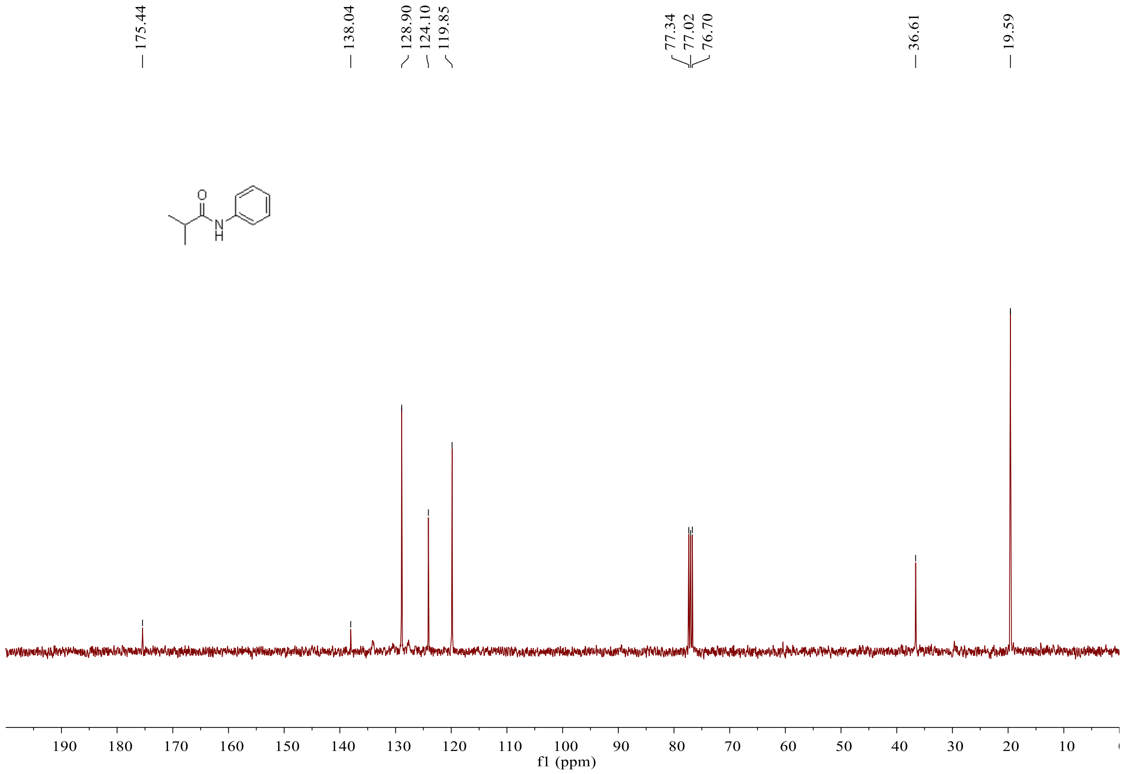

Supplementary Figure 10. ^{13}C NMR spectrum for compound **3b**

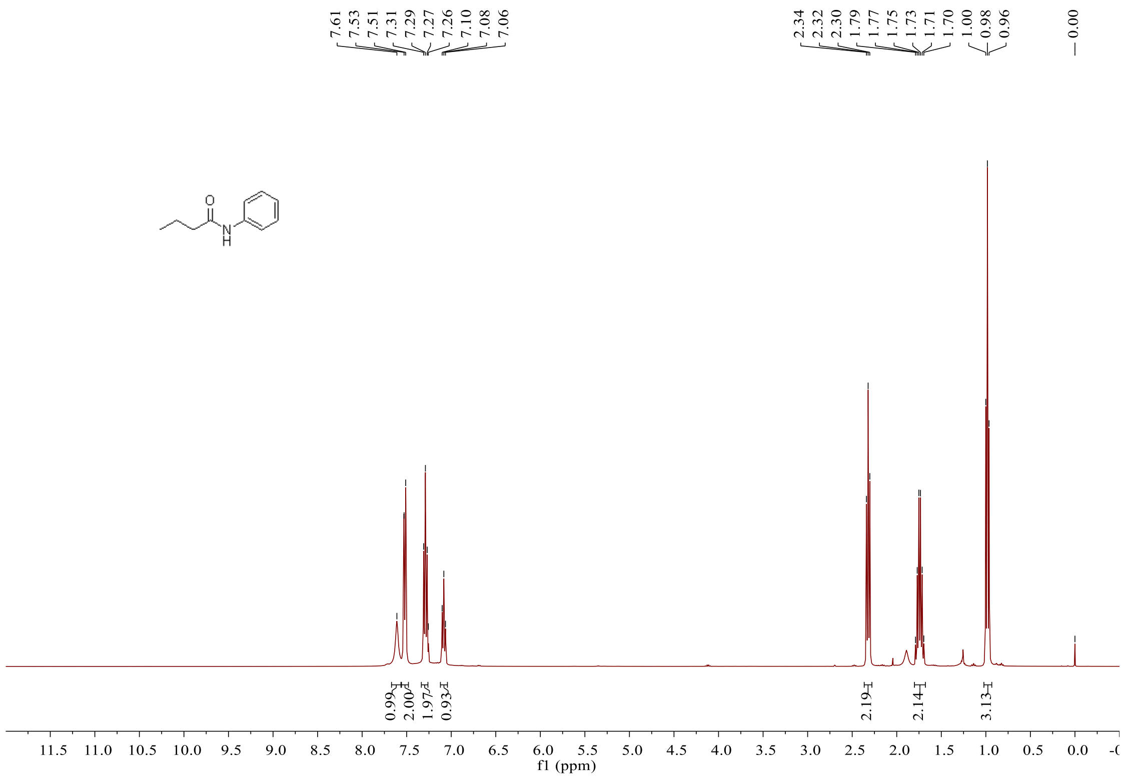

Supplementary Figure 11. ^{1}H NMR spectrum for compound **3c**

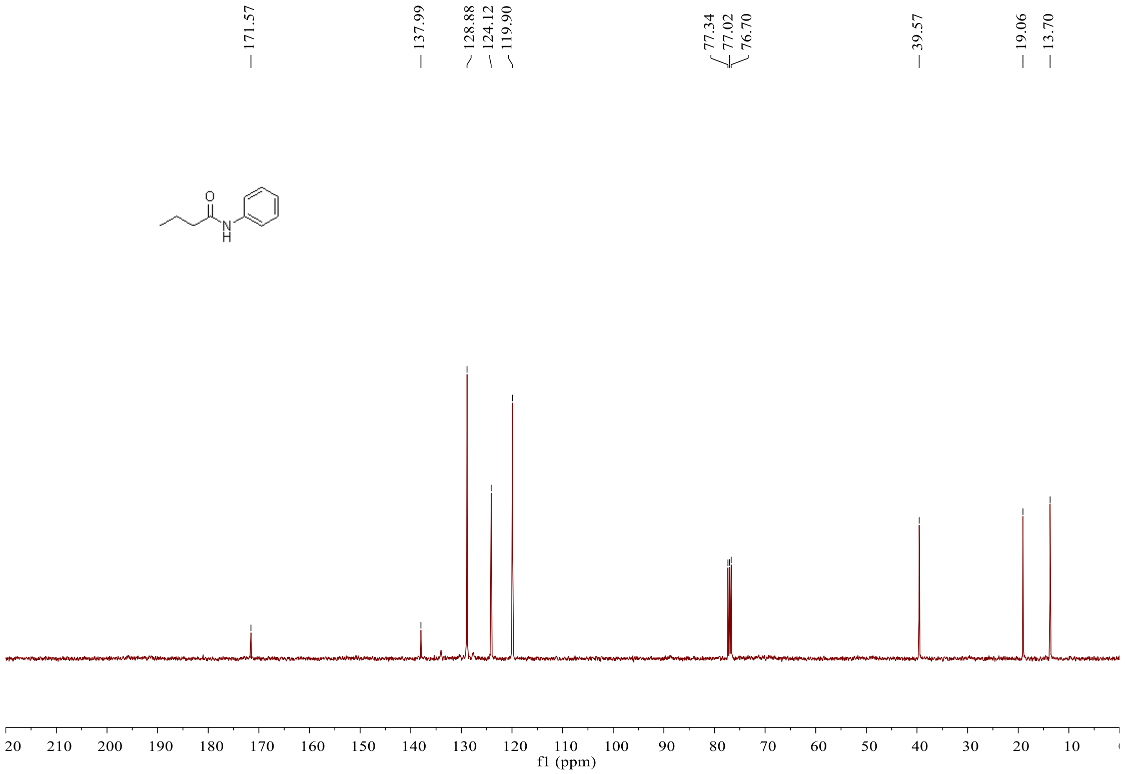

Supplementary Figure 12. ^{13}C NMR spectrum for compound **3c**

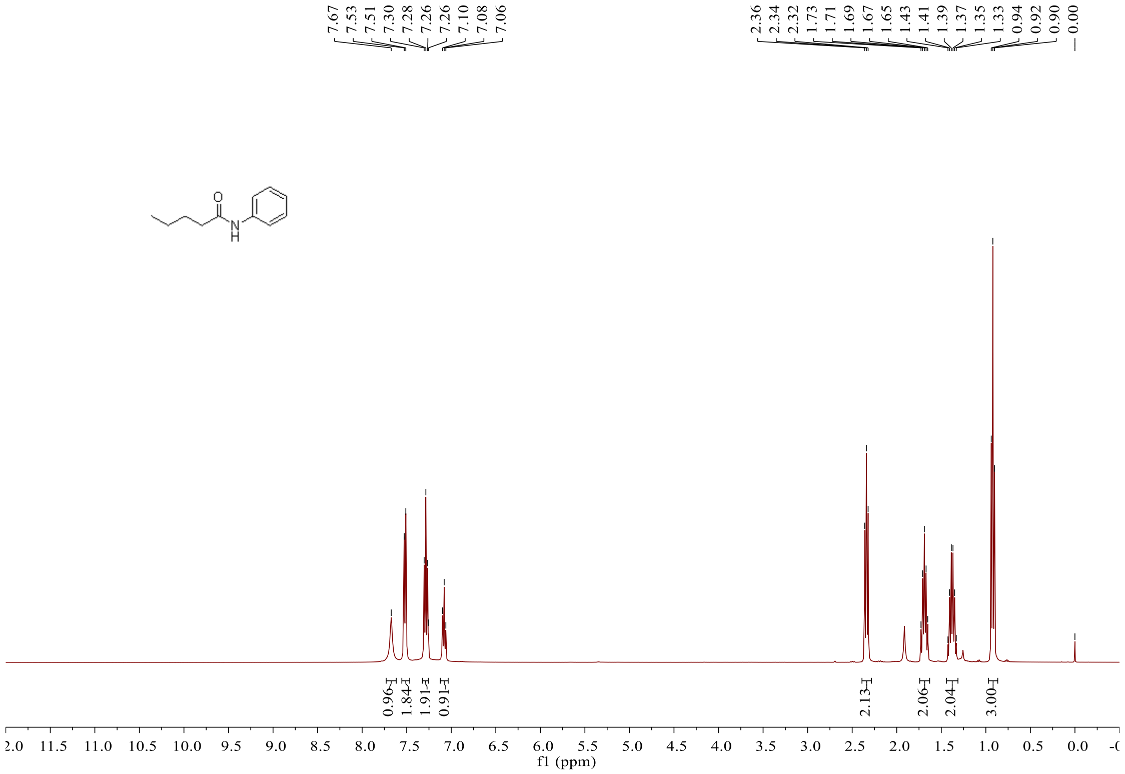

Supplementary Figure 13. ^{1}H NMR spectrum for compound **3d**

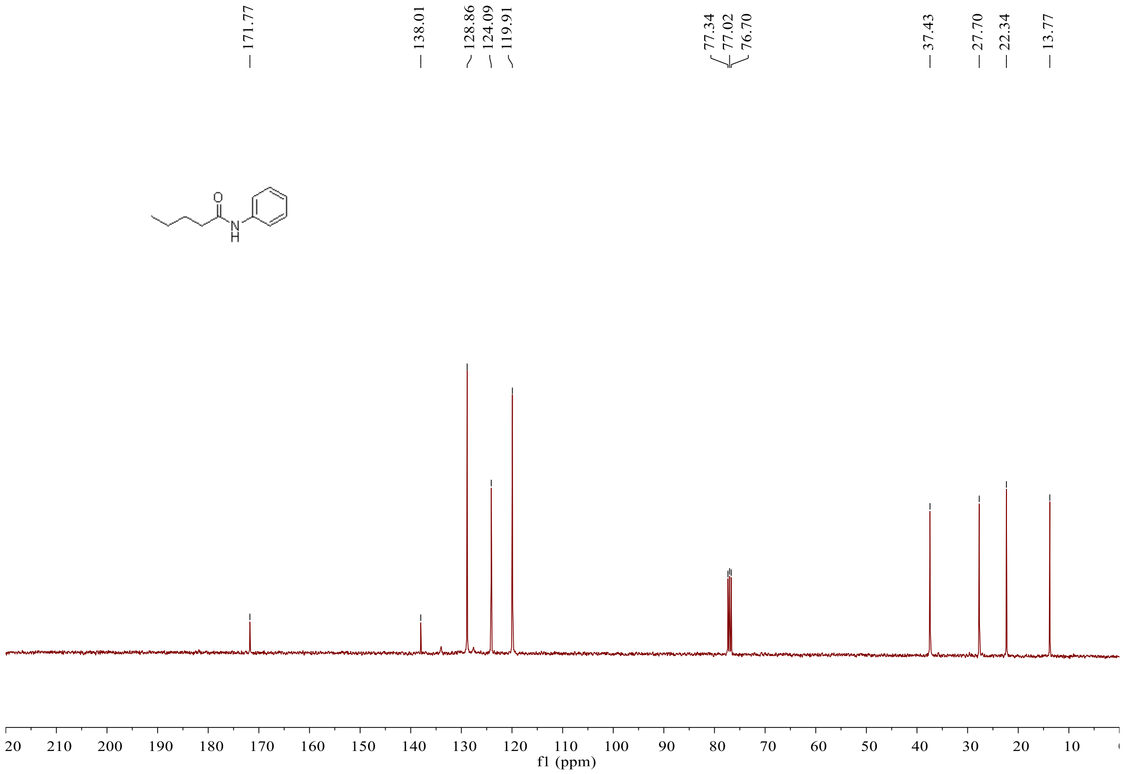

Supplementary Figure 14. ^{13}C NMR spectrum for compound **3d**

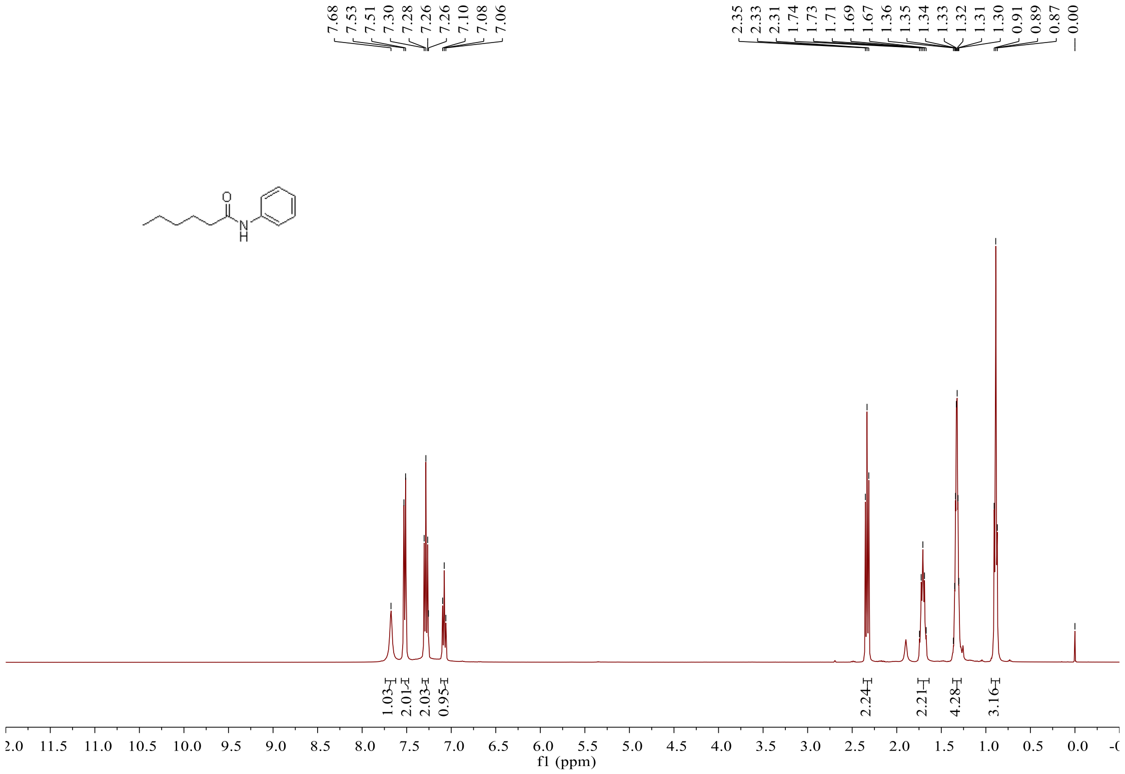

Supplementary Figure 15. ^{1}H NMR spectrum for compound **3e**

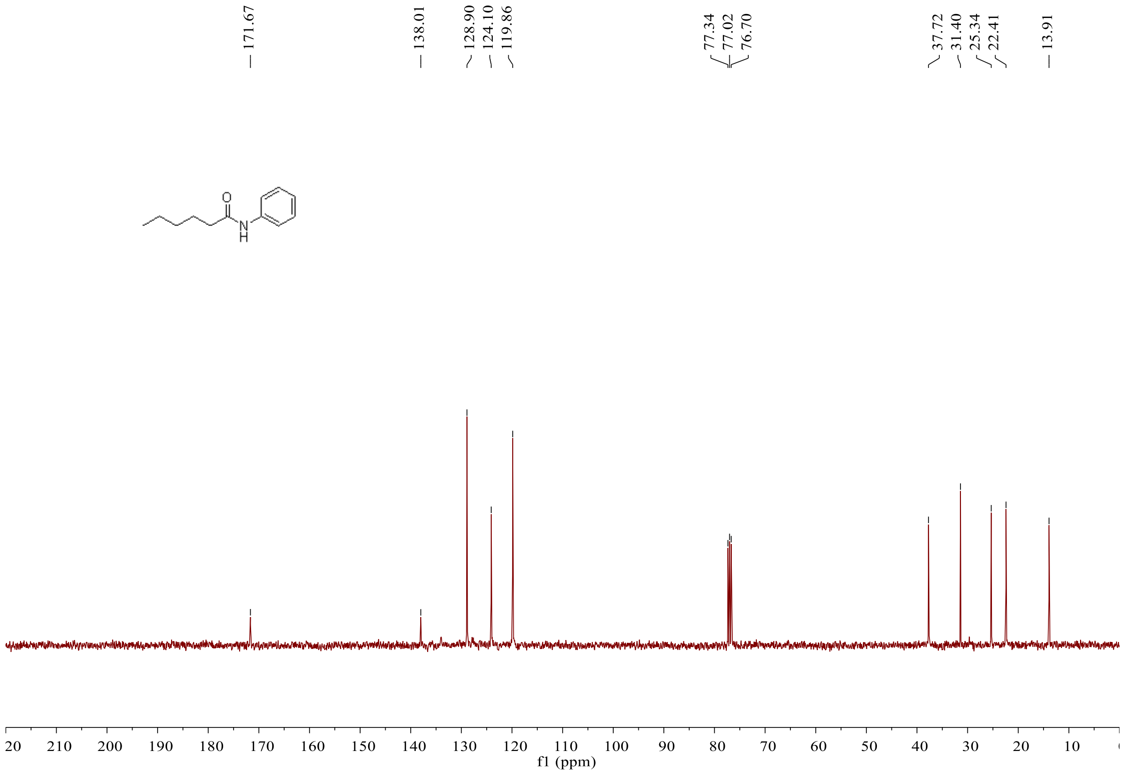

Supplementary Figure 16. ^{13}C NMR spectrum for compound **3e**

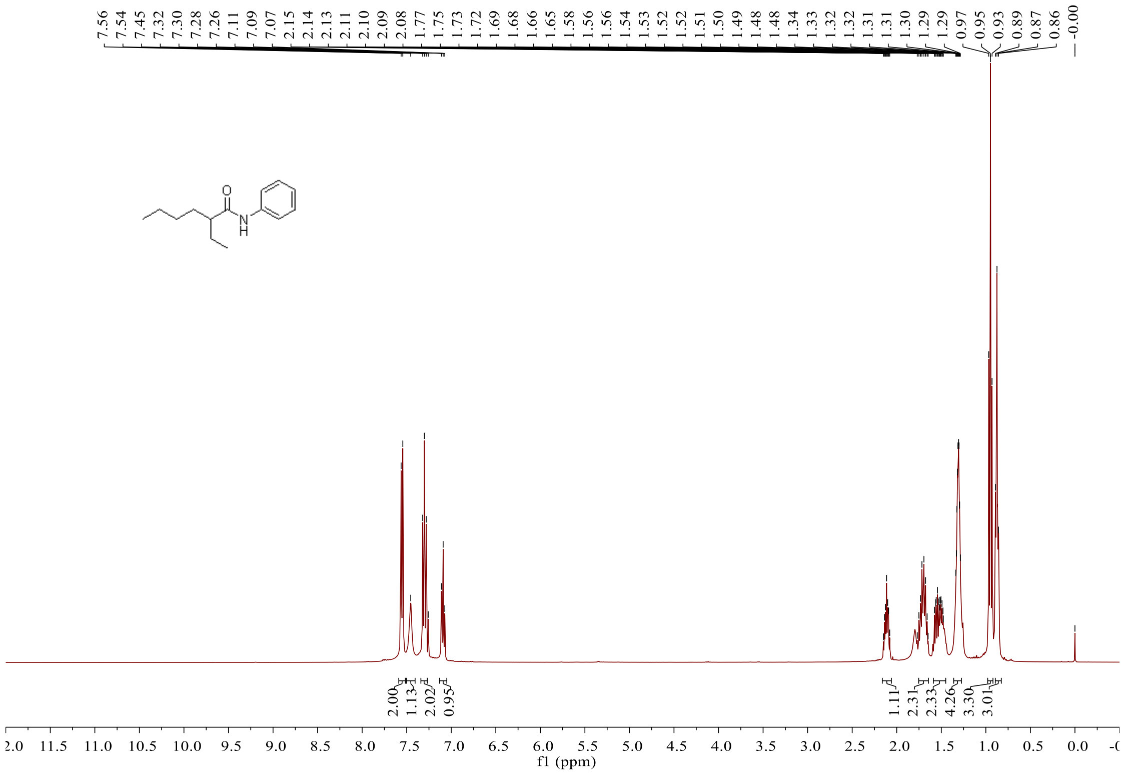

Supplementary Figure 17.^{1}H NMR spectrum for compound **3f**

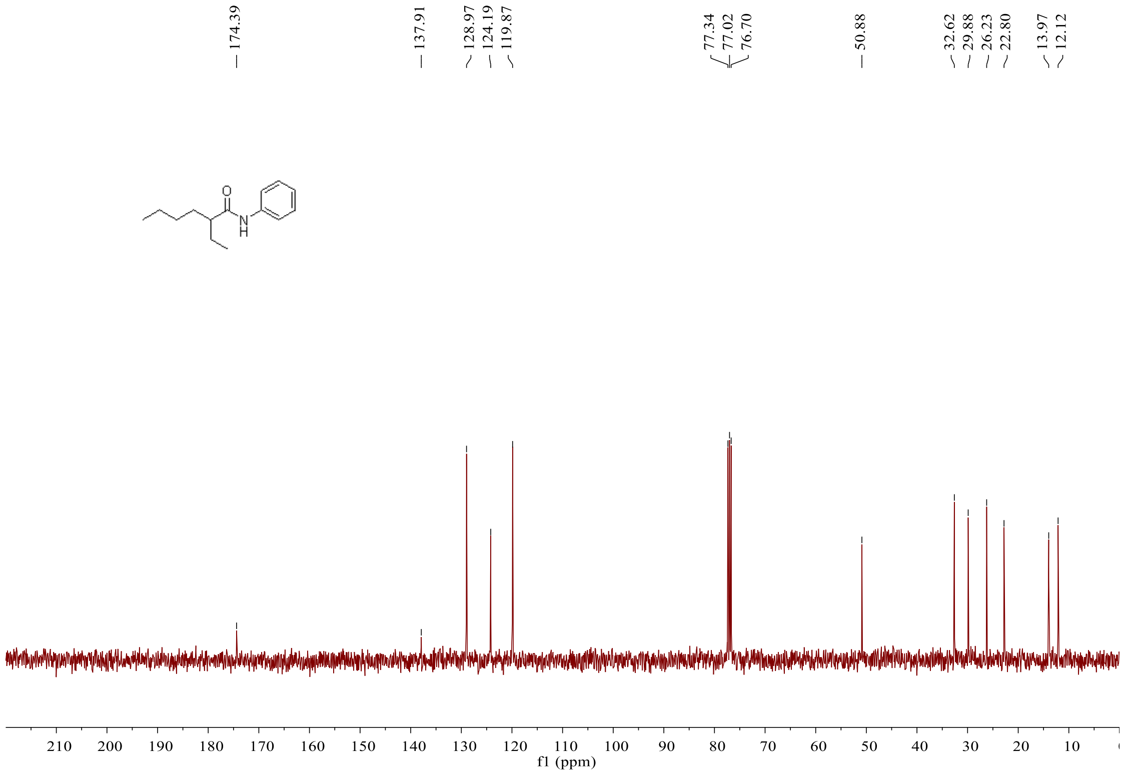

Supplementary Figure 18. ^{13}C NMR spectrum for compound **3f**

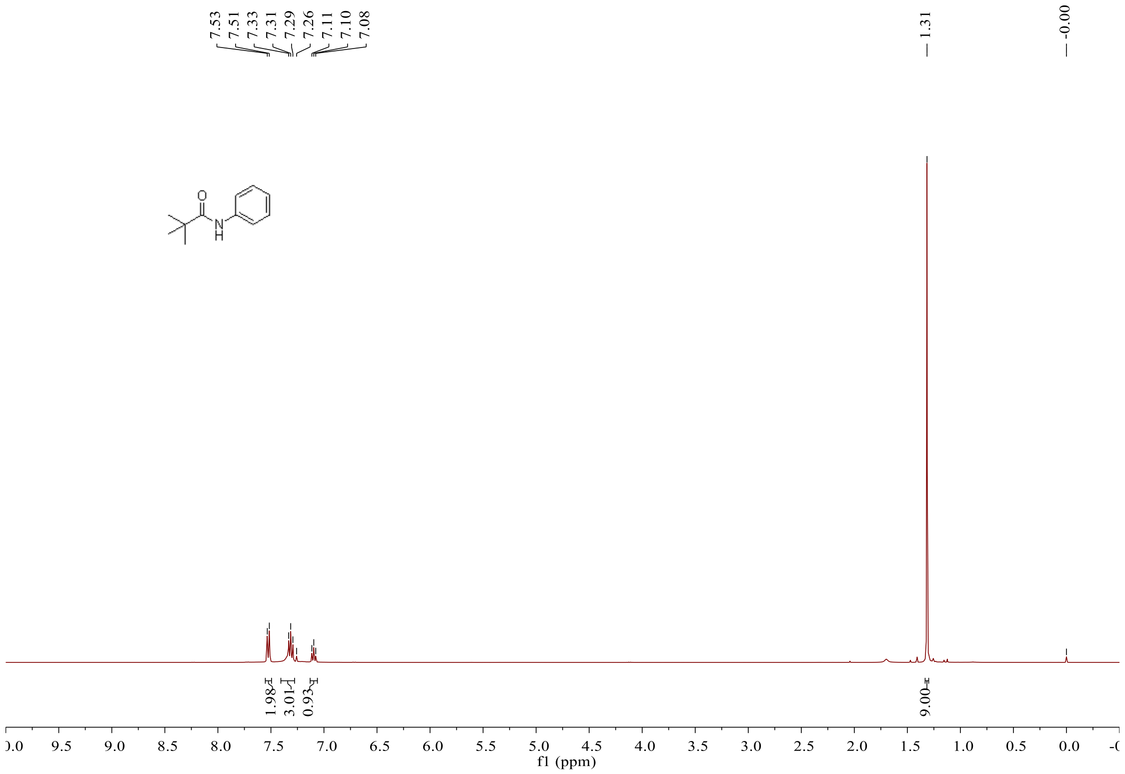

Supplementary Figure 19. ^{1}H NMR spectrum for compound **3g**

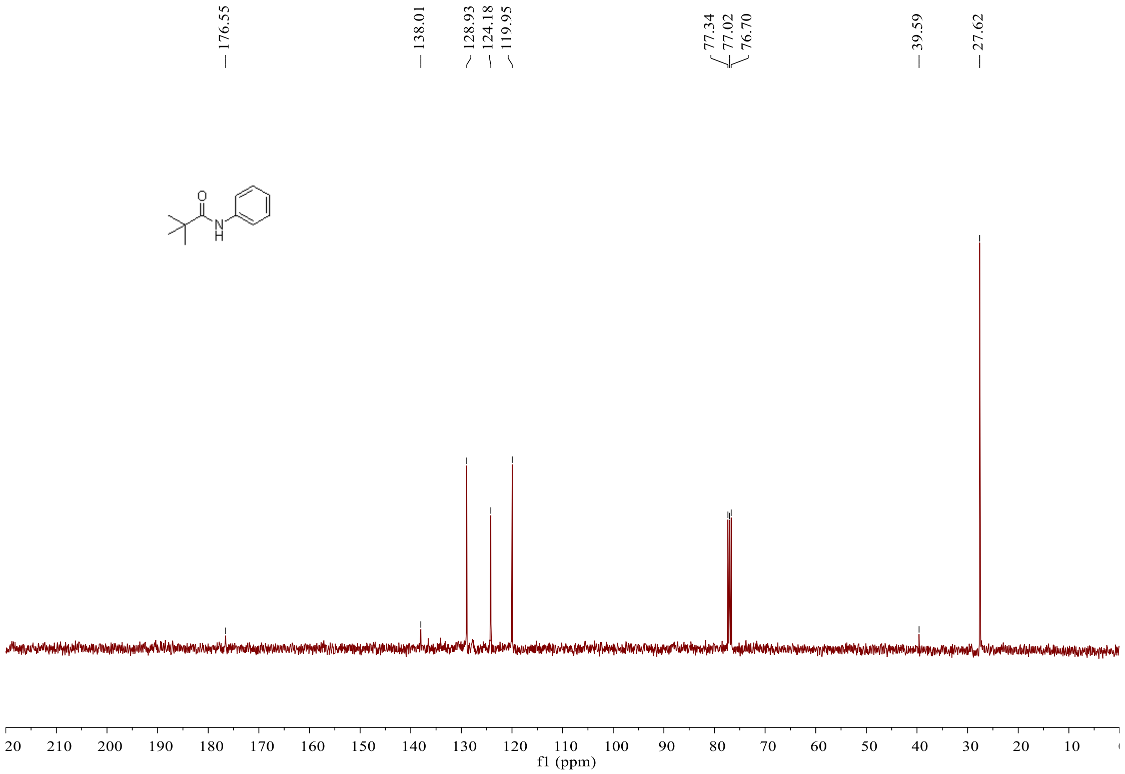

Supplementary Figure 20. ^{13}C NMR spectrum for compound **3g**

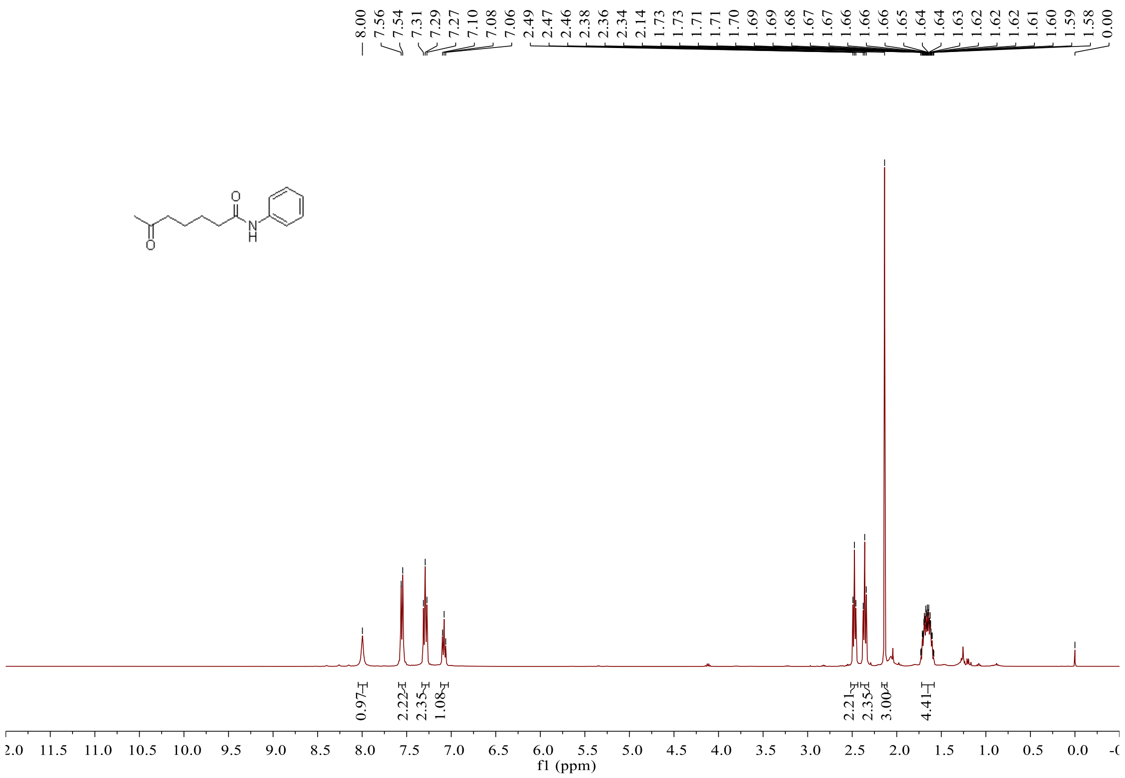

Supplementary Figure 21. ^{1}H NMR spectrum for compound **3h**

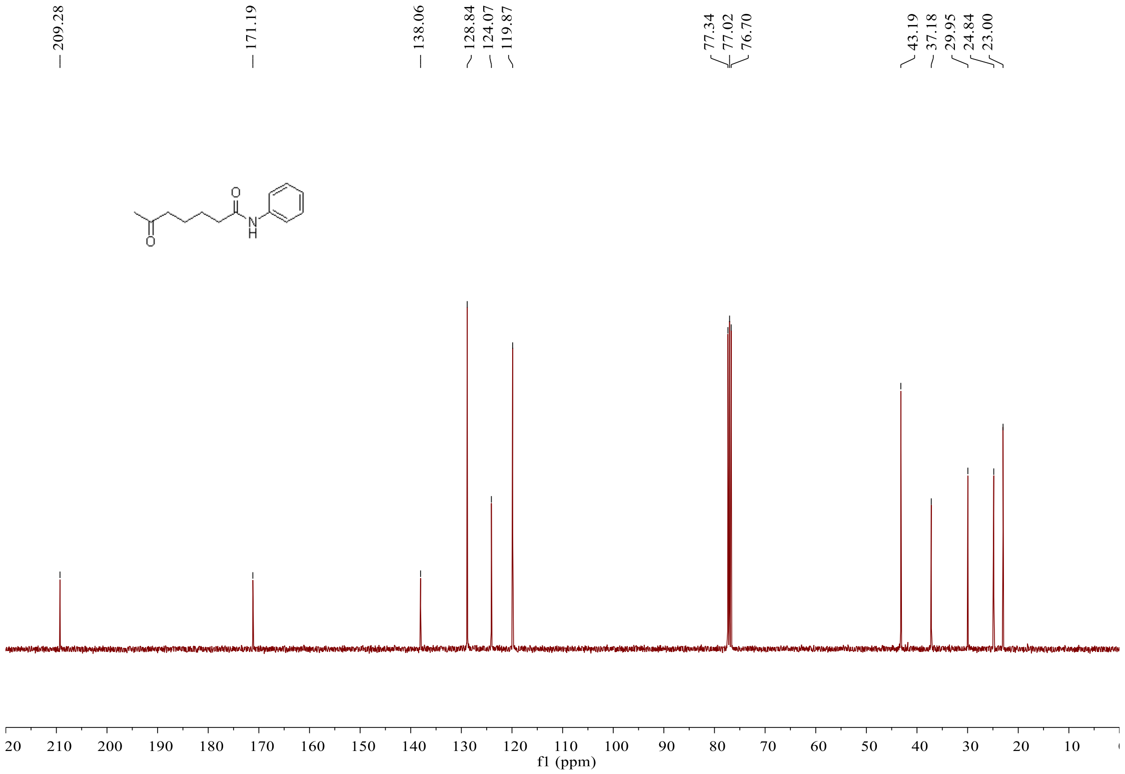

Supplementary Figure 22. ^{13}C NMR spectrum for compound **3h**

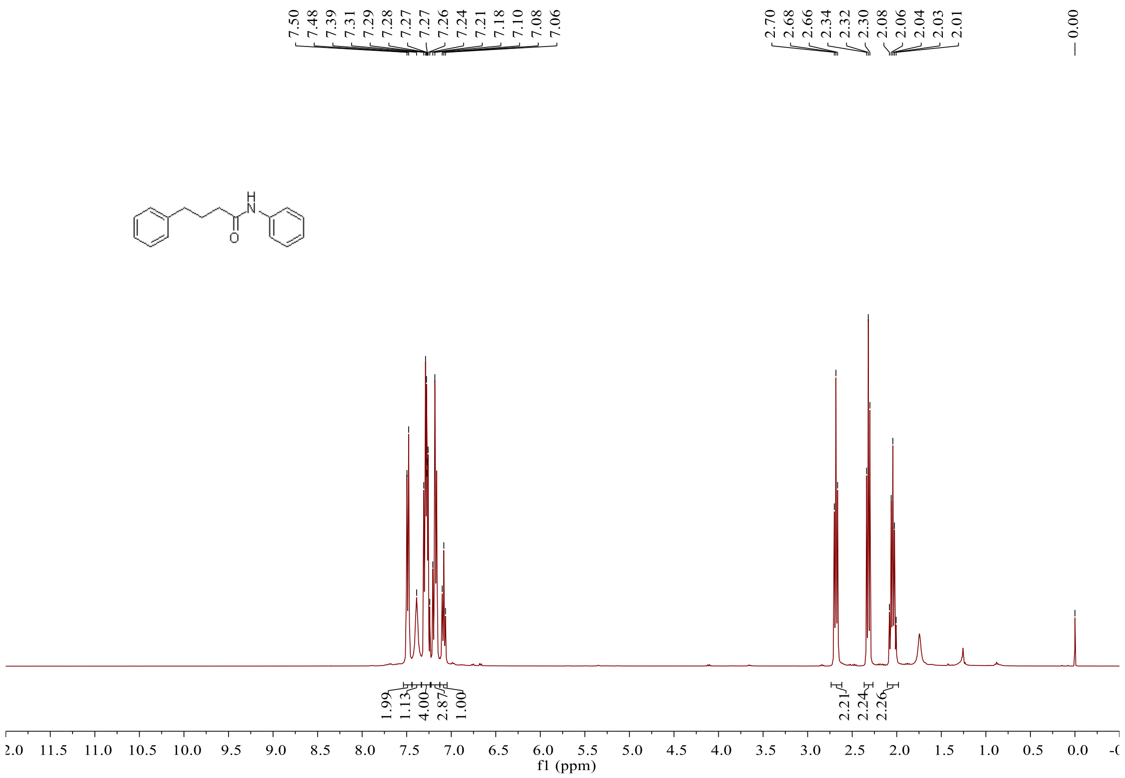

Supplementary Figure 23. ^{1}H NMR spectrum for compound **3i**

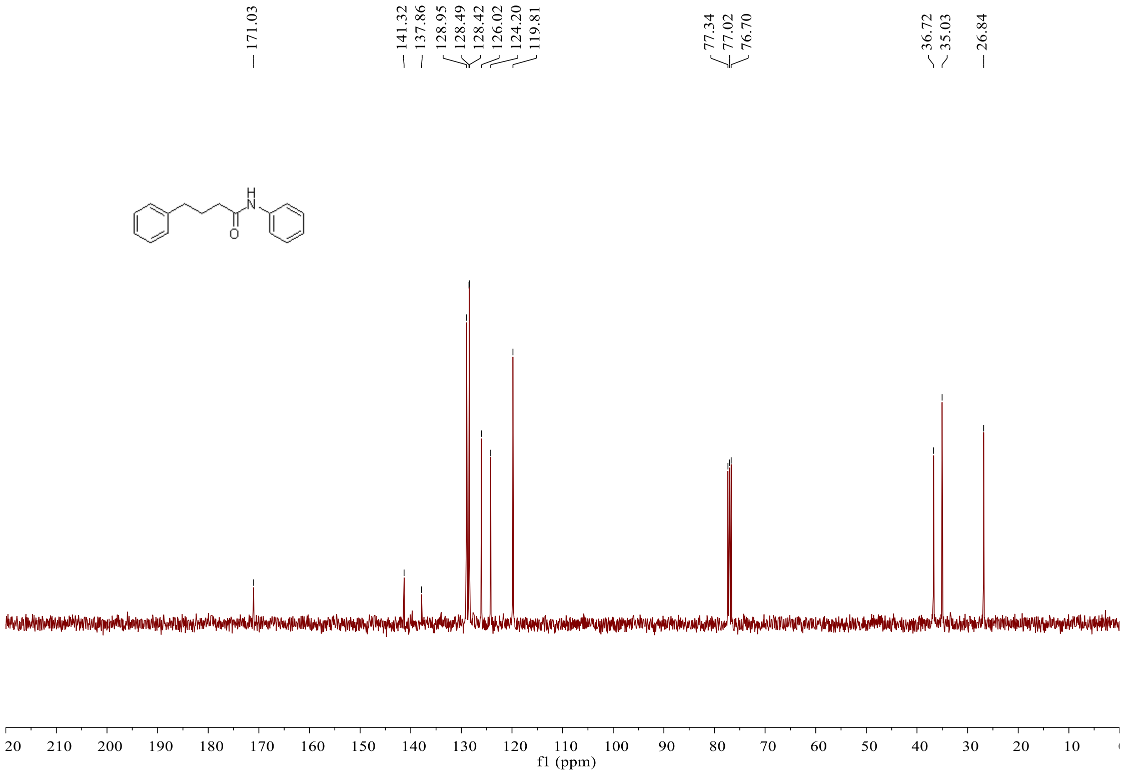

Supplementary Figure 24. ^{13}C NMR spectrum for compound **3i**

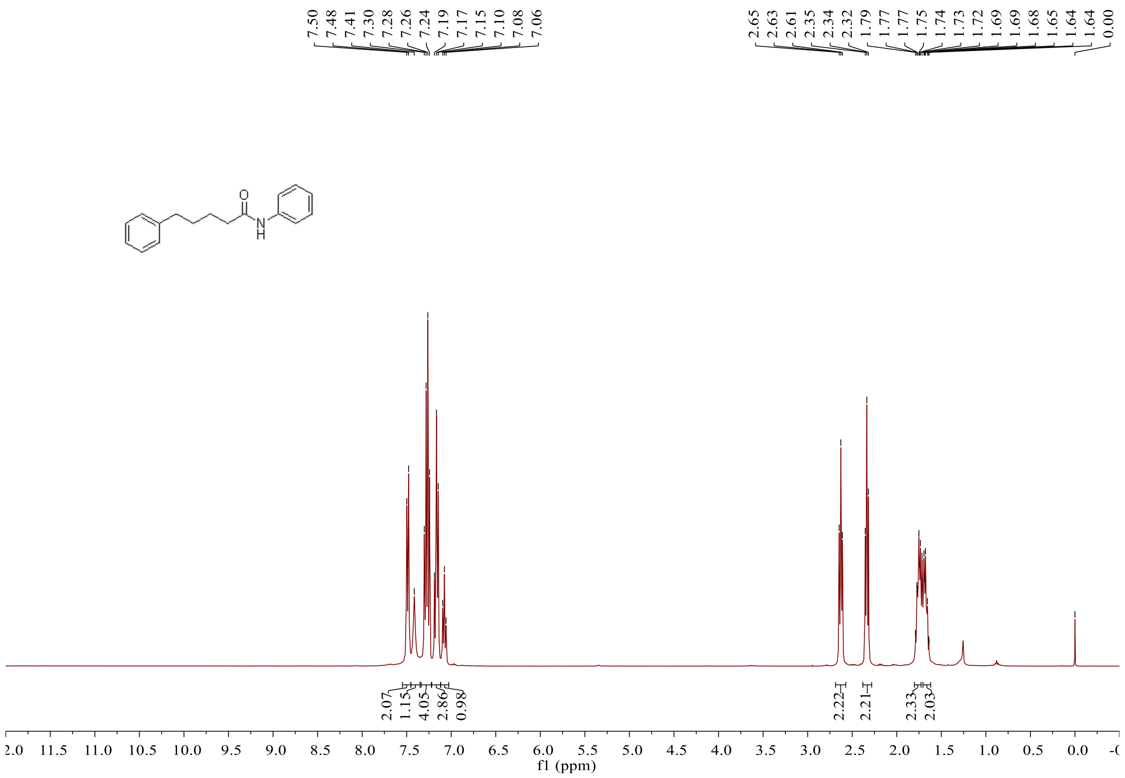

Supplementary Figure 25. ^{1}H NMR spectrum for compound **3j**

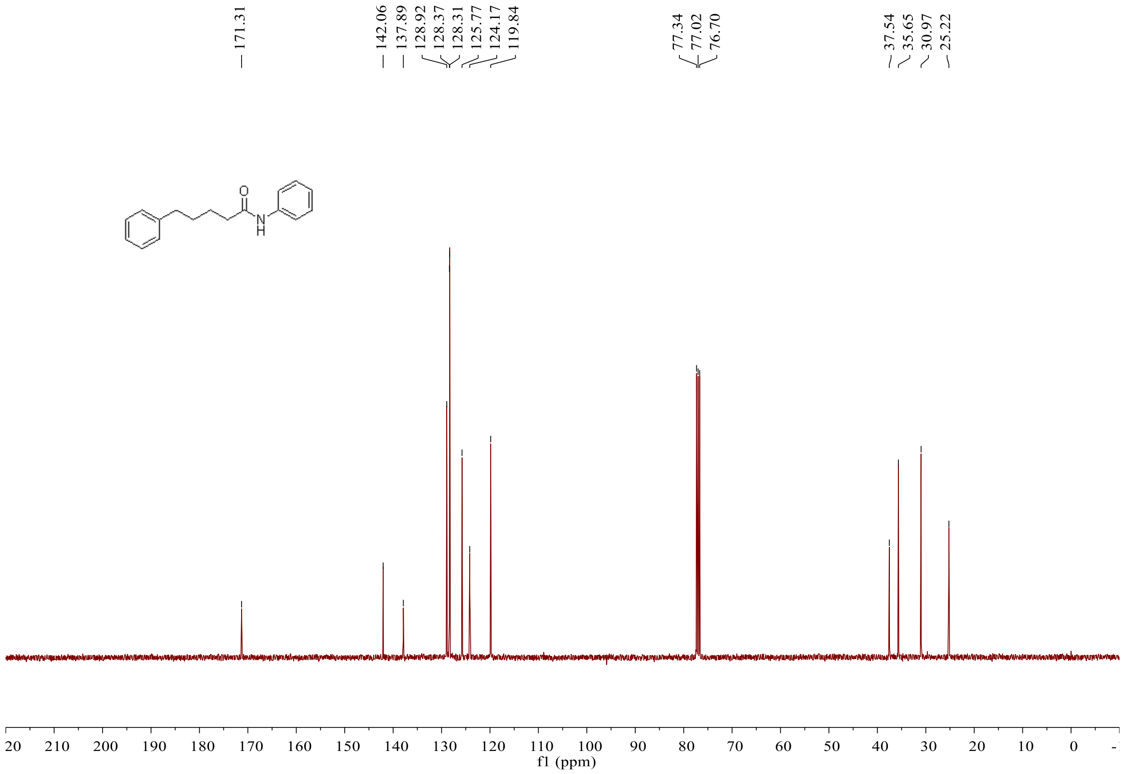

Supplementary Figure 26. ^{13}C NMR spectrum for compound **3j**

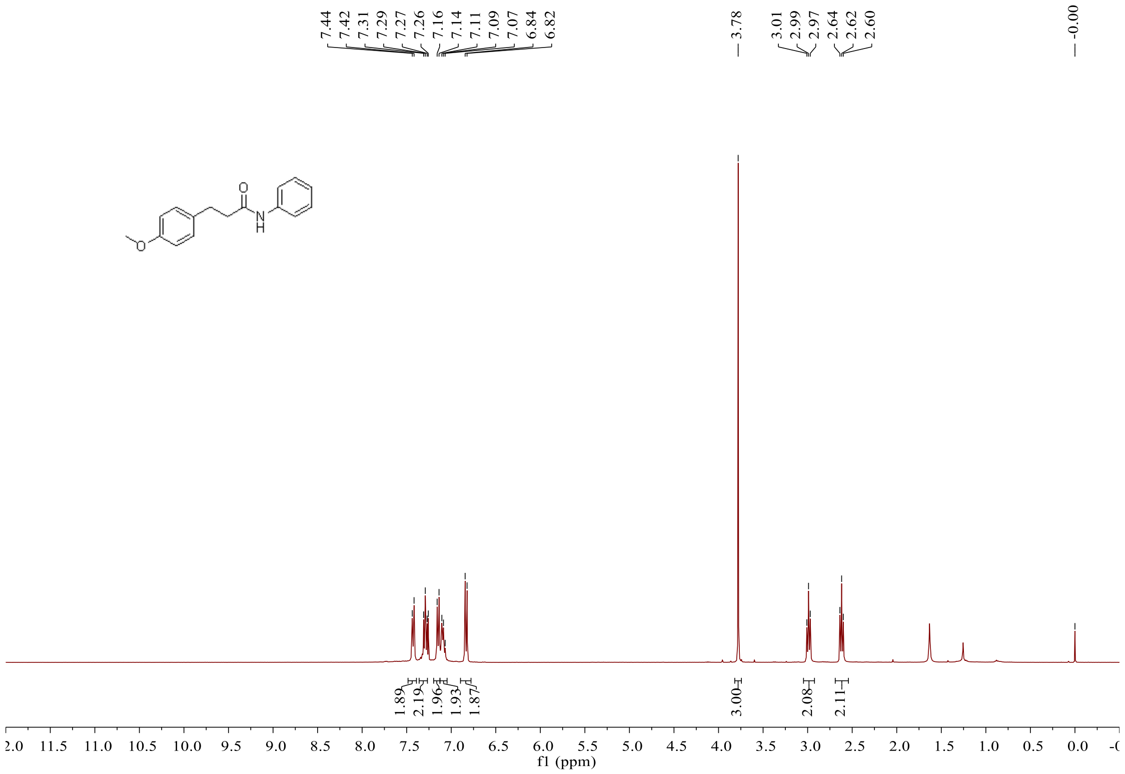

Supplementary Figure 27. ^{1}H NMR spectrum for compound **3k**

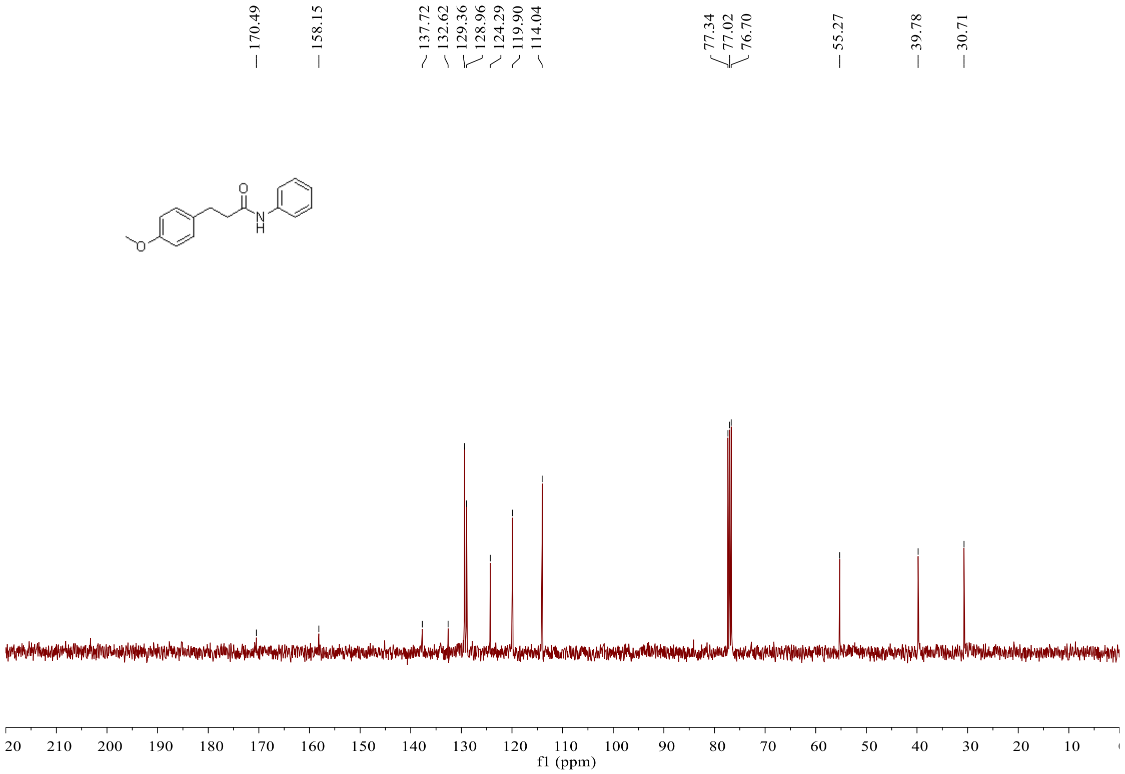

Supplementary Figure 28. ^{13}C NMR spectrum for compound **3k**

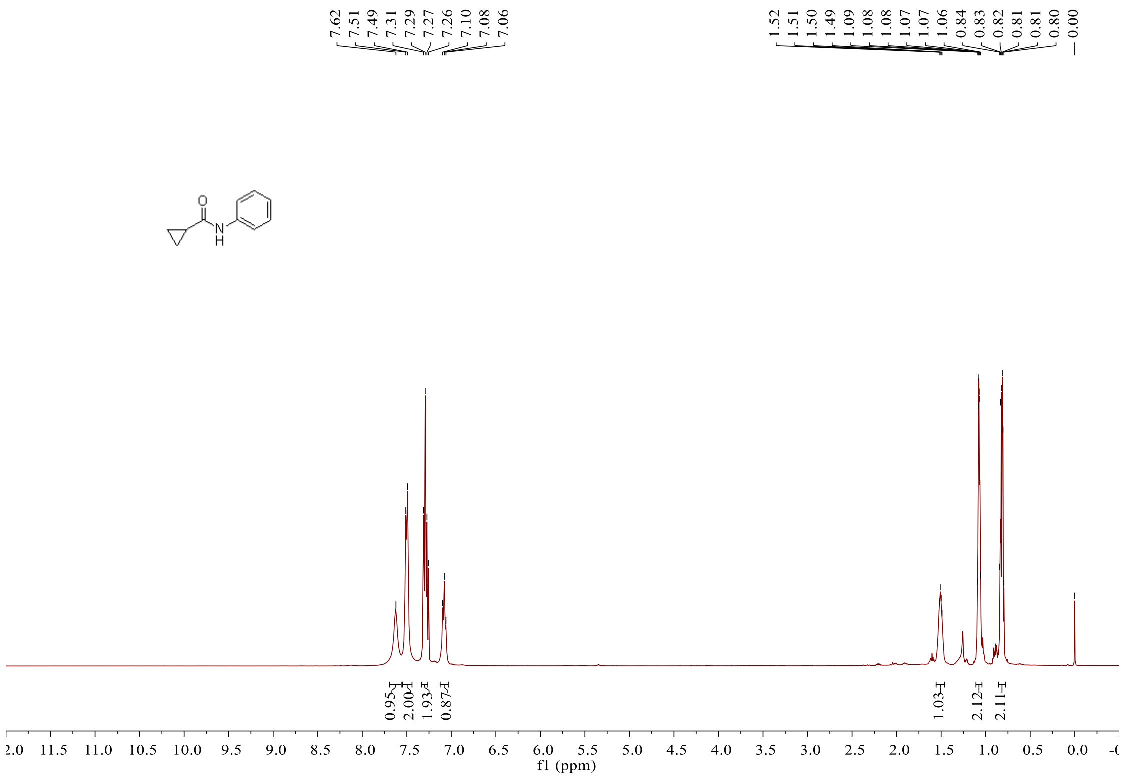

Supplementary Figure 29. ^{1}H NMR spectrum for compound **3l**

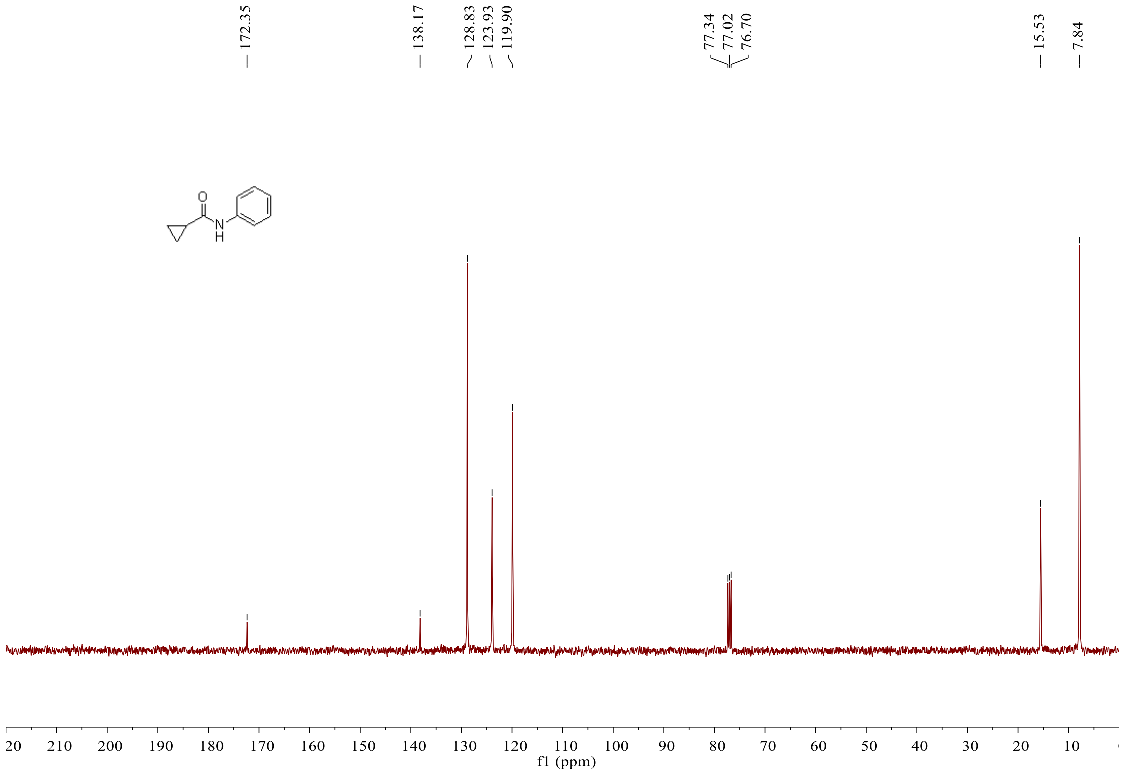

Supplementary Figure 30. ^{13}C NMR spectrum for compound **3l**

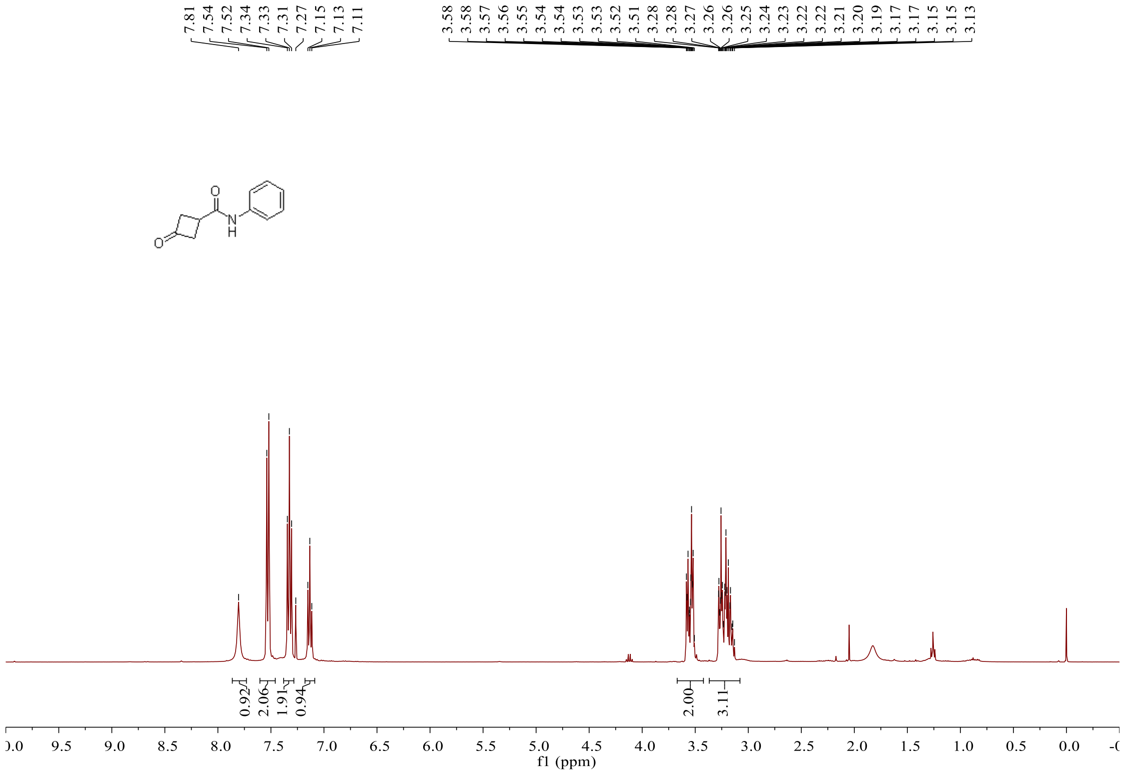

Supplementary Figure 31. ^{1}H NMR spectrum for compound **3m**

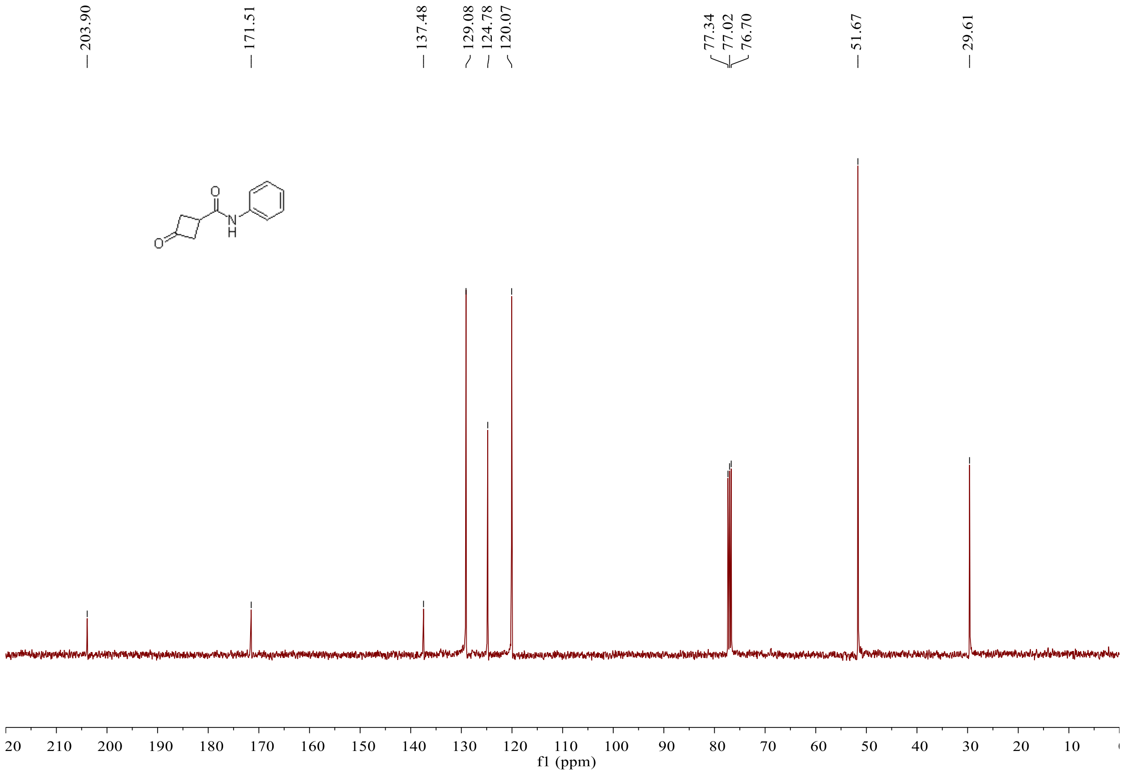

Supplementary Figure 32. ^{13}C NMR spectrum for compound **3m**

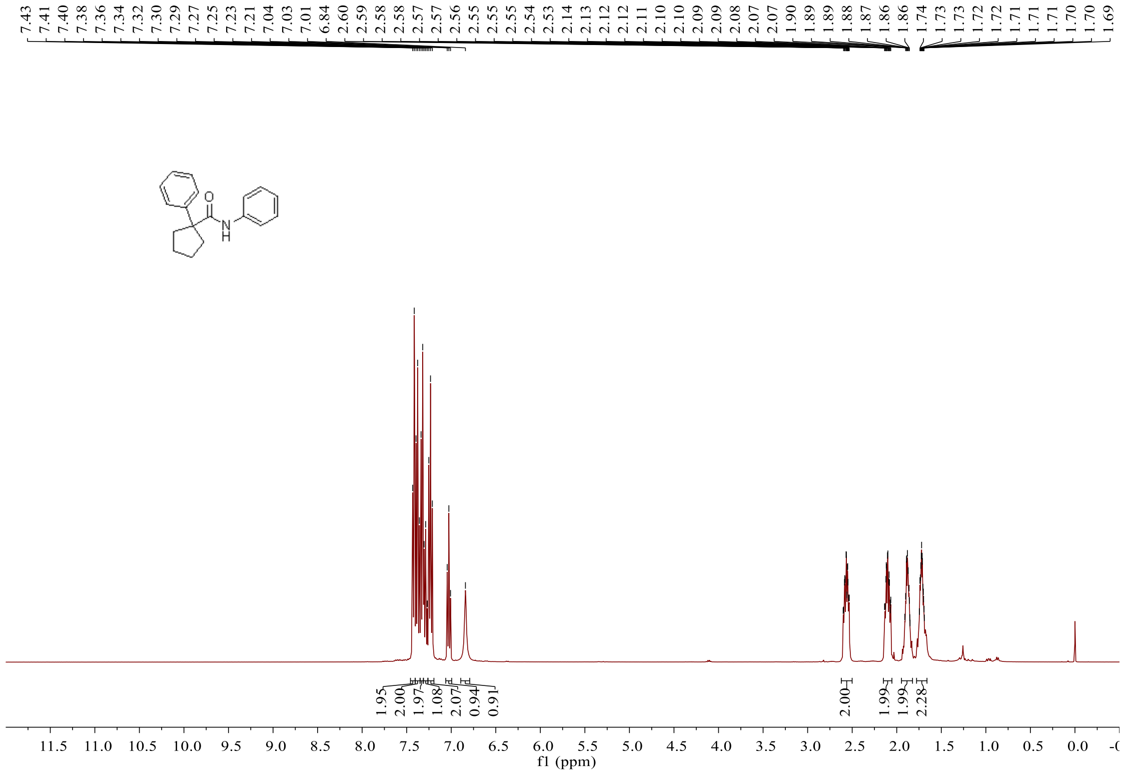

Supplementary Figure 33. ^{1}H NMR spectrum for compound **3n**

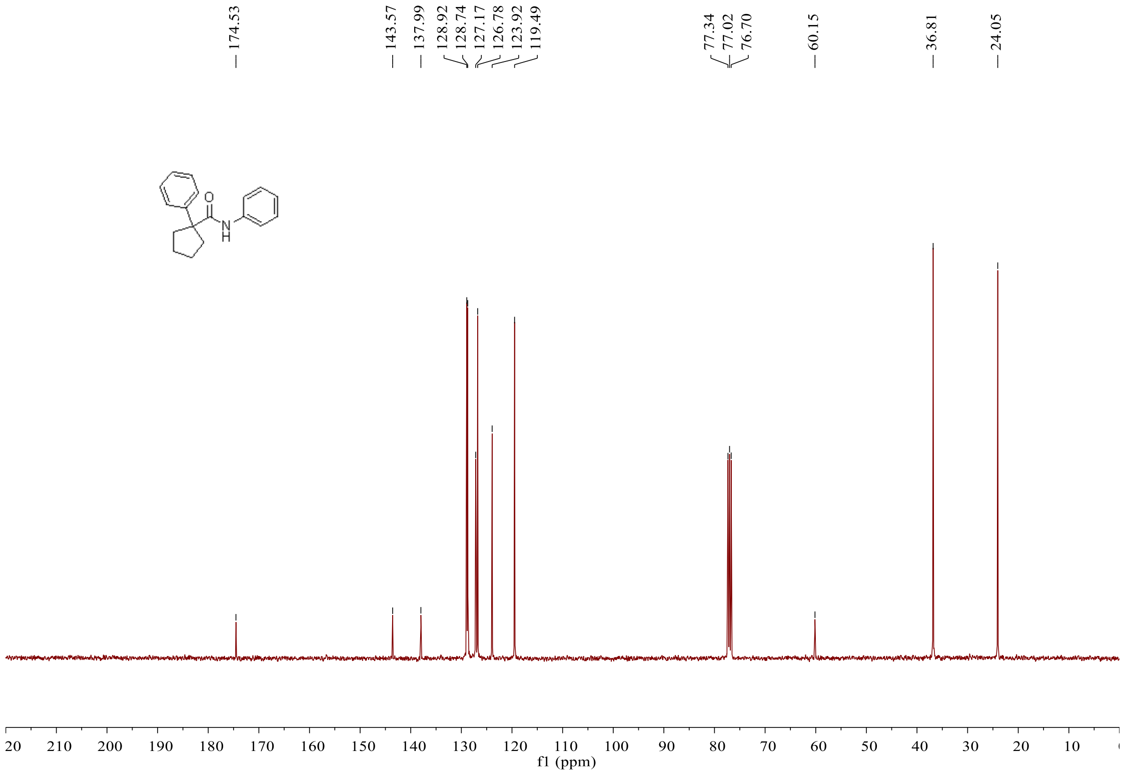

Supplementary Figure 34. ^{13}C NMR spectrum for compound **3n**

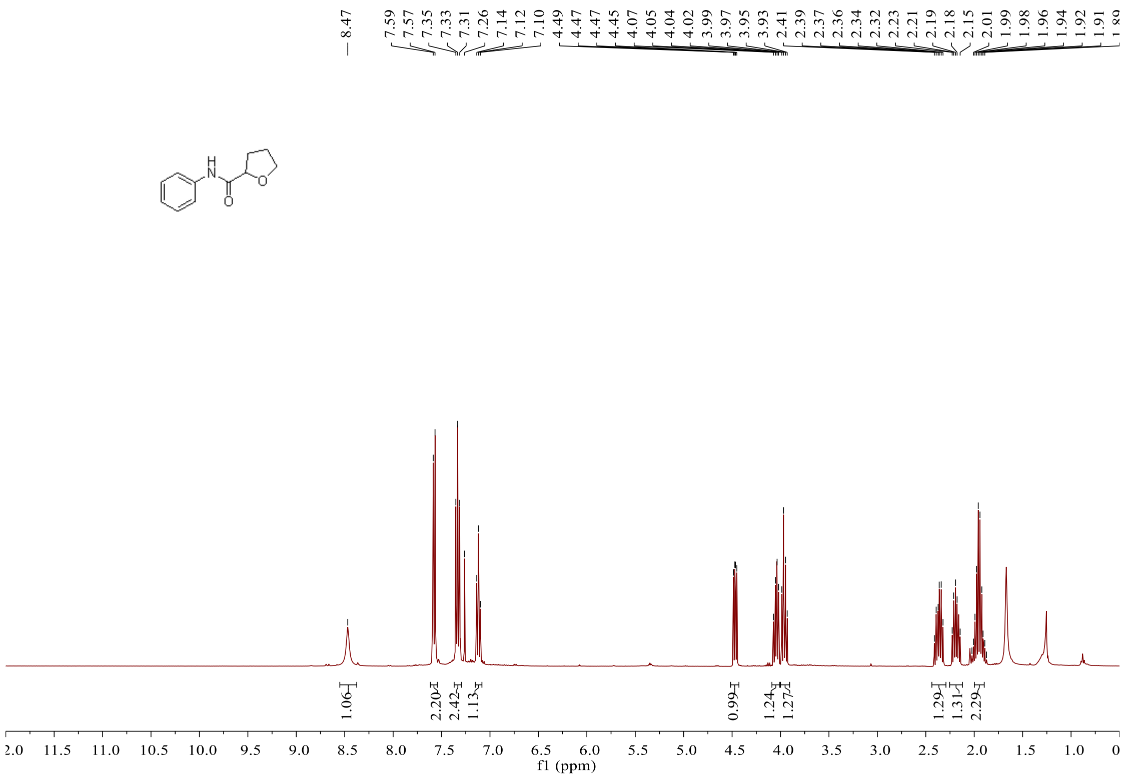

Supplementary Figure 35. ^{1}H NMR spectrum for compound **3o**

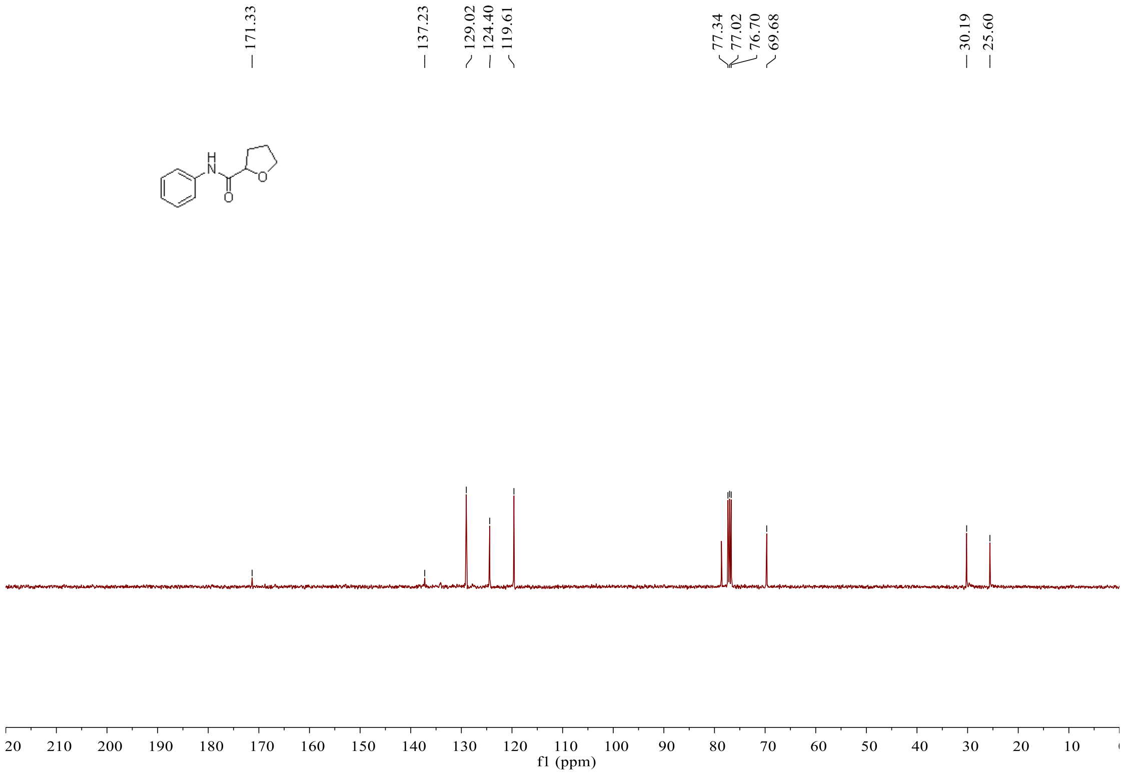

Supplementary Figure 36. ^{13}C NMR spectrum for compound **3o**

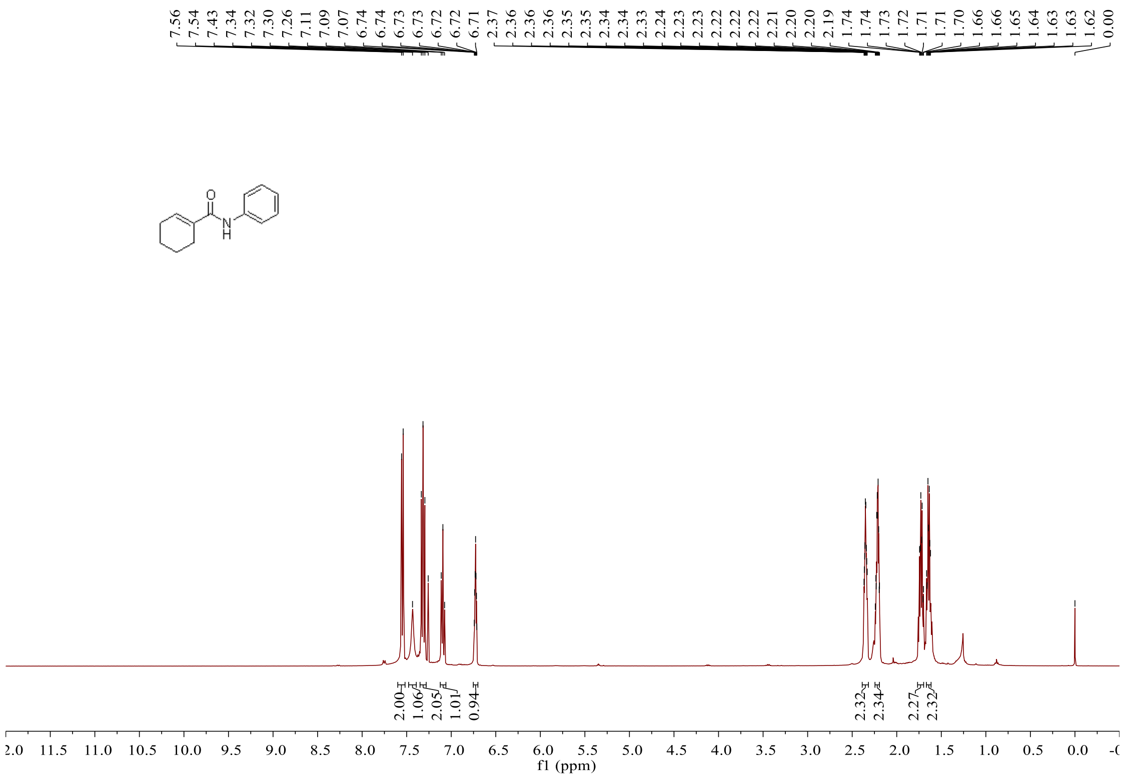

Supplementary Figure 37. ^{1}H NMR spectrum for compound **3p**

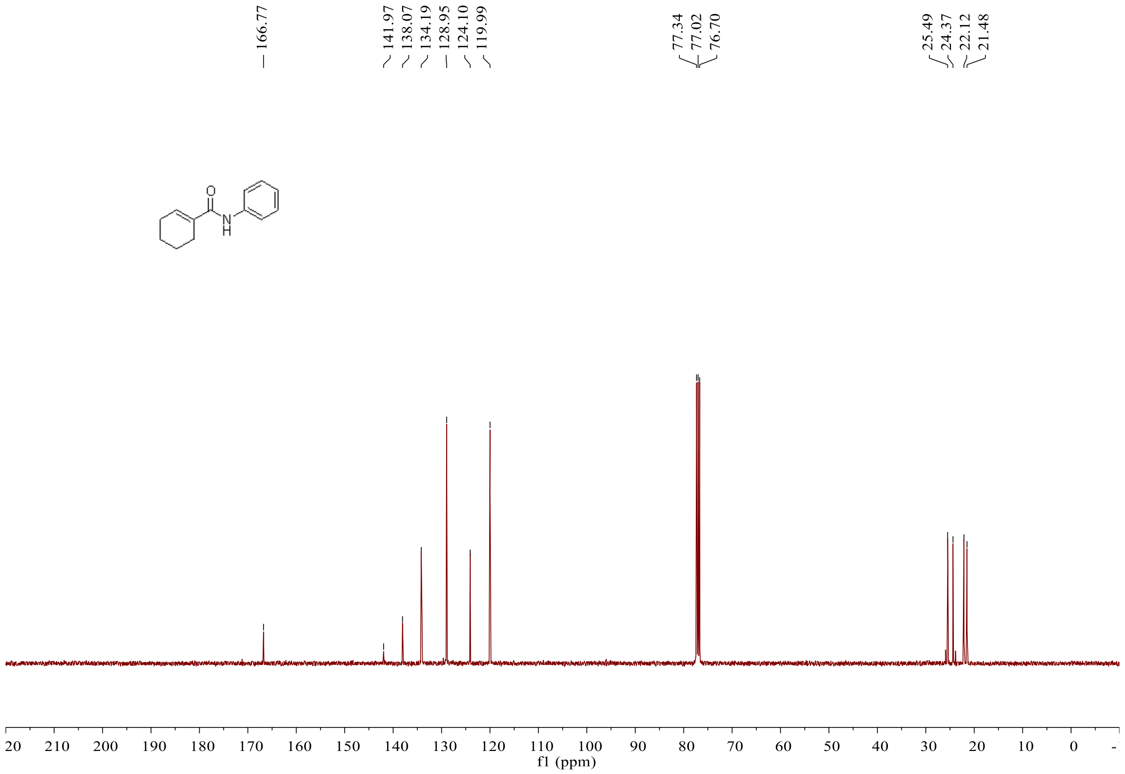

Supplementary Figure 38. ^{13}C NMR spectrum for compound **3p**

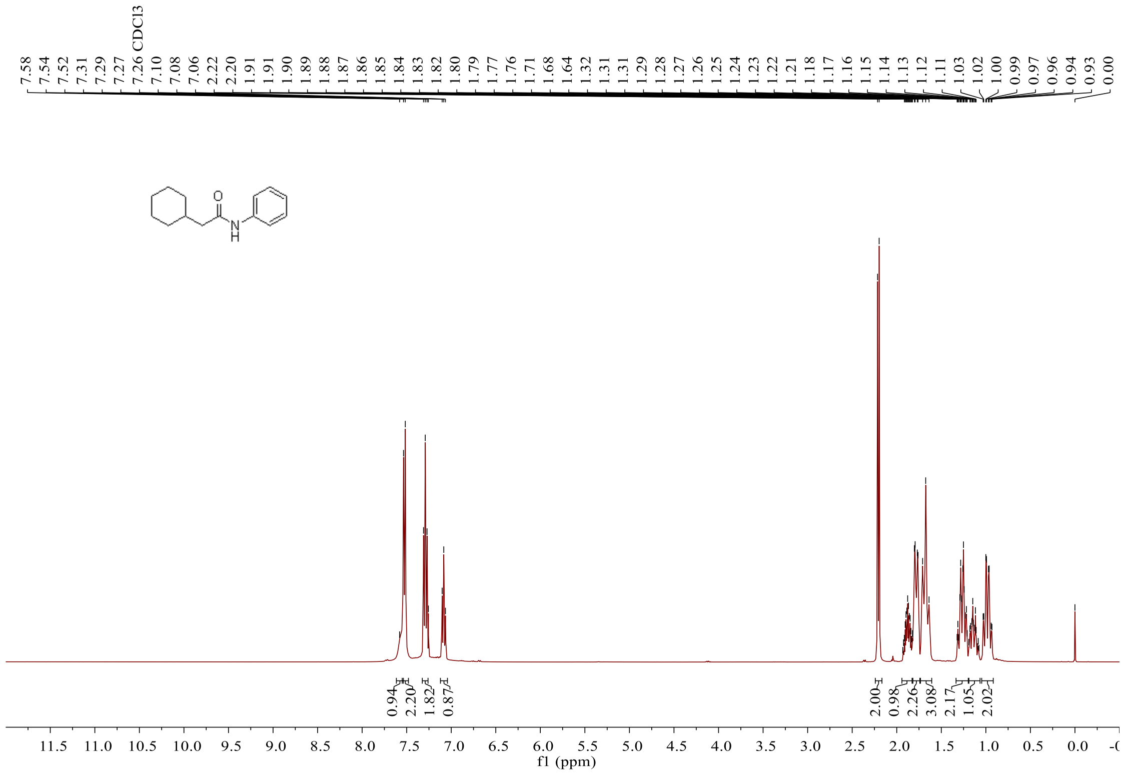

Supplementary Figure 39. ^{1}H NMR spectrum for compound **3q**

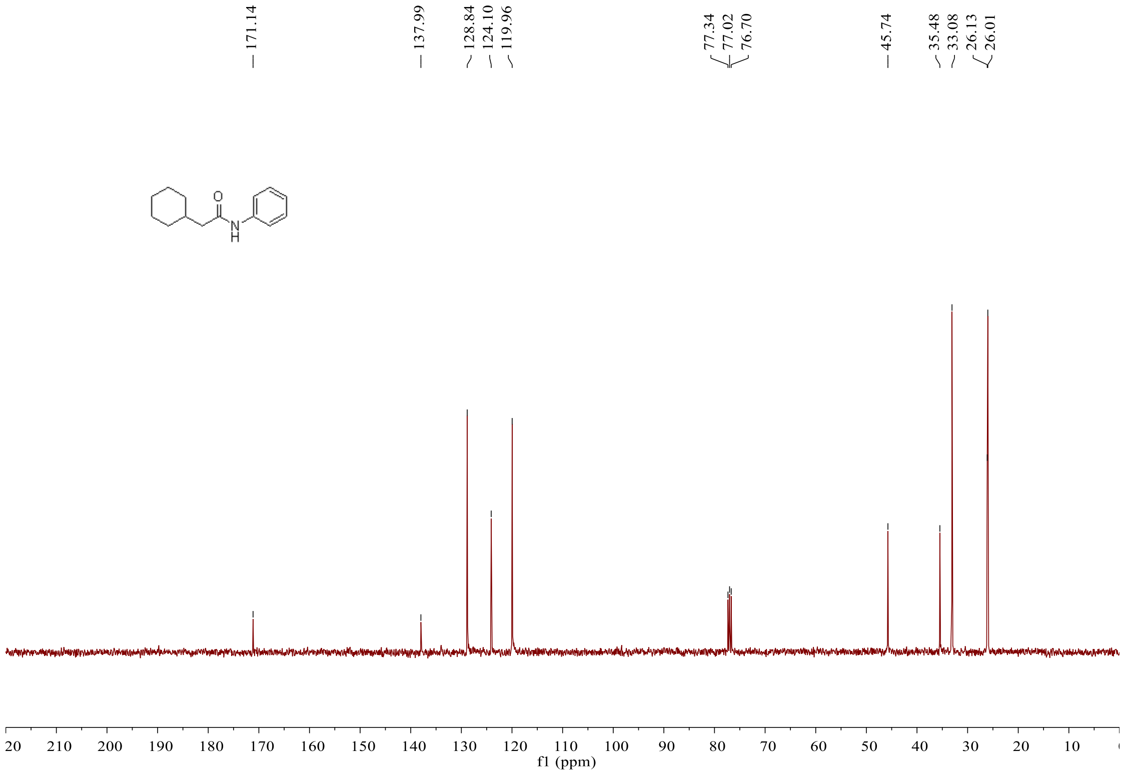

Supplementary Figure 40. ^{13}C NMR spectrum for compound **3q**

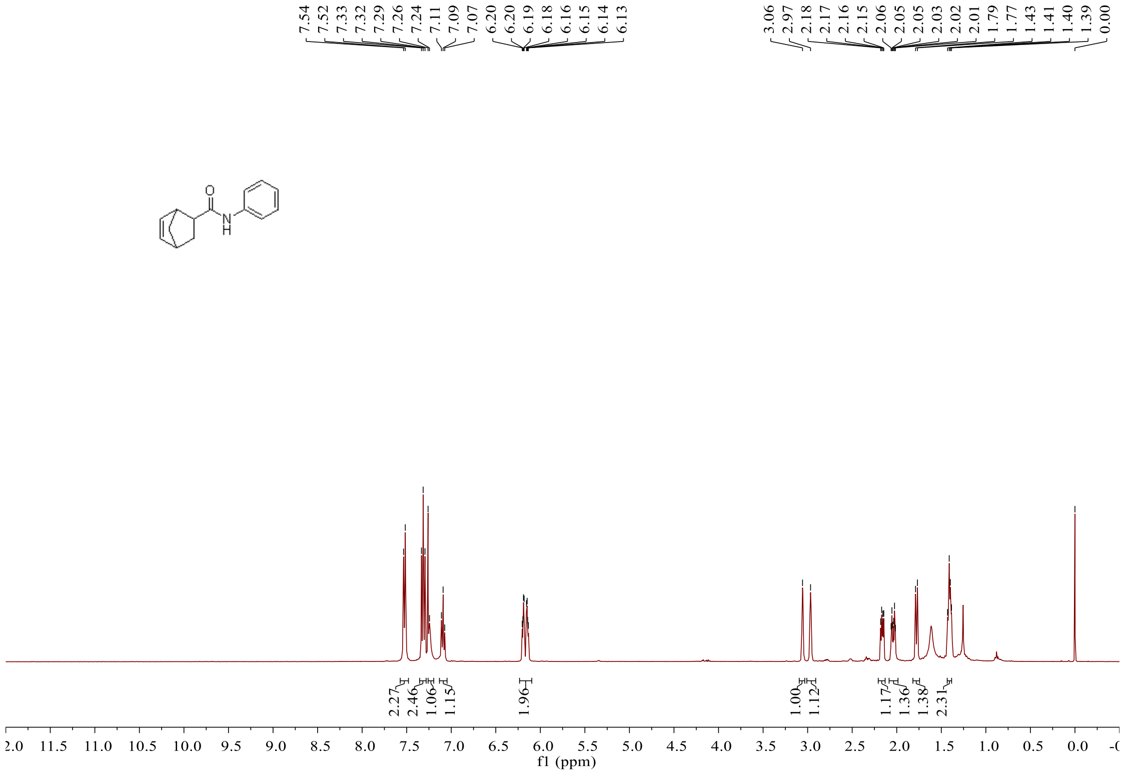

Supplementary Figure 41. ^{1}H NMR spectrum for compound **3r**

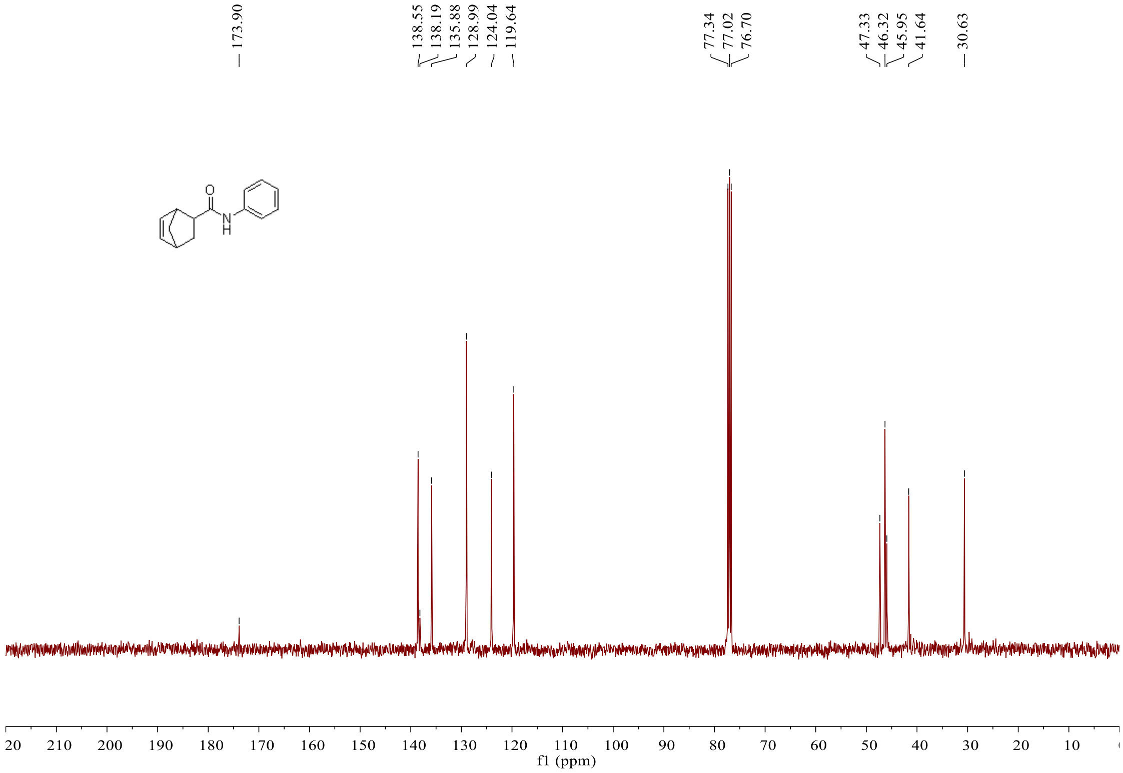

Supplementary Figure 42. ^{13}C NMR spectrum for compound **3r**

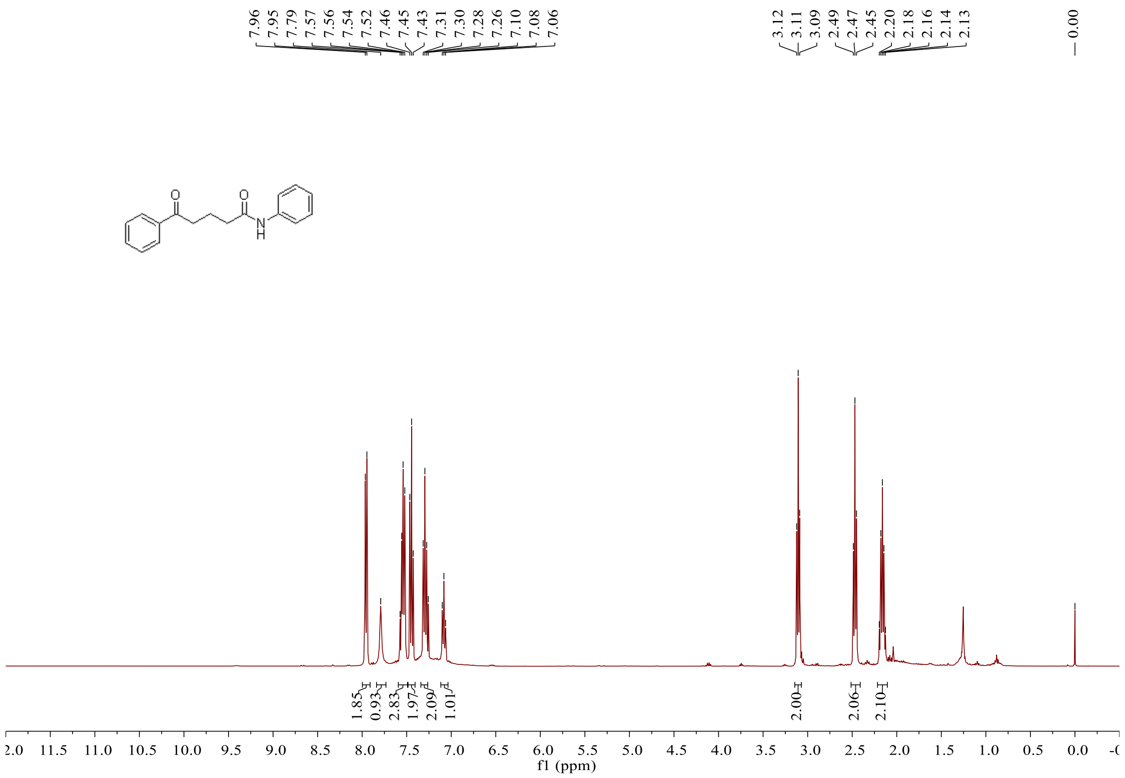

Supplementary Figure 43. ^{1}H NMR spectrum for compound **3s**

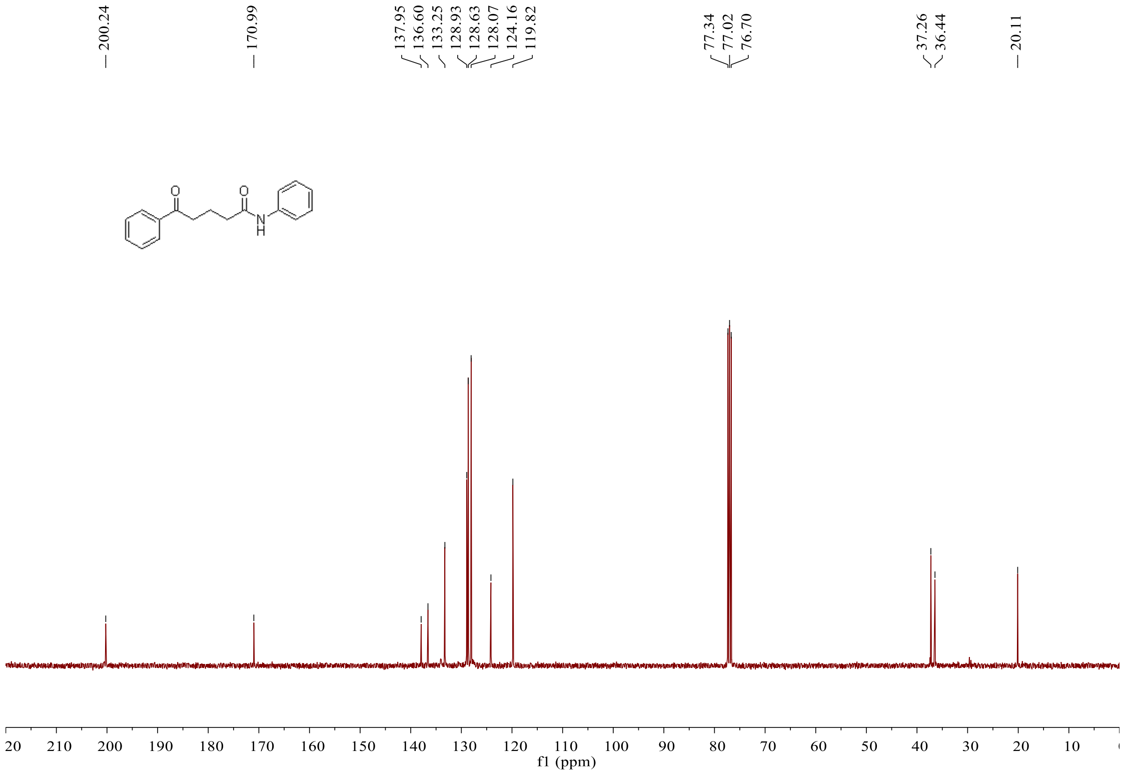

Supplementary Figure 44. ^{13}C NMR spectrum for compound **3s**

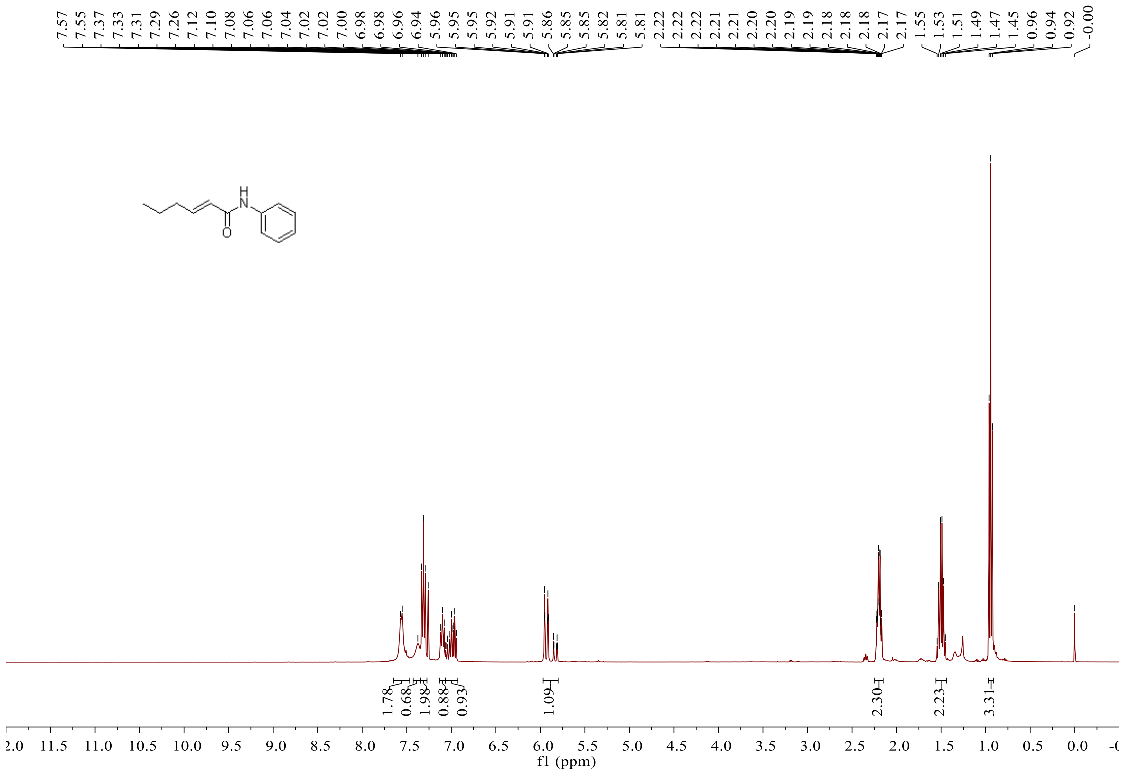

Supplementary Figure 45. ^{1}H NMR spectrum for compound **3t**

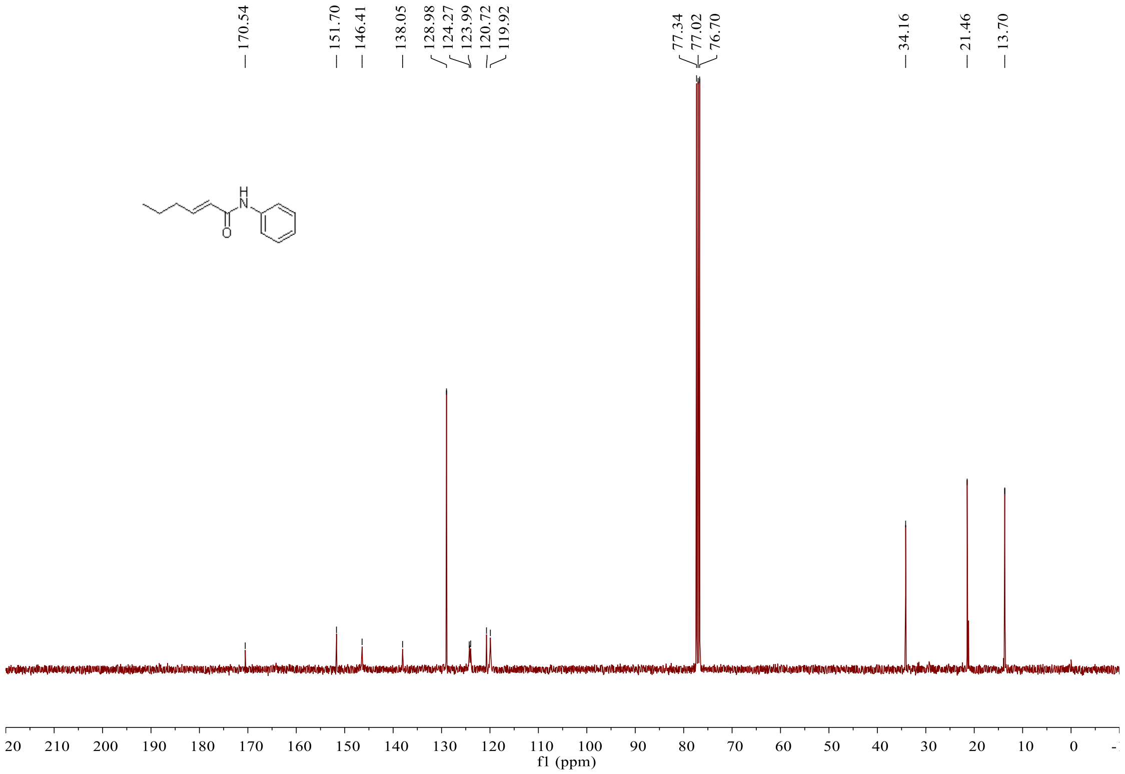

Supplementary Figure 46. ^{13}C NMR spectrum for compound **3t**

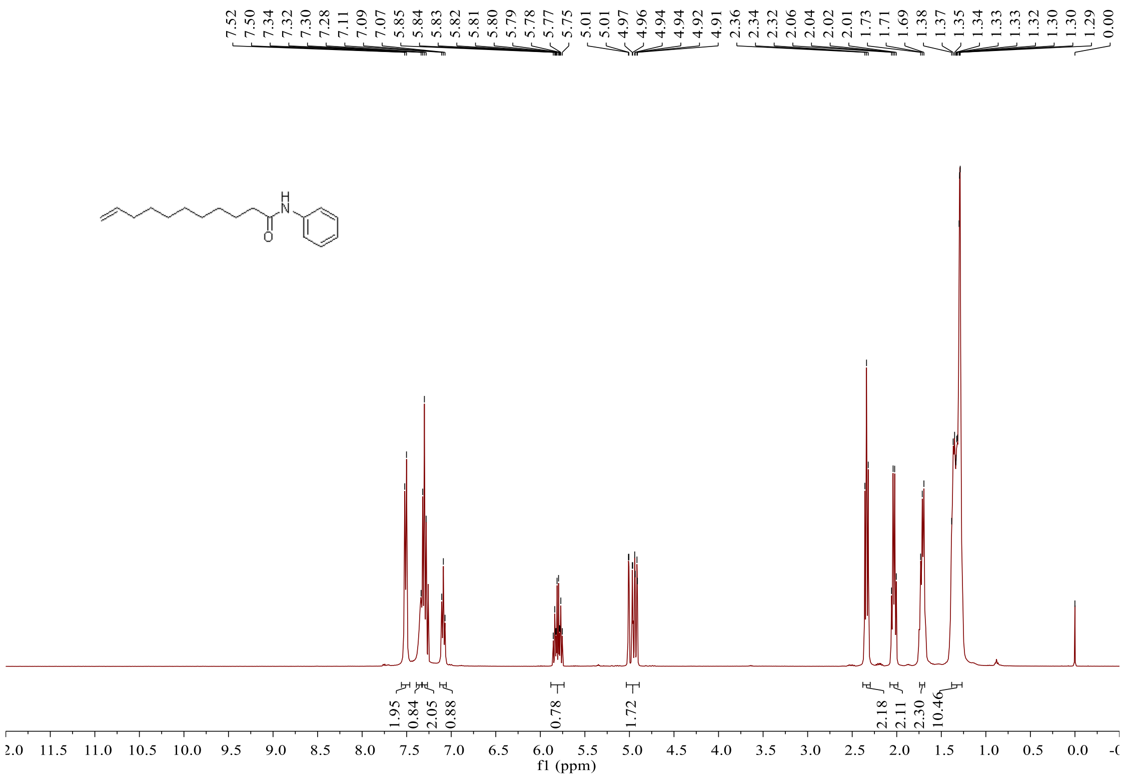

Supplementary Figure 47. ^{1}H NMR spectrum for compound **3u**

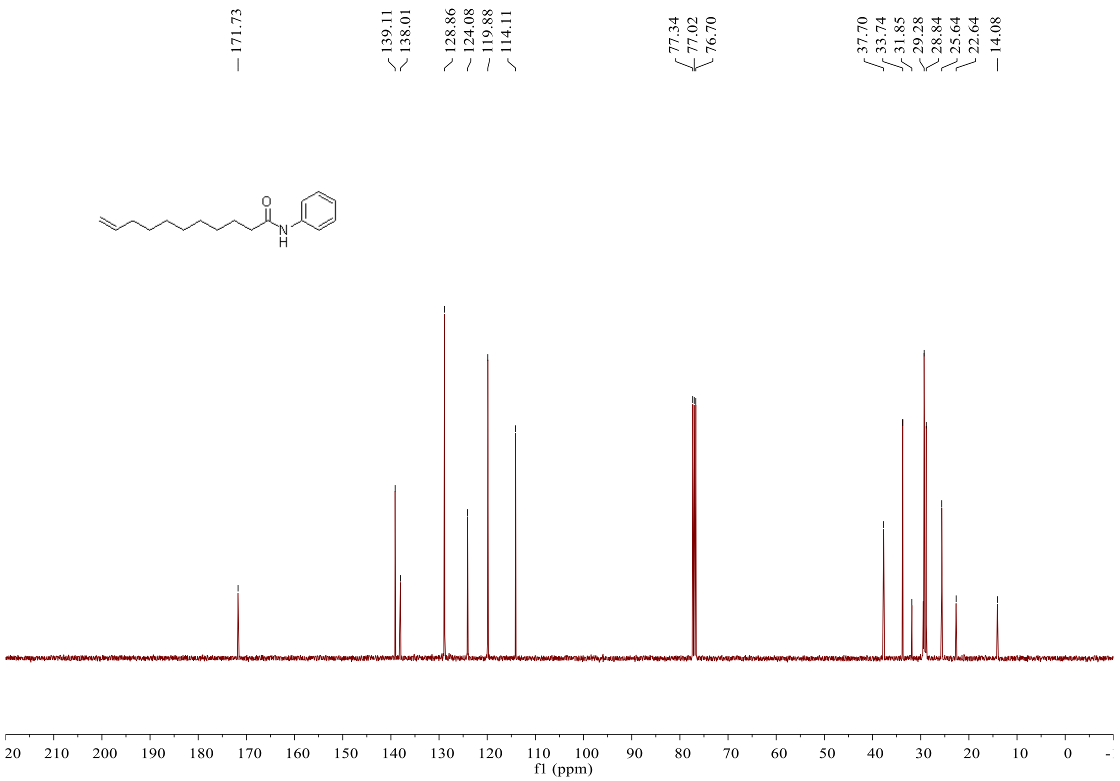

Supplementary Figure 48. ^{13}C NMR spectrum for compound **3u**

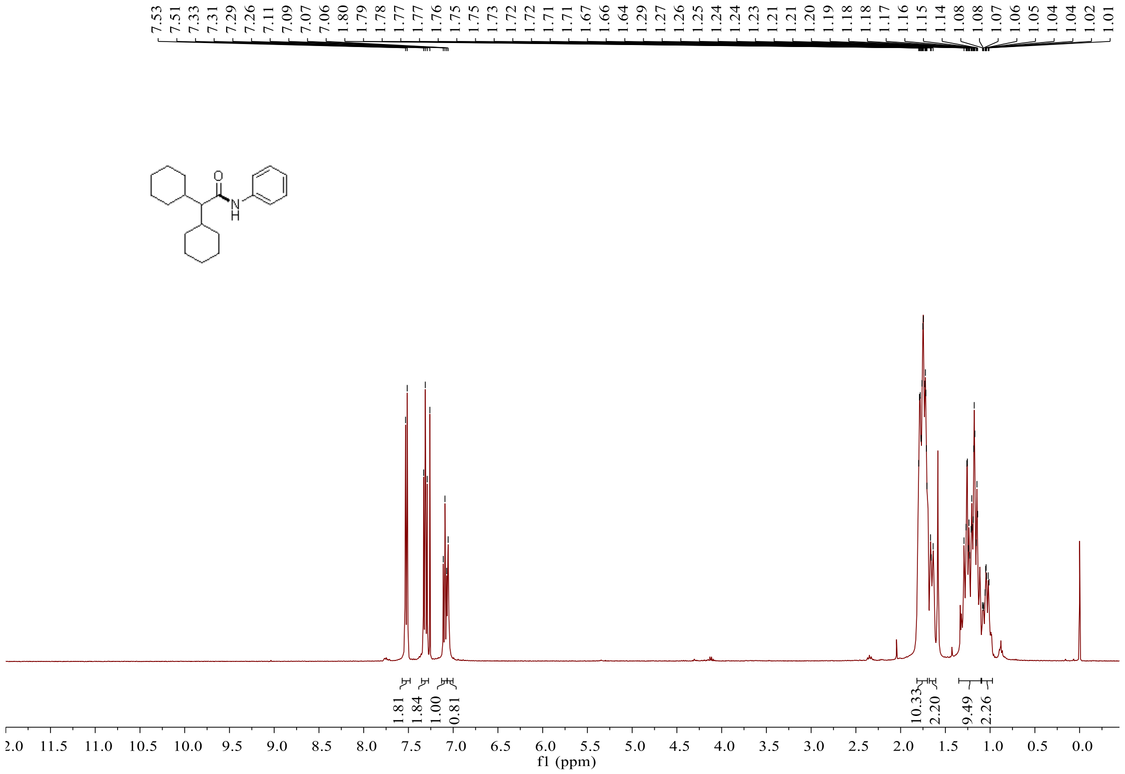

Supplementary Figure 49. ^{1}H NMR spectrum for compound **3v**

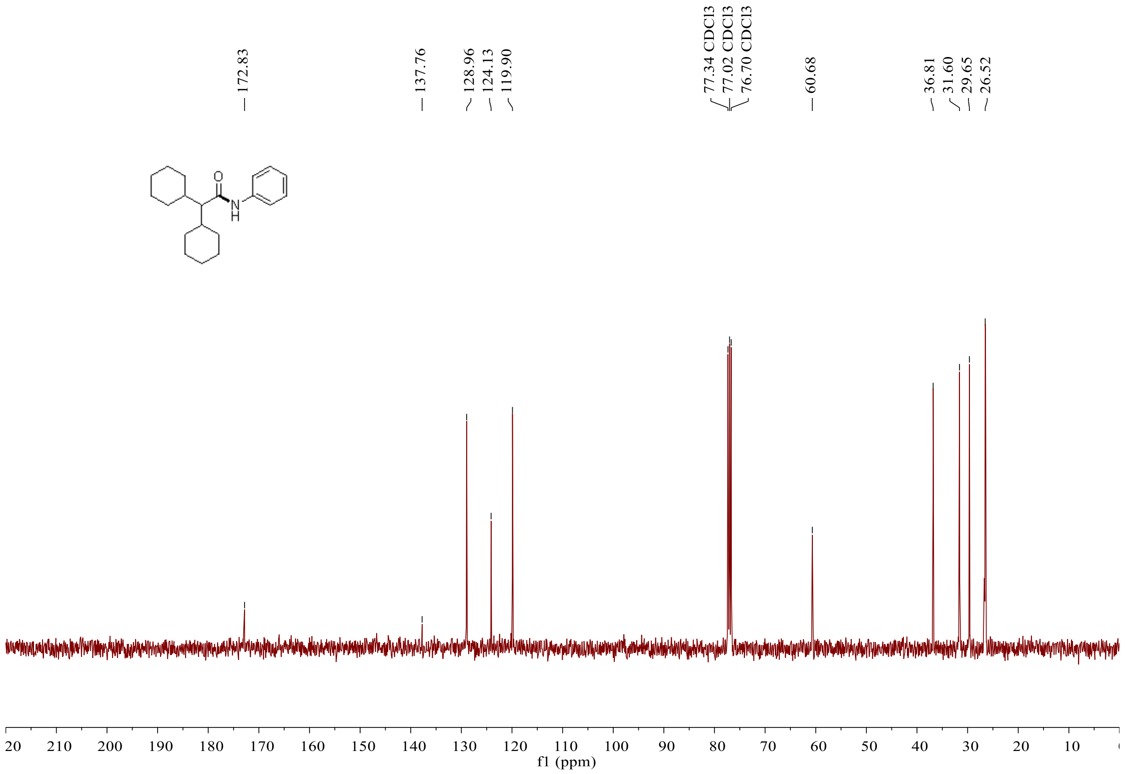

Supplementary Figure 50. ^{13}C NMR spectrum for compound **3v**

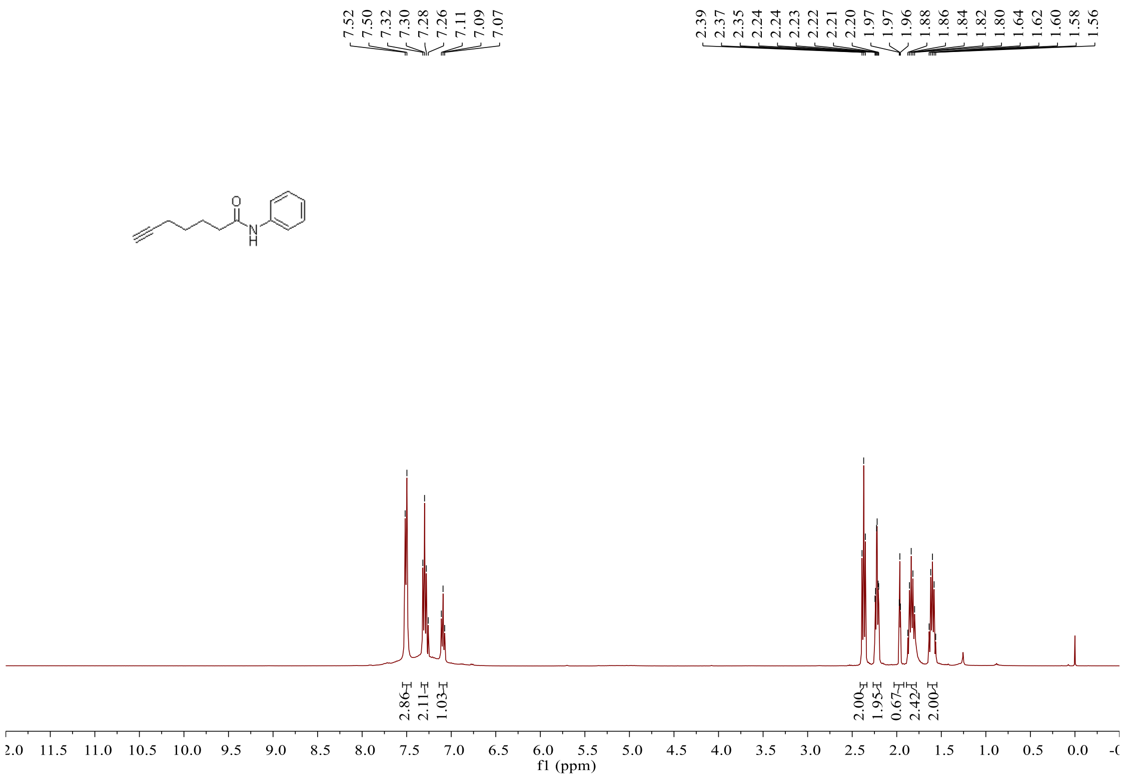

Supplementary Figure 51. ^{1}H NMR spectrum for compound **3w**

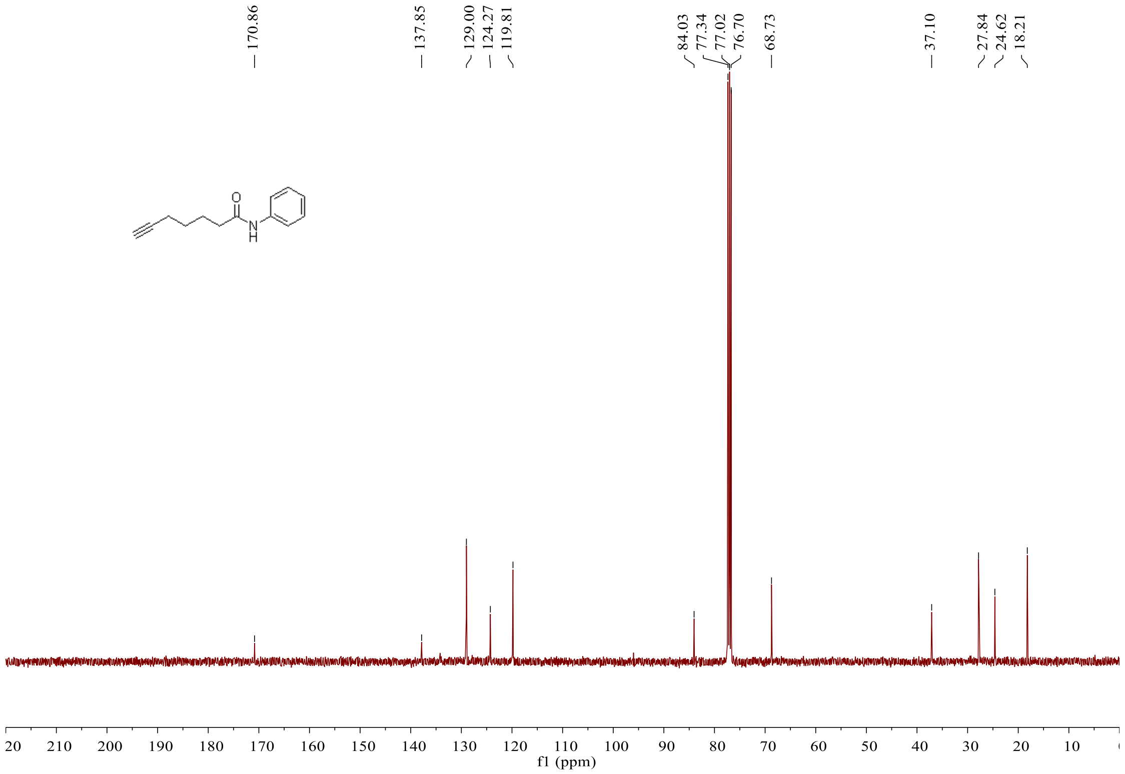

Supplementary Figure 52. ^{13}C NMR spectrum for compound **3w**

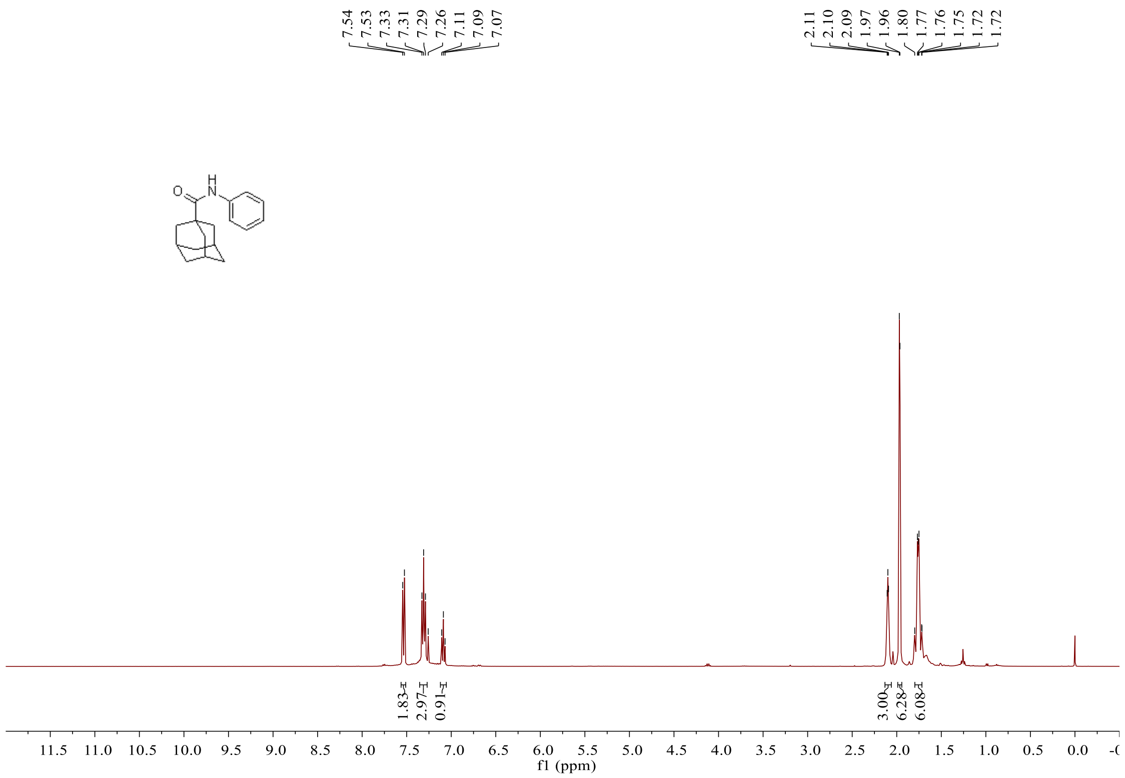

Supplementary Figure 53. ^{1}H NMR spectrum for compound **3x**

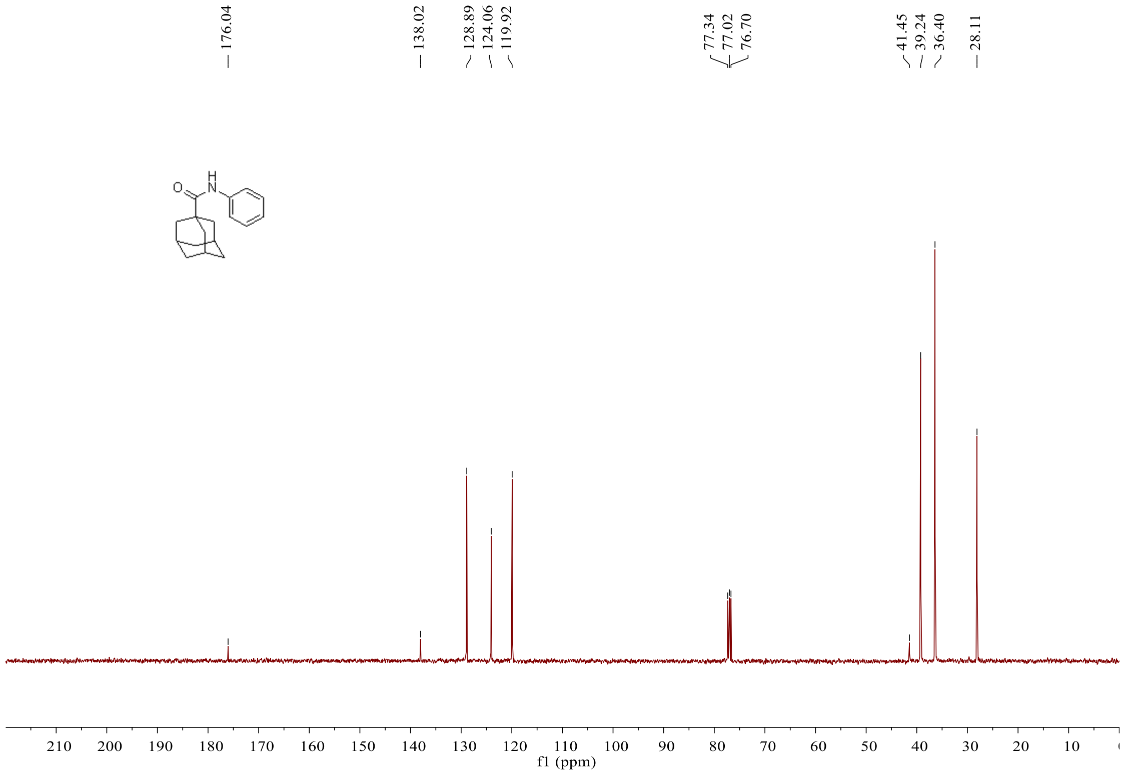

Supplementary Figure 54. ^{13}C NMR spectrum for compound **3x**

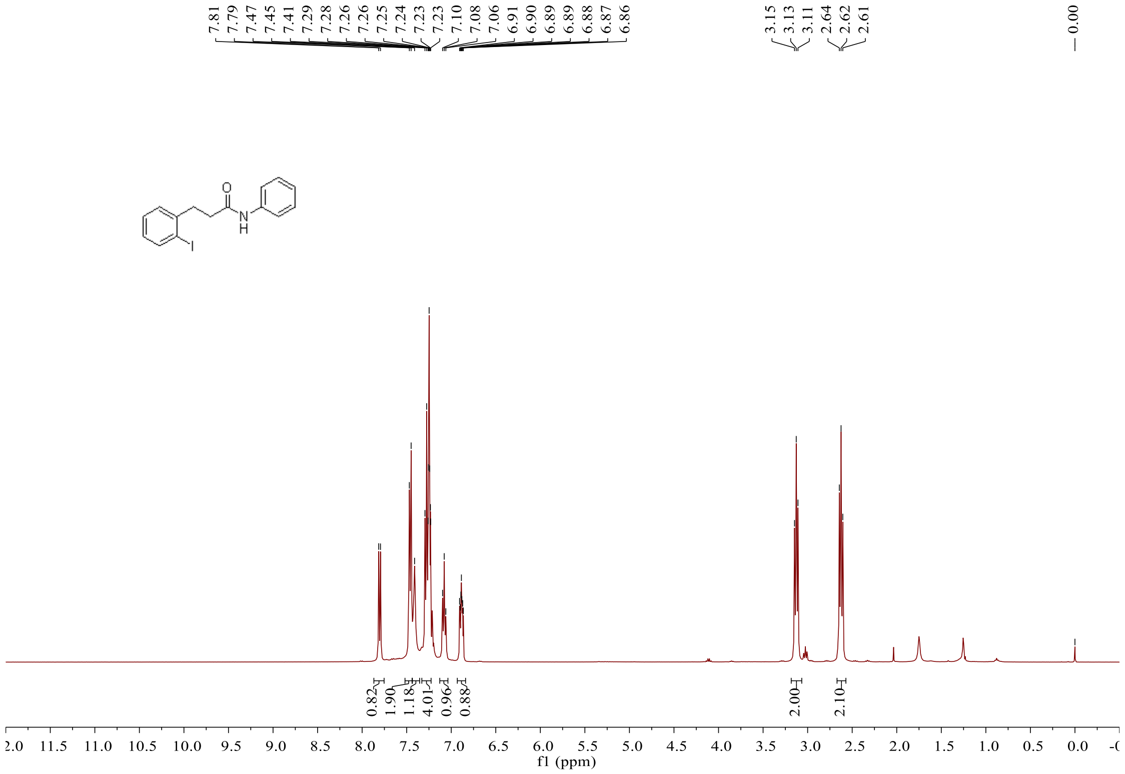

Supplementary Figure 55. ^{1}H NMR spectrum for compound **3y**

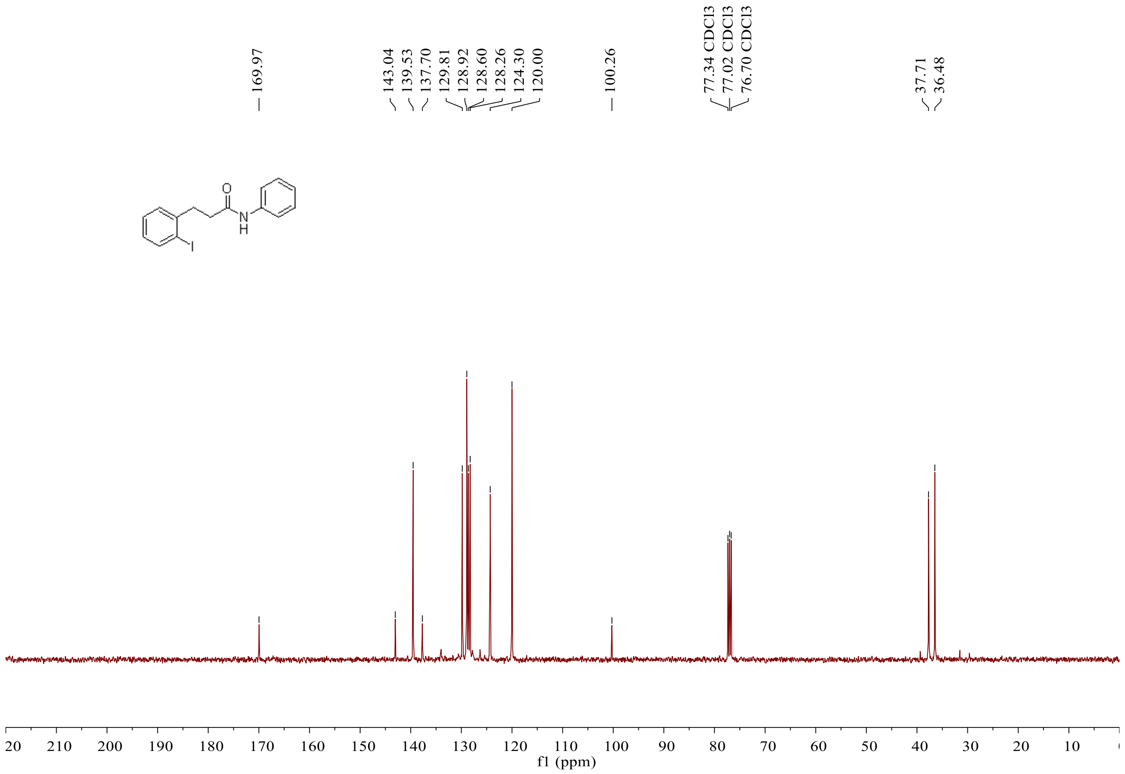

Supplementary Figure 56. ^{13}C NMR spectrum for compound **3y**

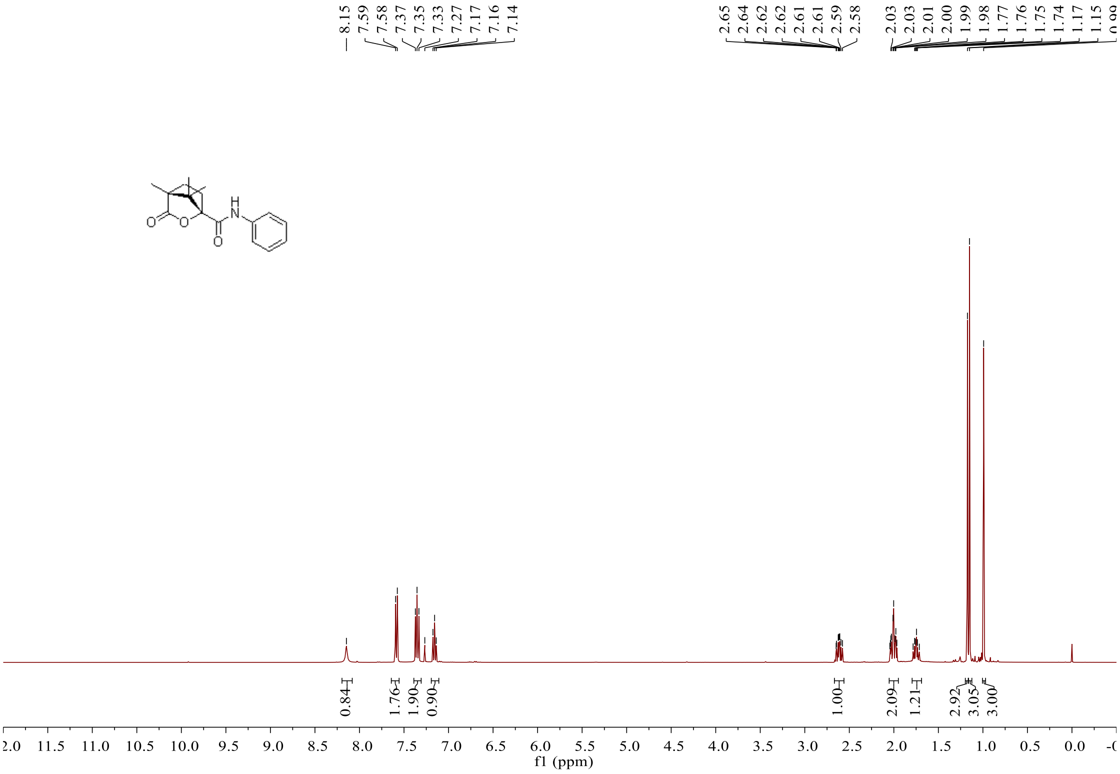

Supplementary Figure 57. ^{1}H NMR spectrum for compound **3z**

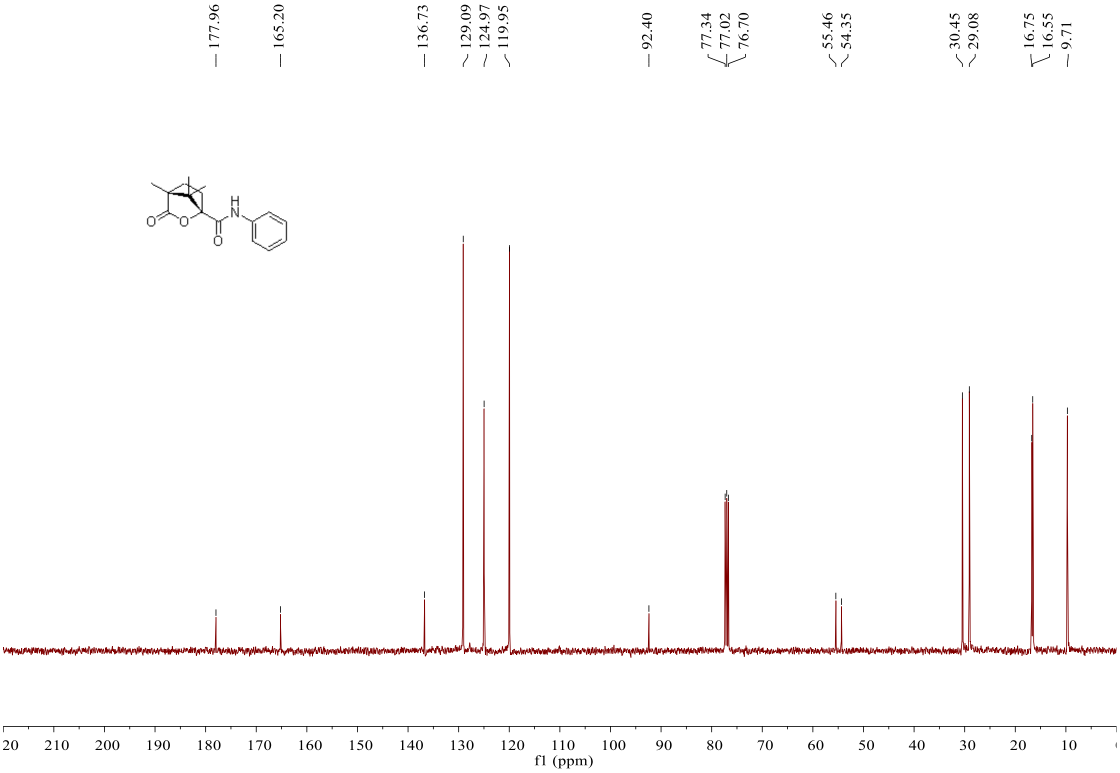

Supplementary Figure 58. ^{13}C NMR spectrum for compound **3z**

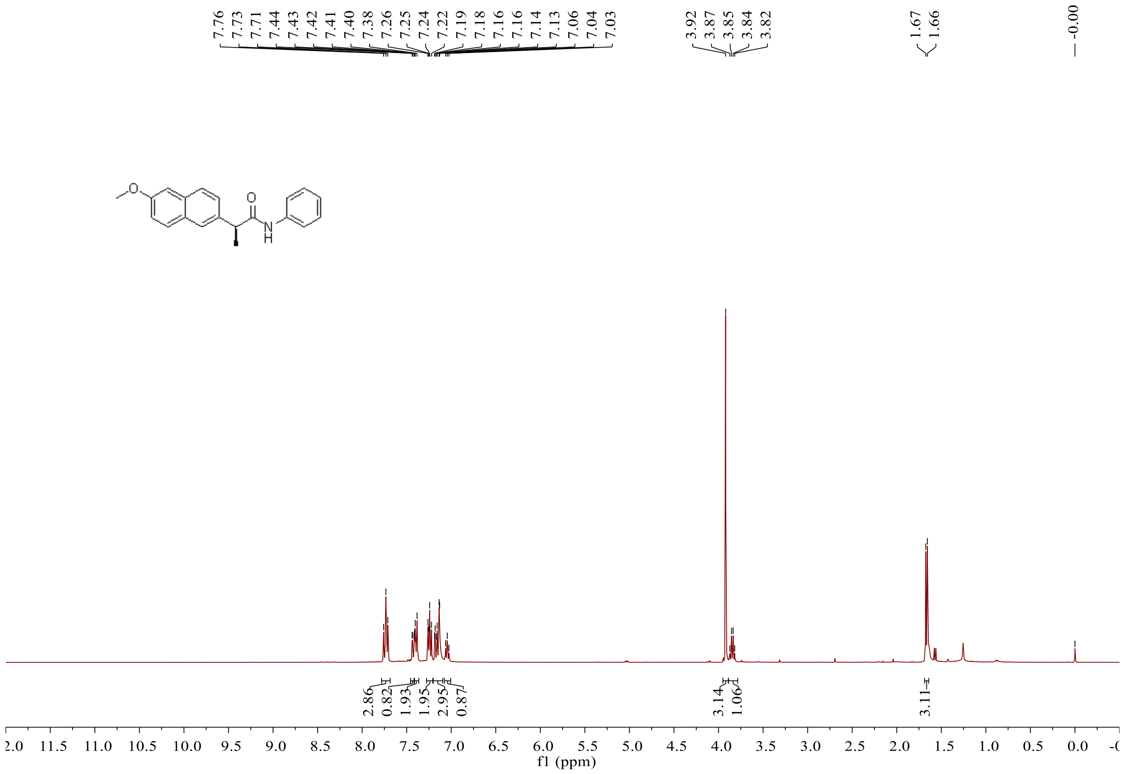

Supplementary Figure 59. ^{1}H NMR spectrum for compound **3aa**

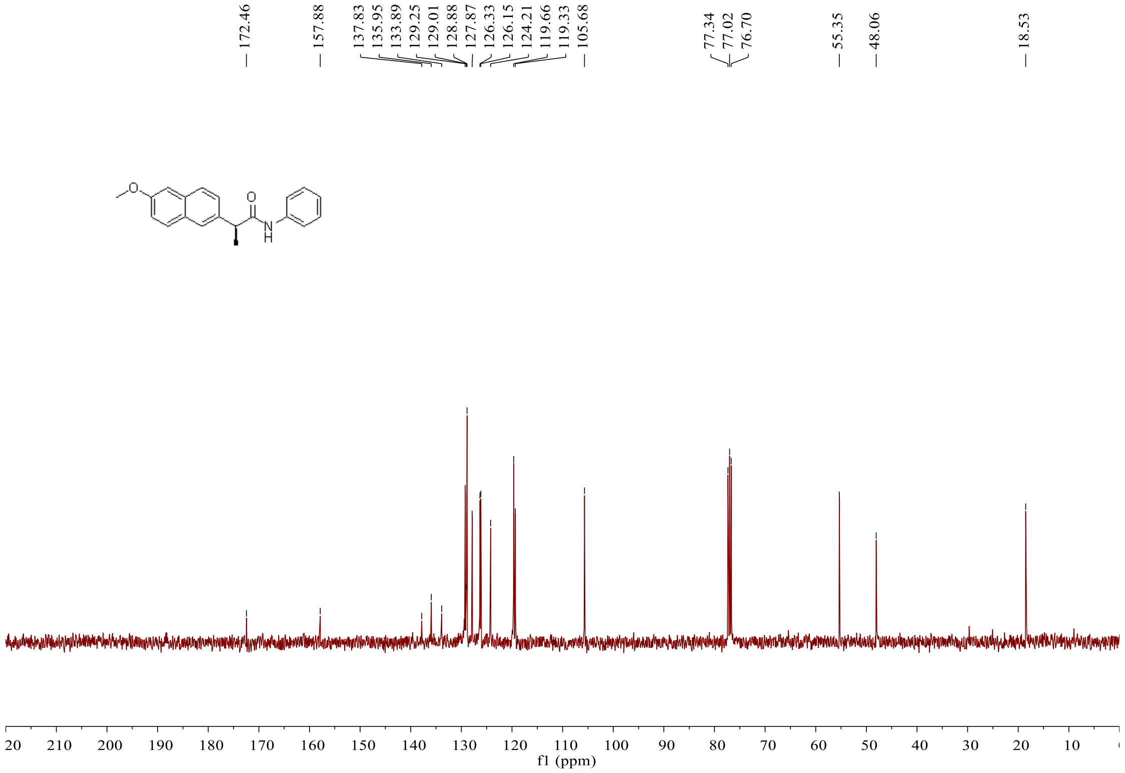

Supplementary Figure 60. ^{13}C NMR spectrum for compound **3aa**

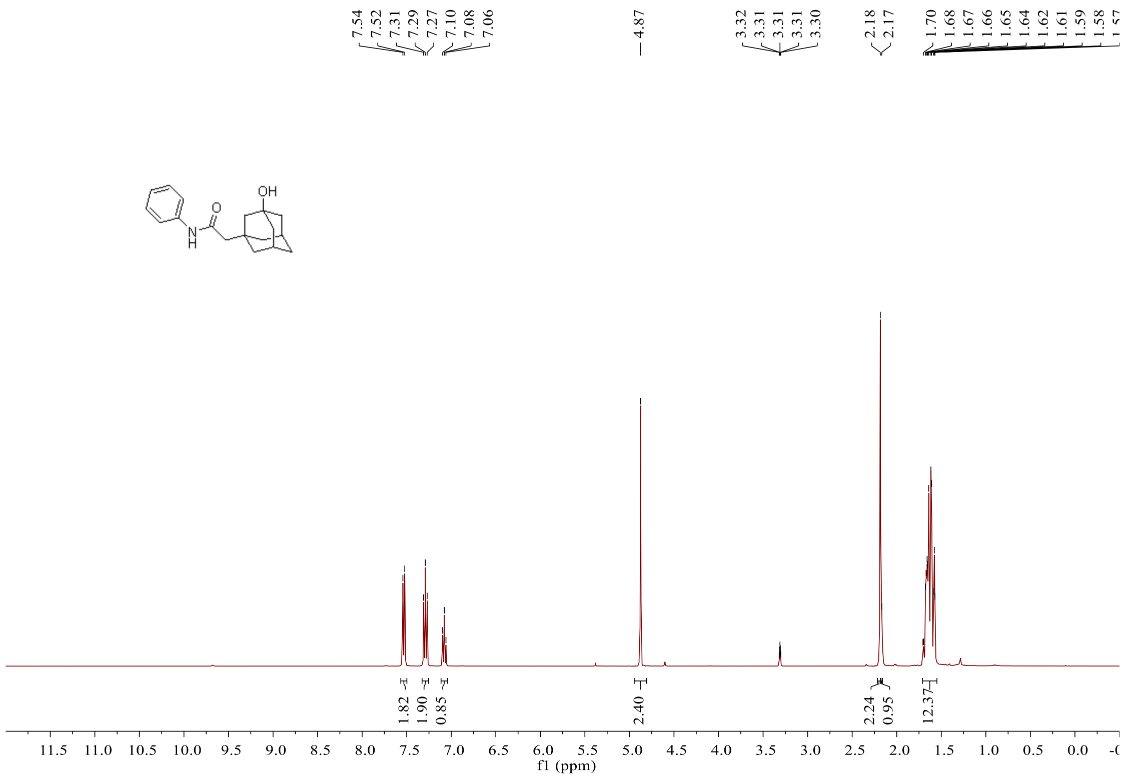

Supplementary Figure 61. ^{1}H NMR spectrum for compound **3bb**

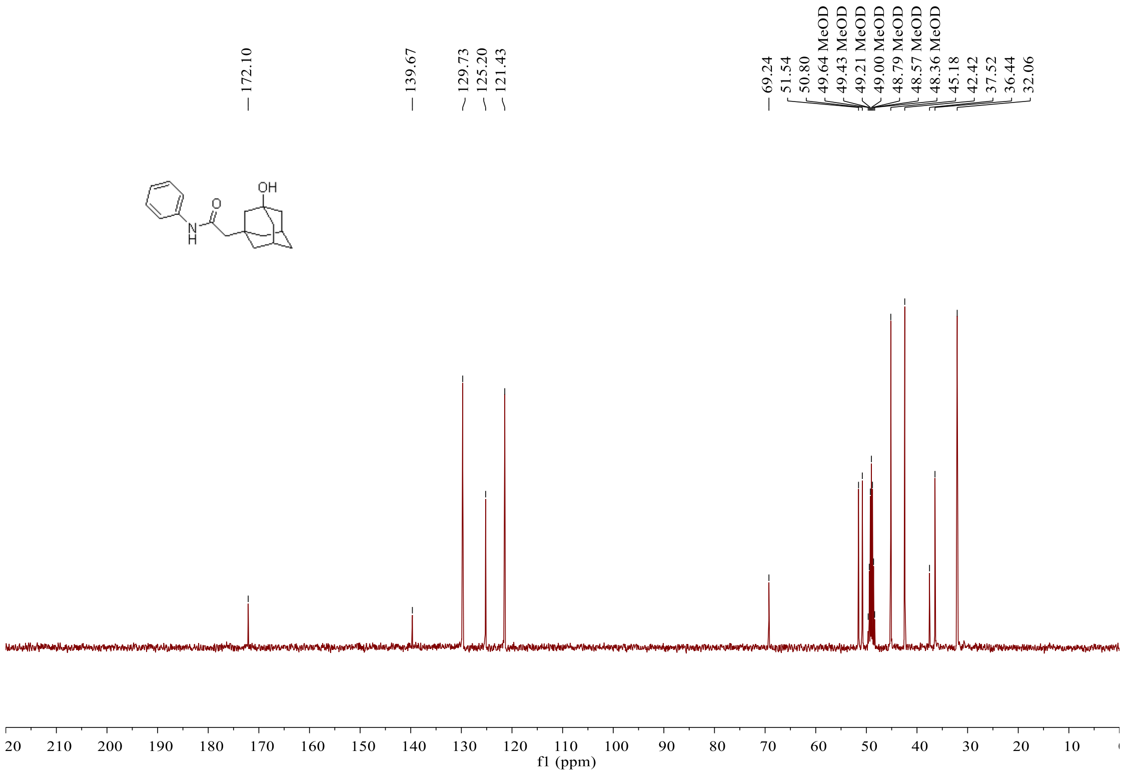

Supplementary Figure 62. ^{13}C NMR spectrum for compound **3bb**

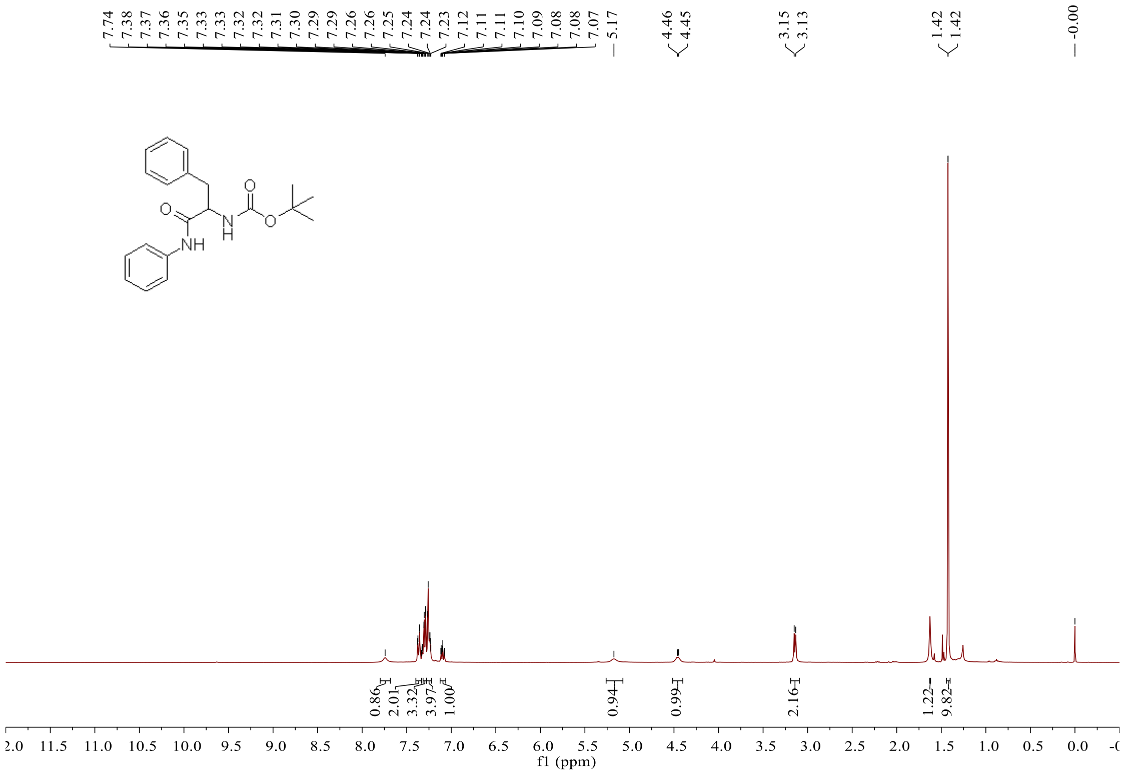

Supplementary Figure 63. ^{1}H NMR spectrum for compound **3cc**

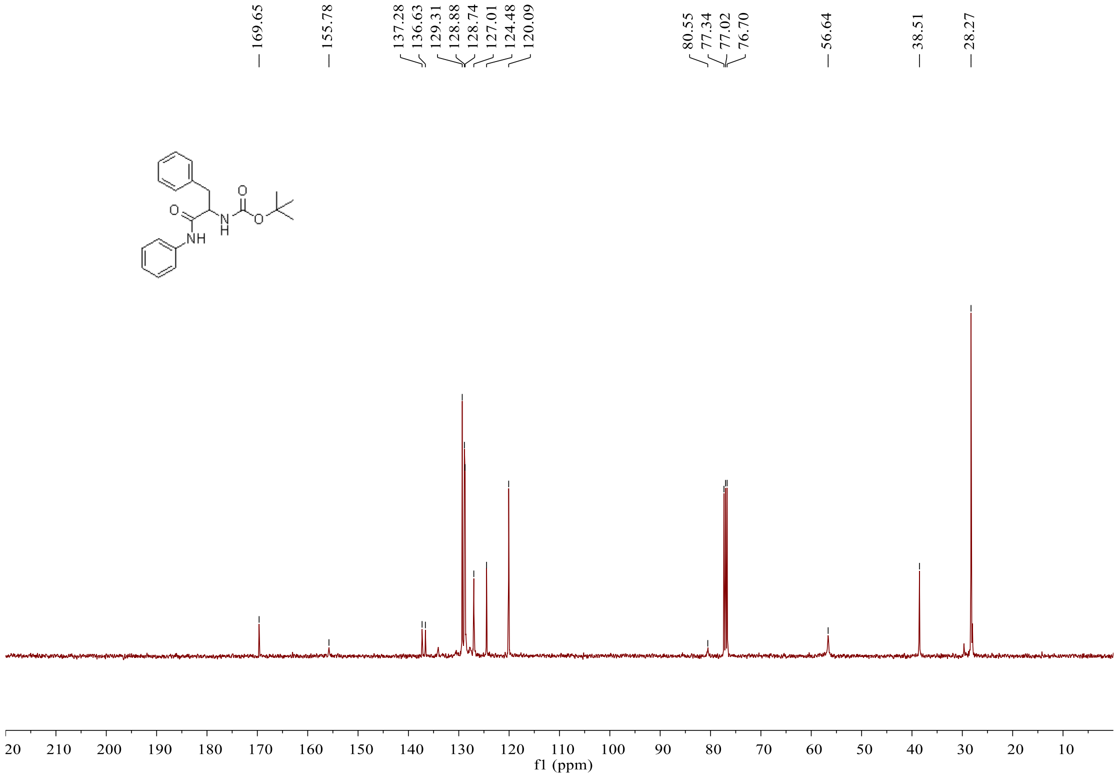

Supplementary Figure 64. ^{13}C NMR spectrum for compound **3cc**

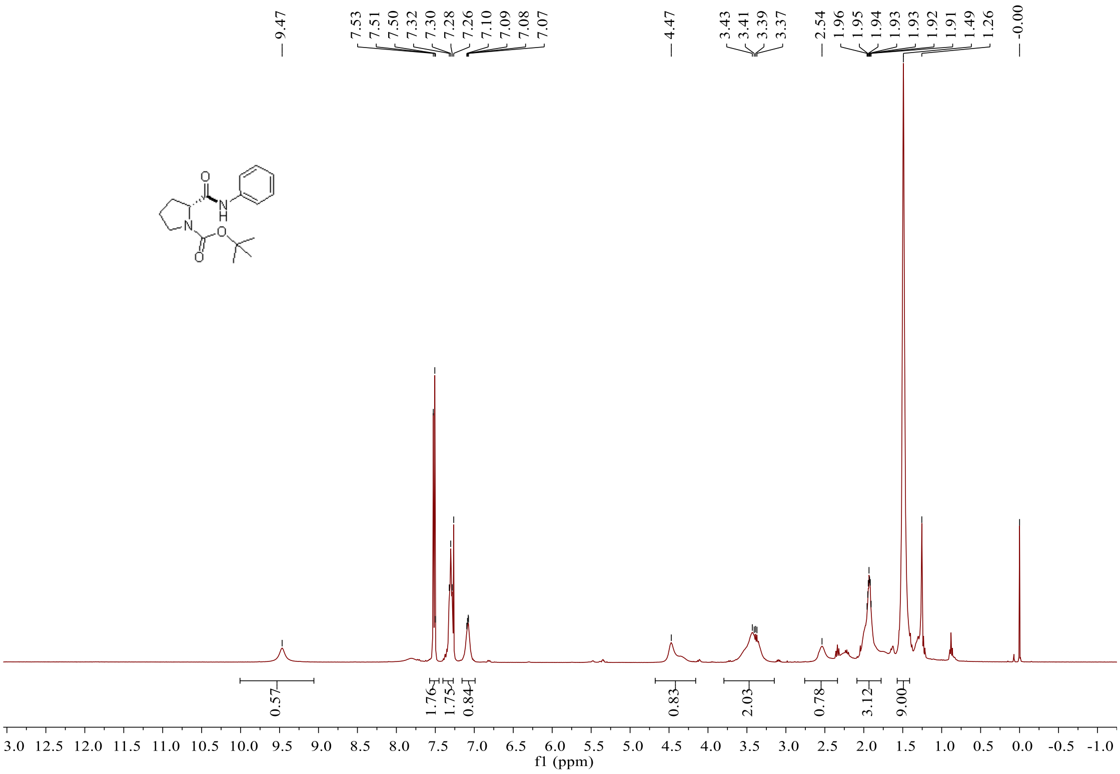

Supplementary Figure 65. ^{1}H NMR spectrum for compound **3dd**

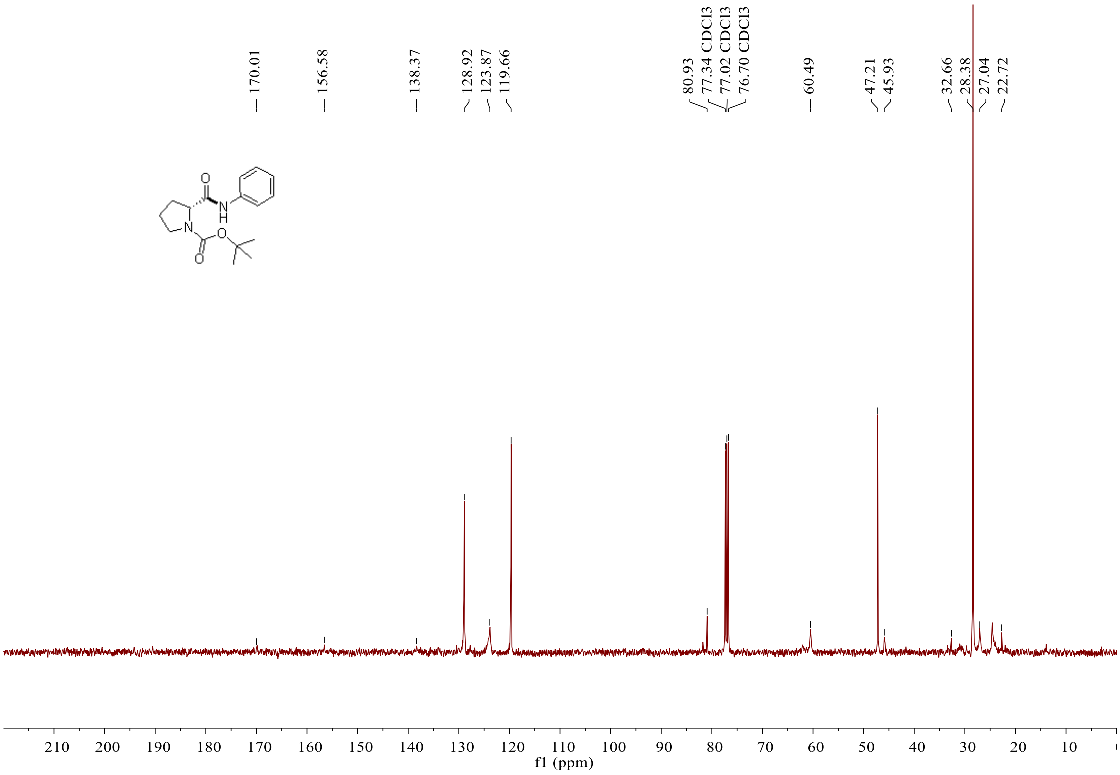

Supplementary Figure 66. ^{13}C NMR spectrum for compound **3dd**

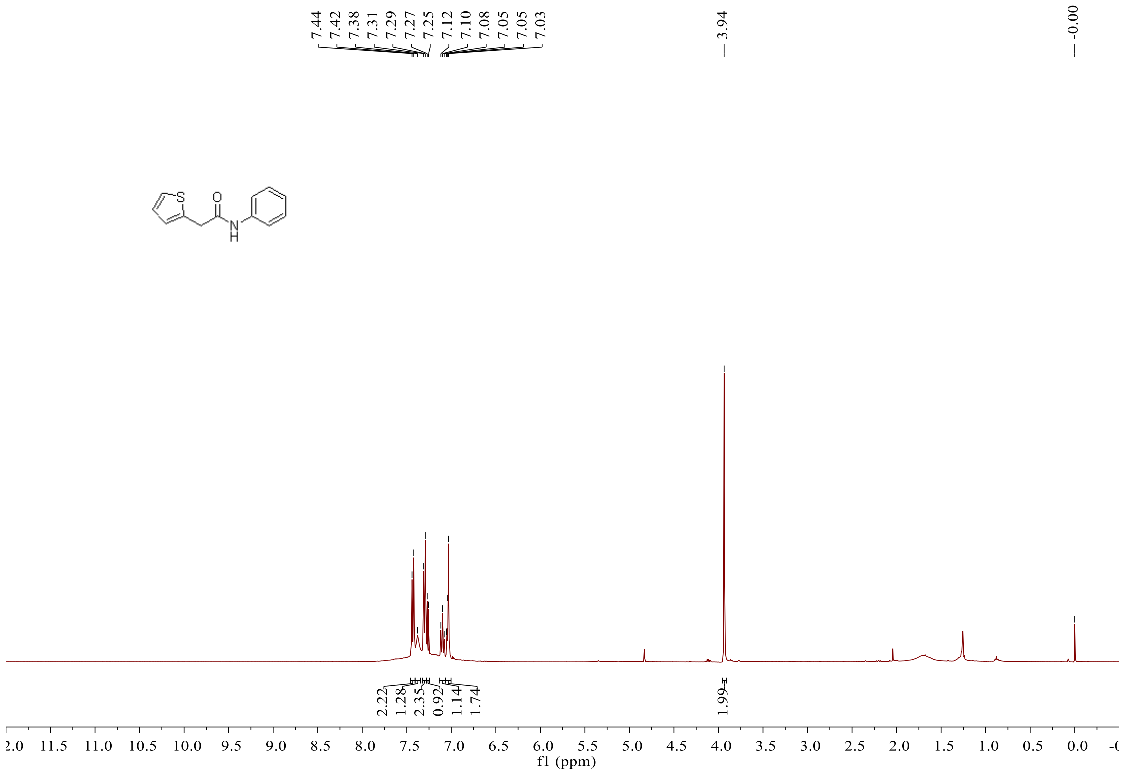

Supplementary Figure 67. ^{1}H NMR spectrum for compound **3ee**

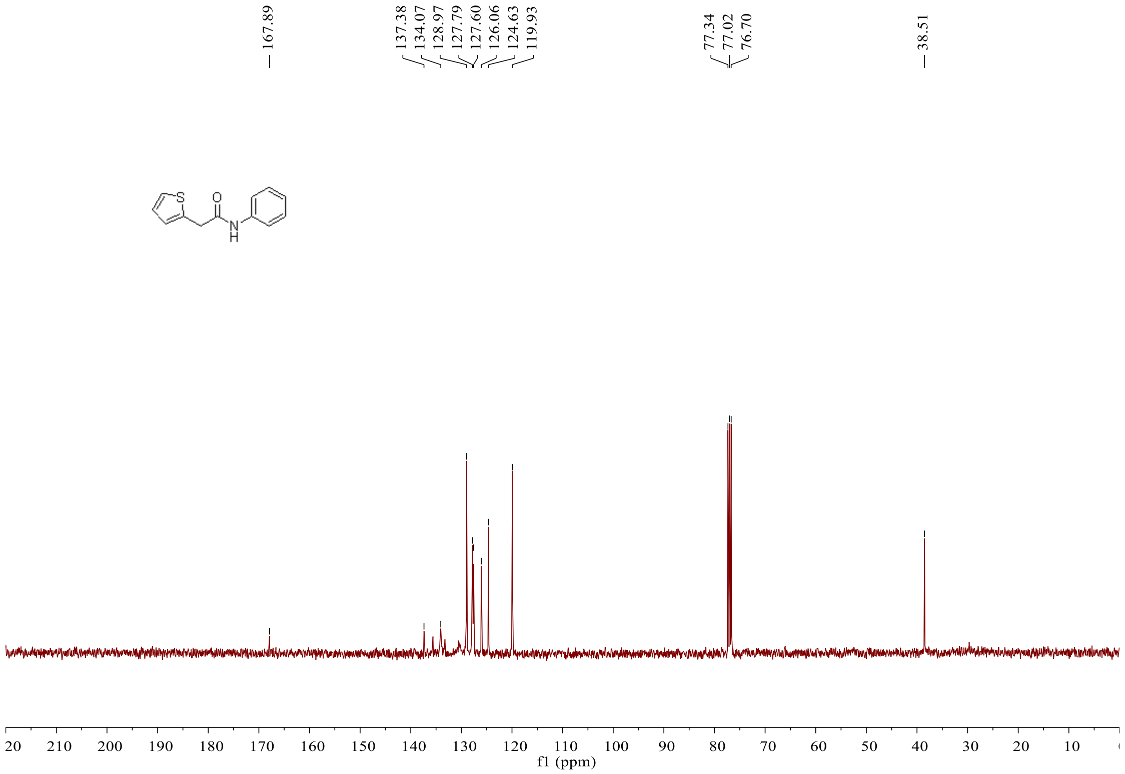

Supplementary Figure 68. ^{13}C NMR spectrum for compound **3ee**

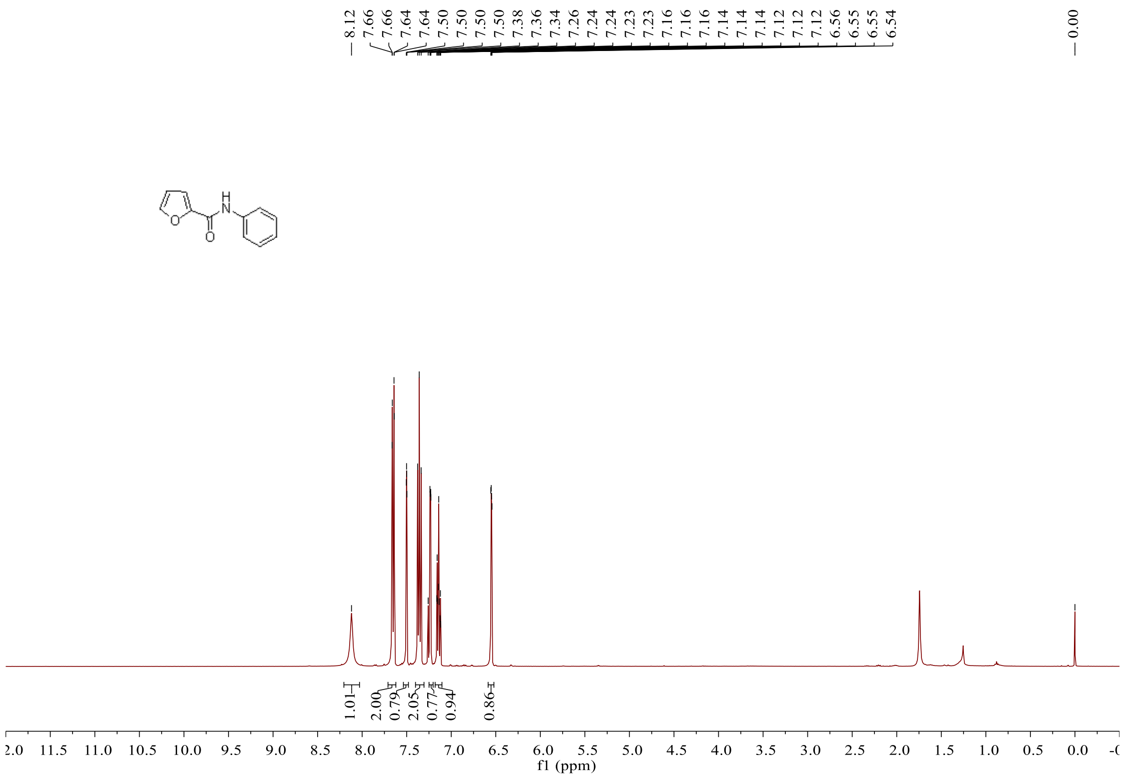

Supplementary Figure 69. ^{1}H NMR spectrum for compound **3ff**

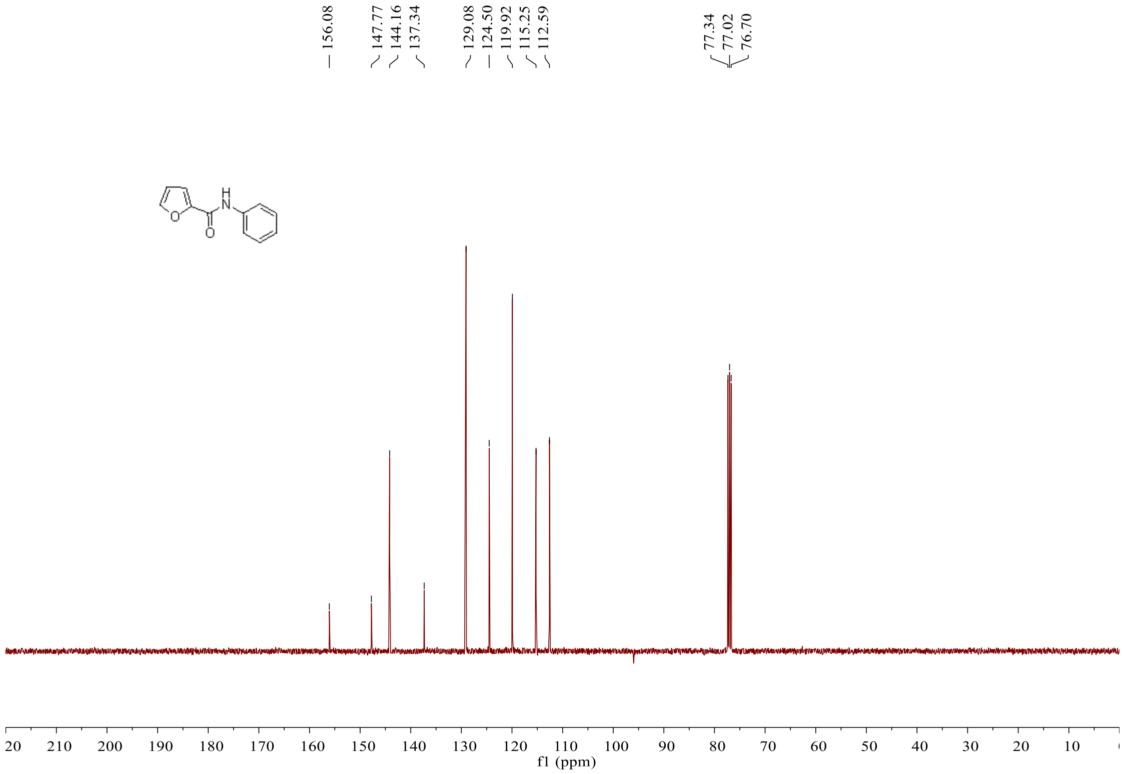

Supplementary Figure 70. ^{13}C NMR spectrum for compound **3ff**

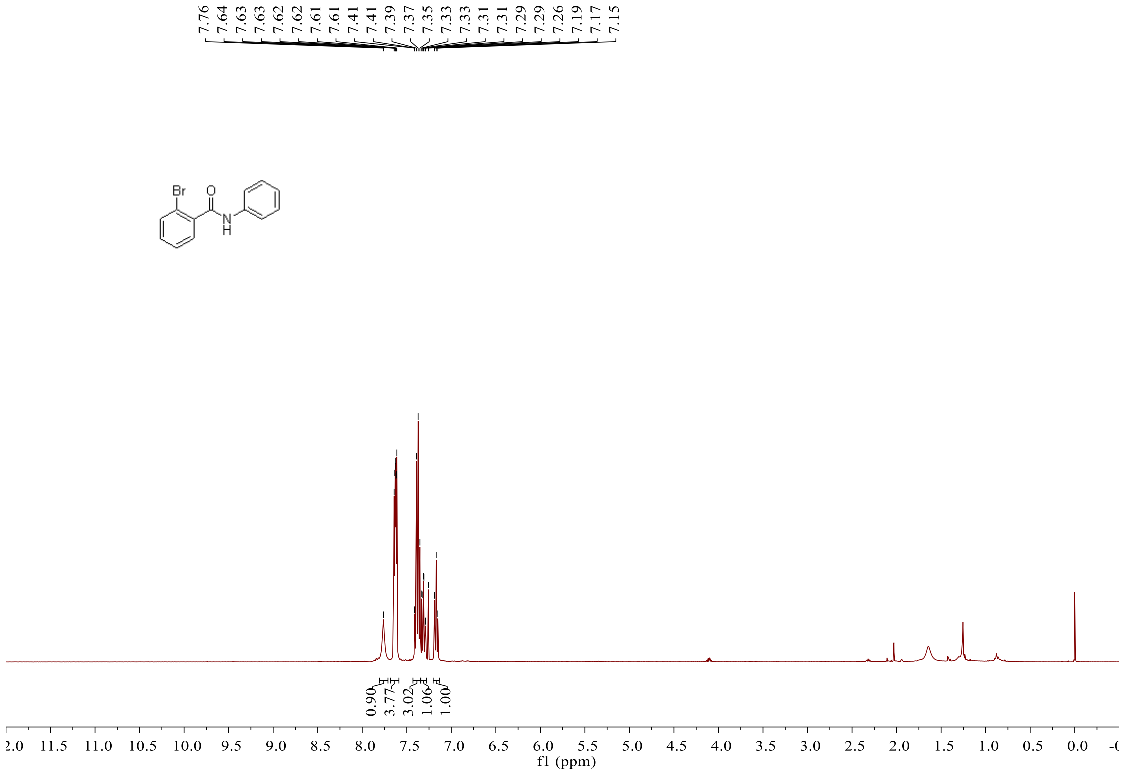

Supplementary Figure 71. ^{1}H NMR spectrum for compound **3gg**

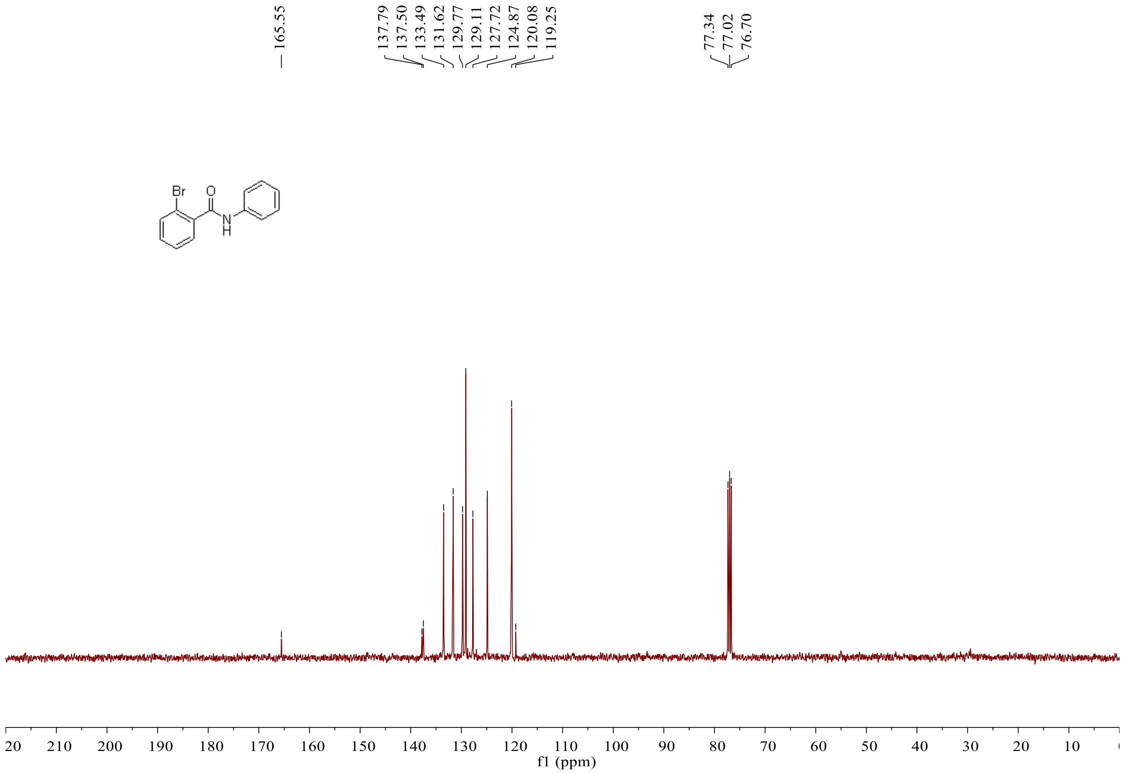

Supplementary Figure 72. ^{13}C NMR spectrum for compound **3gg**

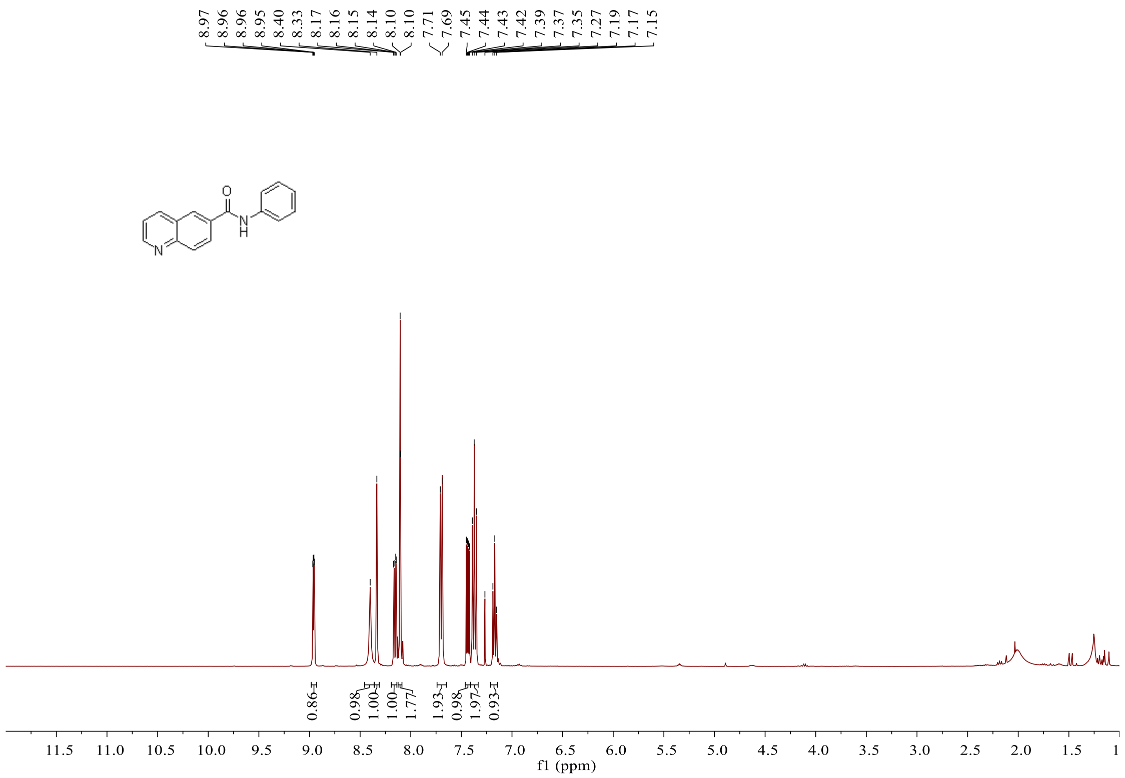

Supplementary Figure 73. ^{1}H NMR spectrum for compound **3hh**

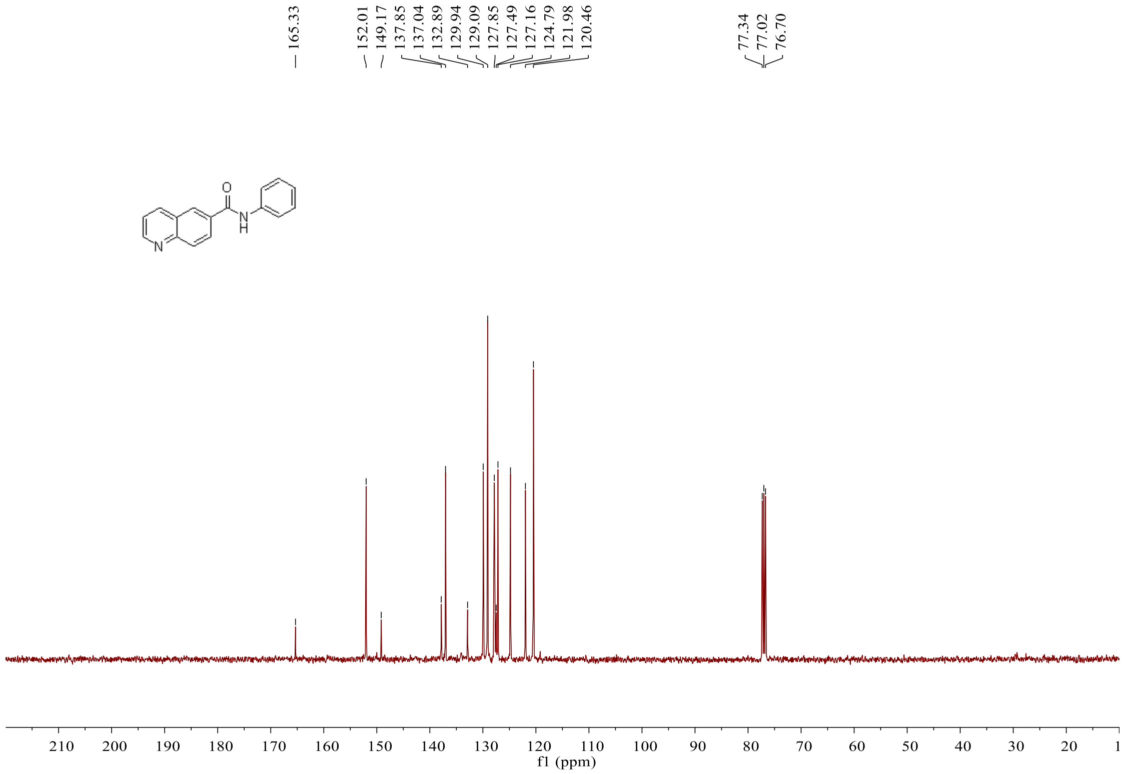

Supplementary Figure 74. ^{13}C NMR spectrum for compound **3hh**

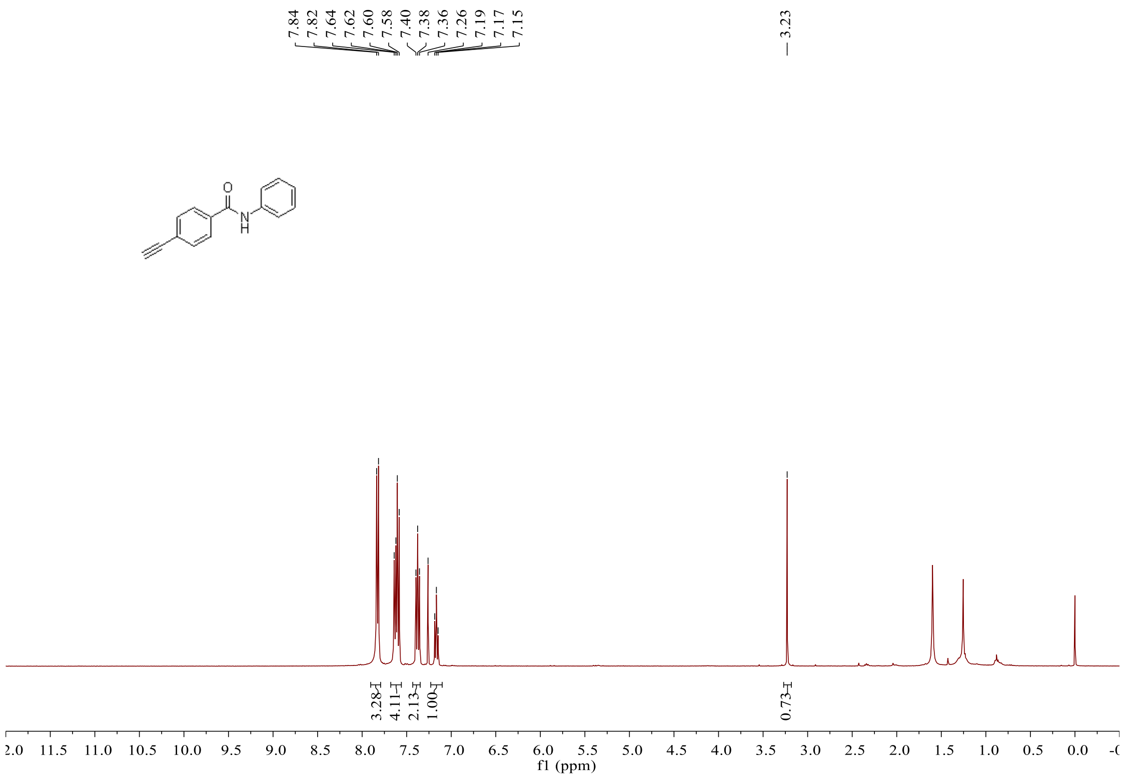

Supplementary Figure 75. ^{1}H NMR spectrum for compound **3ii**

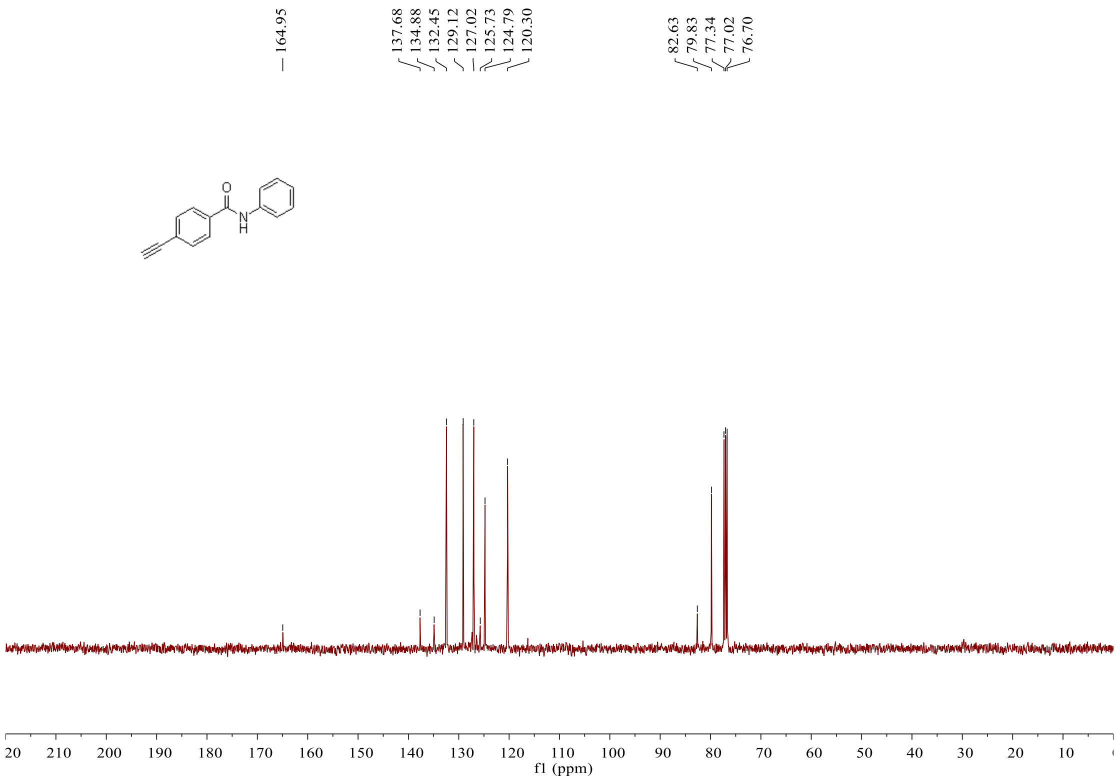

Supplementary Figure 76. ^{13}C NMR spectrum for compound **3ii**

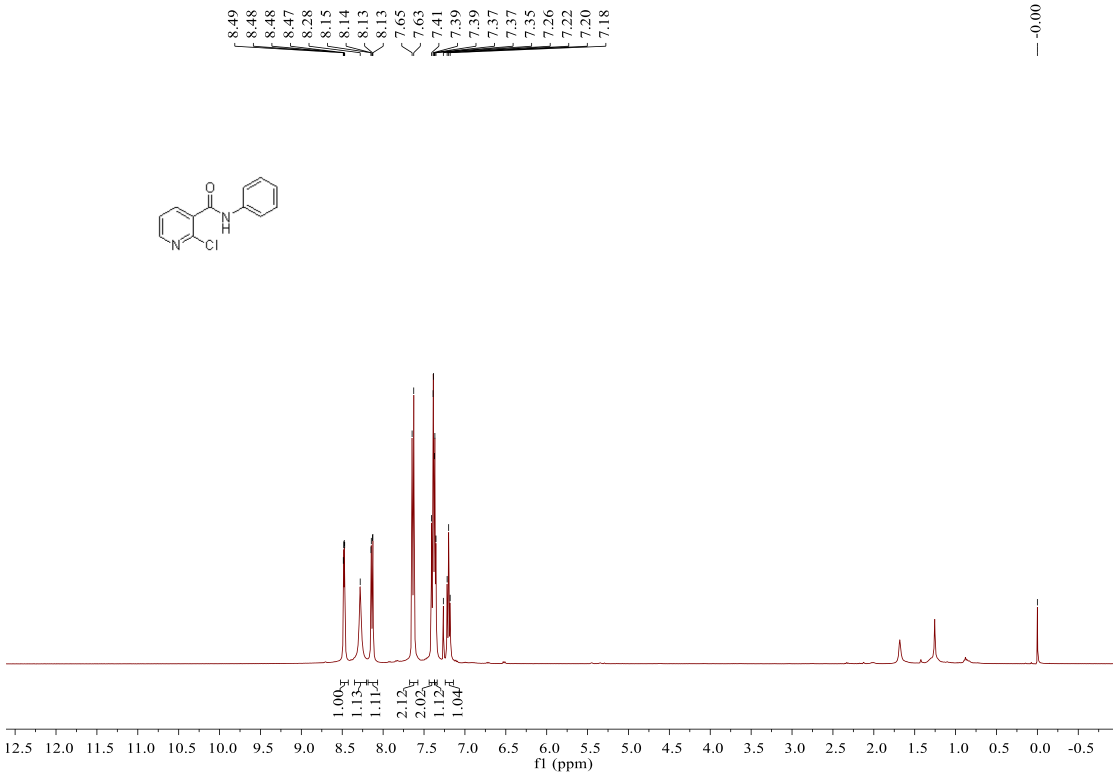

Supplementary Figure 77. ^{1}H NMR spectrum for compound **3jj**

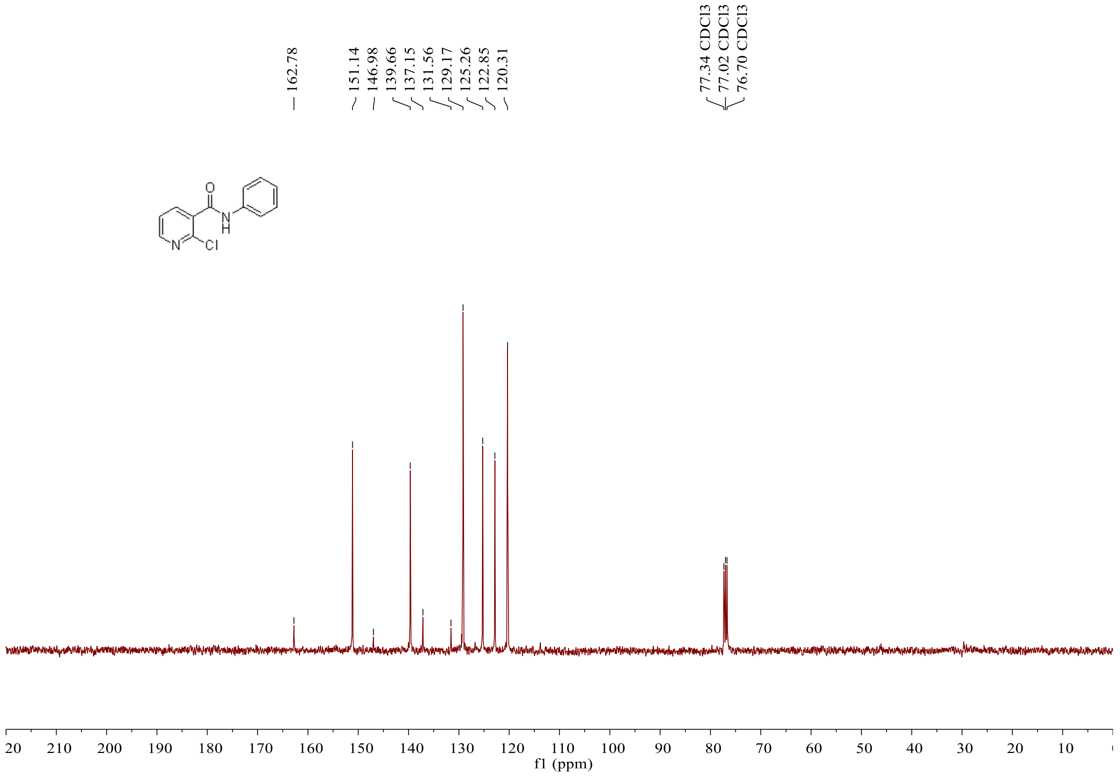

Supplementary Figure 78. ^{13}C NMR spectrum for compound **3jj**

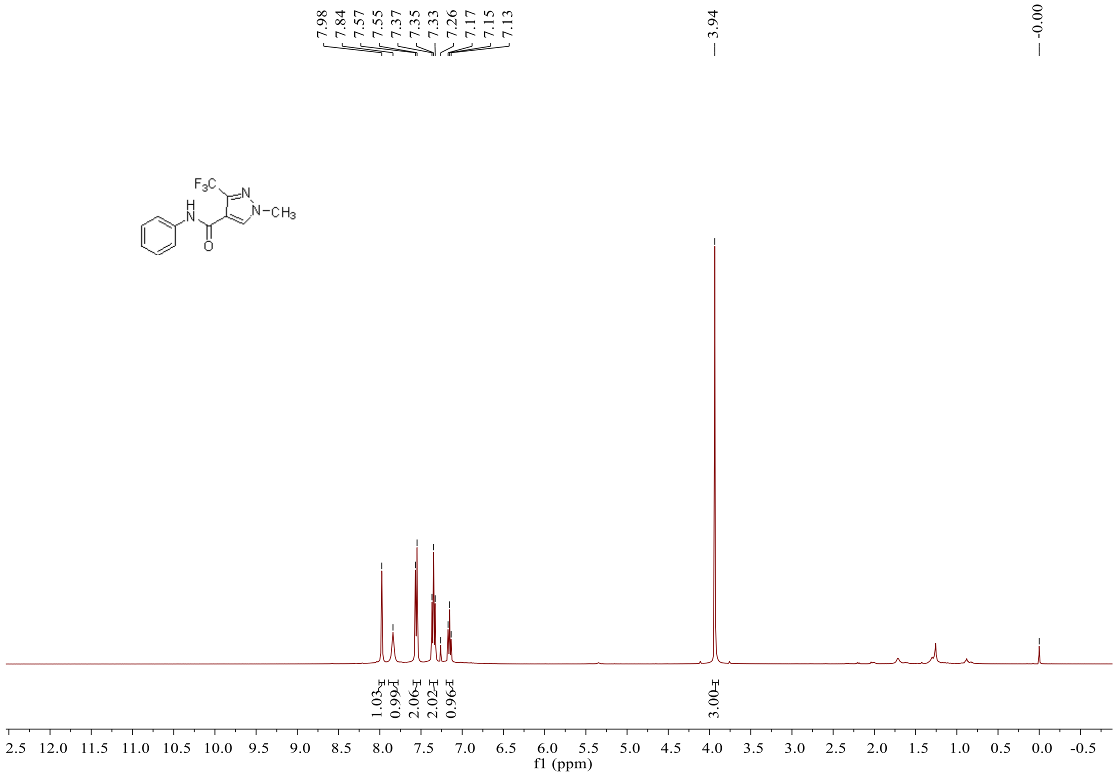

Supplementary Figure 79. ^{1}H NMR spectrum for compound **3kk**

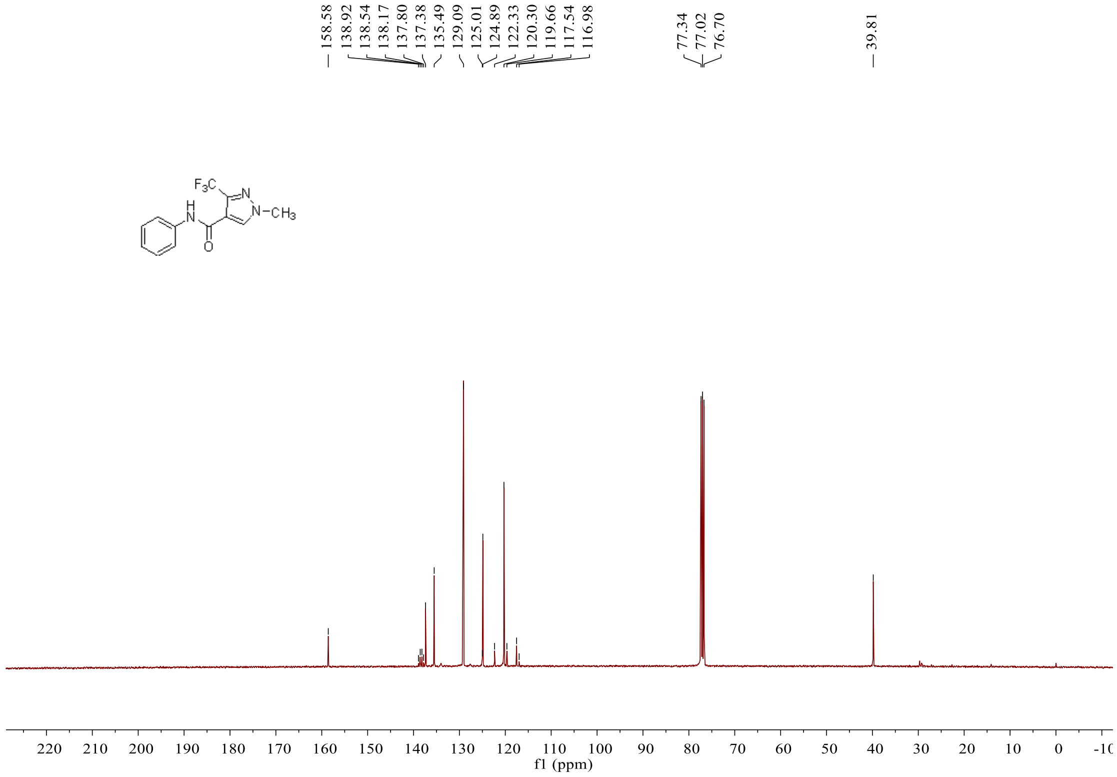

Supplementary Figure 80. ^{13}C NMR spectrum for compound **3kk**

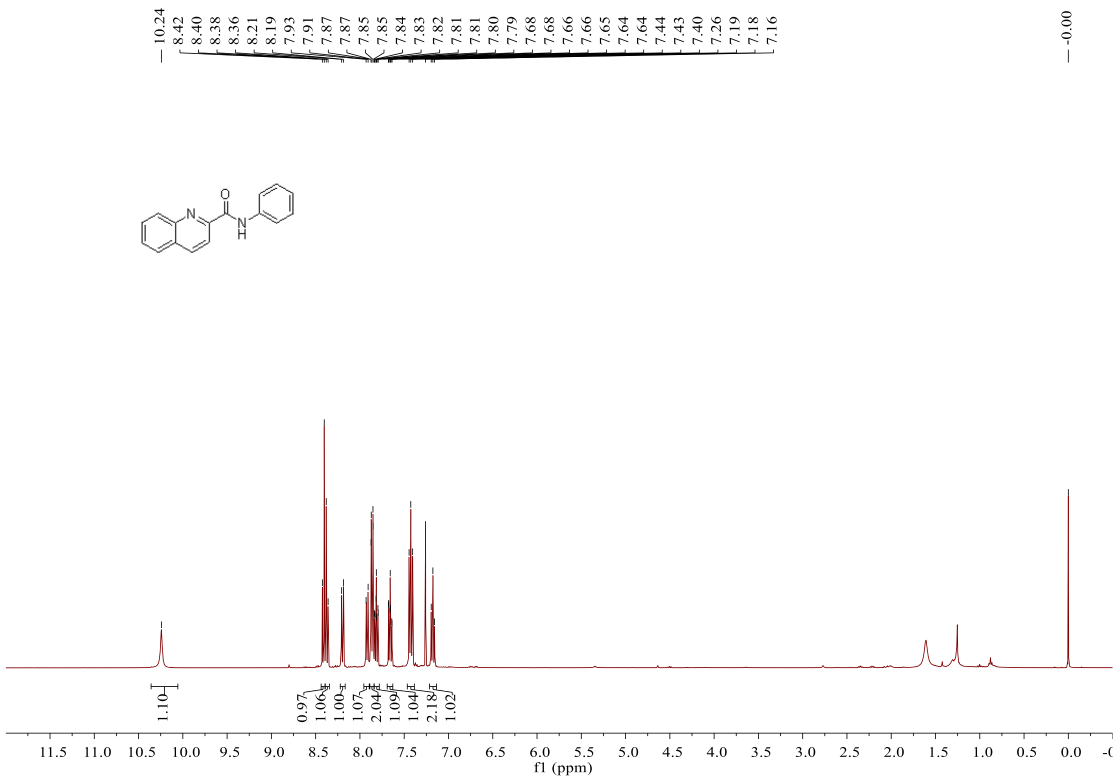

Supplementary Figure 81. ^{1}H NMR spectrum for compound **3ll**

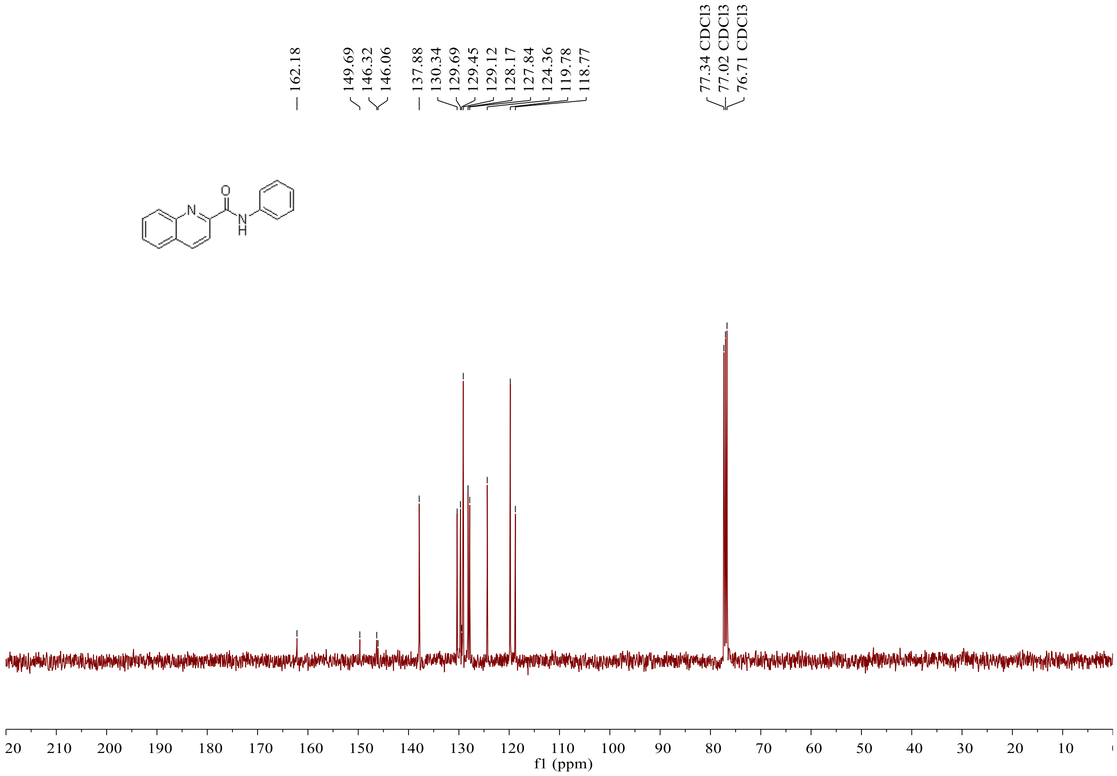

Supplementary Figure 82. ^{13}C NMR spectrum for compound **3ll**

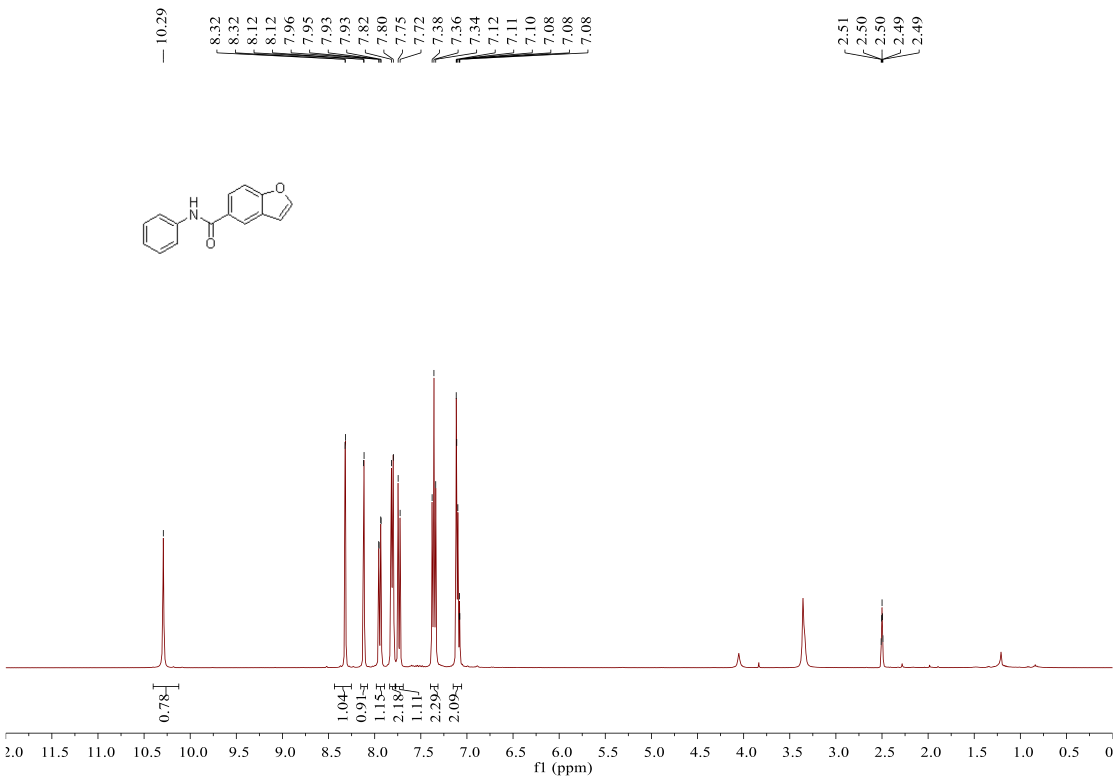

Supplementary Figure 83. ^{1}H NMR spectrum for compound **3mm**

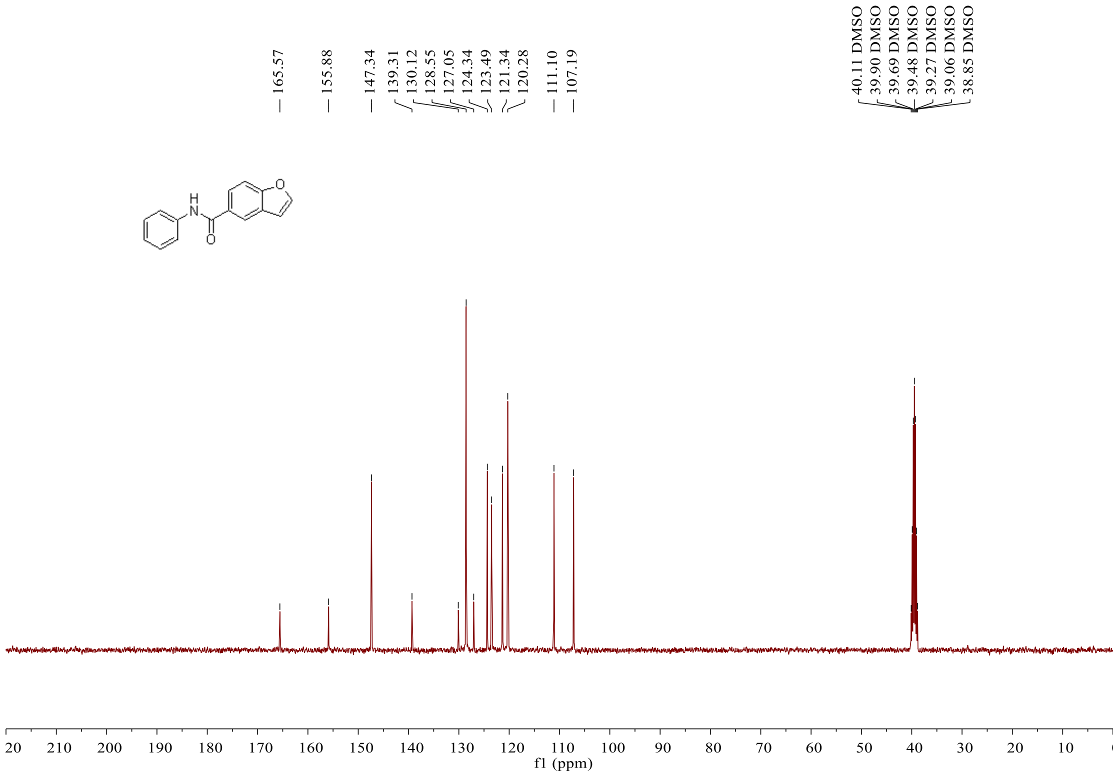

Supplementary Figure 84. ^{13}C NMR spectrum for compound **3mm**

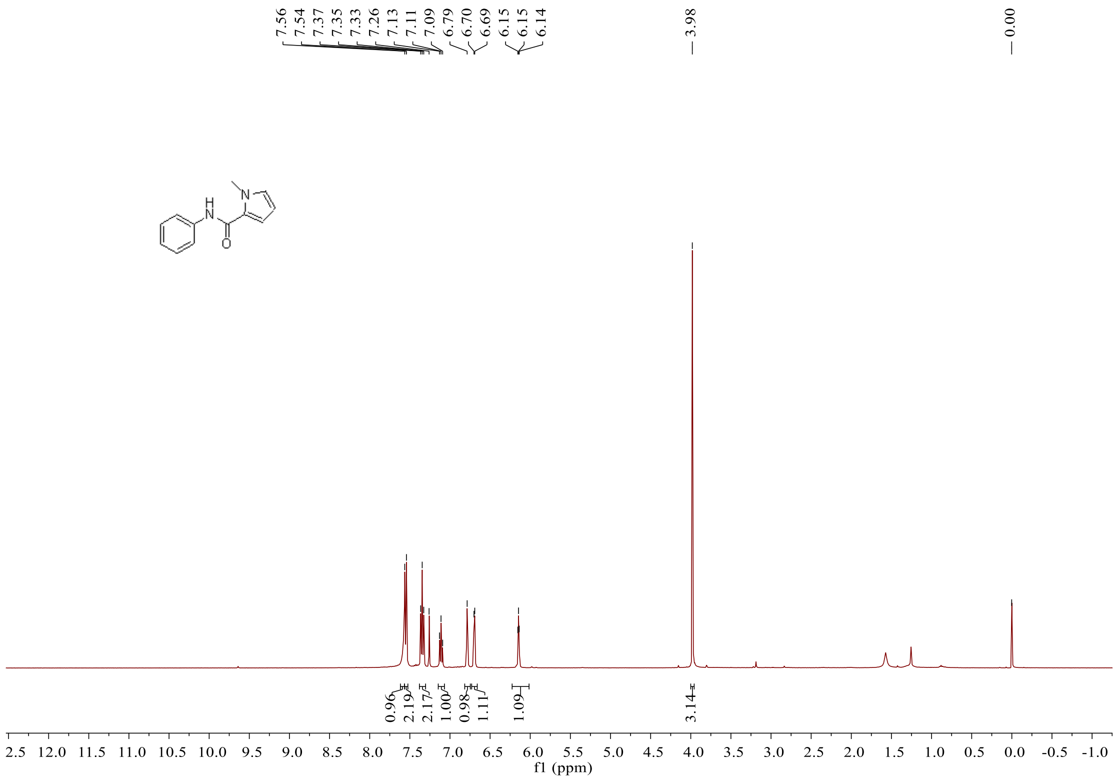

Supplementary Figure 85. ^{1}H NMR spectrum for compound **3nn**

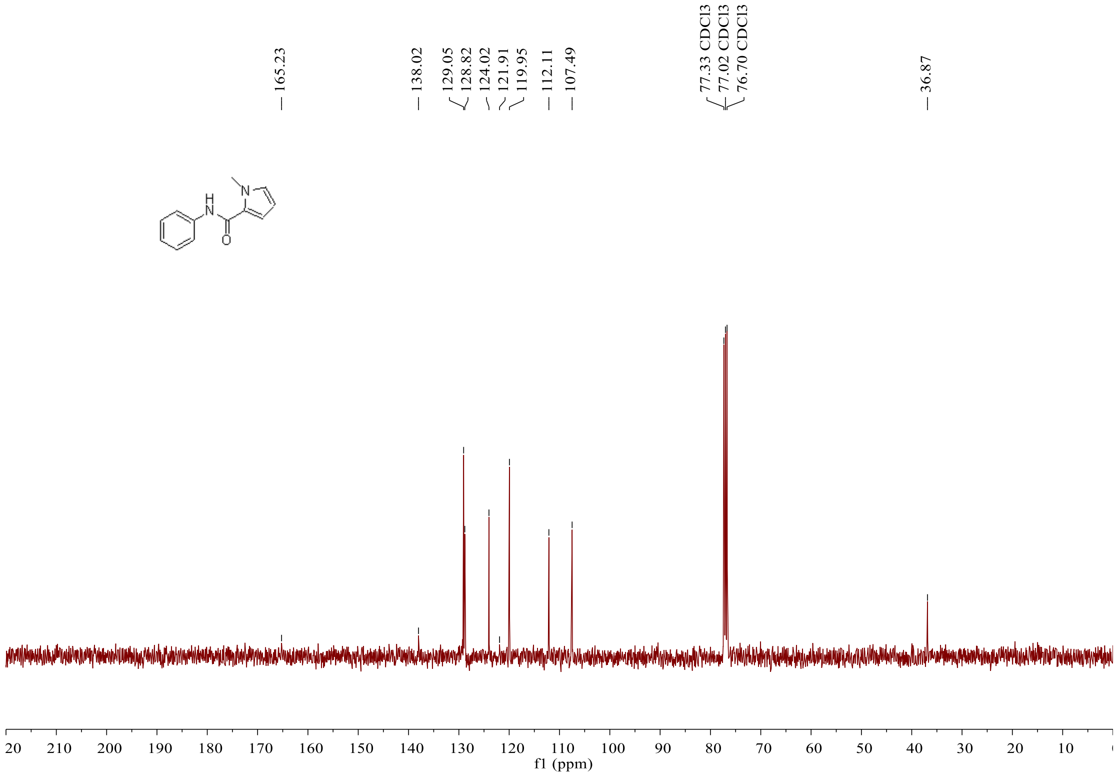

Supplementary Figure 86. ^{13}C NMR spectrum for compound **3nn**

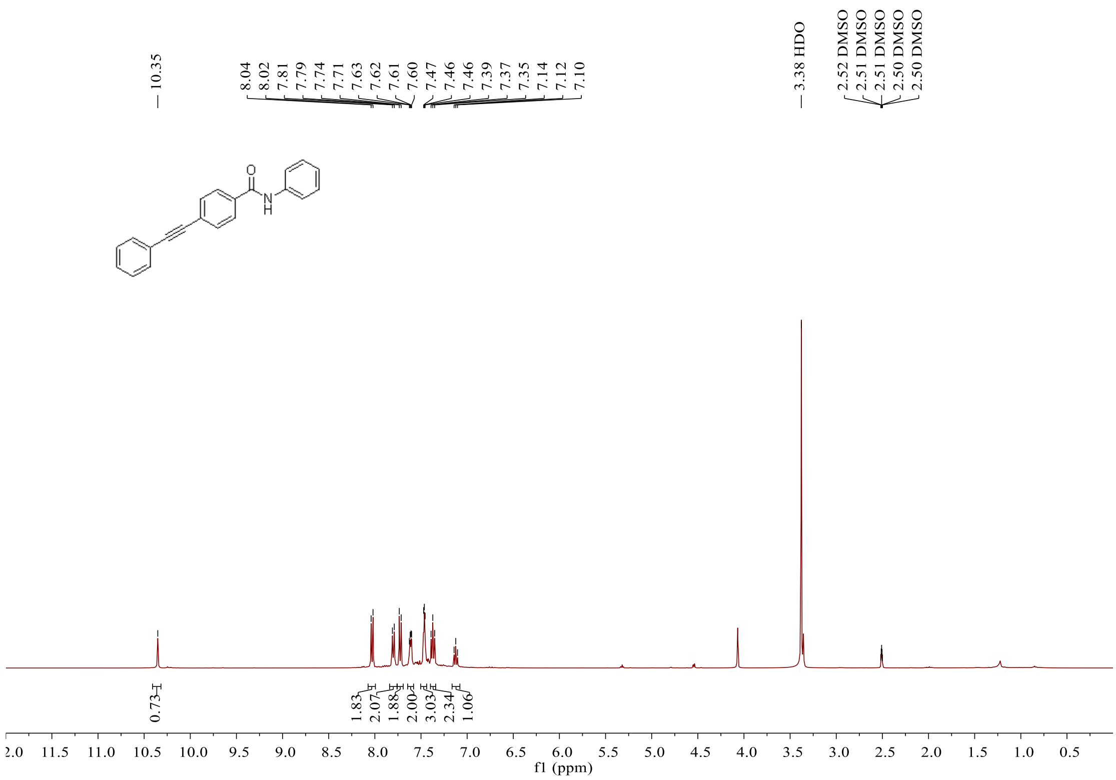

Supplementary Figure 87. ^{1}H NMR spectrum for compound **3oo**

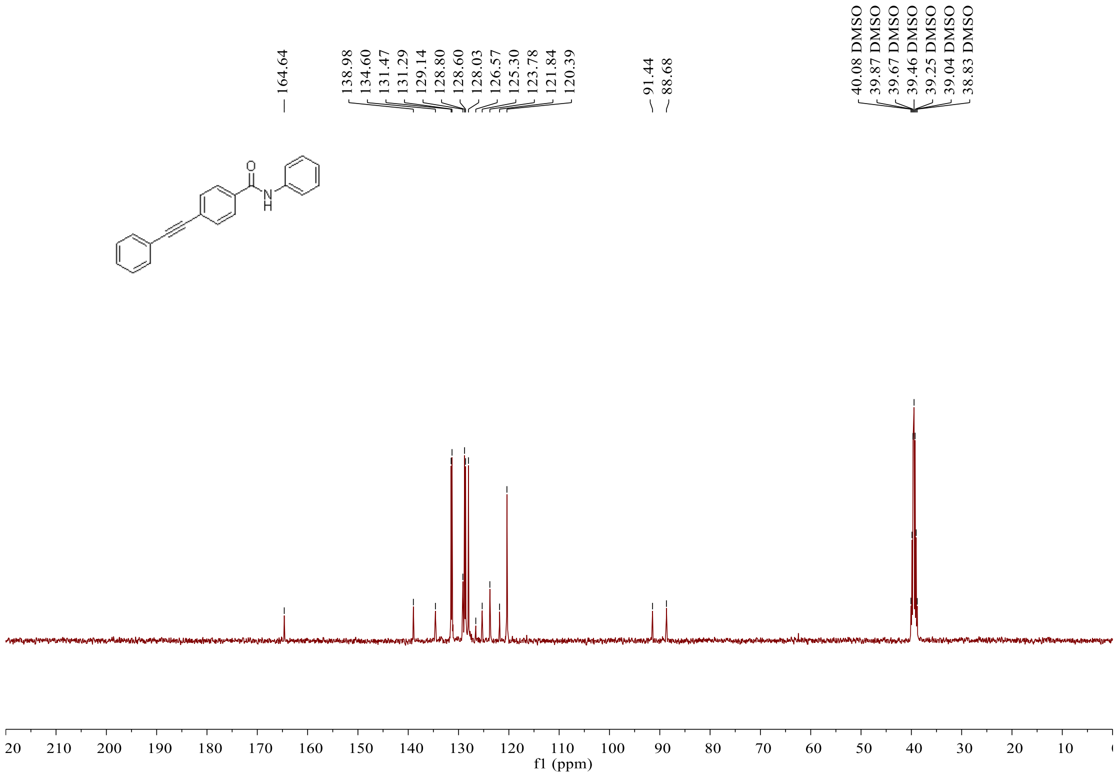

Supplementary Figure 88. ^{13}C NMR spectrum for compound **3oo**

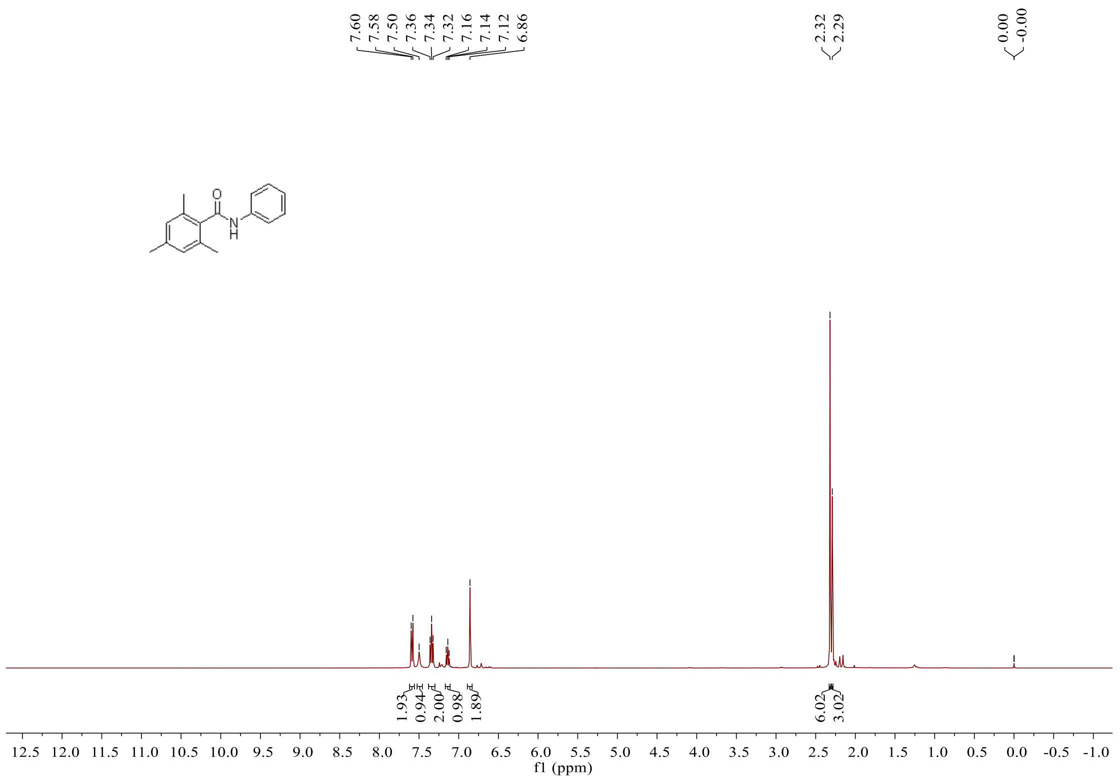

Supplementary Figure 89. ^{1}H NMR spectrum for compound **3pp**

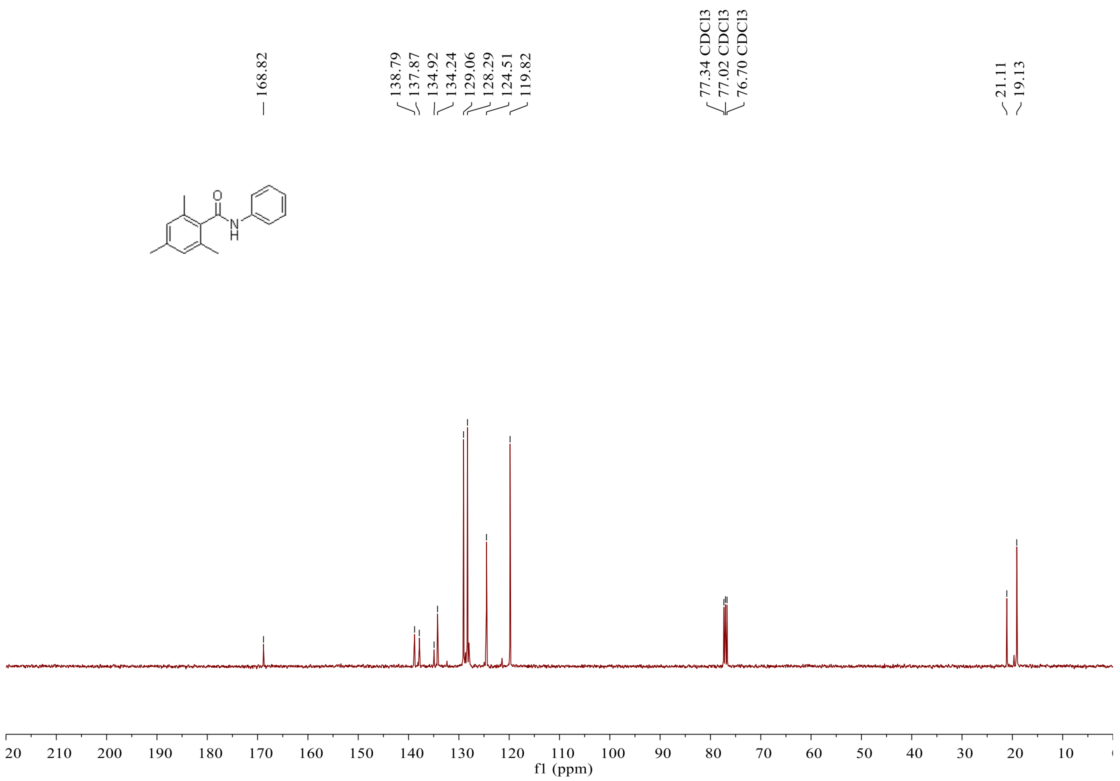

Supplementary Figure 90. ^{13}C NMR spectrum for compound **3pp**

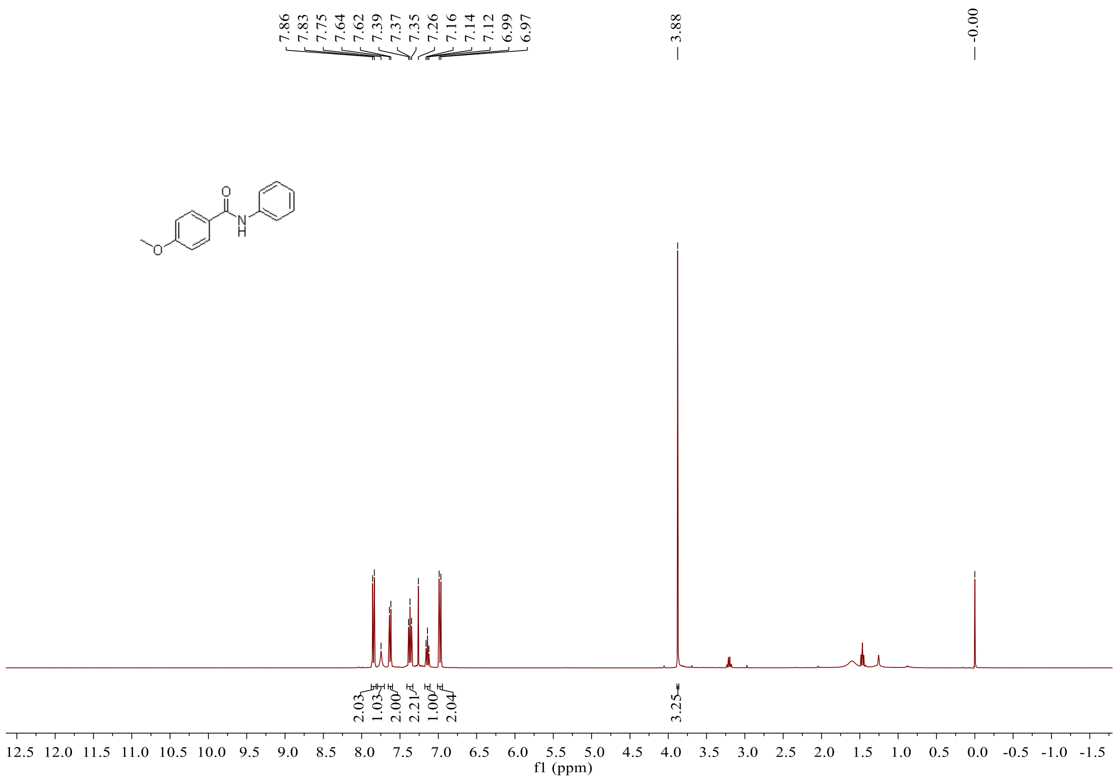

Supplementary Figure 91. ^{1}H NMR spectrum for compound **3qq**

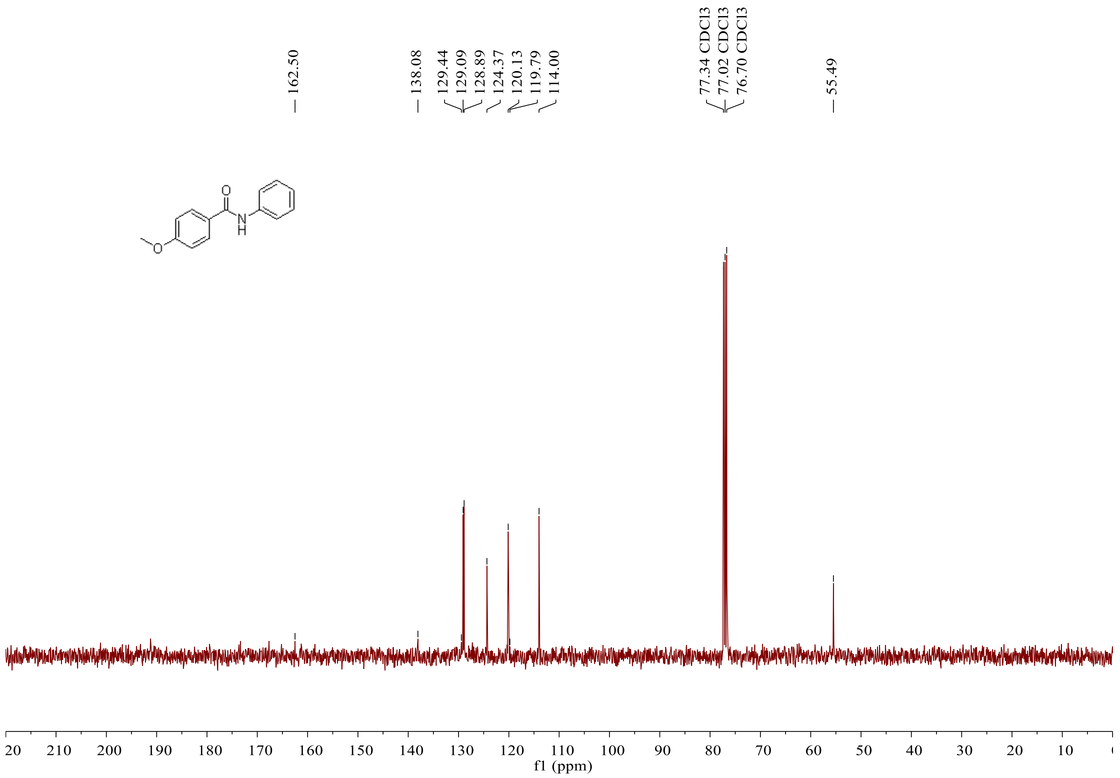

Supplementary Figure 92. ^{13}C NMR spectrum for compound **3qq**

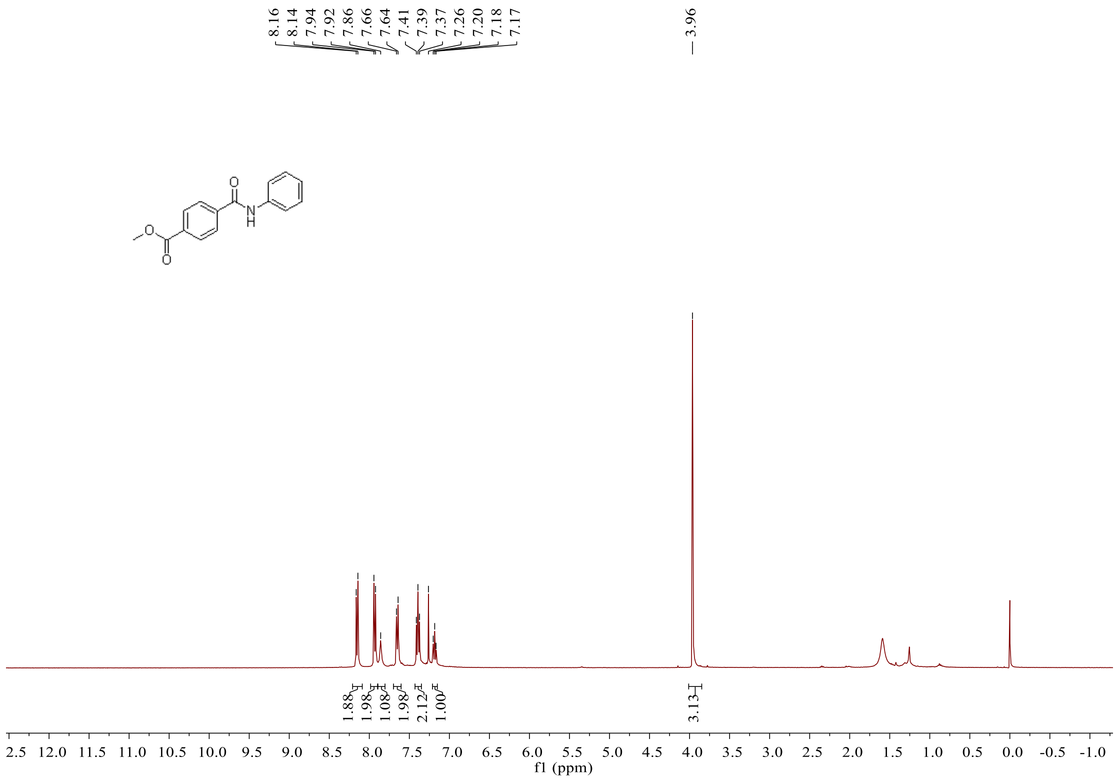

Supplementary Figure 93. ^{1}H NMR spectrum for compound **3rr**

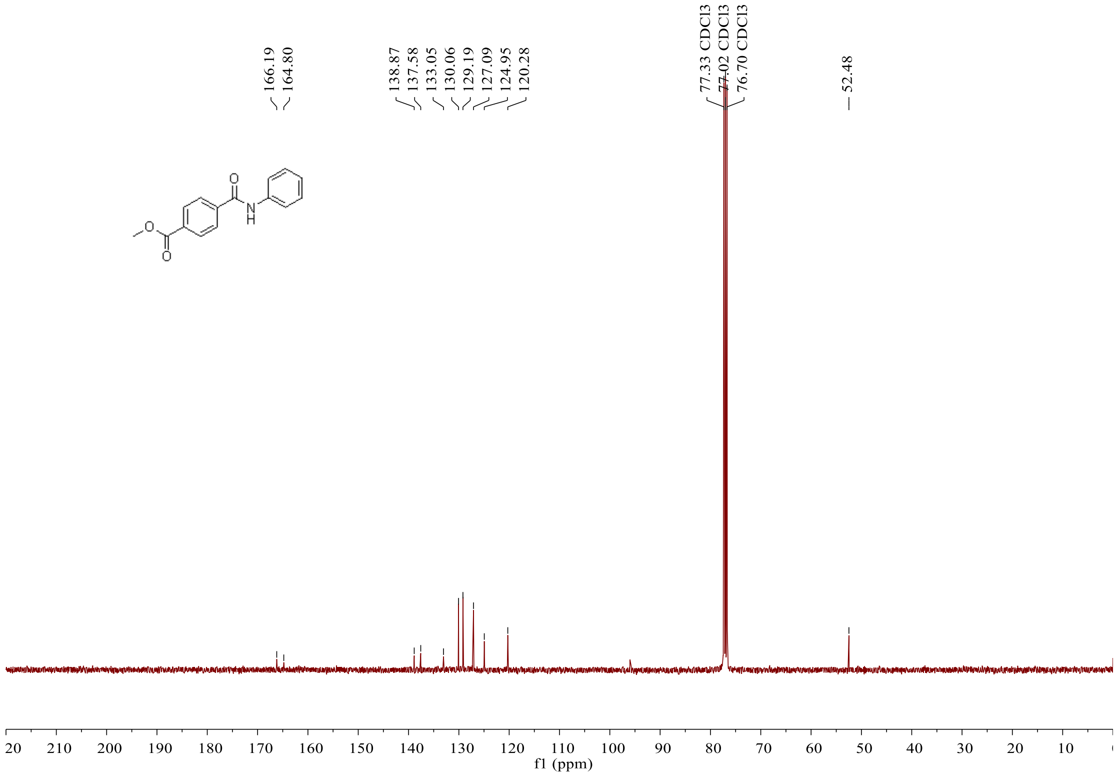

Supplementary Figure 94. ^{13}C NMR spectrum for compound **3rr**

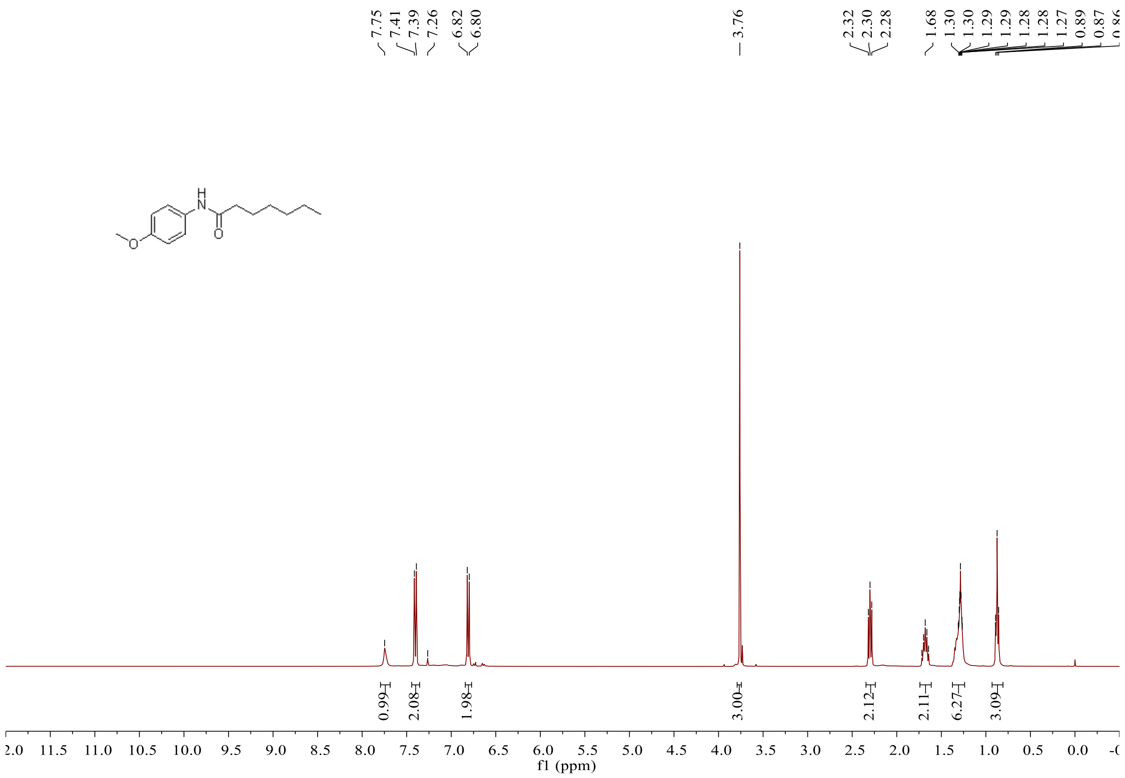

Supplementary Figure 95. ^{1}H NMR spectrum for compound **3ss**

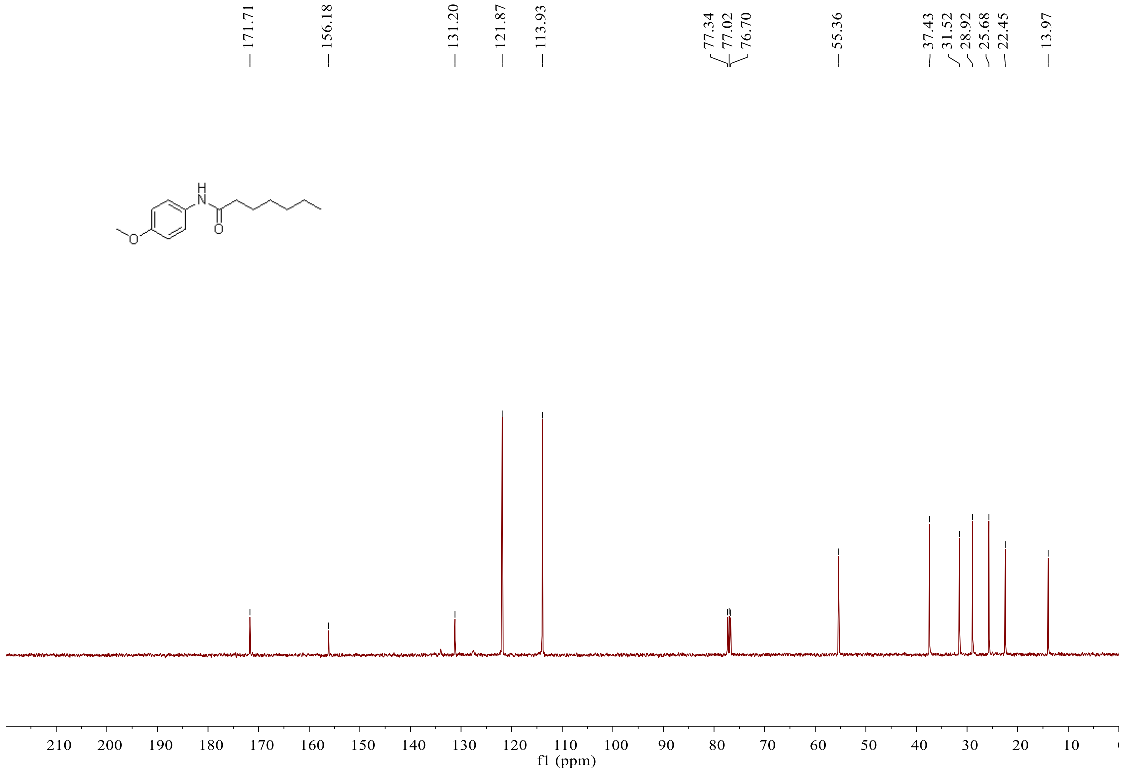

Supplementary Figure 96. ^{13}C NMR spectrum for compound **3ss**

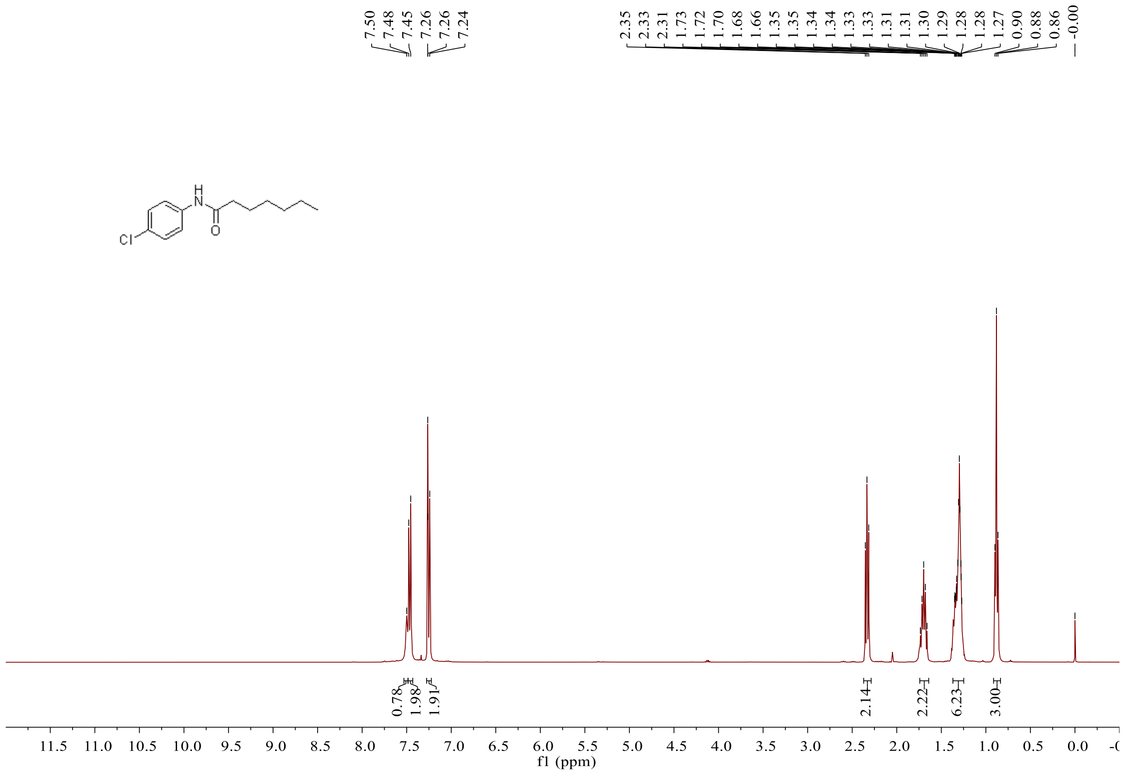

Supplementary Figure 97. ^{1}H NMR spectrum for compound **3tt**

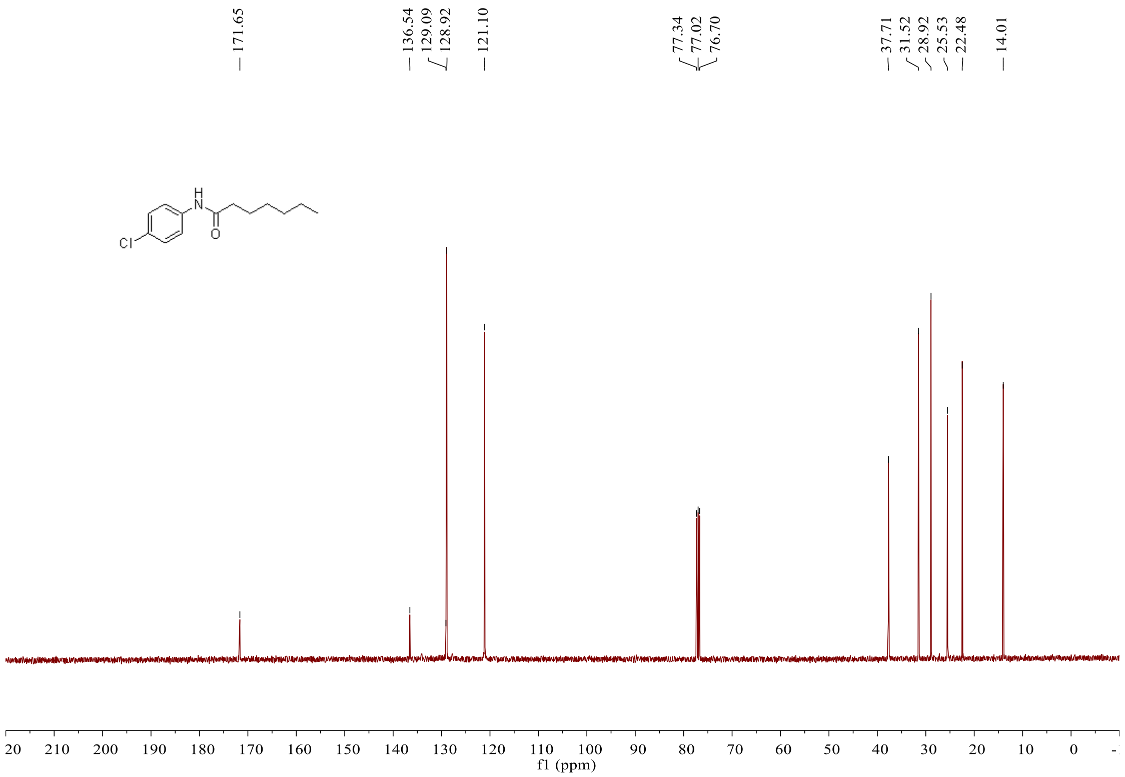

Supplementary Figure 98. ^{13}C NMR spectrum for compound **3tt**

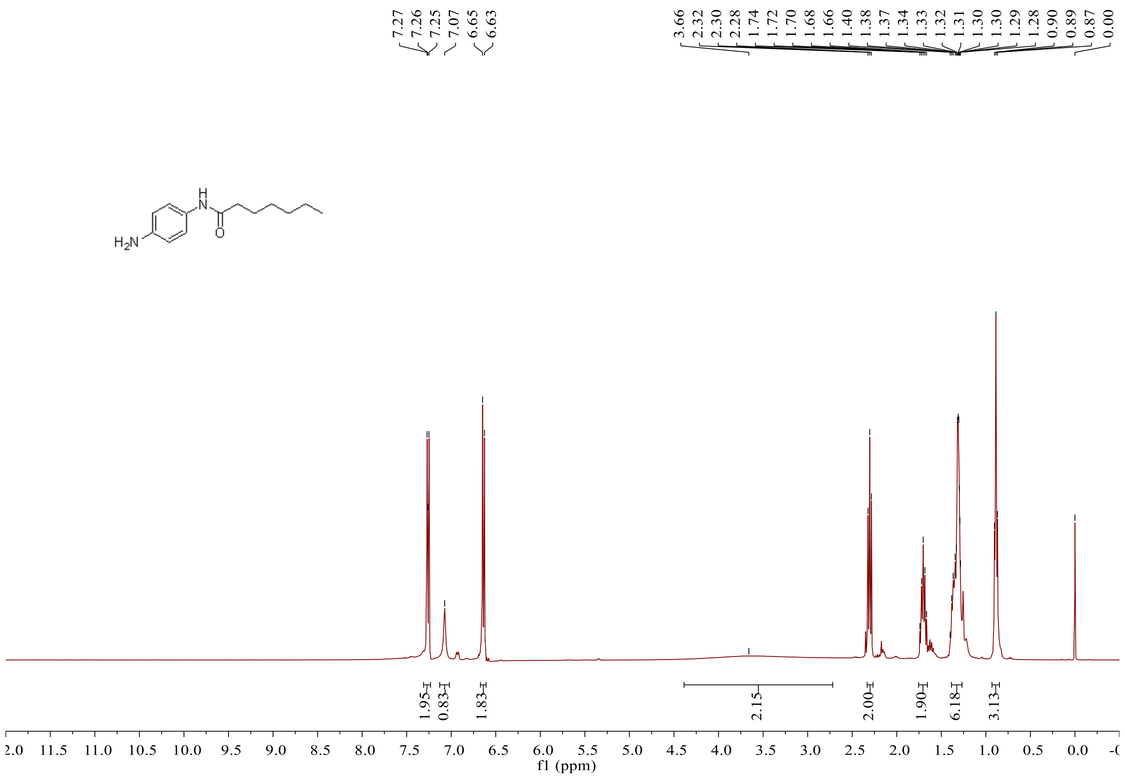

Supplementary Figure 99. ^{1}H NMR spectrum for compound **3uu**

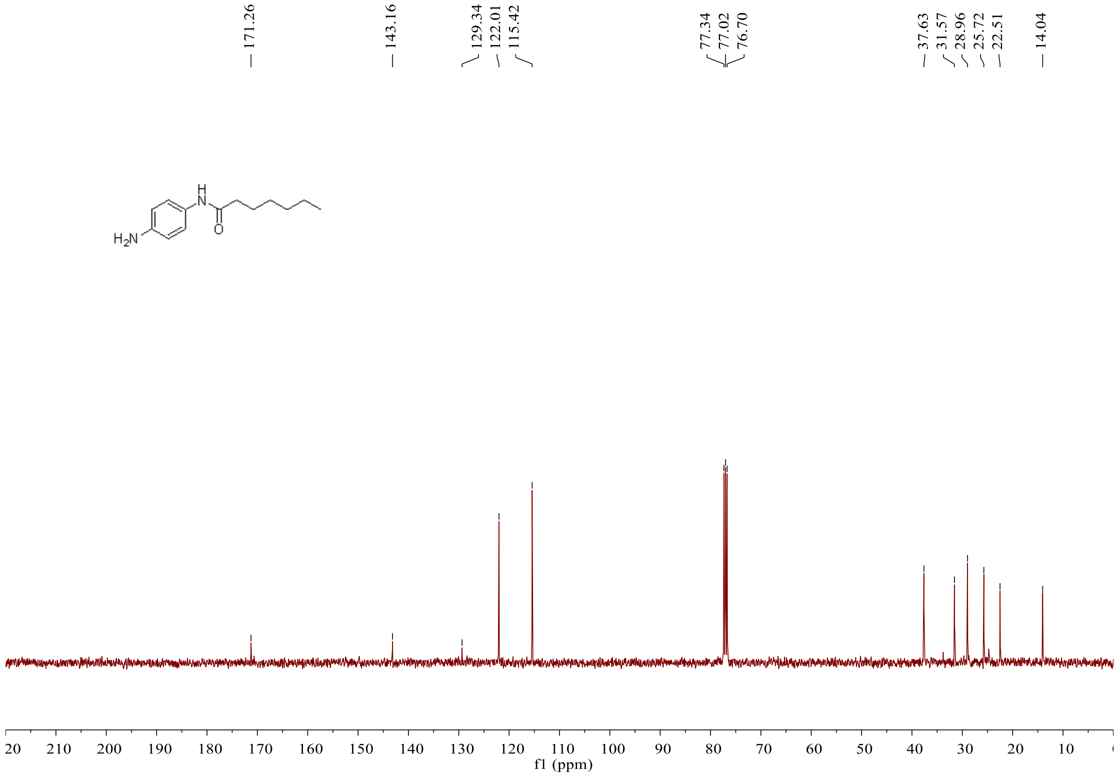

Supplementary Figure 100. ^{13}C NMR spectrum for compound **3uu**

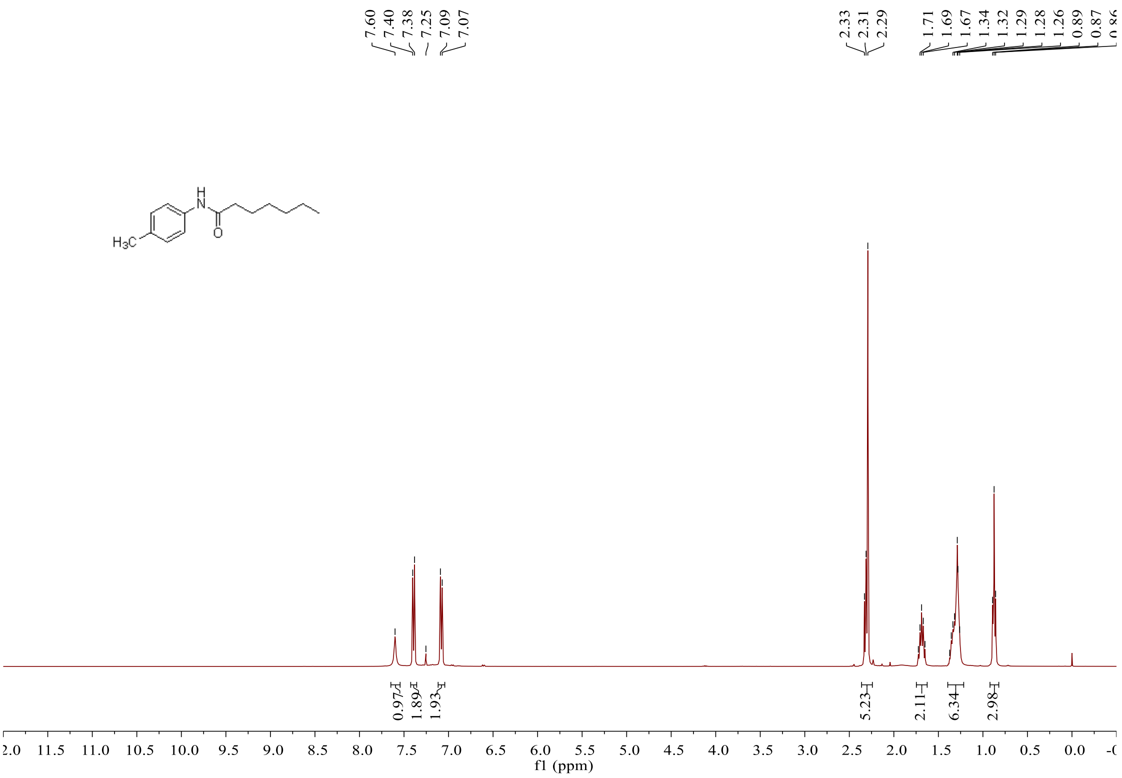

Supplementary Figure 101. ^{1}H NMR spectrum for compound **3vv**

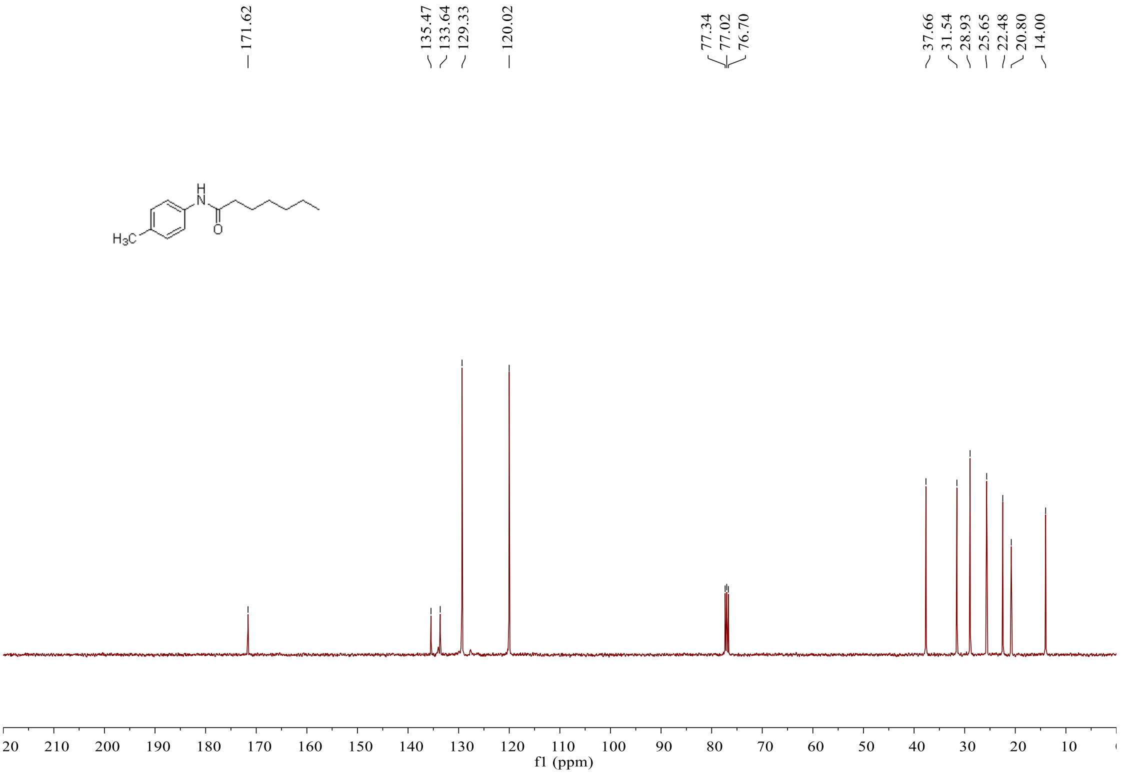

Supplementary Figure 102. ^{13}C NMR spectrum for compound **3vv**

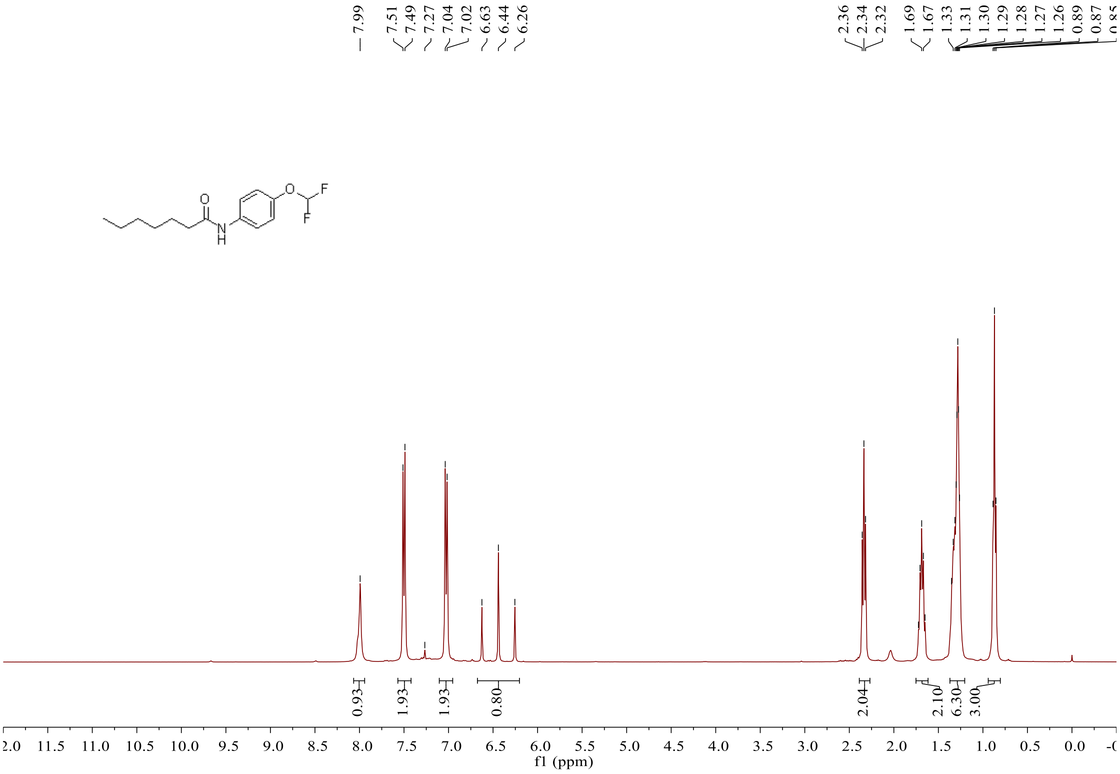

Supplementary Figure 103. ^{1}H NMR spectrum for compound **3ww**

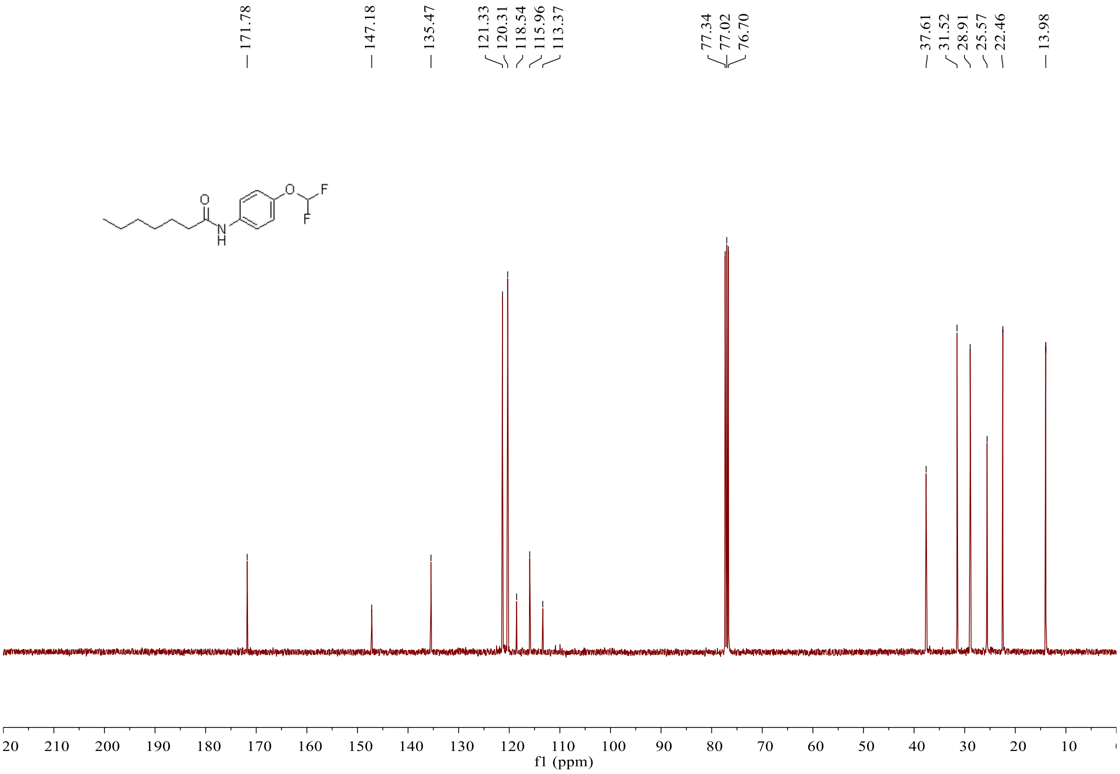

Supplementary Figure 104. ^{13}C NMR spectrum for compound **3ww**

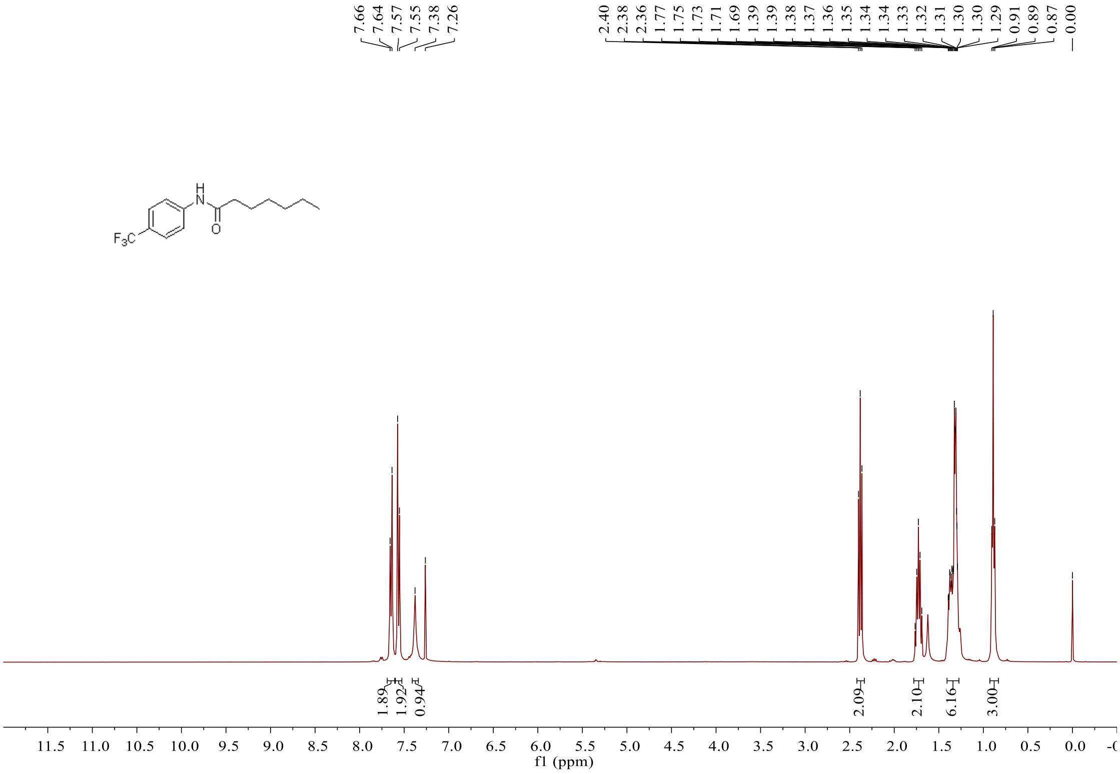

Supplementary Figure 105. ^{1}H NMR spectrum for compound **3xx**

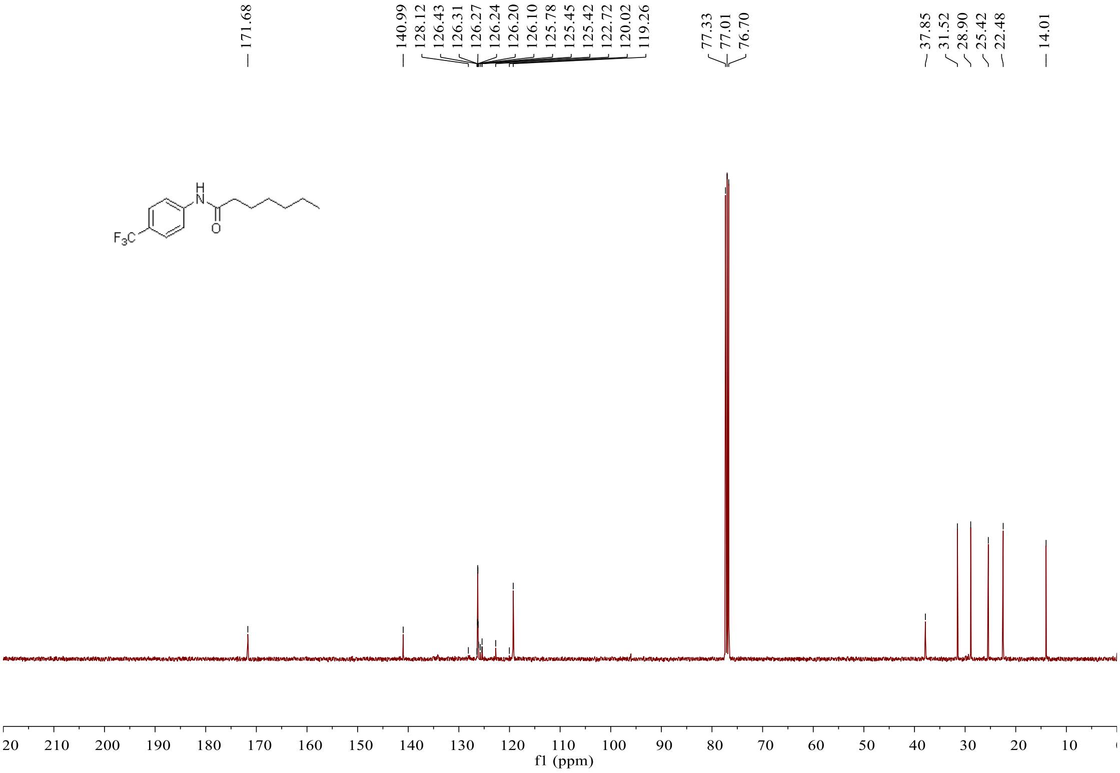

Supplementary Figure 106. ^{13}C NMR spectrum for compound **3xx**

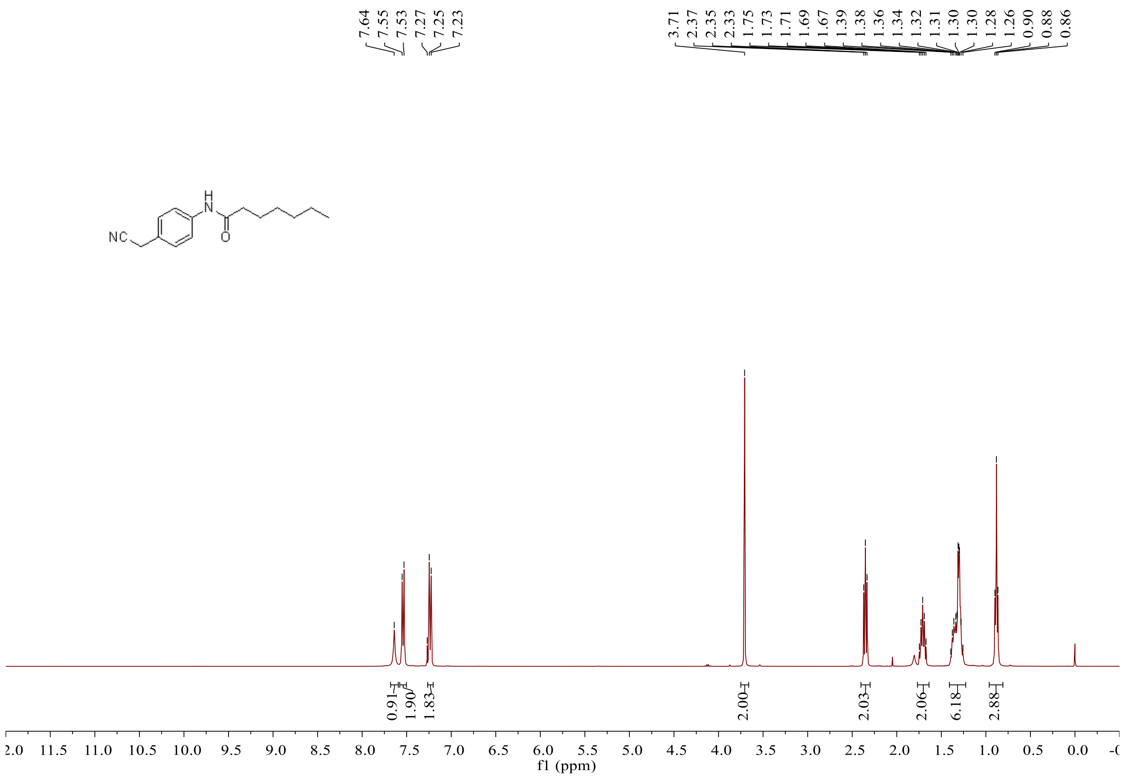

Supplementary Figure 107. ^{1}H NMR spectrum for compound **3yy**

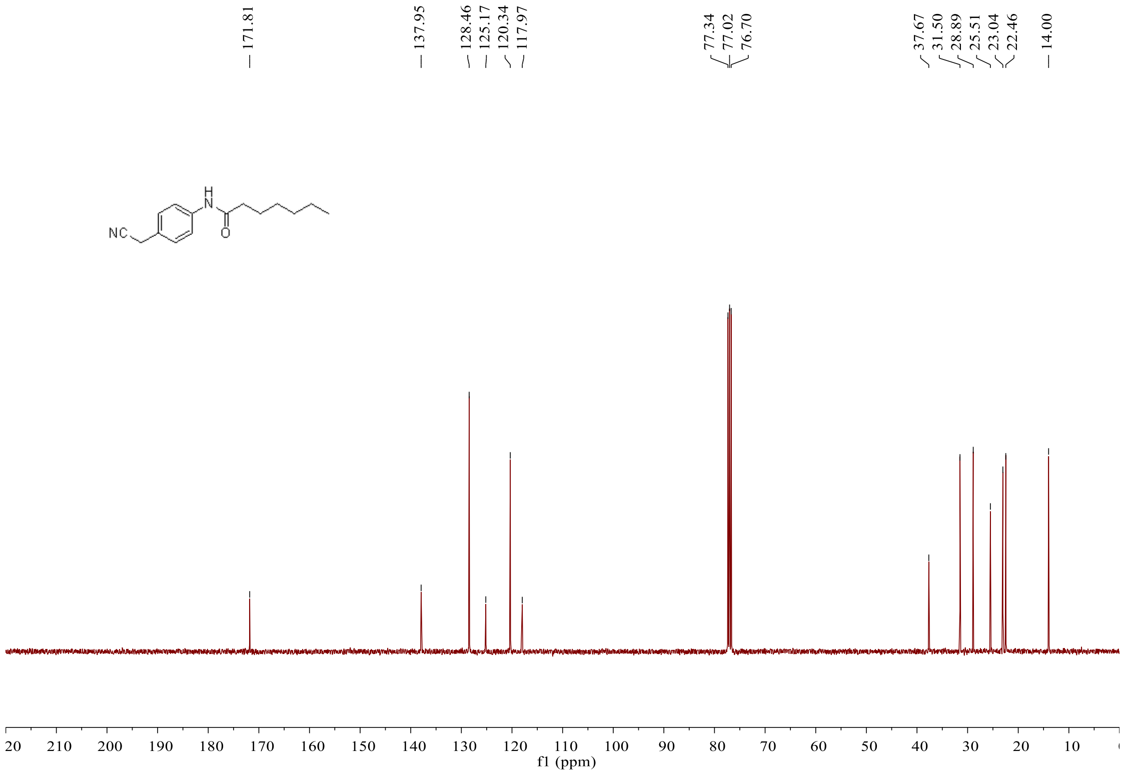

Supplementary Figure 108. ^{13}C NMR spectrum for compound **3yy**

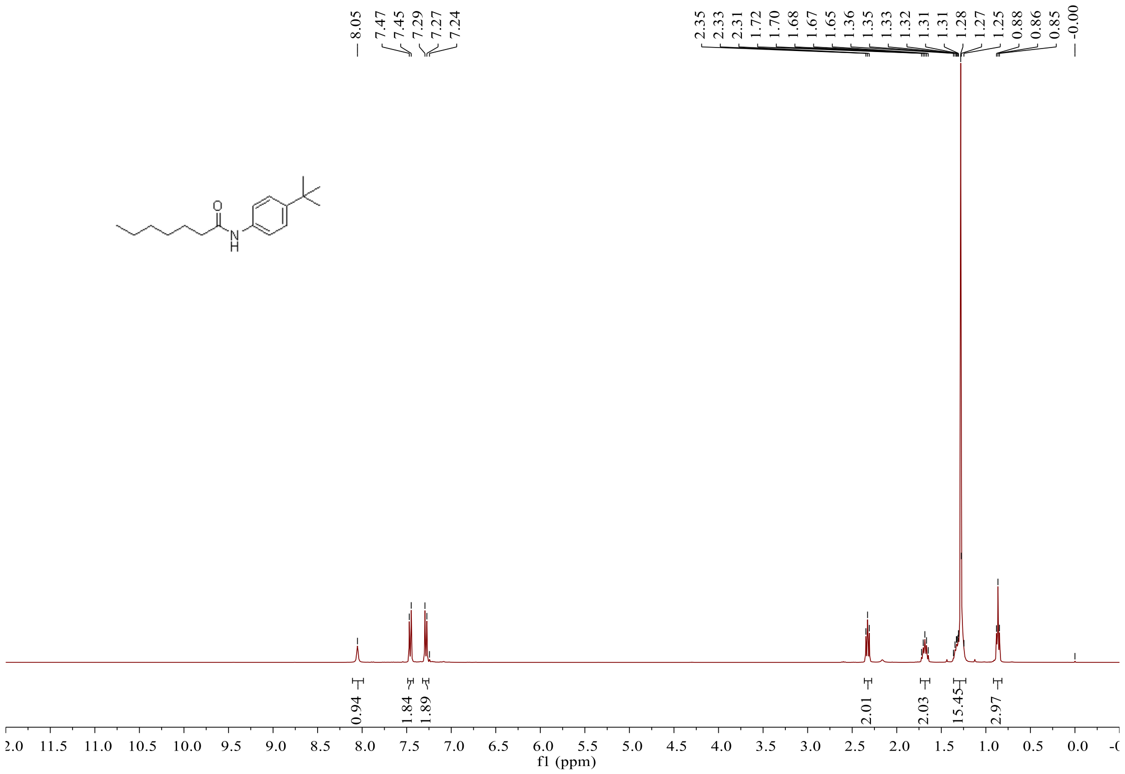

Supplementary Figure 109. ^{1}H NMR spectrum for compound **3zz**

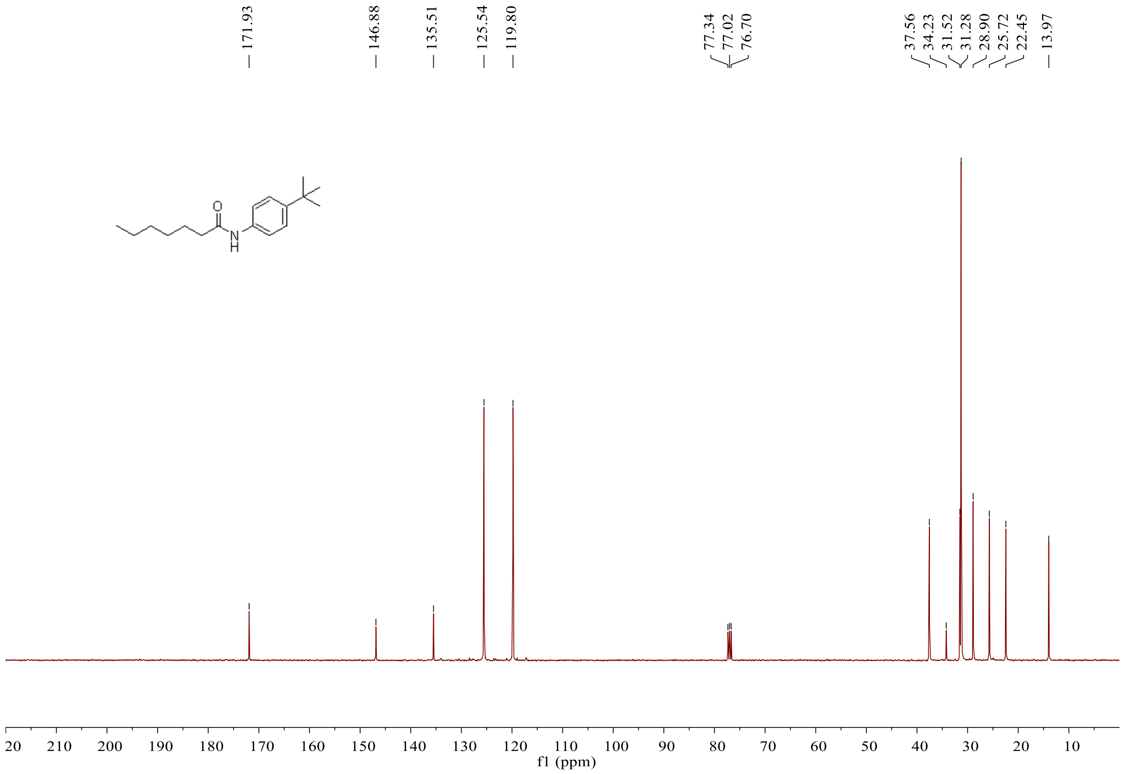

Supplementary Figure 110. ^{13}C NMR spectrum for compound **3zz**

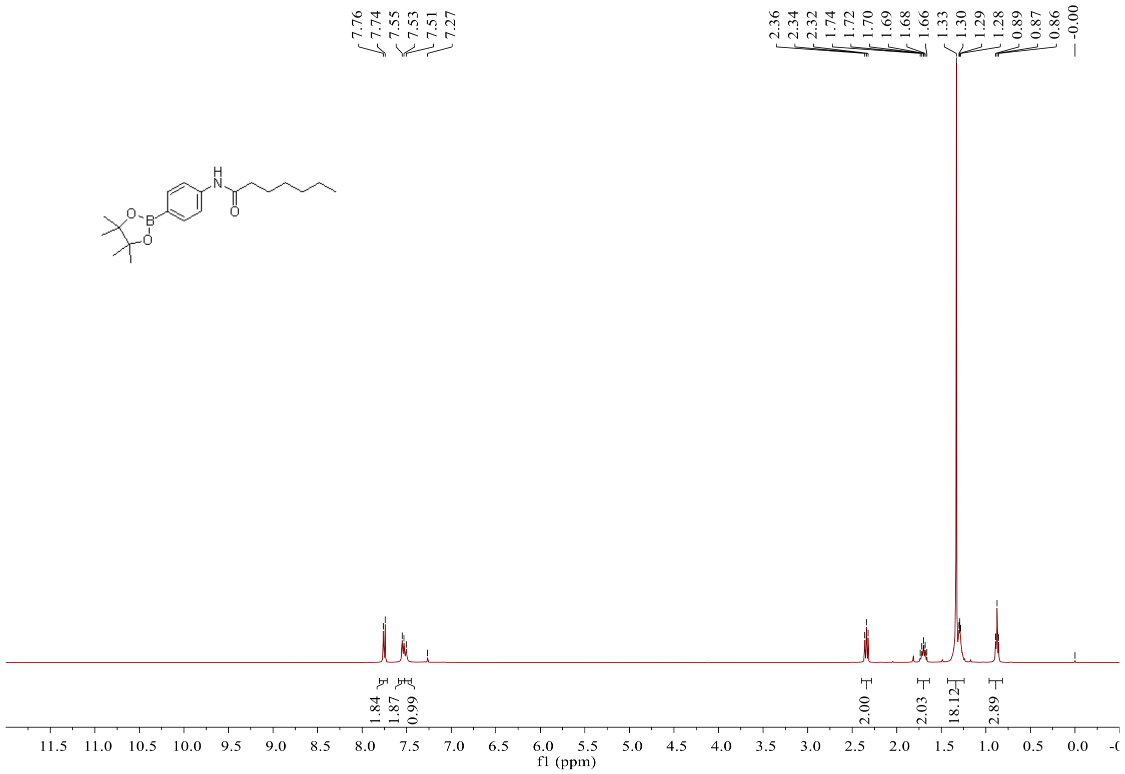

Supplementary Figure 111. ^{1}H NMR spectrum for compound **3ab**

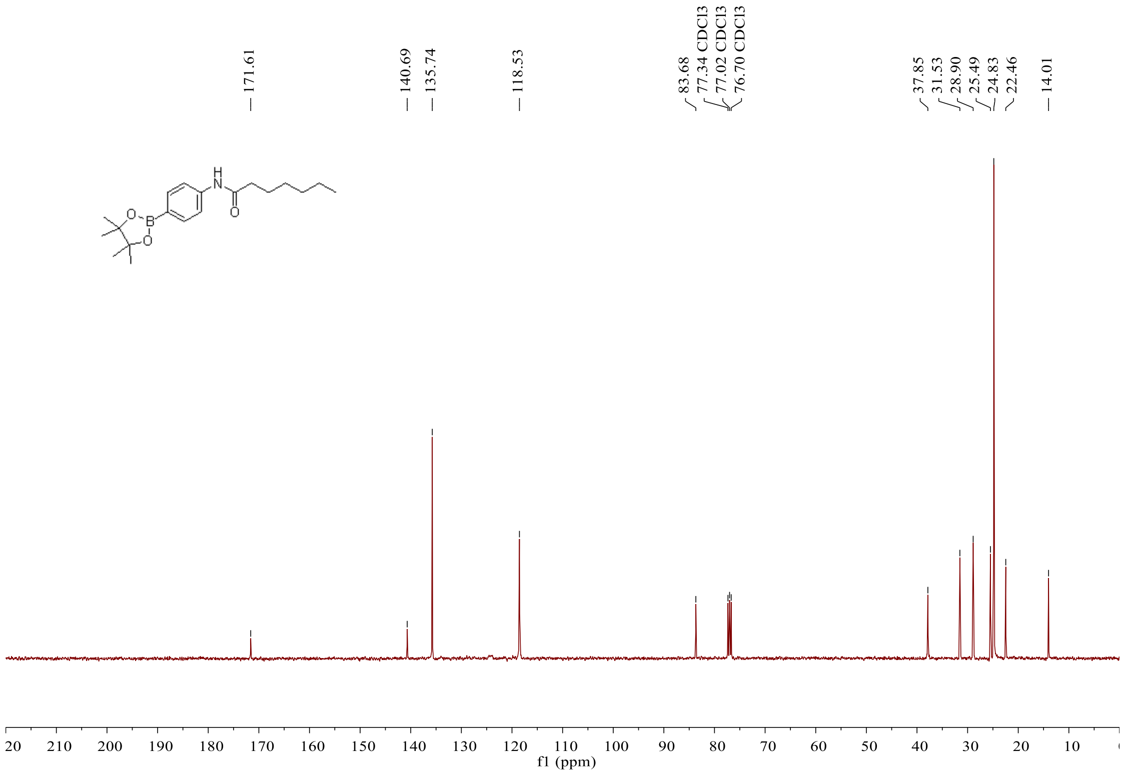

Supplementary Figure 112. ^{13}C NMR spectrum for compound **3ab**

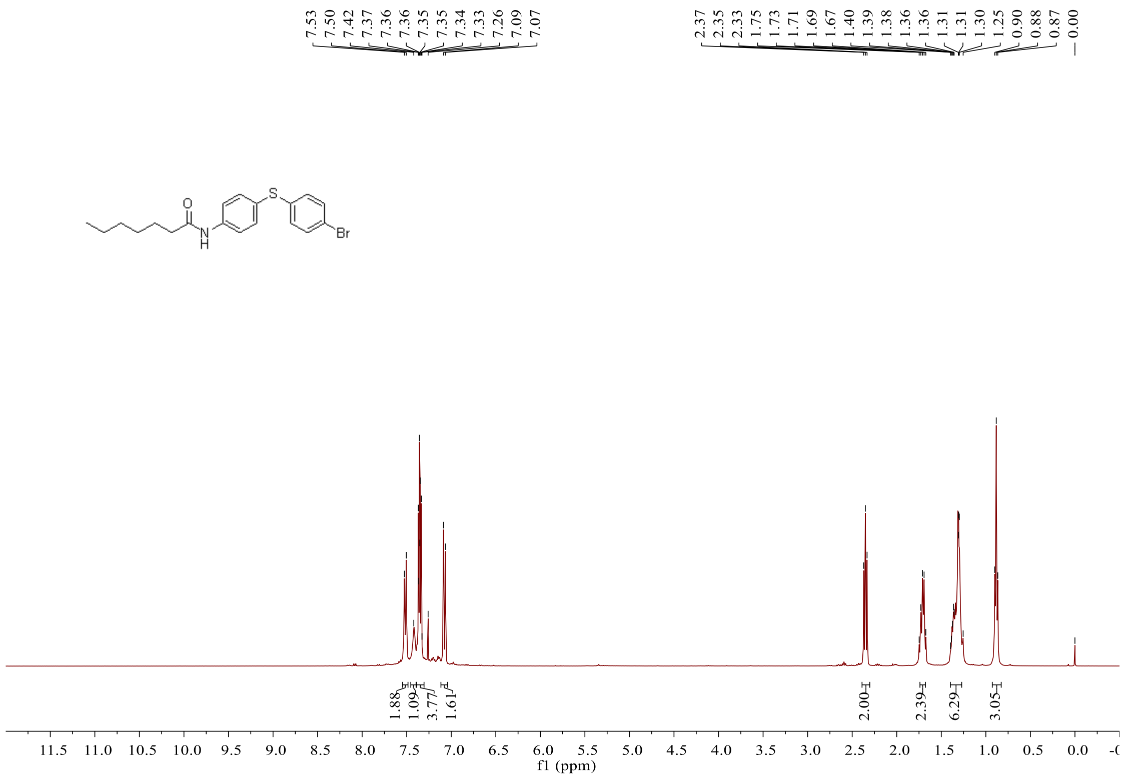

Supplementary Figure 113. ^{1}H NMR spectrum for compound **3ac**

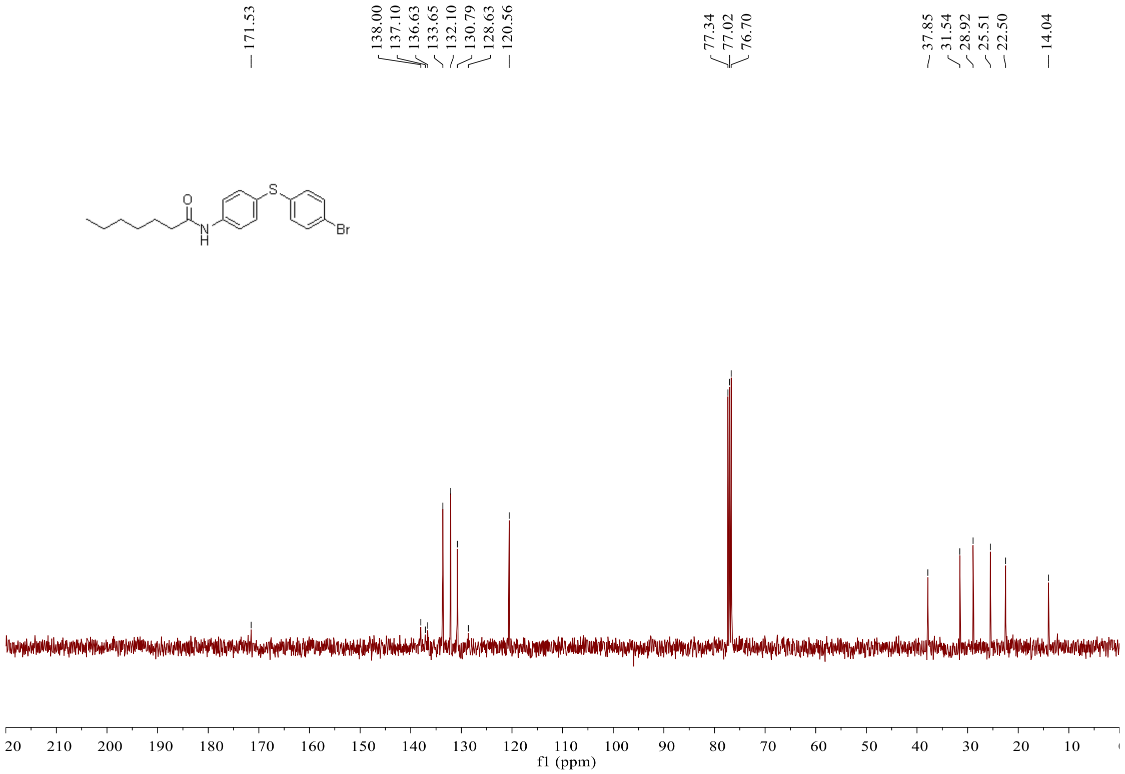

Supplementary Figure 114. ^{13}C NMR spectrum for compound **3ac**

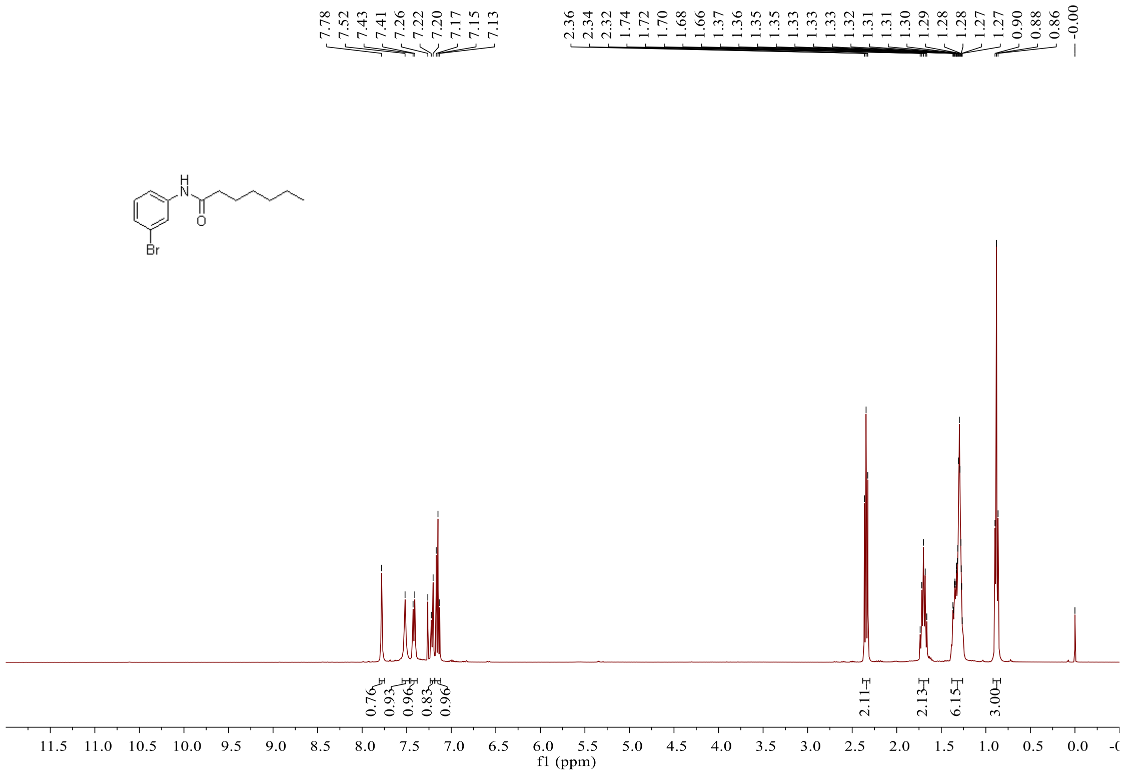

Supplementary Figure 115. ^{1}H NMR spectrum for compound **3ad**

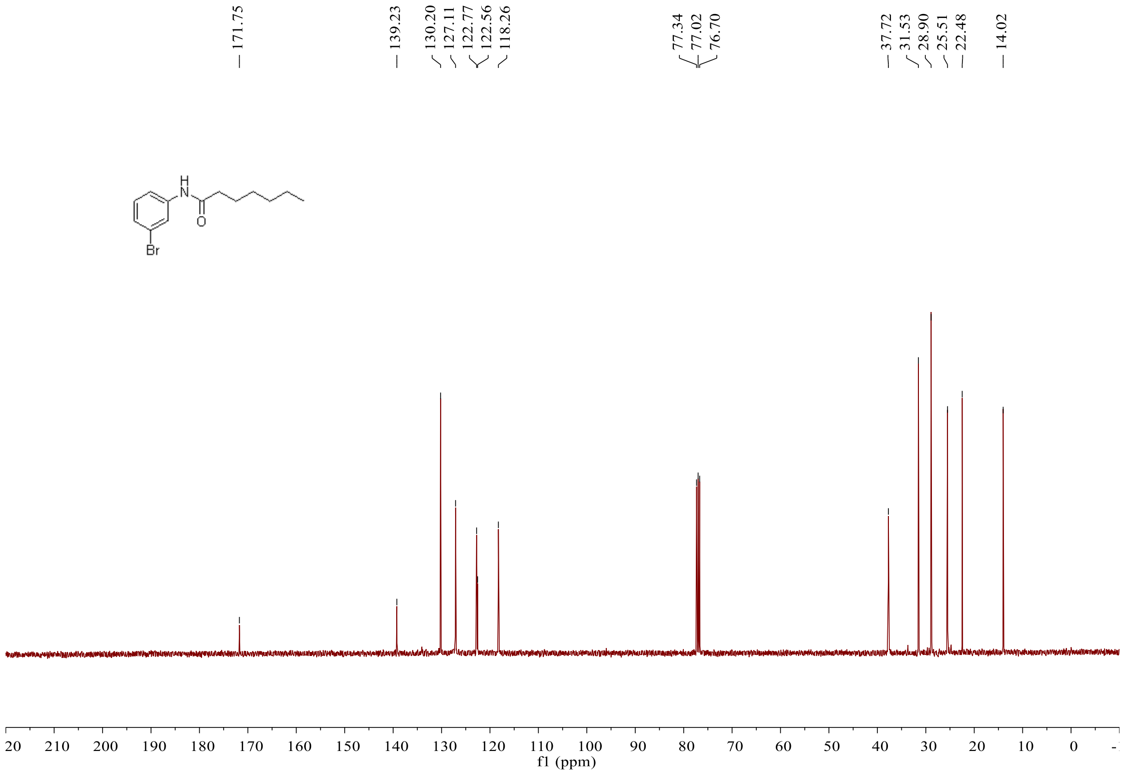

Supplementary Figure 116. ^{13}C NMR spectrum for compound **3ad**

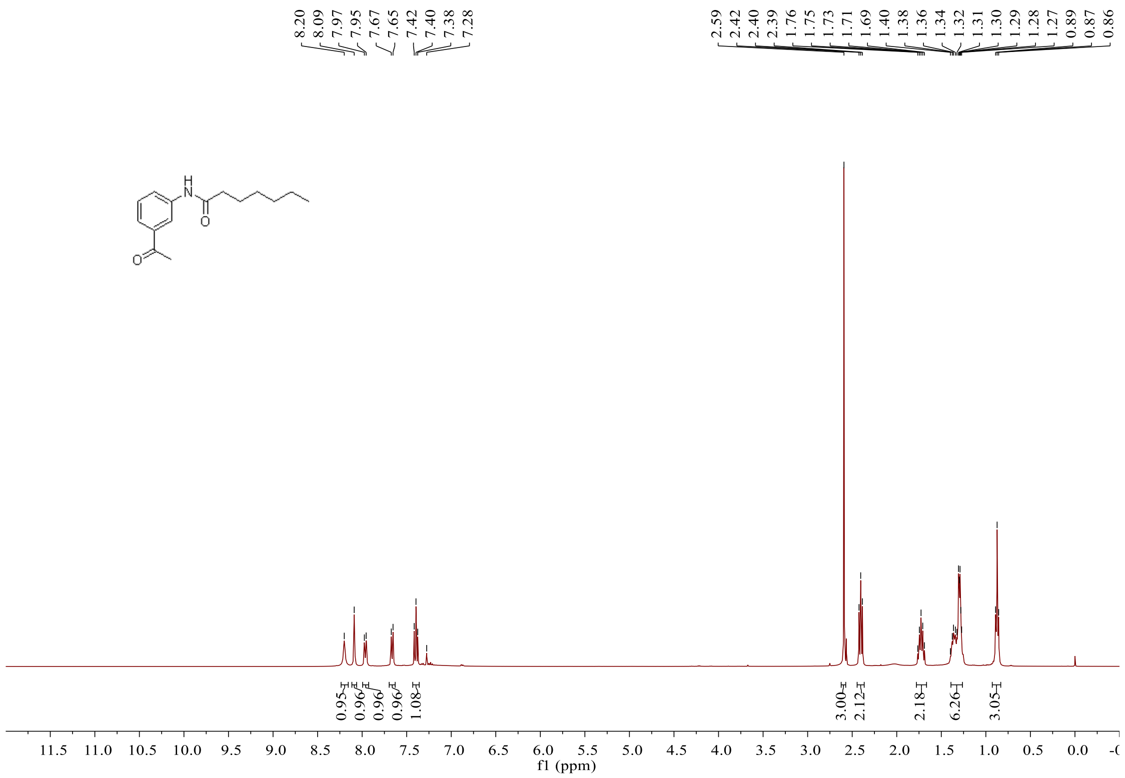

Supplementary Figure 117. ^{1}H NMR spectrum for compound **3ae**

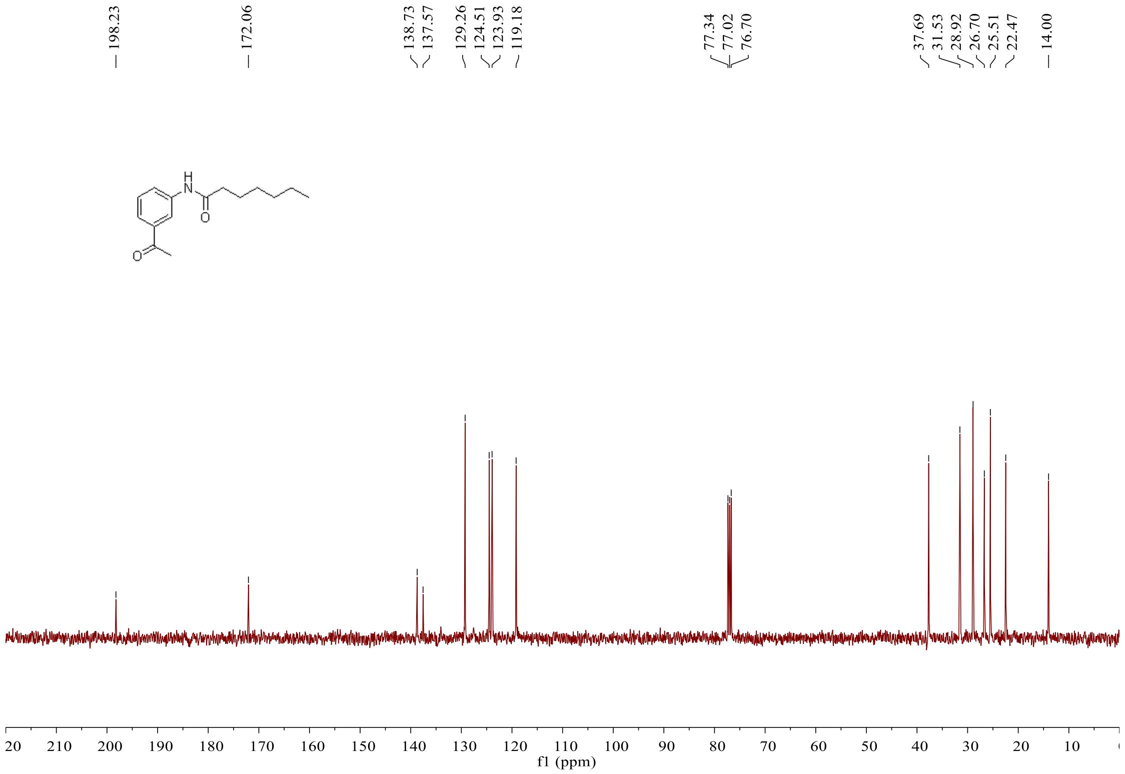

Supplementary Figure 118. ^{13}C NMR spectrum for compound **3ae**

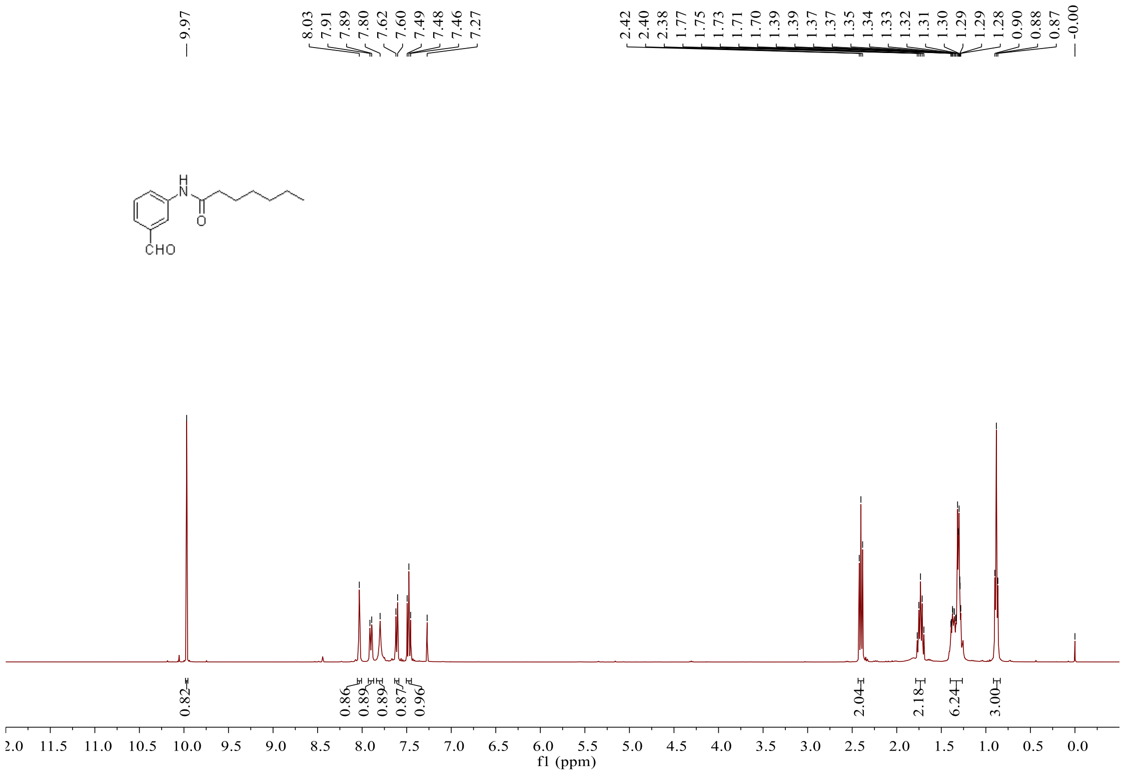

Supplementary Figure 119. ^{1}H NMR spectrum for compound **3af**

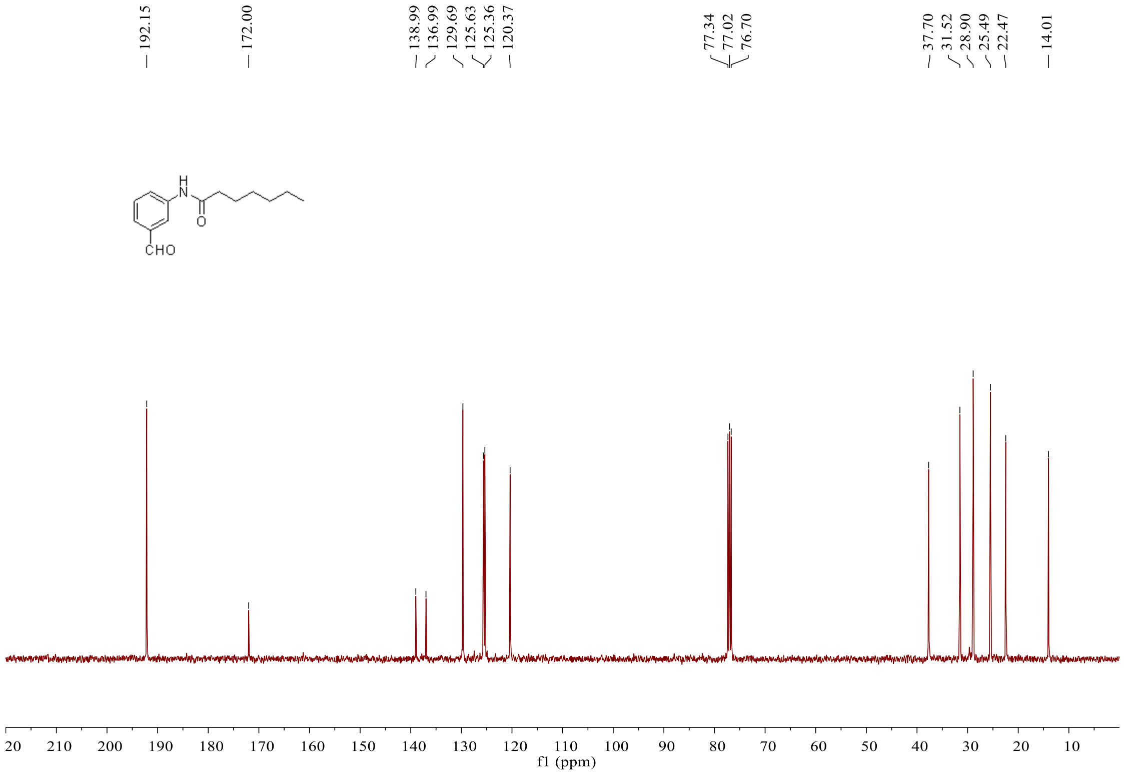

Supplementary Figure 120. ^{13}C NMR spectrum for compound **3af**

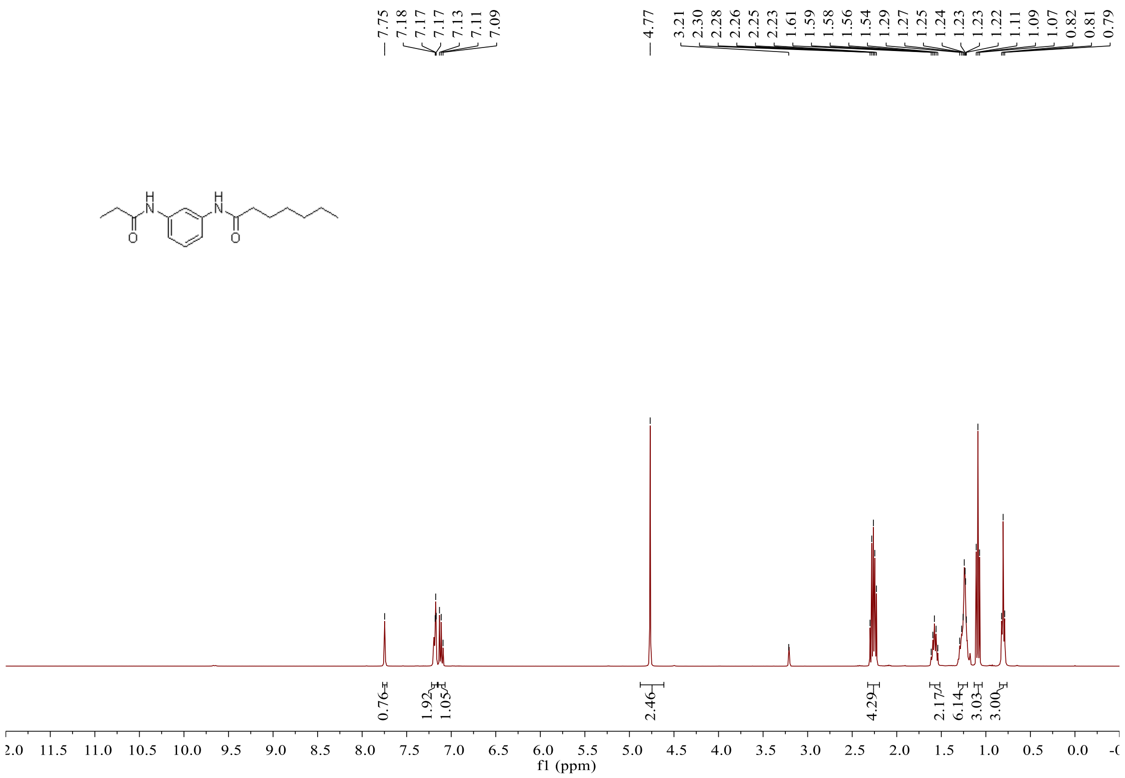

Supplementary Figure 121. ^{1}H NMR spectrum for compound **3ag**

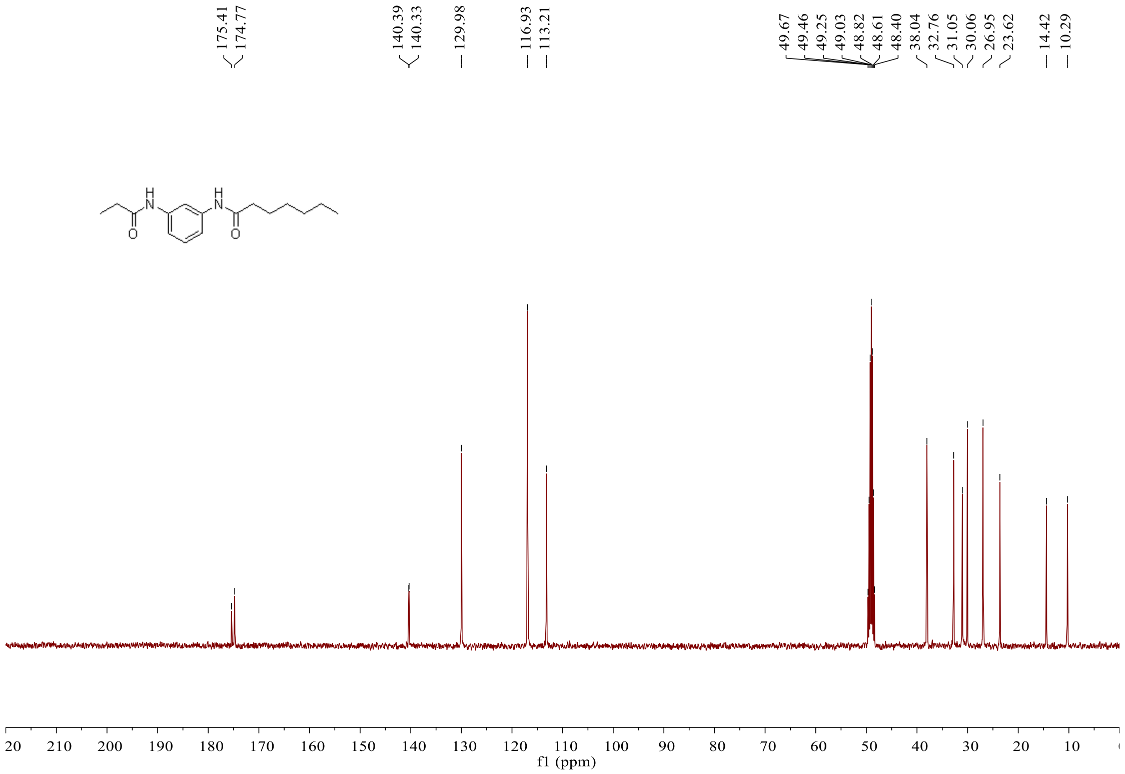

Supplementary Figure 122. ^{13}C NMR spectrum for compound **3ag**

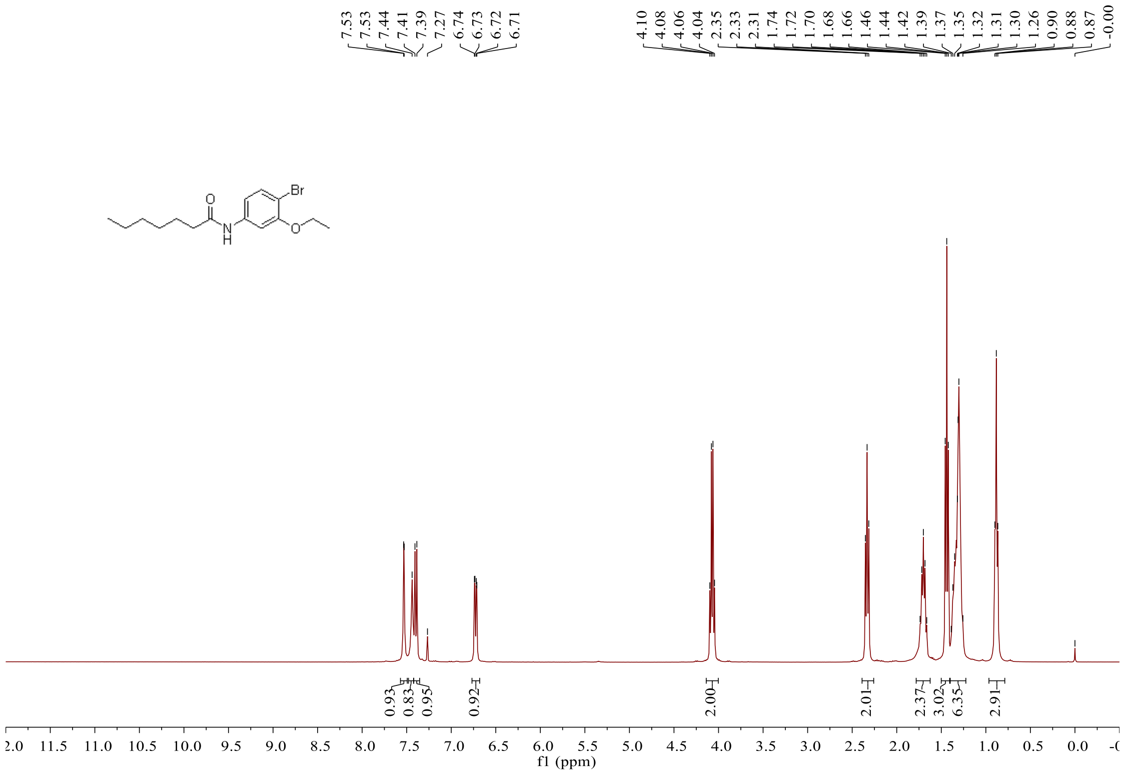

Supplementary Figure 123. ^{1}H NMR spectrum for compound **3ah**

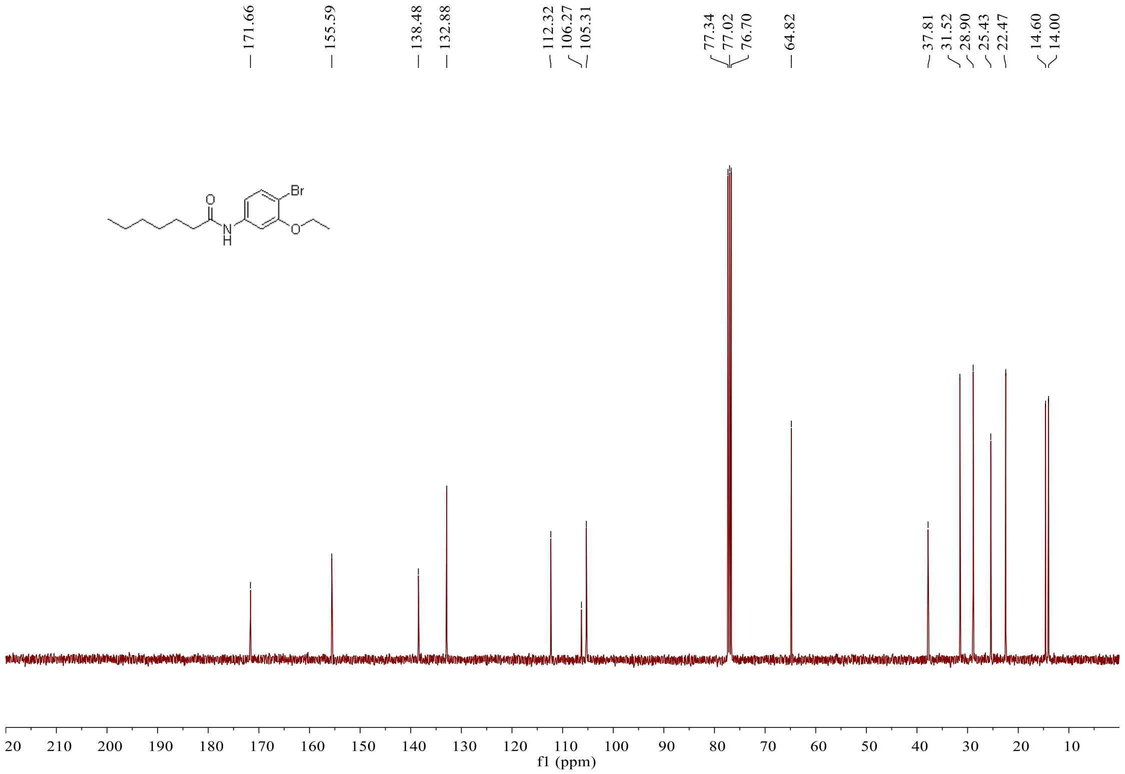

Supplementary Figure 124. ^{13}C NMR spectrum for compound **3ah**

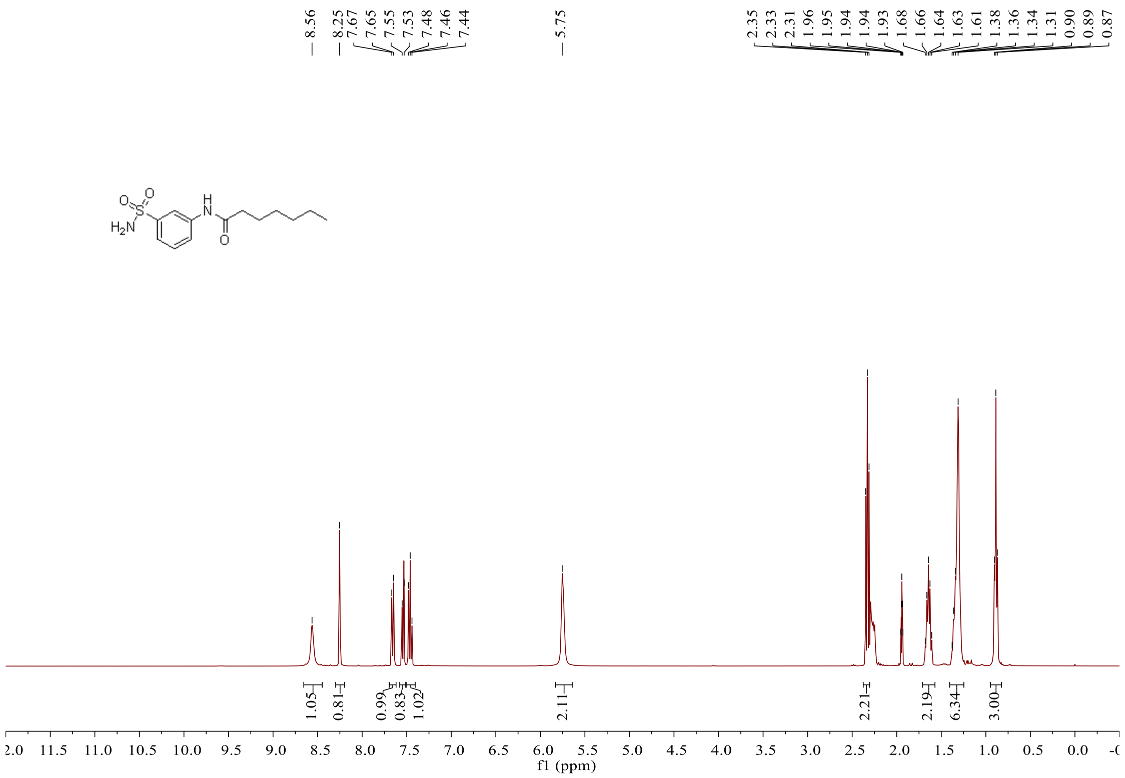

Supplementary Figure 125. ^{1}H NMR spectrum for compound **3ai**

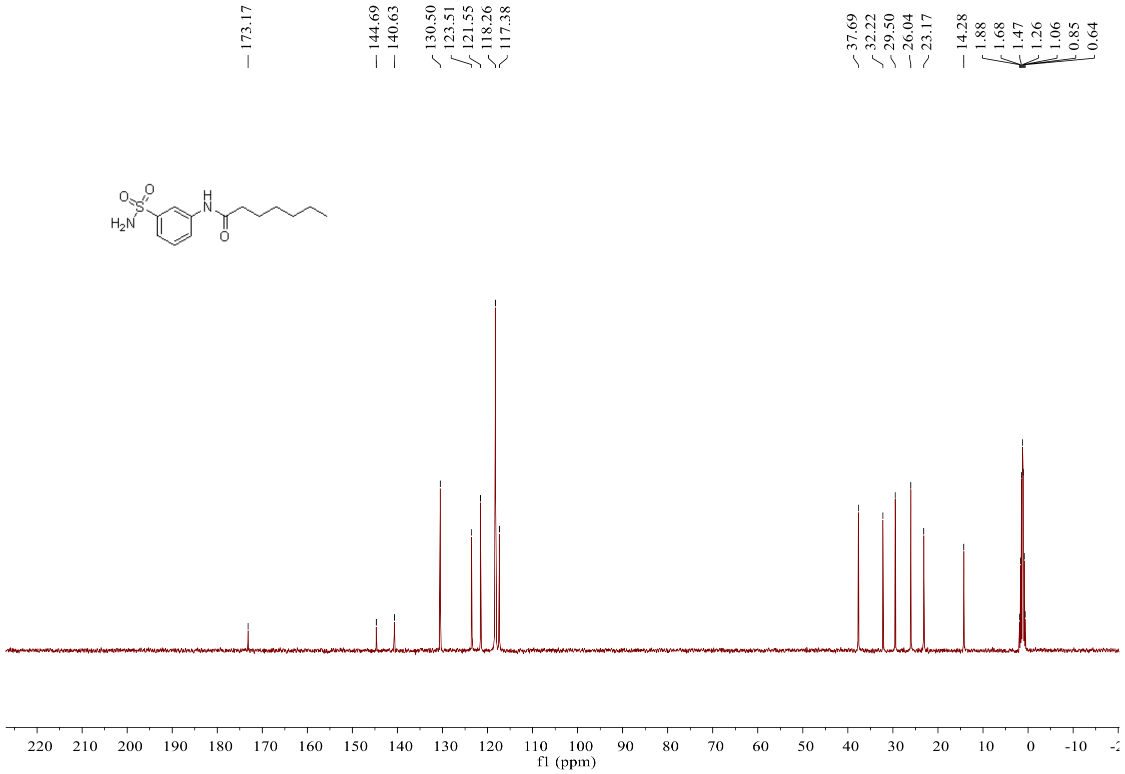

Supplementary Figure 126. ^{13}C NMR spectrum for compound **3ai**

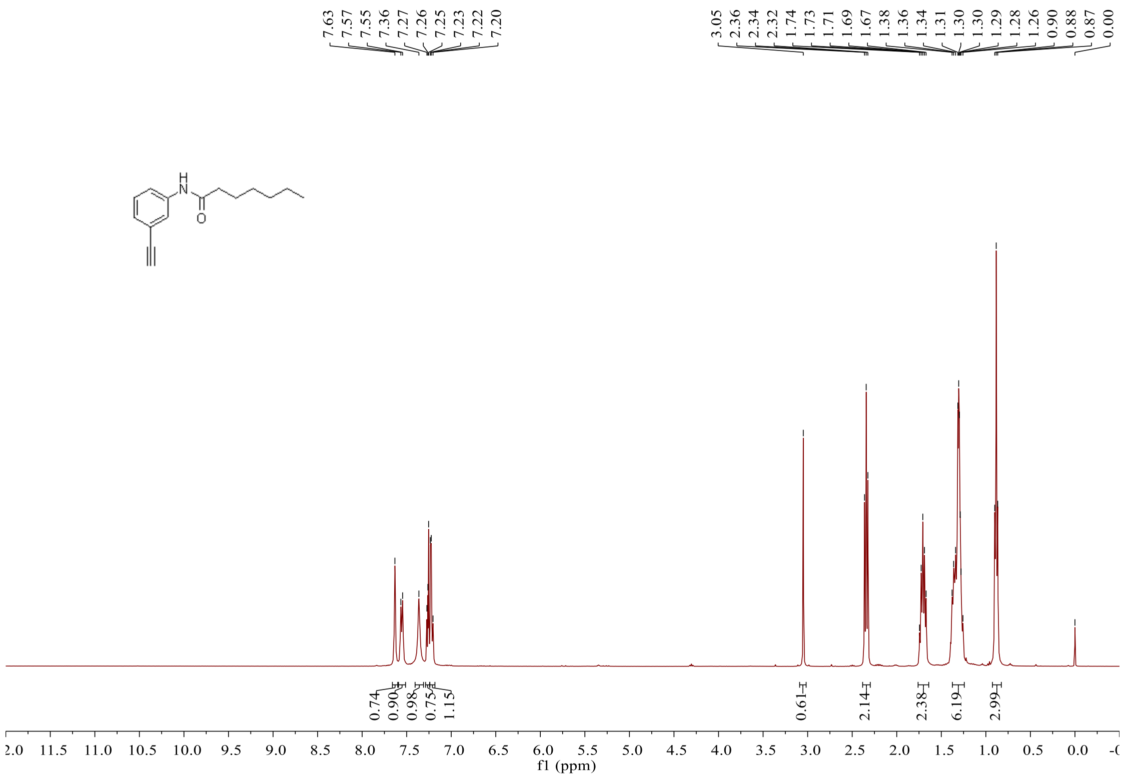

Supplementary Figure 127. ^{1}H NMR spectrum for compound **3aj**

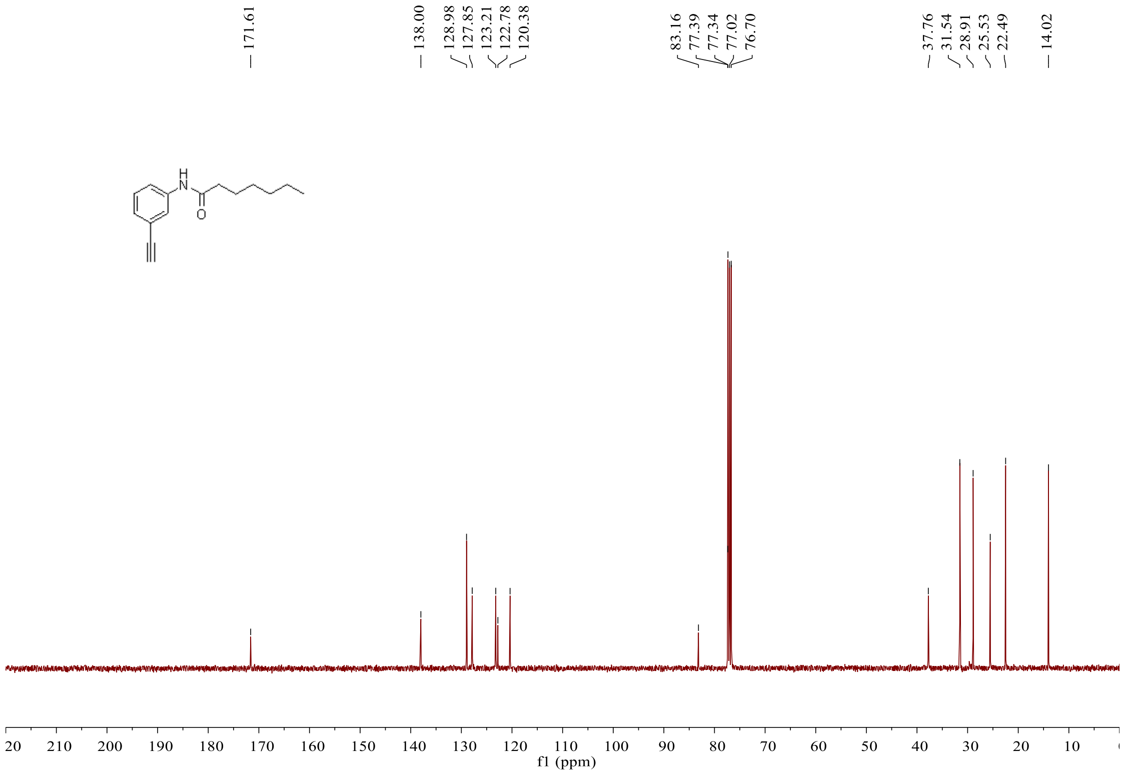

Supplementary Figure 128. ^{13}C NMR spectrum for compound **3aj**

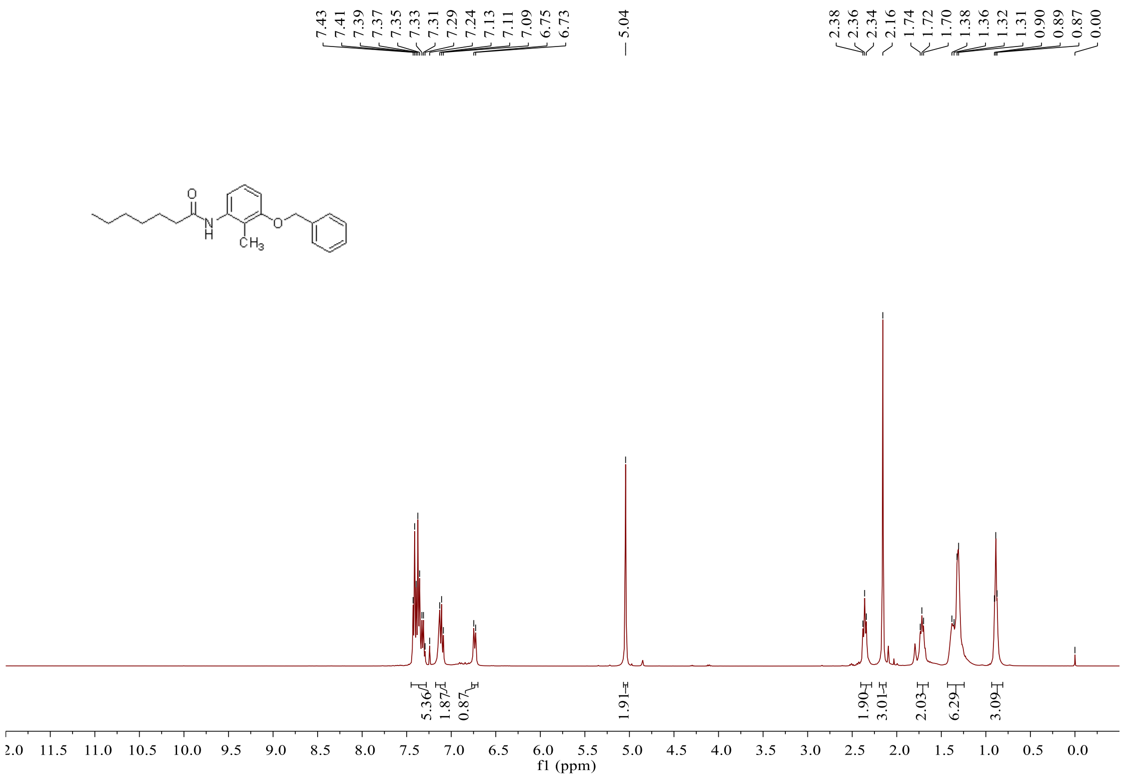

Supplementary Figure 129. ^{1}H NMR spectrum for compound **3ak**

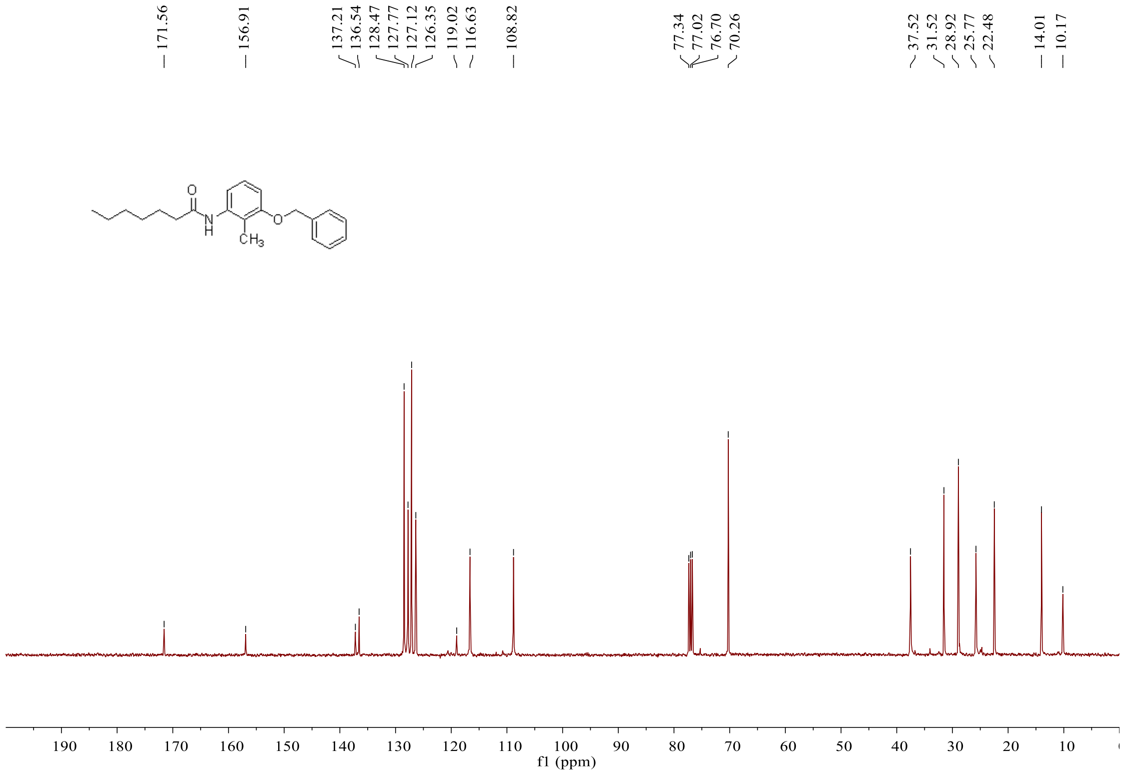

Supplementary Figure 130. ^{13}C NMR spectrum for compound **3ak**

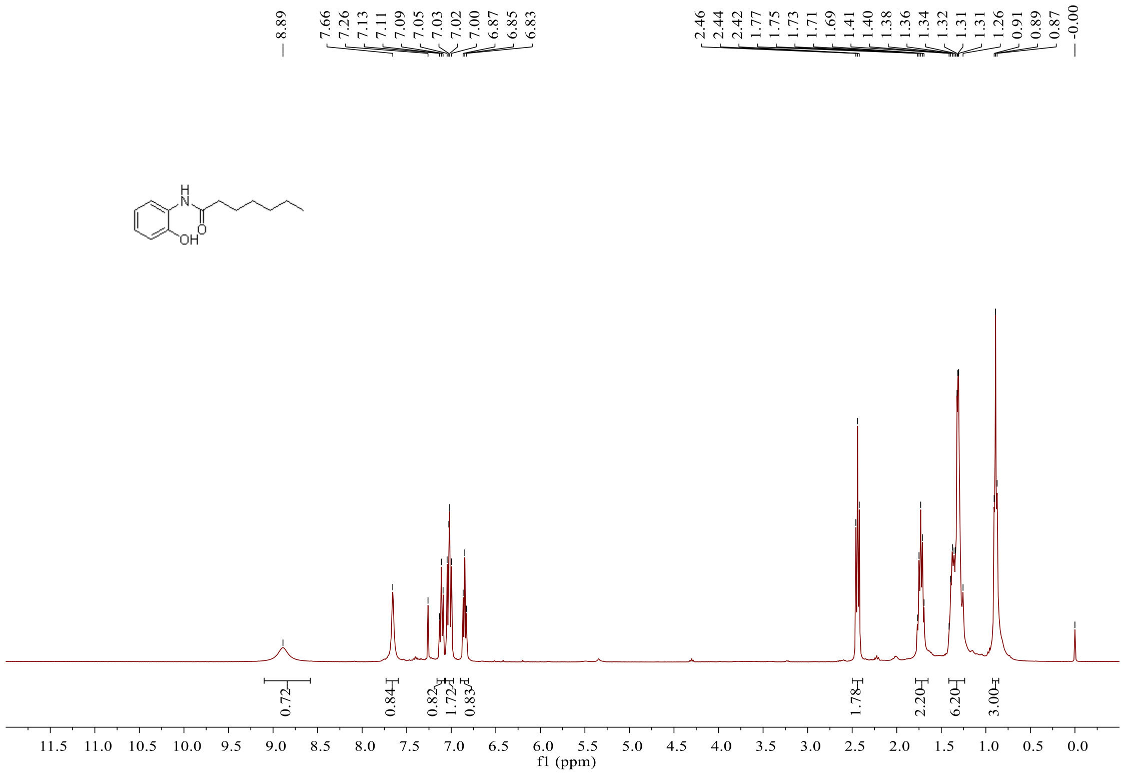

Supplementary Figure 131. ^{1}H NMR spectrum for compound **3al**

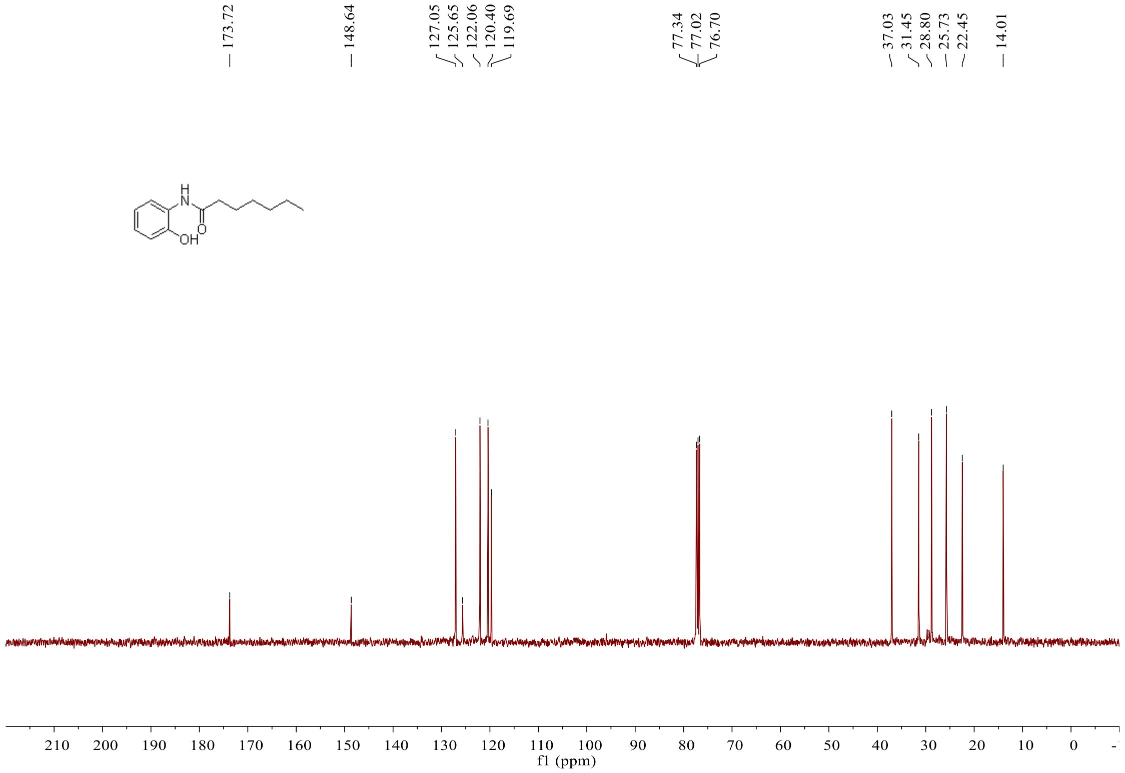

Supplementary Figure 132. ^{13}C NMR spectrum for compound **3al**

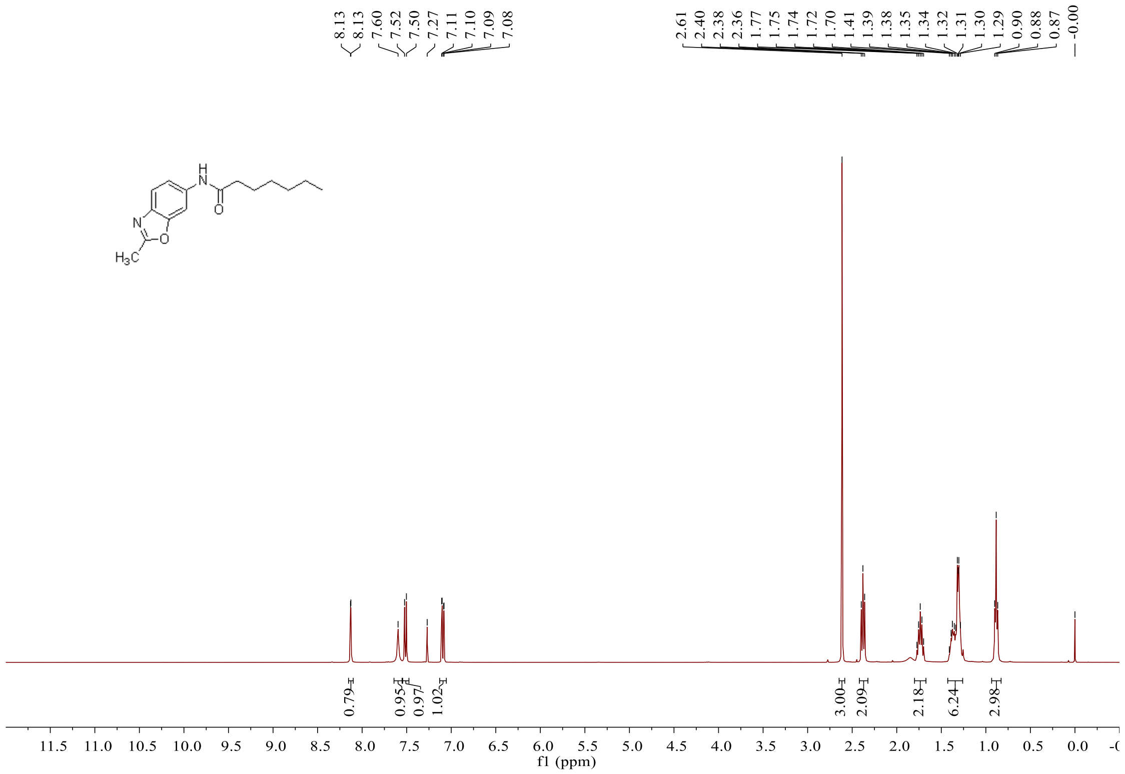

Supplementary Figure 133. ^{1}H NMR spectrum for compound **3am**

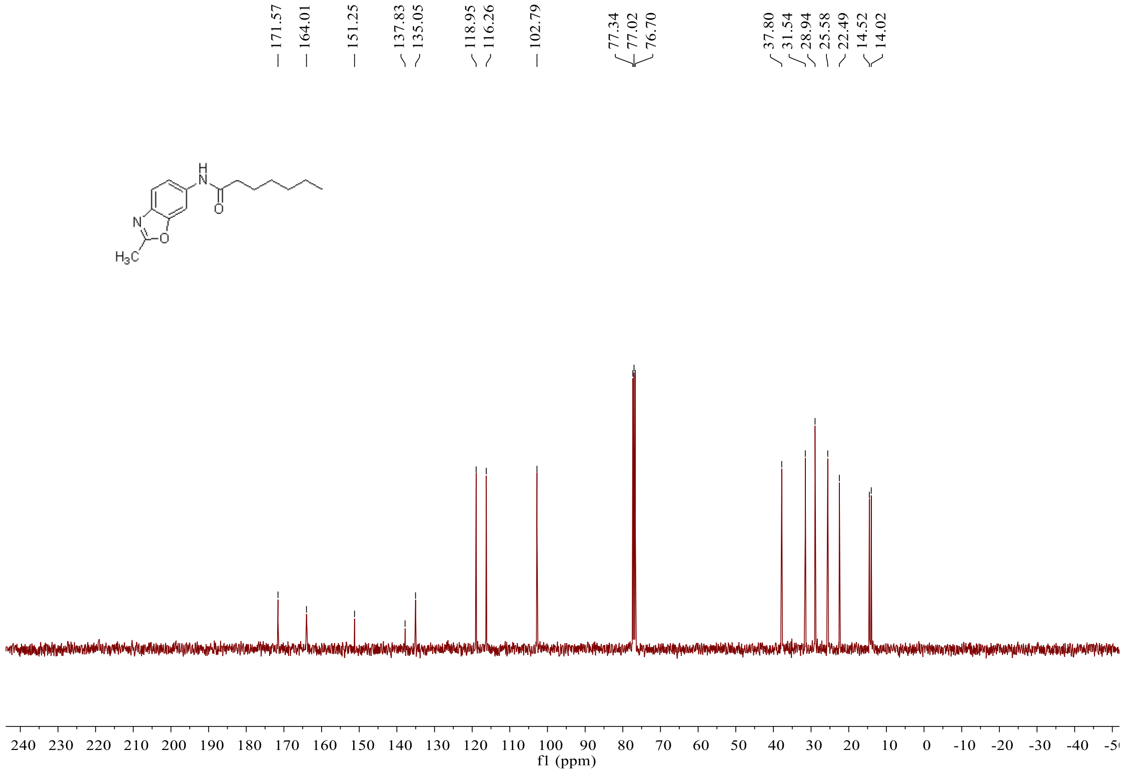

Supplementary Figure 134. ^{13}C NMR spectrum for compound **3am**

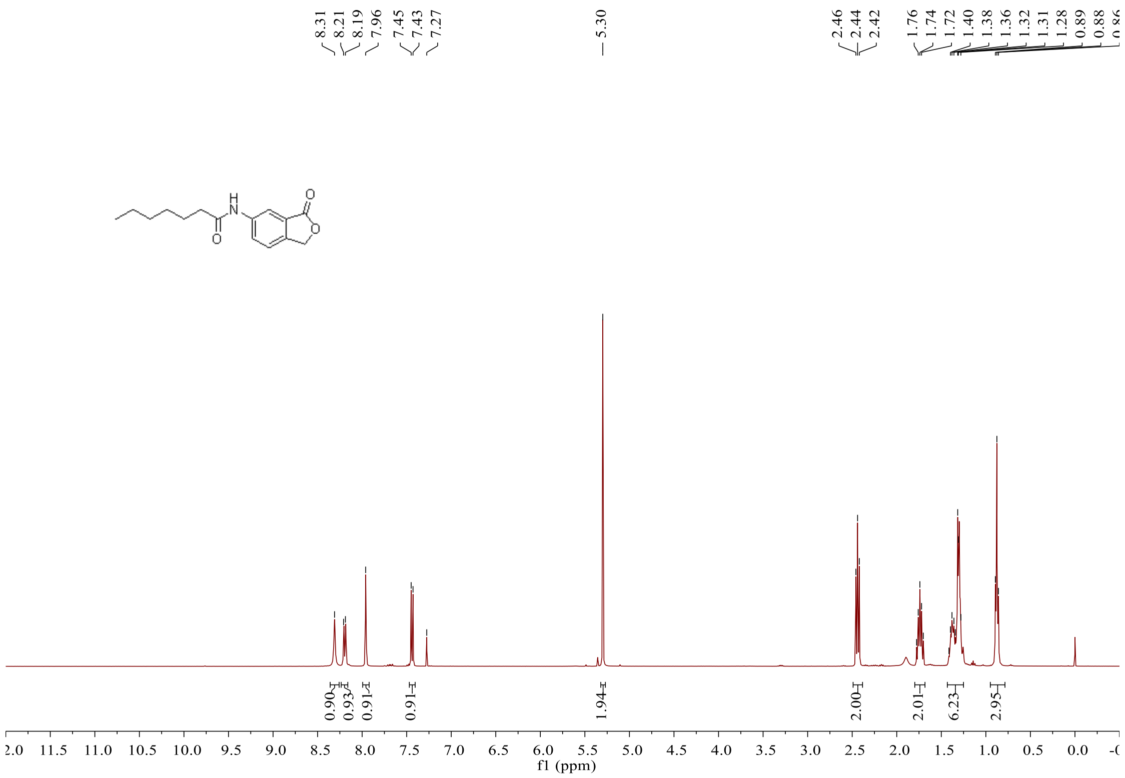

Supplementary Figure 135. ^{1}H NMR spectrum for compound **3an**

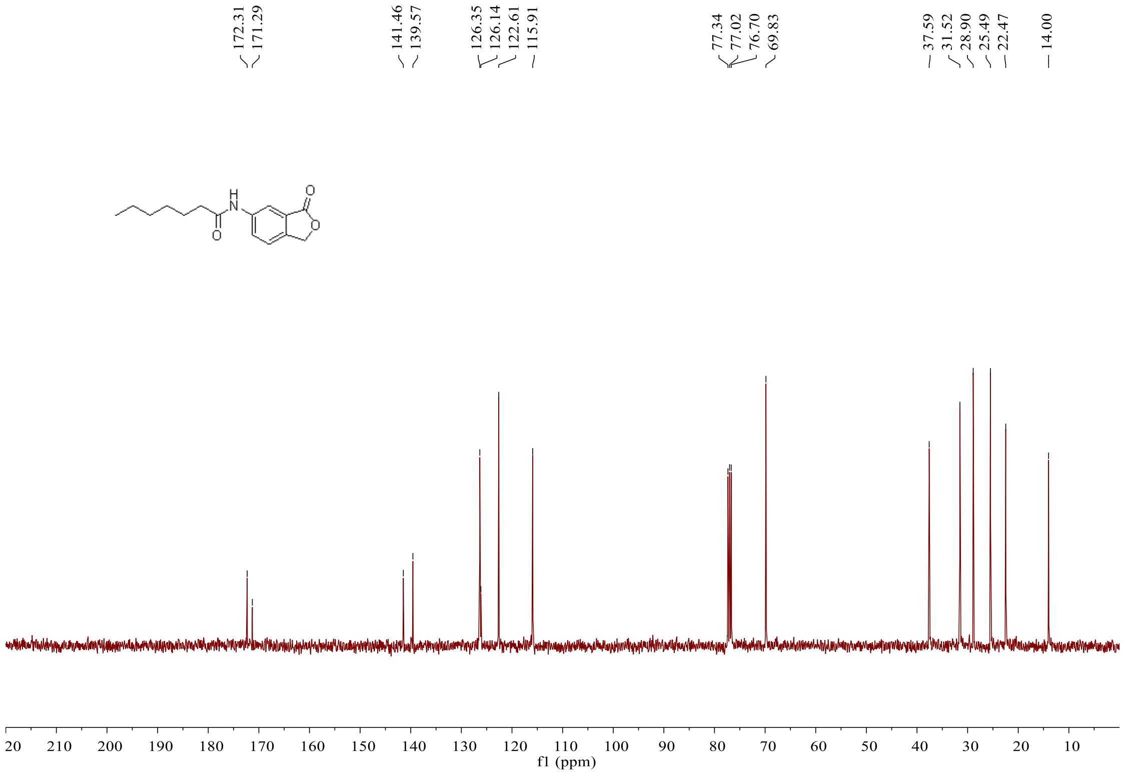

Supplementary Figure 136. ^{13}C NMR spectrum spectra for compound **3an**

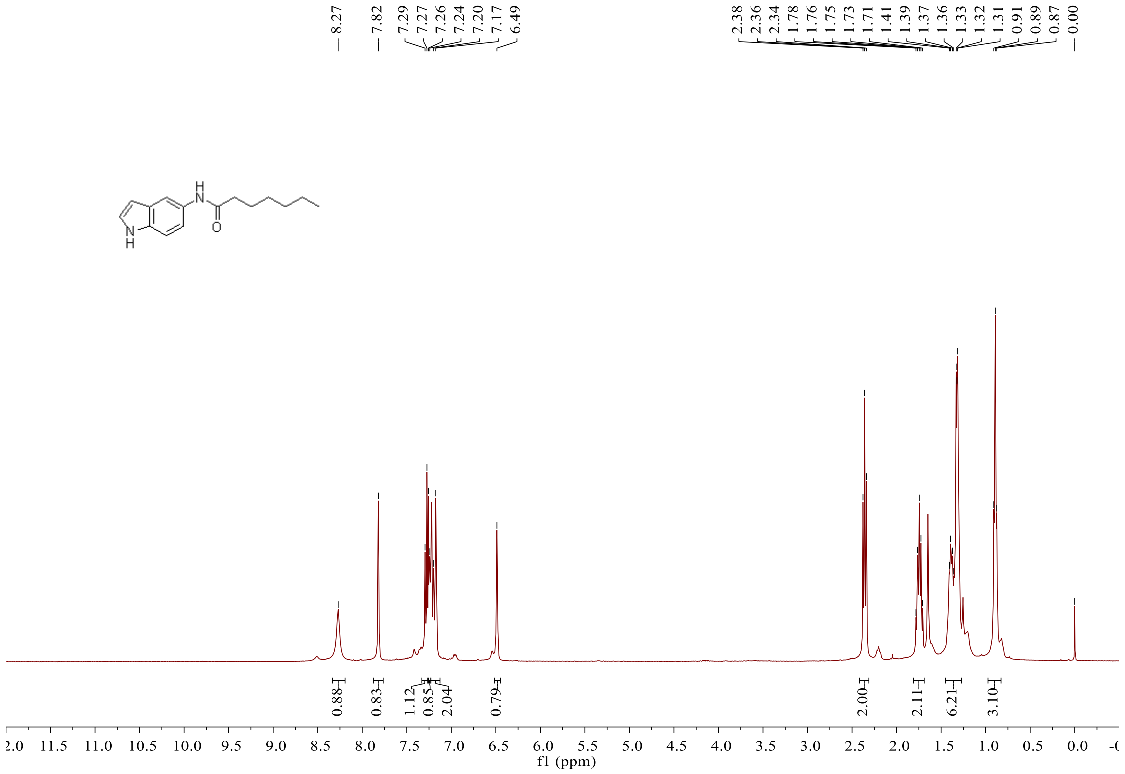

Supplementary Figure 137. ^{1}H NMR spectrum for compound **3ao**

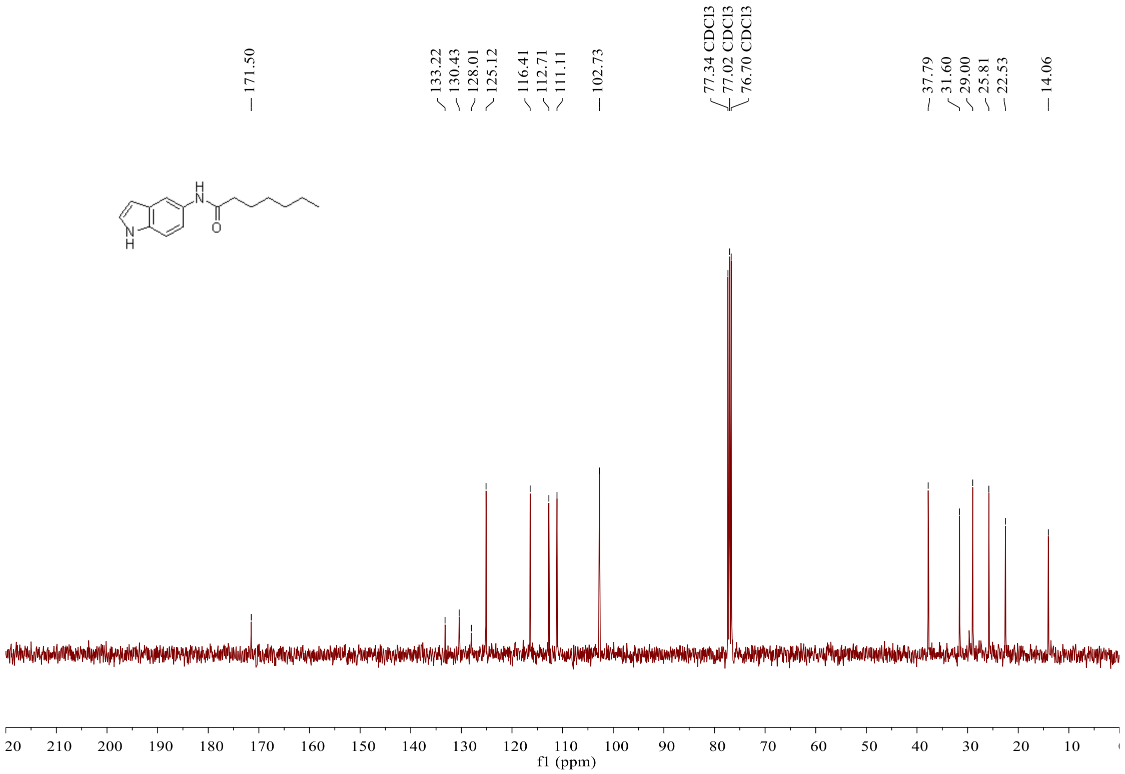

Supplementary Figure 138. ^{13}C NMR spectrum for compound **3ao**

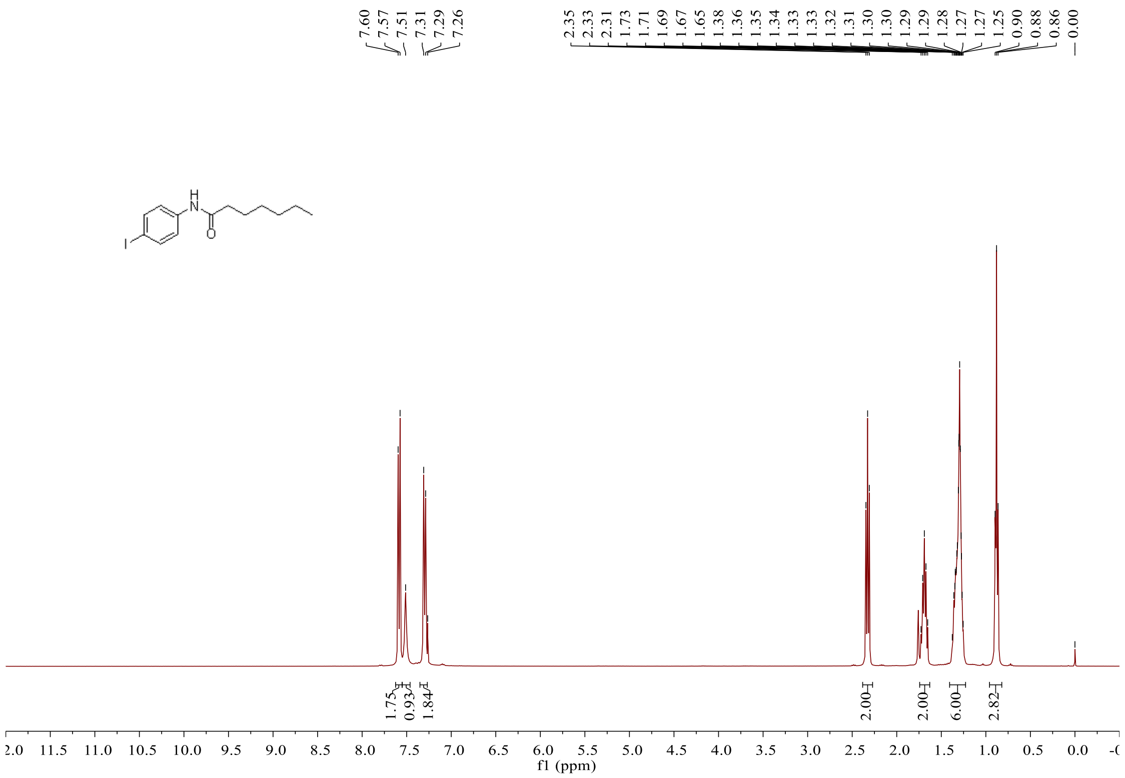

Supplementary Figure 139. ^{1}H NMR spectrum for compound **3ap**

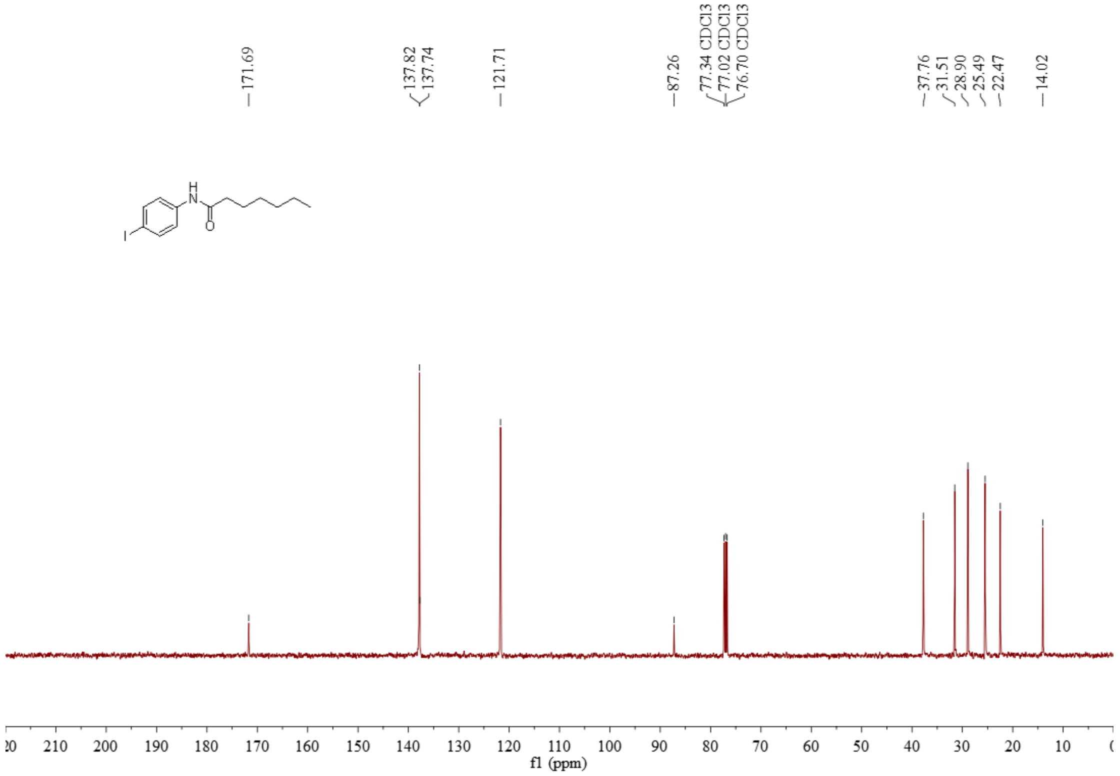

Supplementary Figure 140. ^{13}C NMR spectrum for compound **3ap**

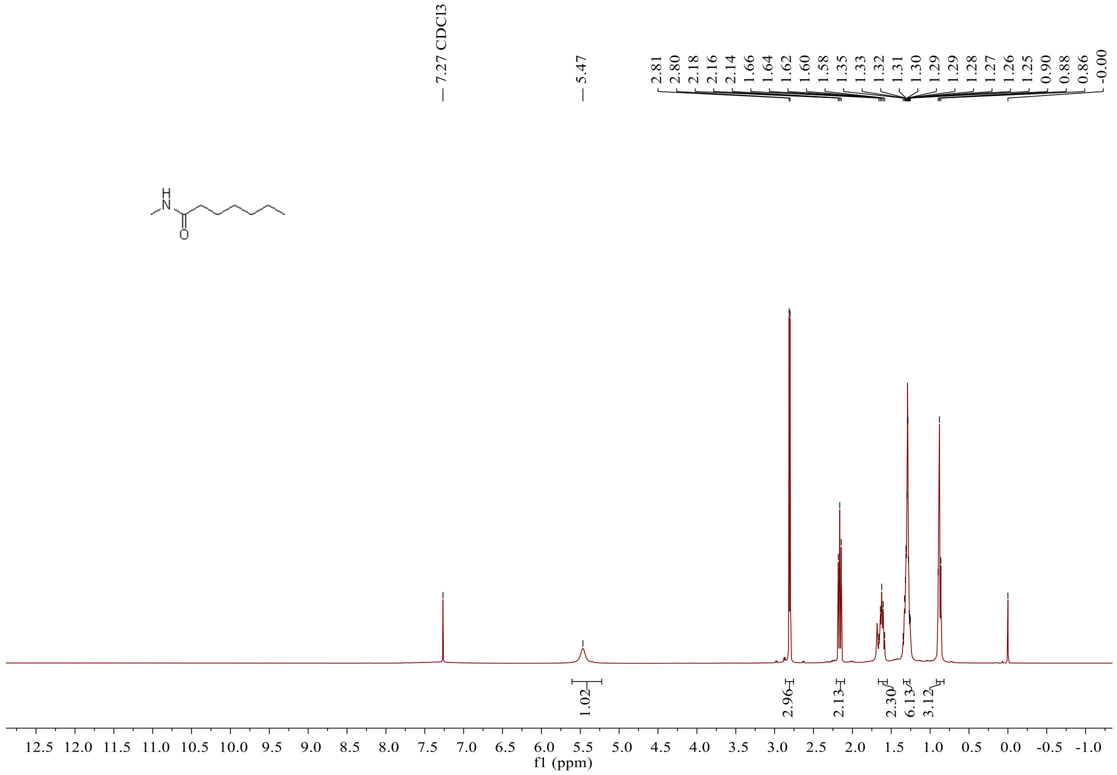

Supplementary Figure 141. ^{1}H NMR spectrum for compound **3aq**

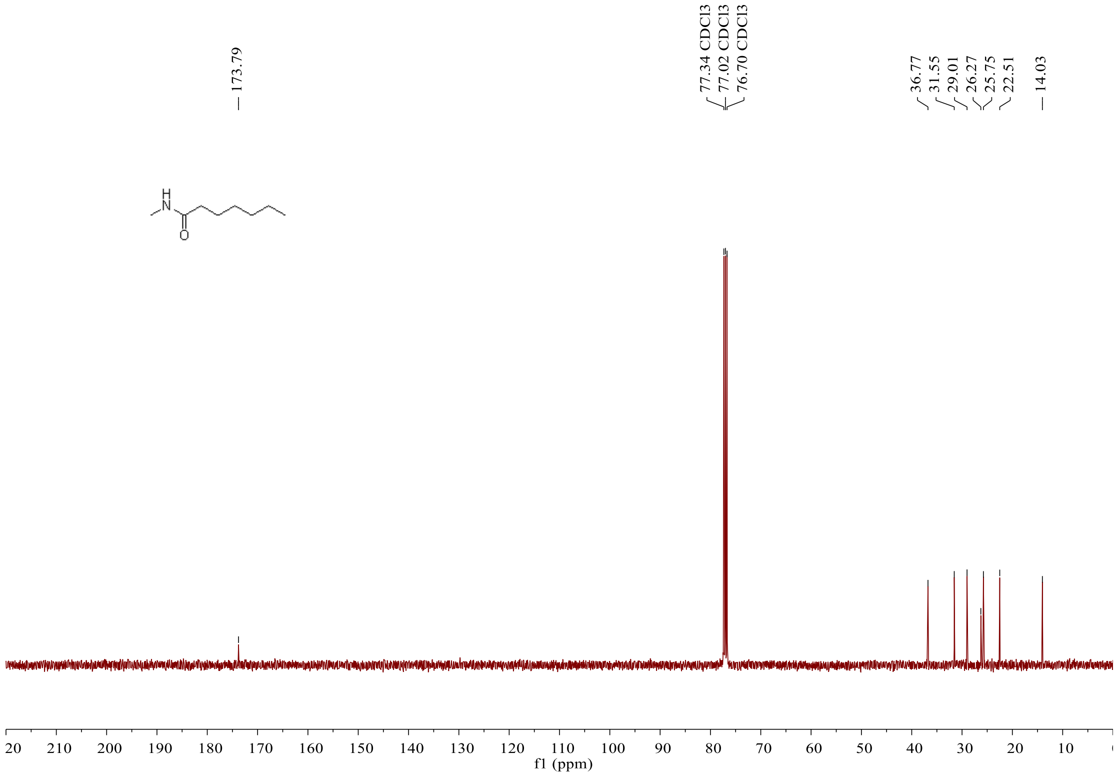

Supplementary Figure 142. ^{13}C NMR spectrum for compound **3aq**

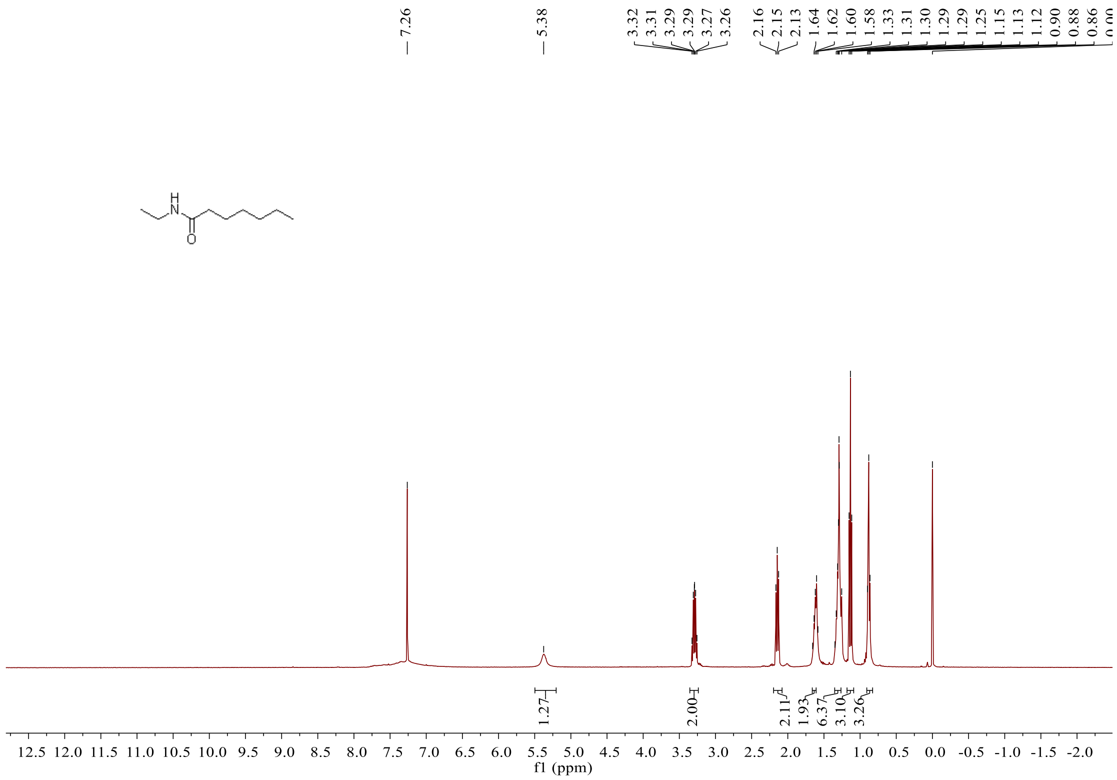

Supplementary Figure 143. ^{1}H NMR spectrum for compound **3ar**

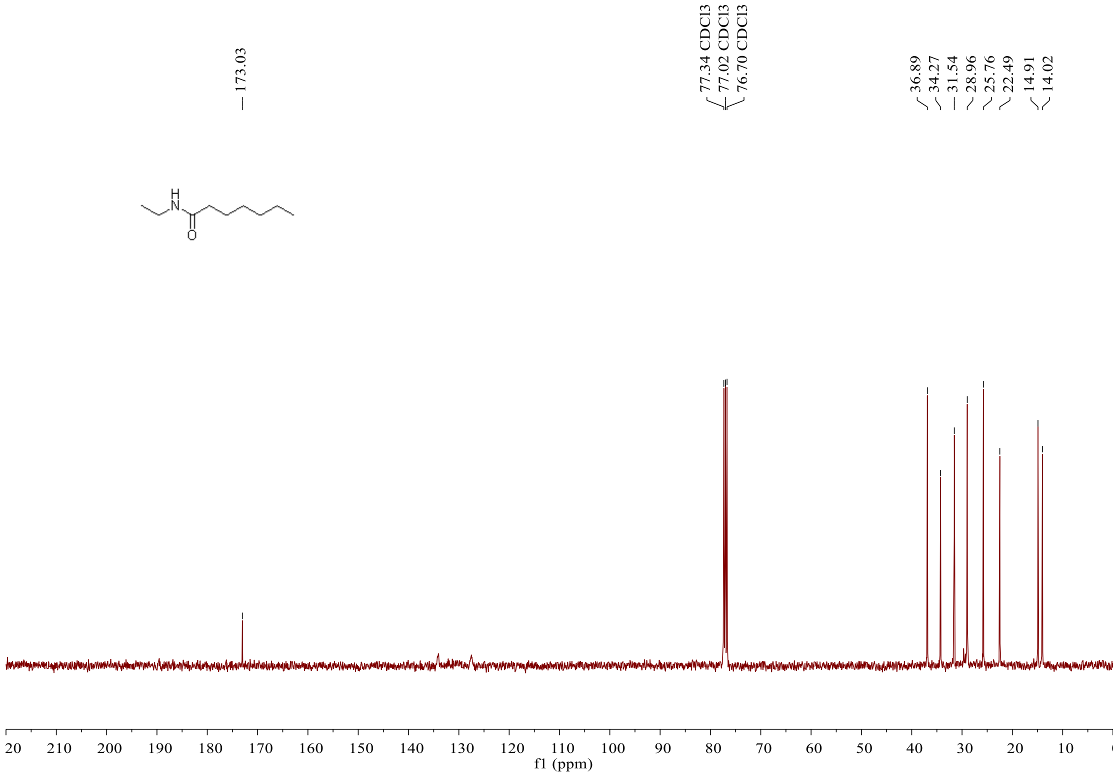

Supplementary Figure 144. ^{13}C NMR spectrum for compound **3ar**

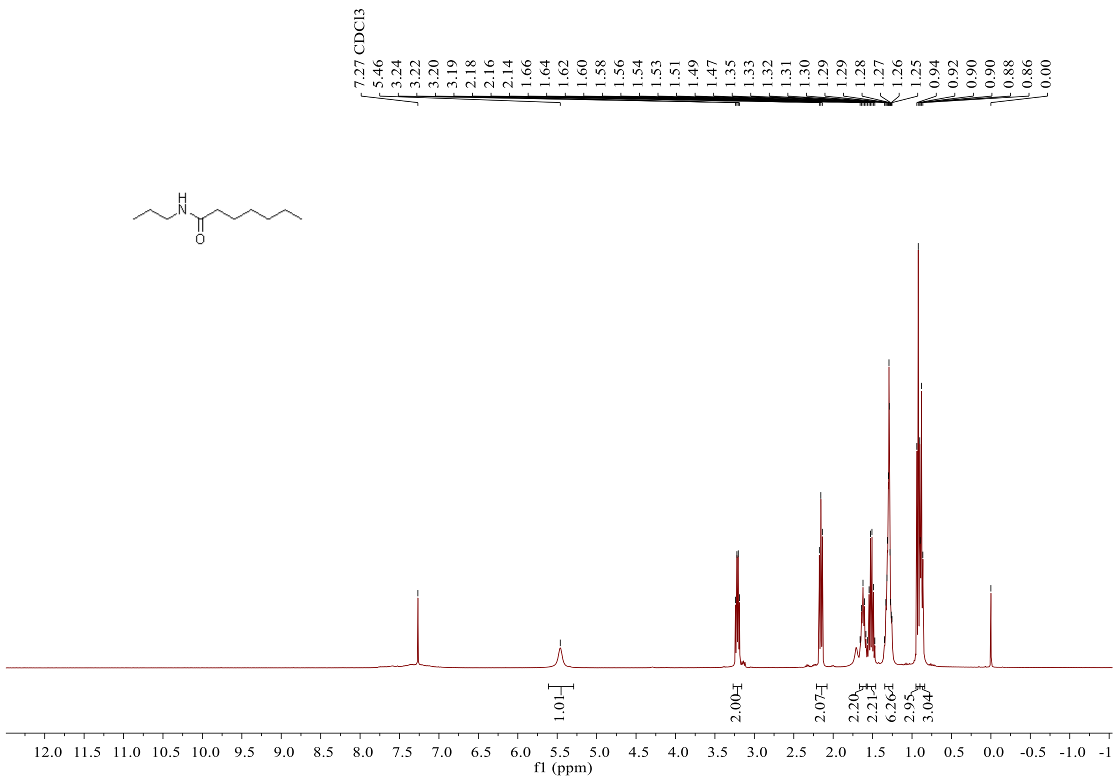

Supplementary Figure 145. ^{1}H NMR spectrum for compound **3as**

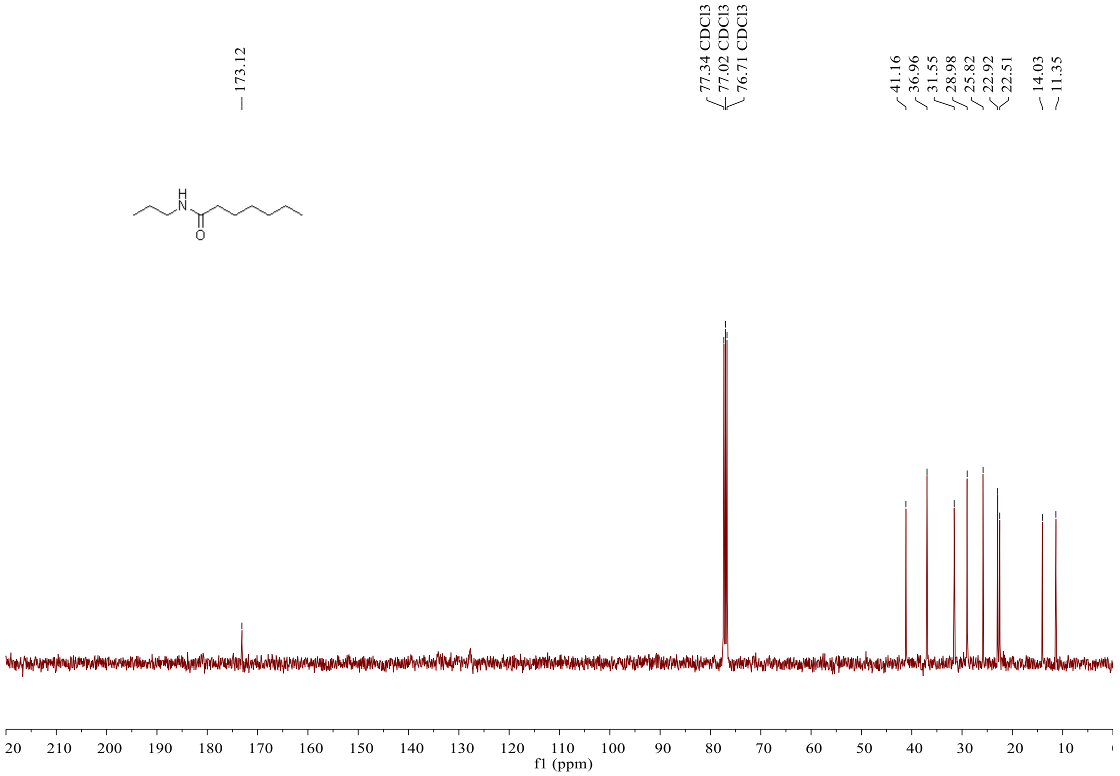

Supplementary Figure 146. ^{13}C NMR spectrum for compound **3as**

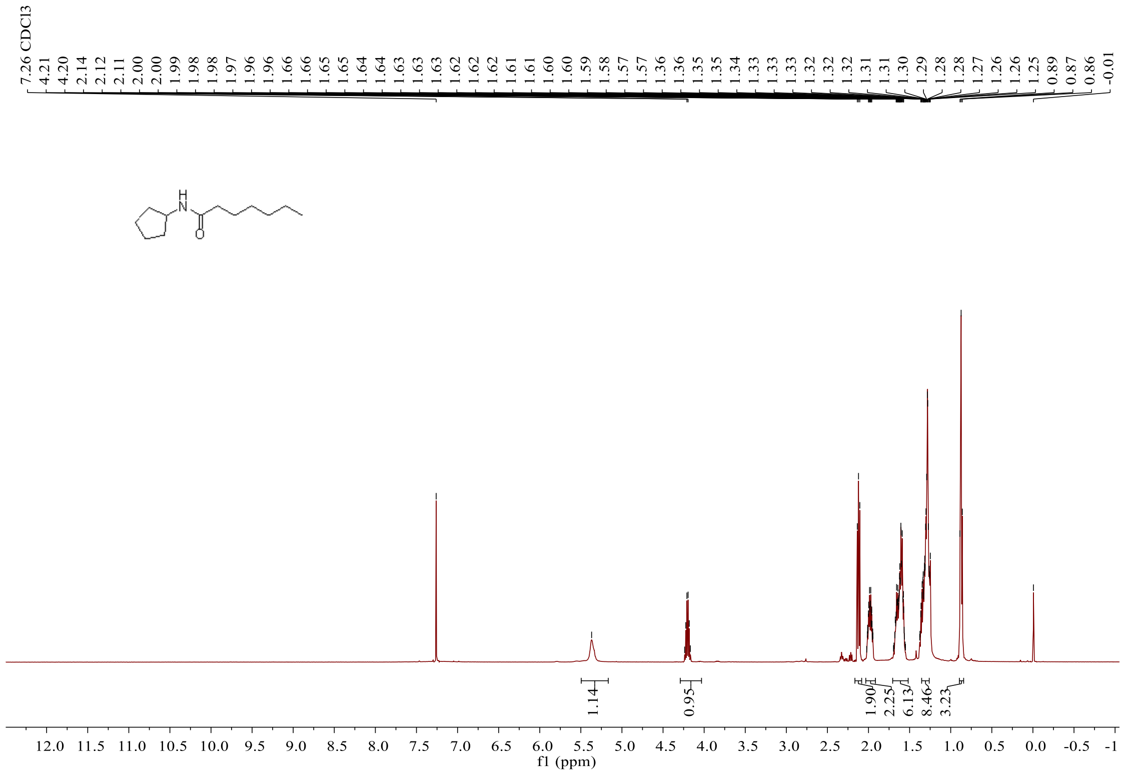

Supplementary Figure 147. ^{1}H NMR spectrum for compound **3at**

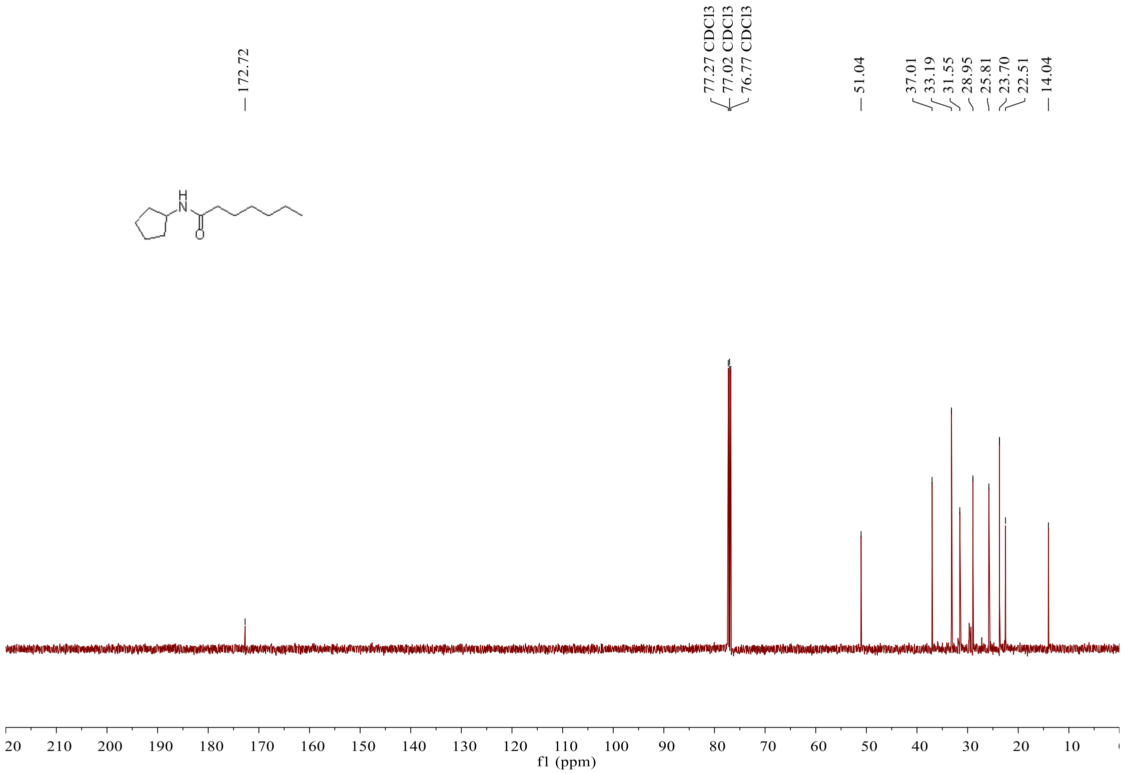

Supplementary Figure 148. ^{13}C NMR spectrum for compound **3at**

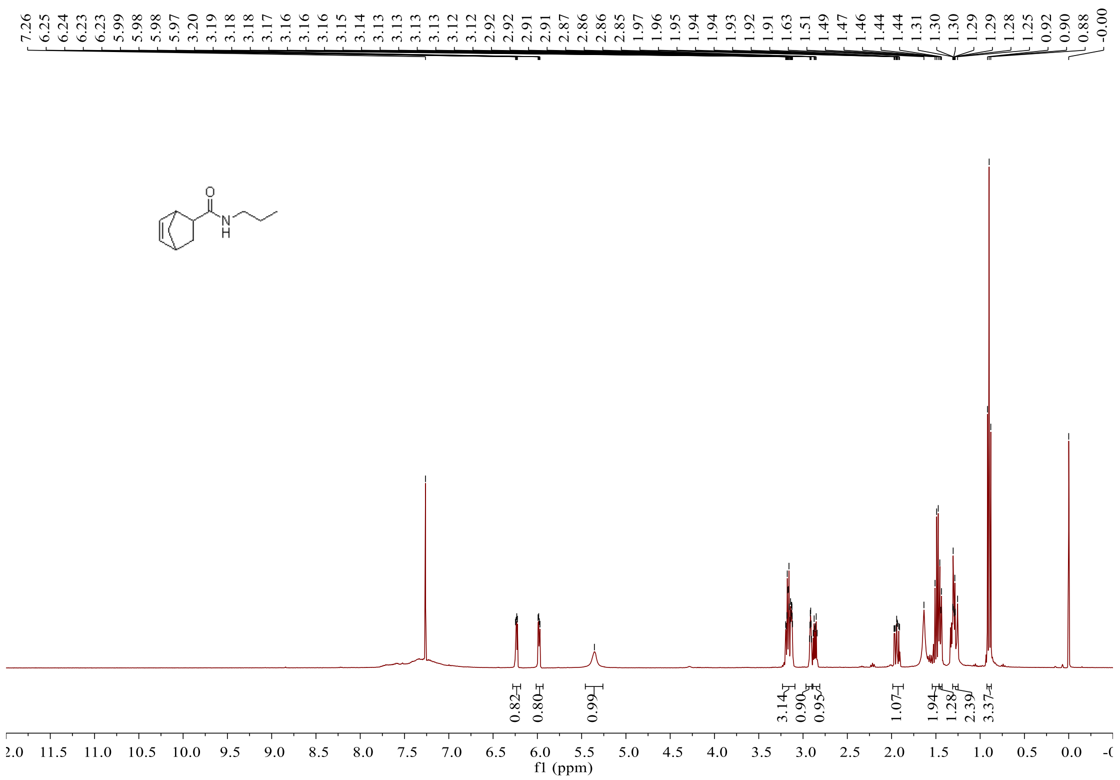

Supplementary Figure 149. ^{1}H NMR spectrum for compound **3au**

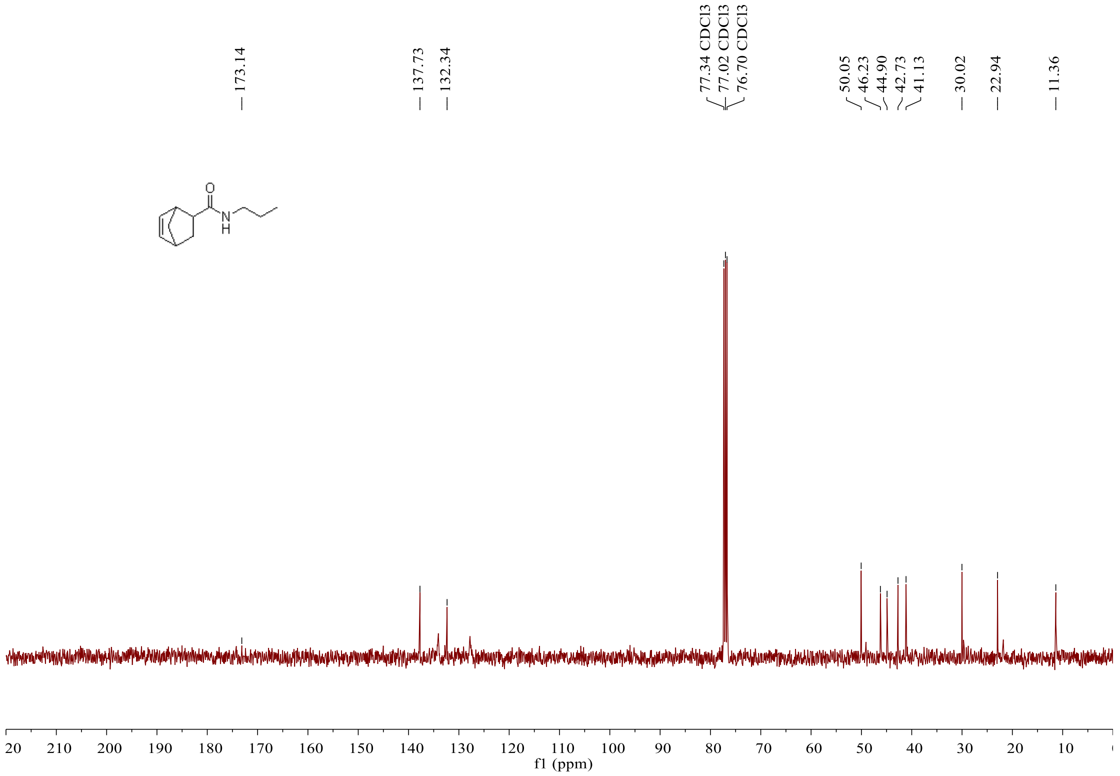

Supplementary Figure 150. ^{13}C NMR spectrum for compound **3au**

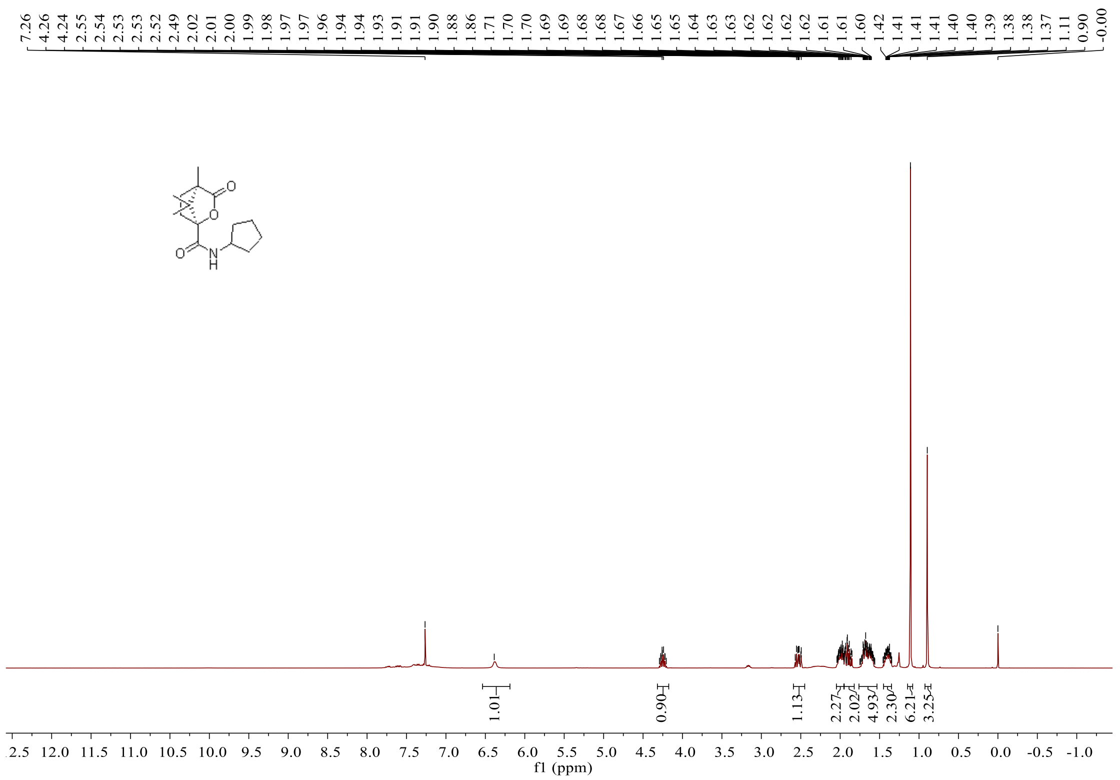

Supplementary Figure 151. ^{1}H NMR spectrum for compound **3av**

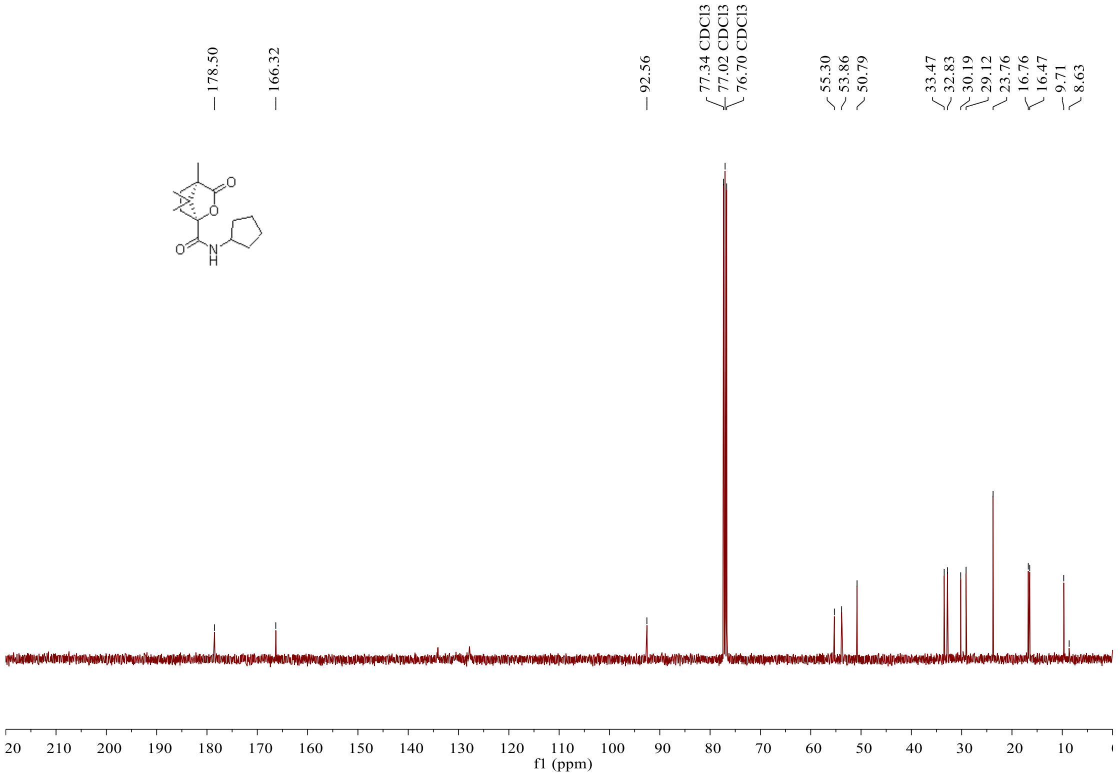

Supplementary Figure 152. ^{13}C NMR spectrum for compound **3av**

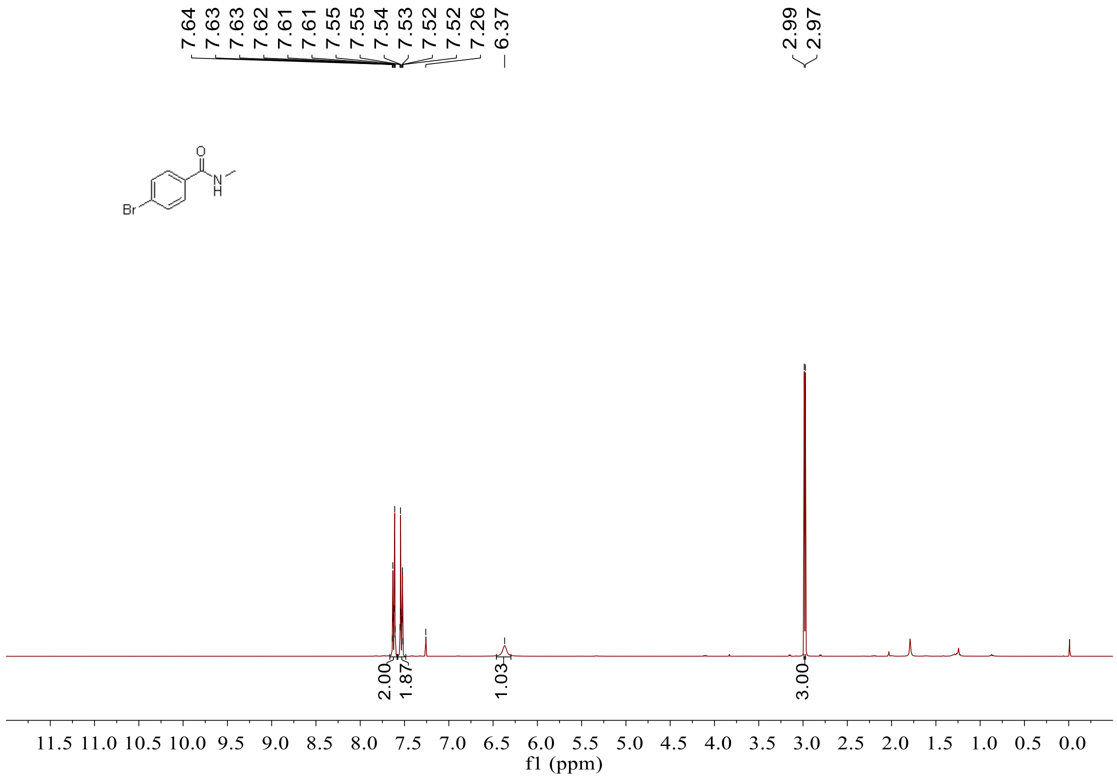

Supplementary Figure 153. ^{1}H NMR spectrum for compound **3aw**

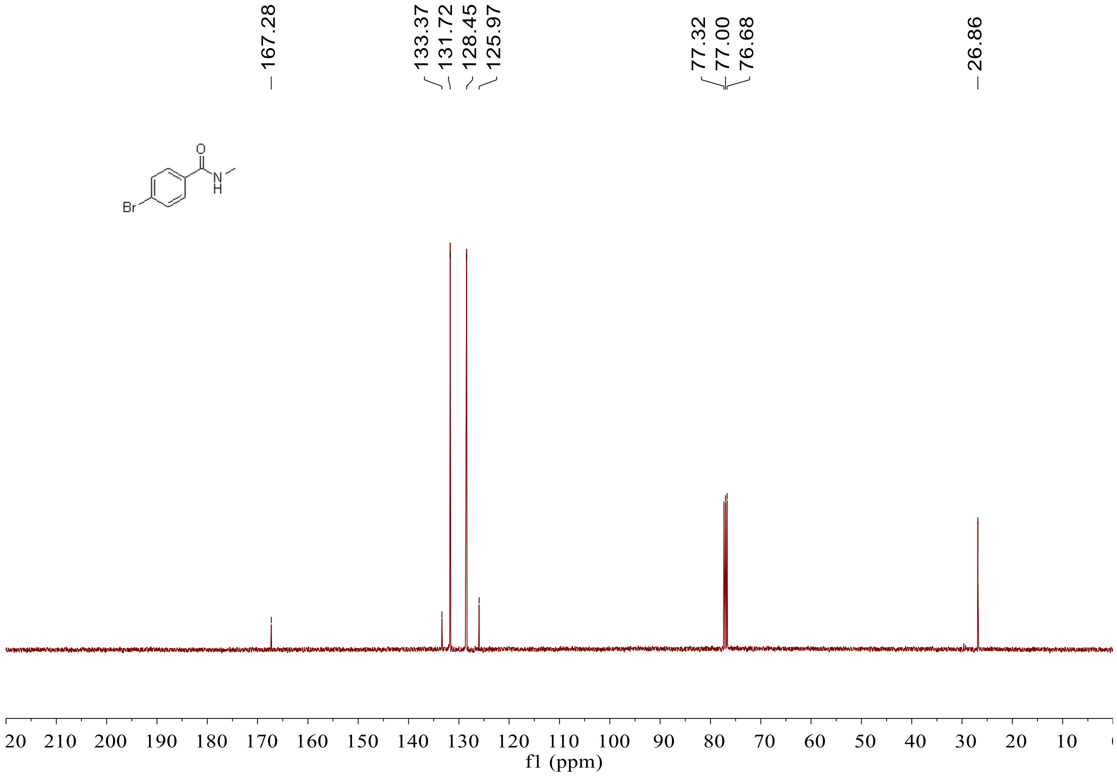

Supplementary Figure 154. ^{13}C NMR spectrum for compound **3aw**

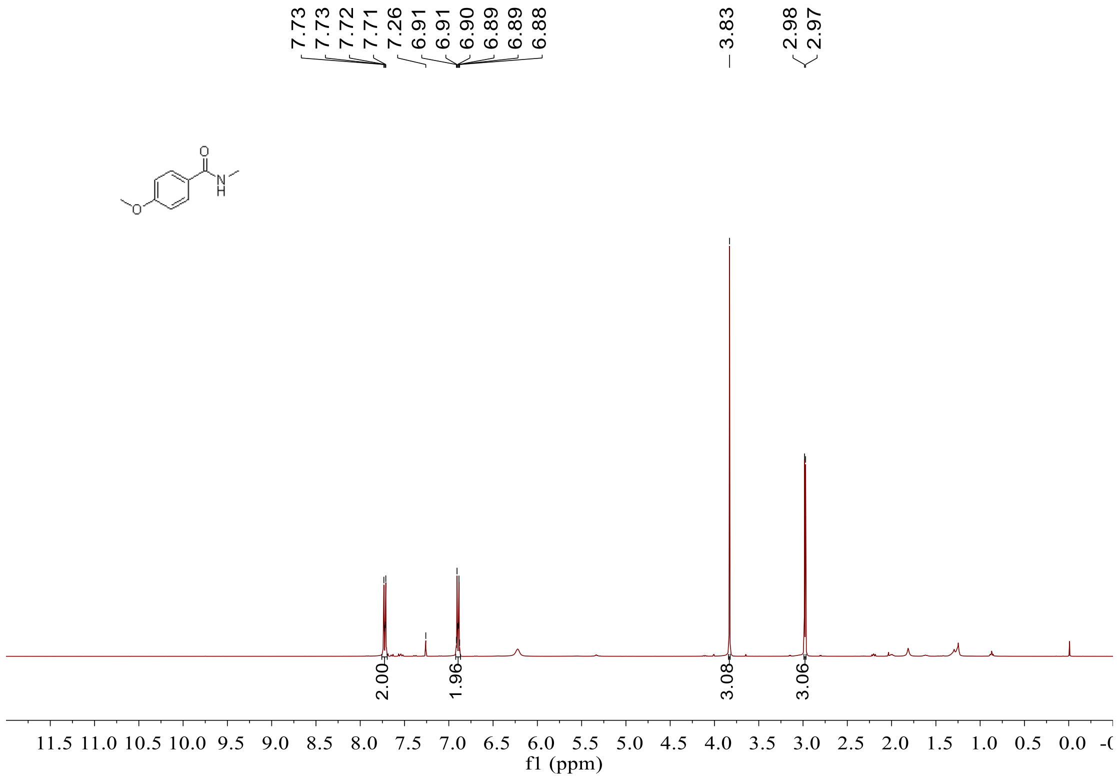

Supplementary Figure 155. ^{1}H NMR spectrum for compound **3ax**

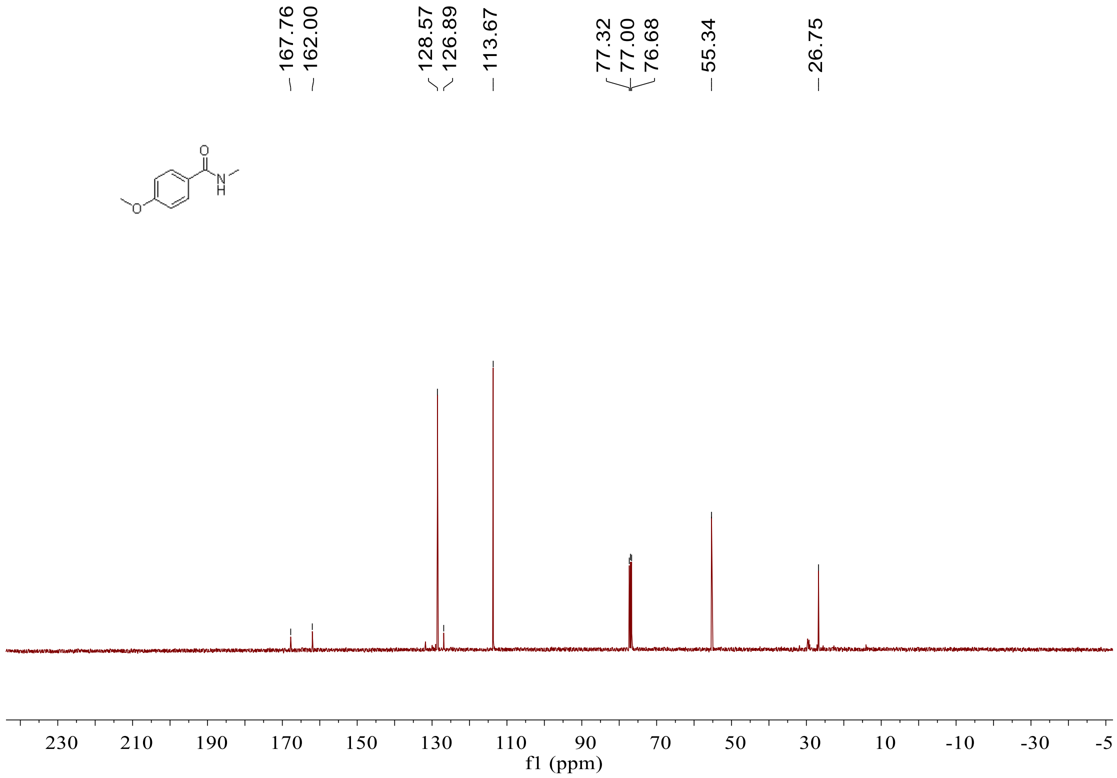

Supplementary Figure 156. ^{13}C NMR spectrum for compound **3ax**

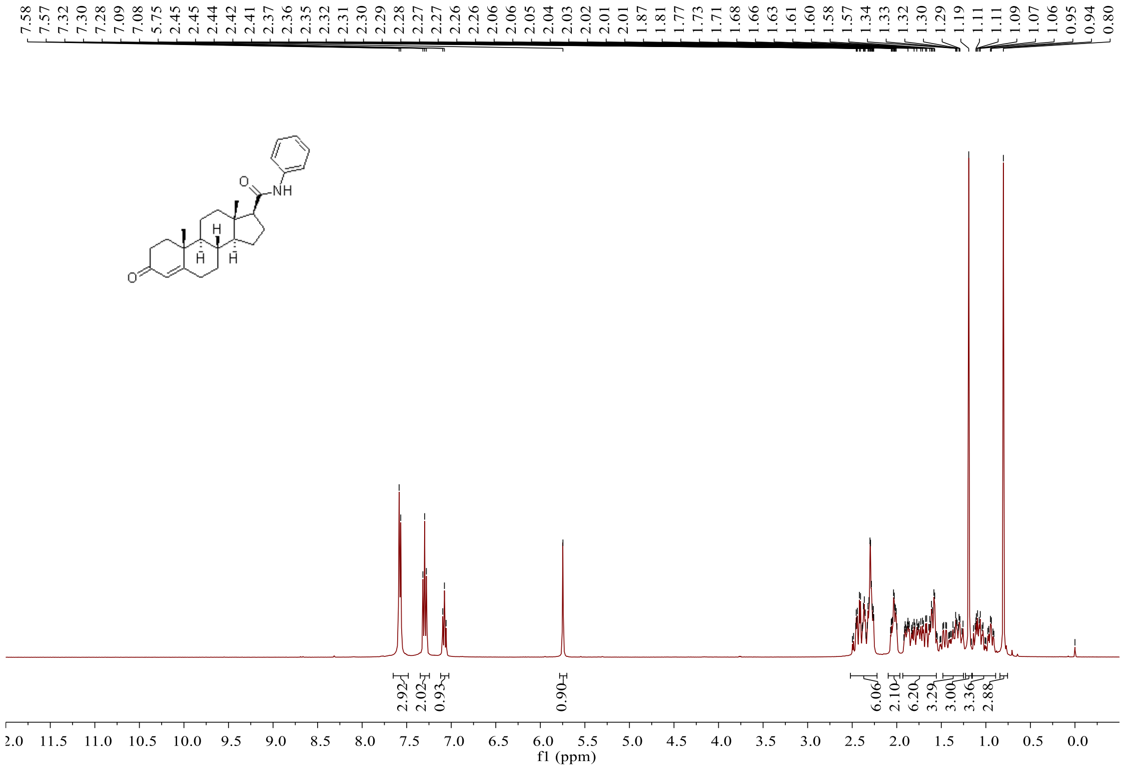

Supplementary Figure 157. ^{1}H NMR spectrum for compound **3ay**

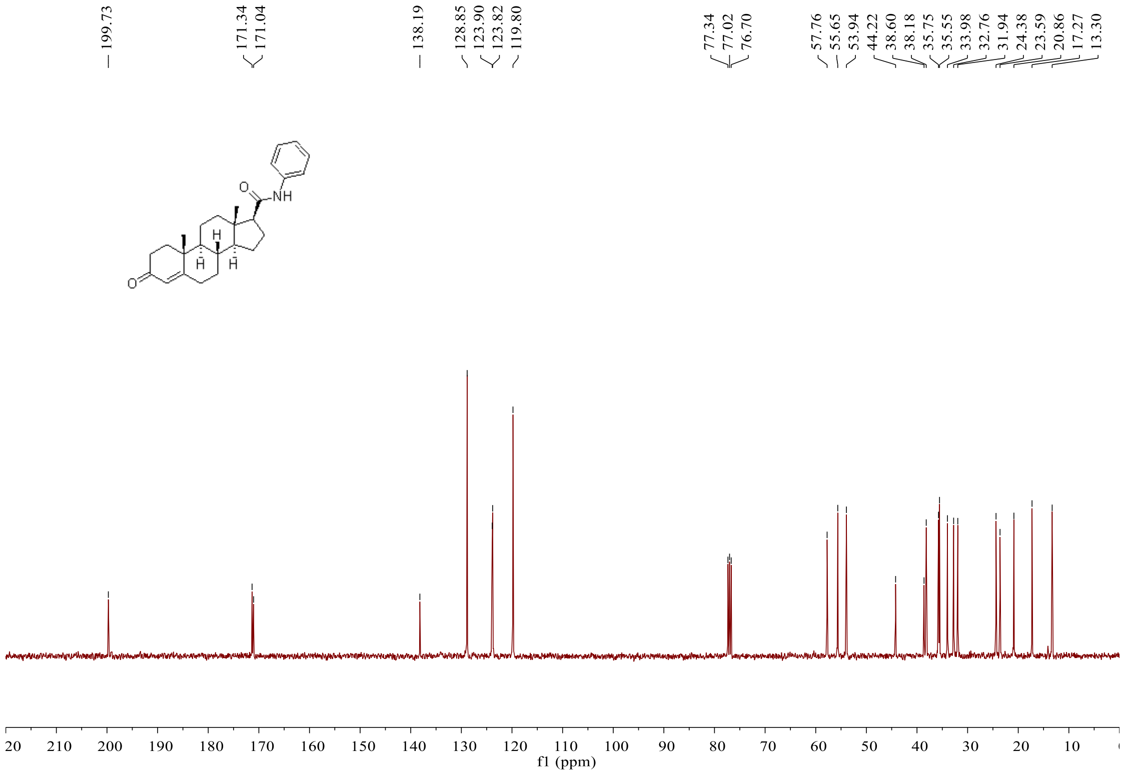

Supplementary Figure 158. ^{13}C NMR spectrum for compound **3ay**

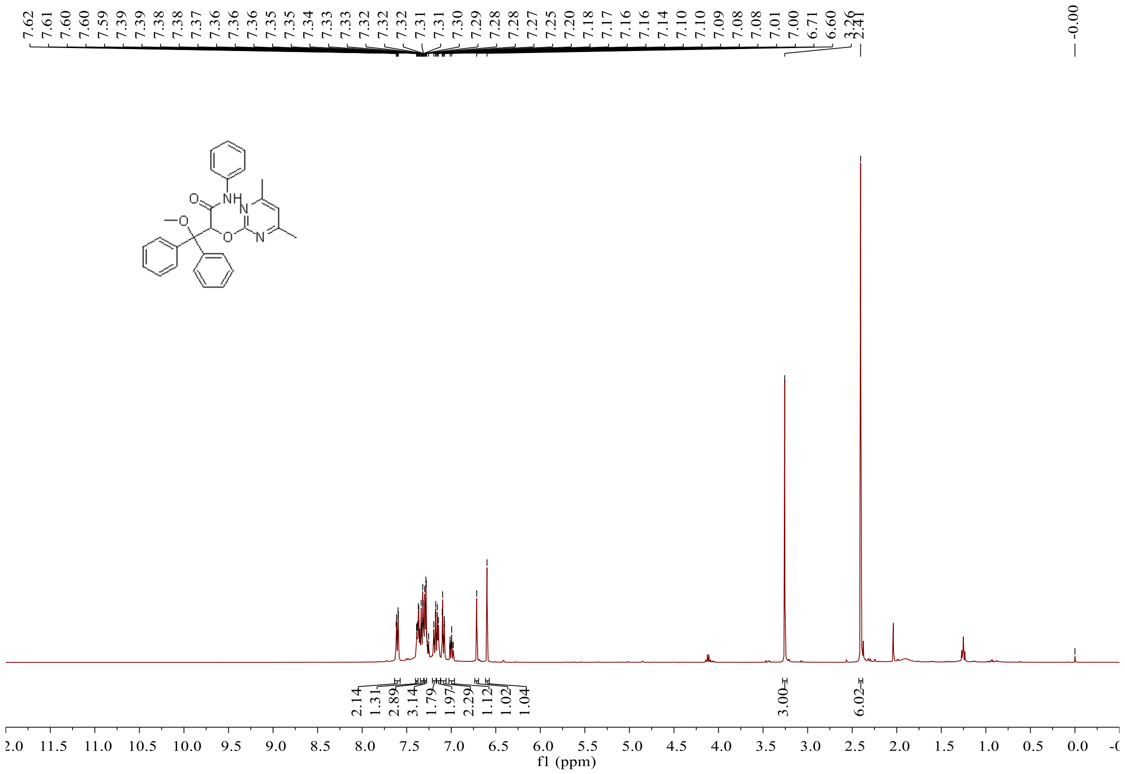

Supplementary Figure 159. ^{1}H NMR spectrum for compound **3az**

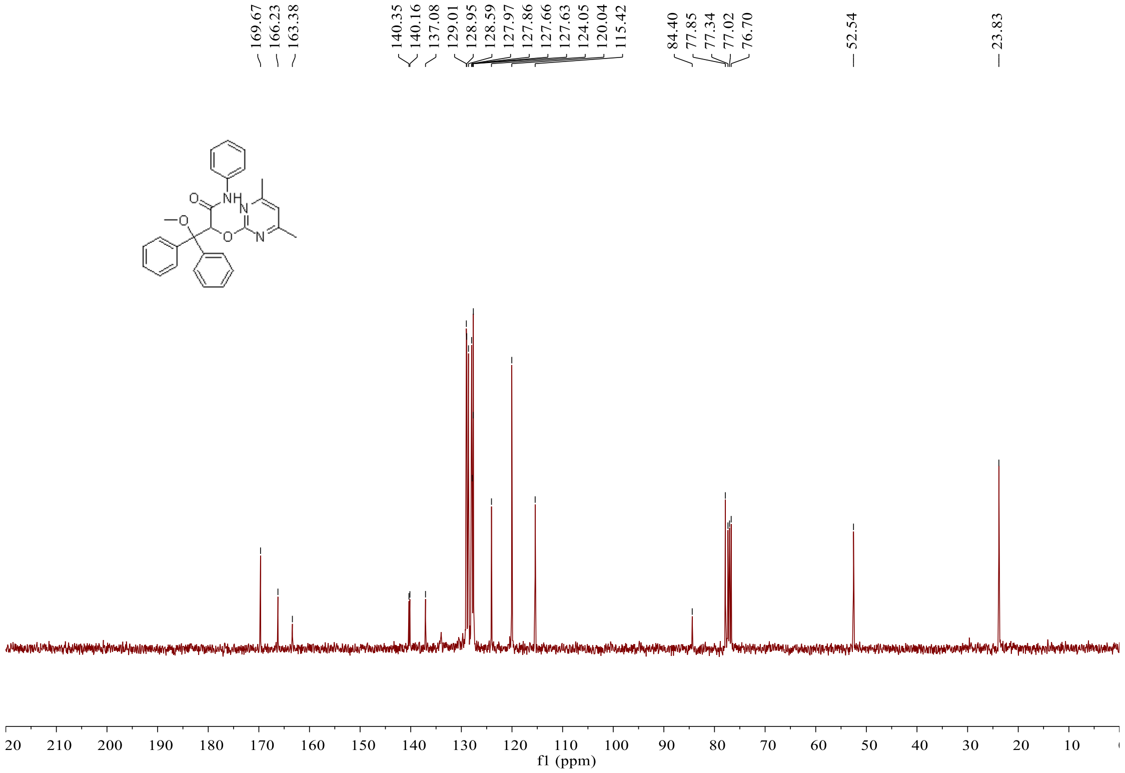

Supplementary Figure 160. ^{13}C NMR spectrum for compound **3az**

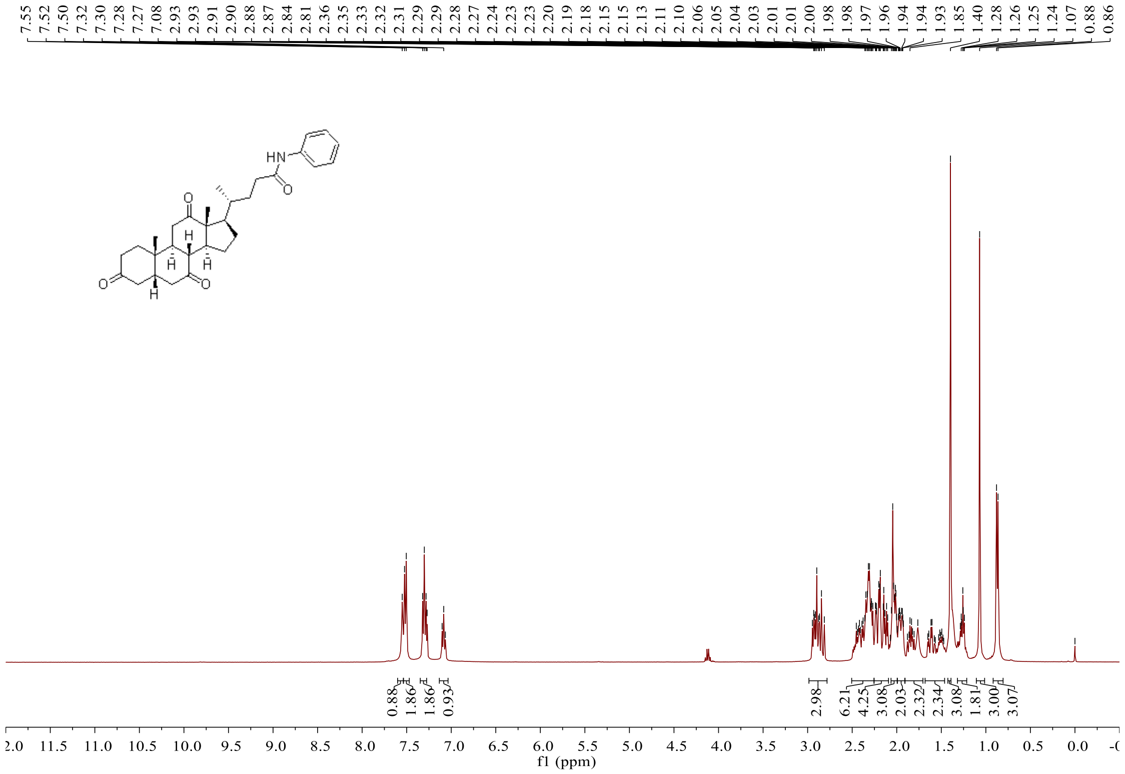

Supplementary Figure 161. ^{1}H NMR spectrum for compound **3ba**

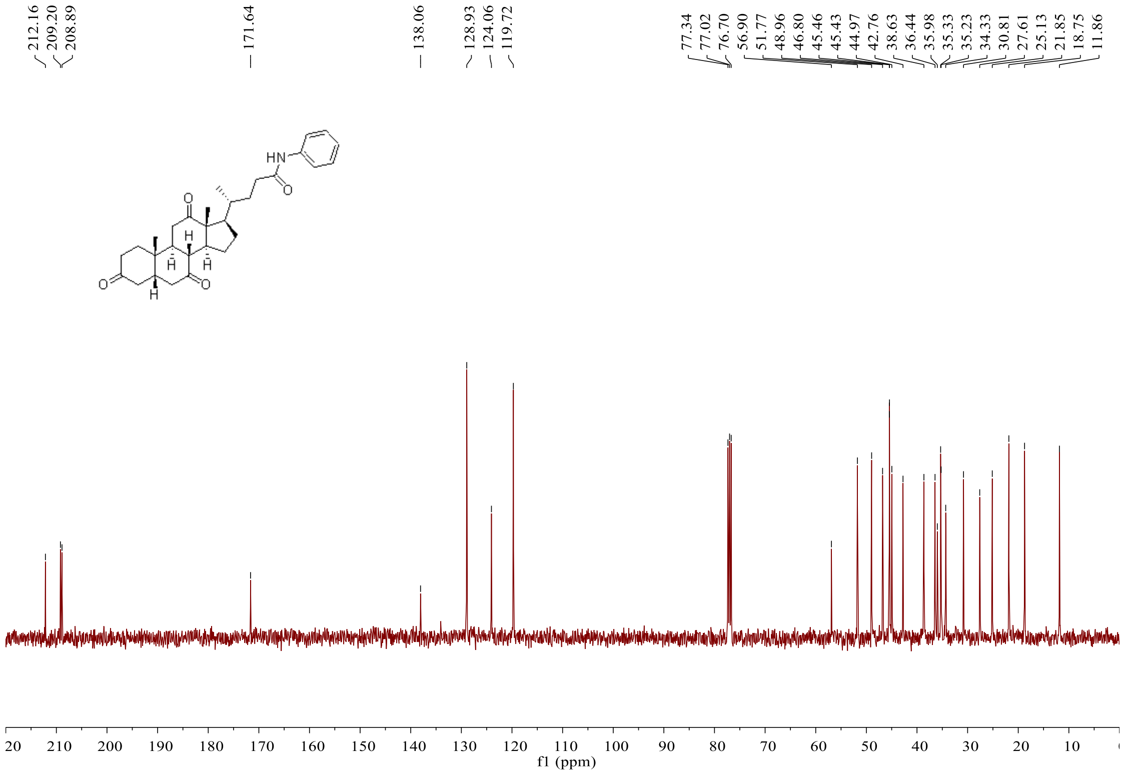

Supplementary Figure 162. ^{13}C NMR spectrum for compound **3ba**

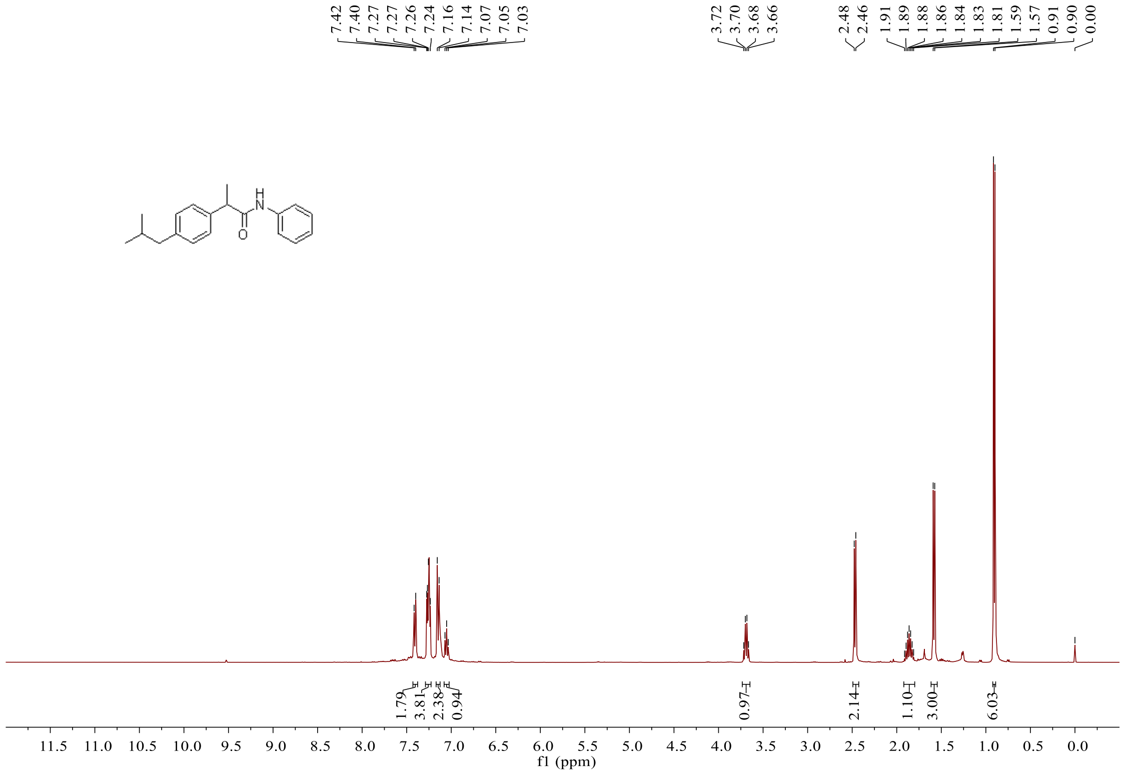

Supplementary Figure 163. ^{1}H NMR spectrum for compound **3bc**

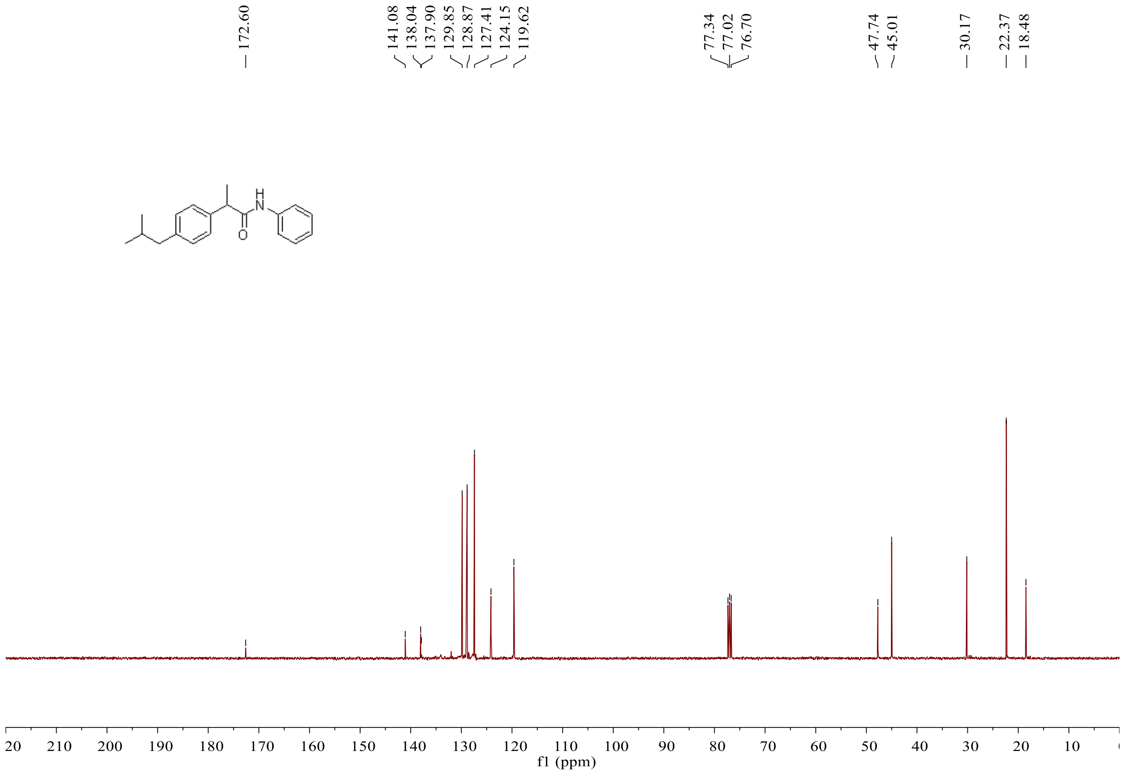

Supplementary Figure 164. ^{13}C NMR spectrum for compound **3bc**

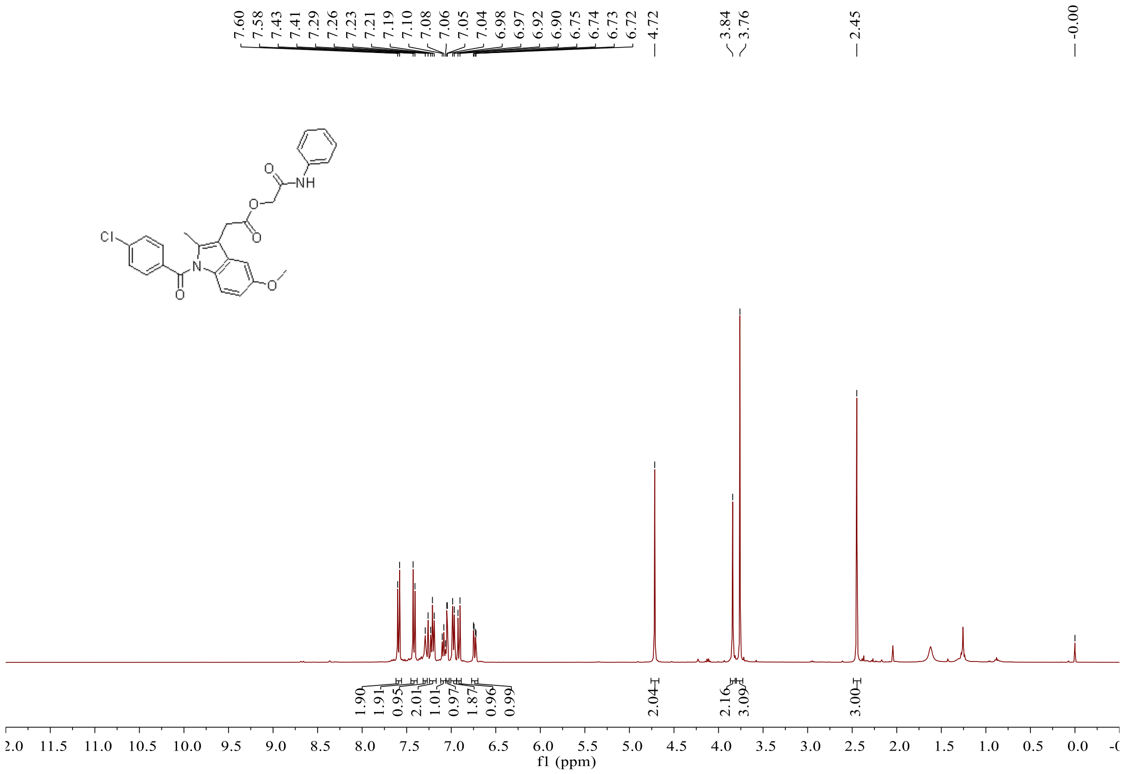

Supplementary Figure 165. ^{1}H NMR spectrum for compound **3bd**

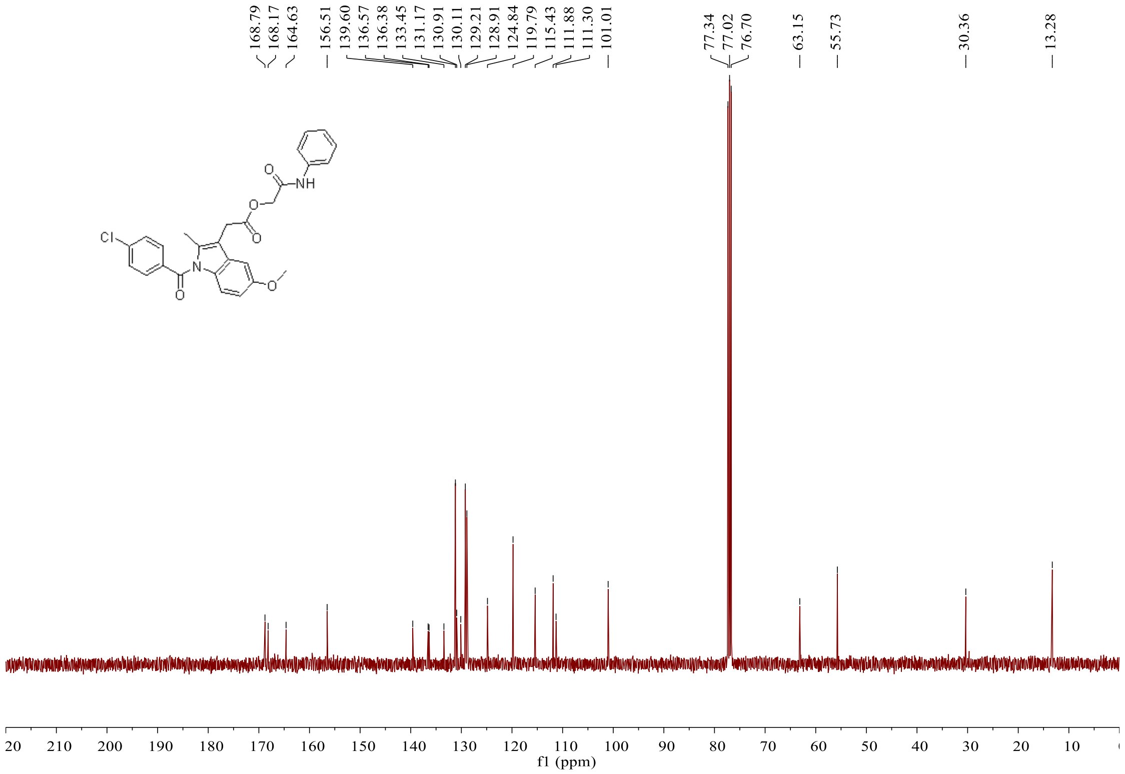

Supplementary Figure 166. ^{13}C NMR spectrum for compound **3bd**

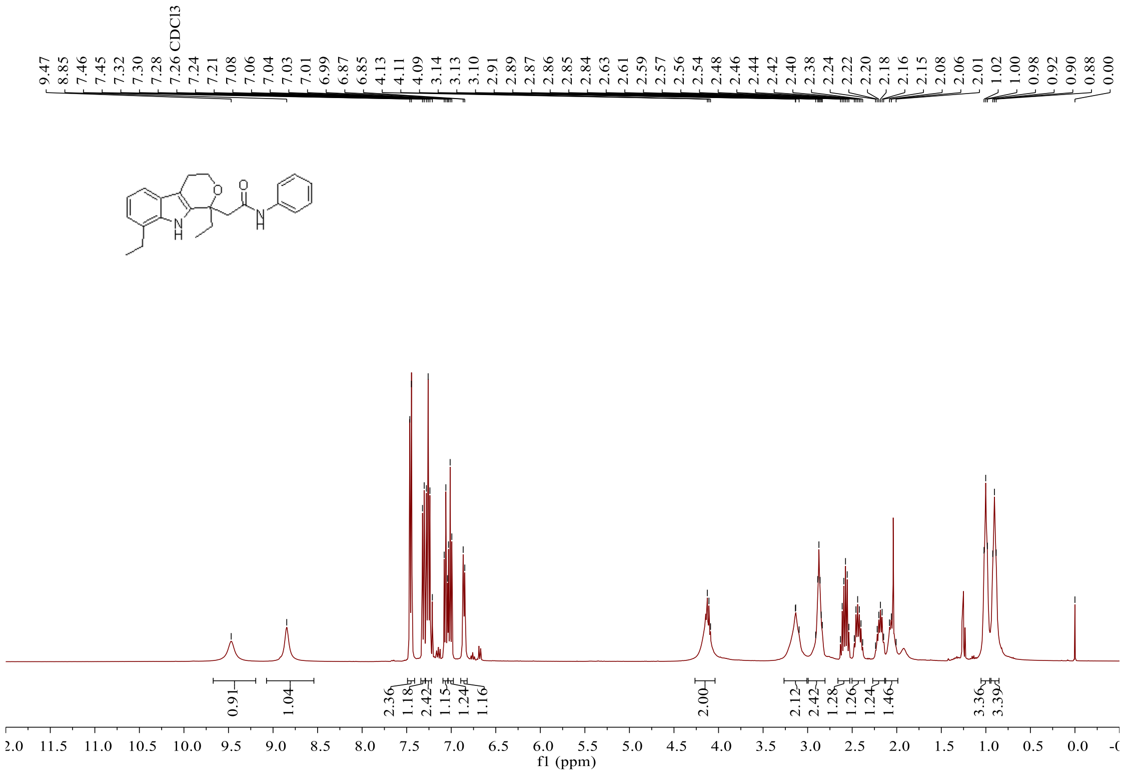

Supplementary Figure 167. ^{1}H NMR spectrum for compound **3be**

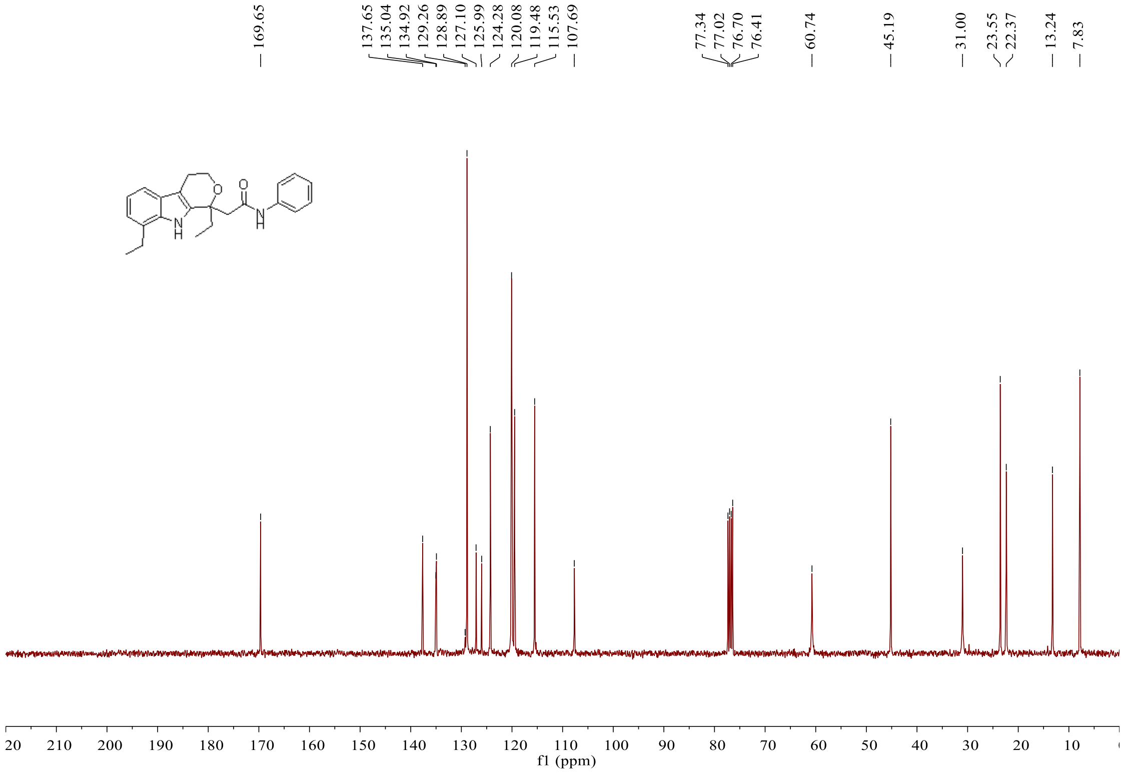

Supplementary Figure 168. ^{13}C NMR spectrum for compound **3be**

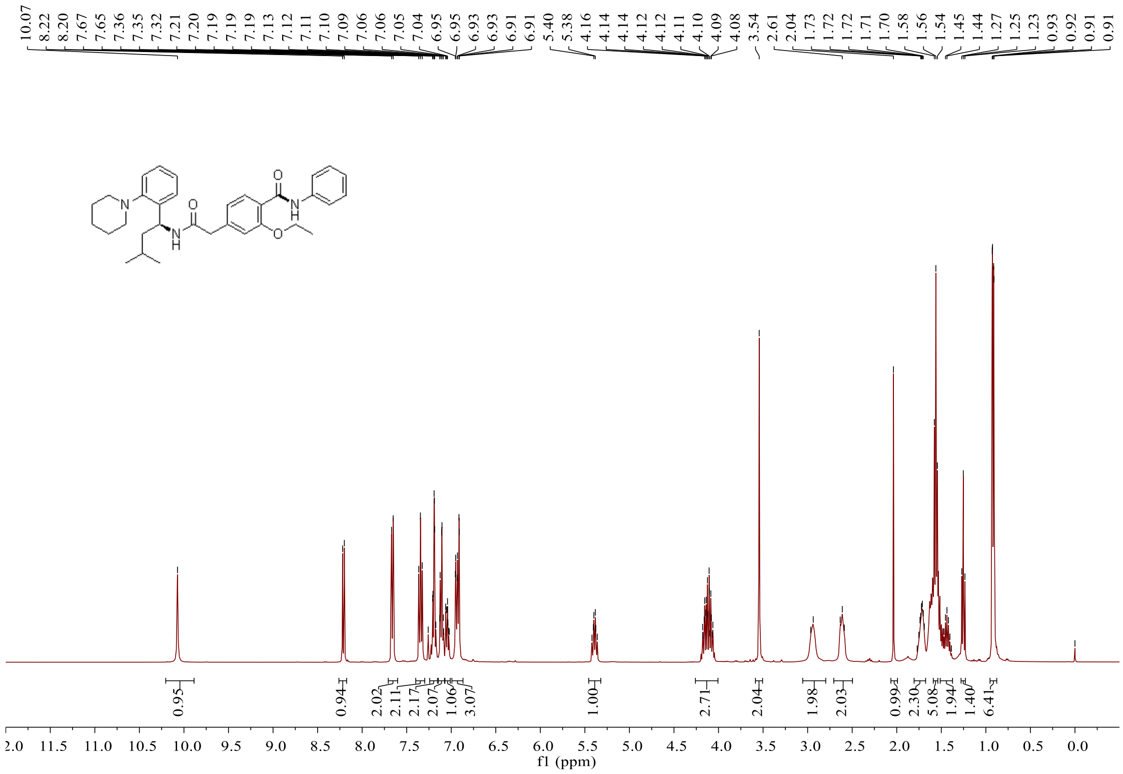

Supplementary Figure 169. ^{1}H NMR spectrum for compound **3bf**

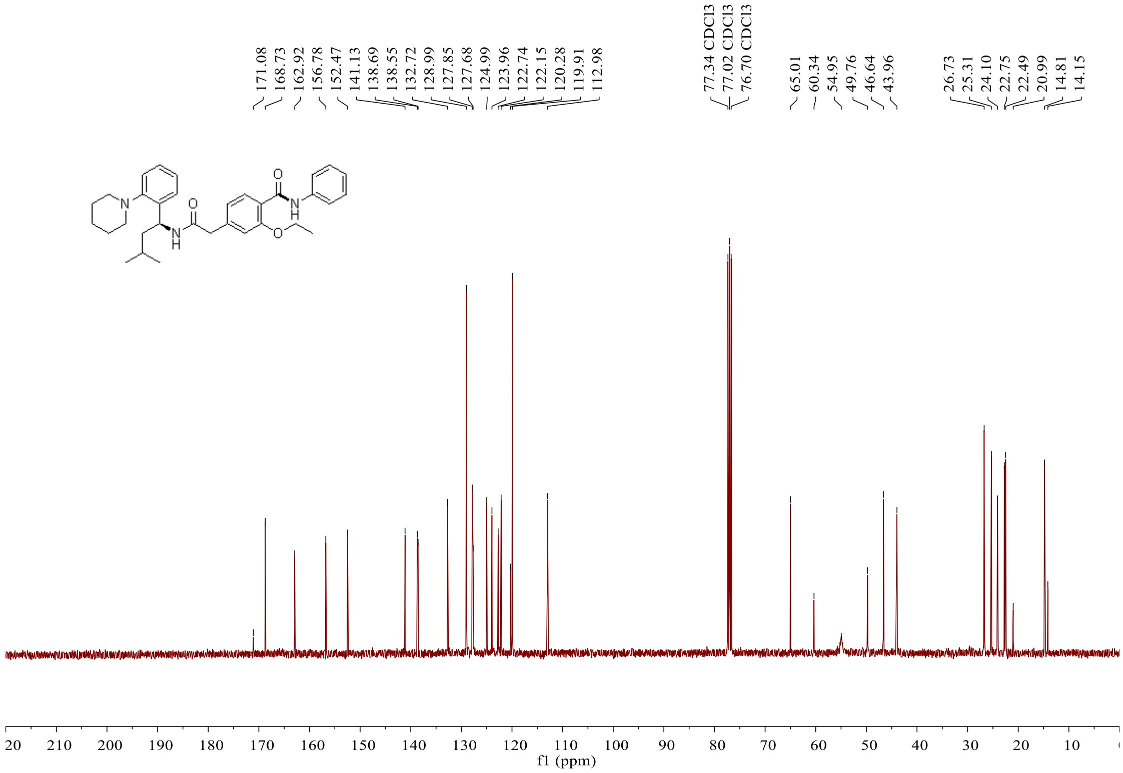

Supplementary Figure 170. ^{13}C NMR spectrum for compound **3bf**

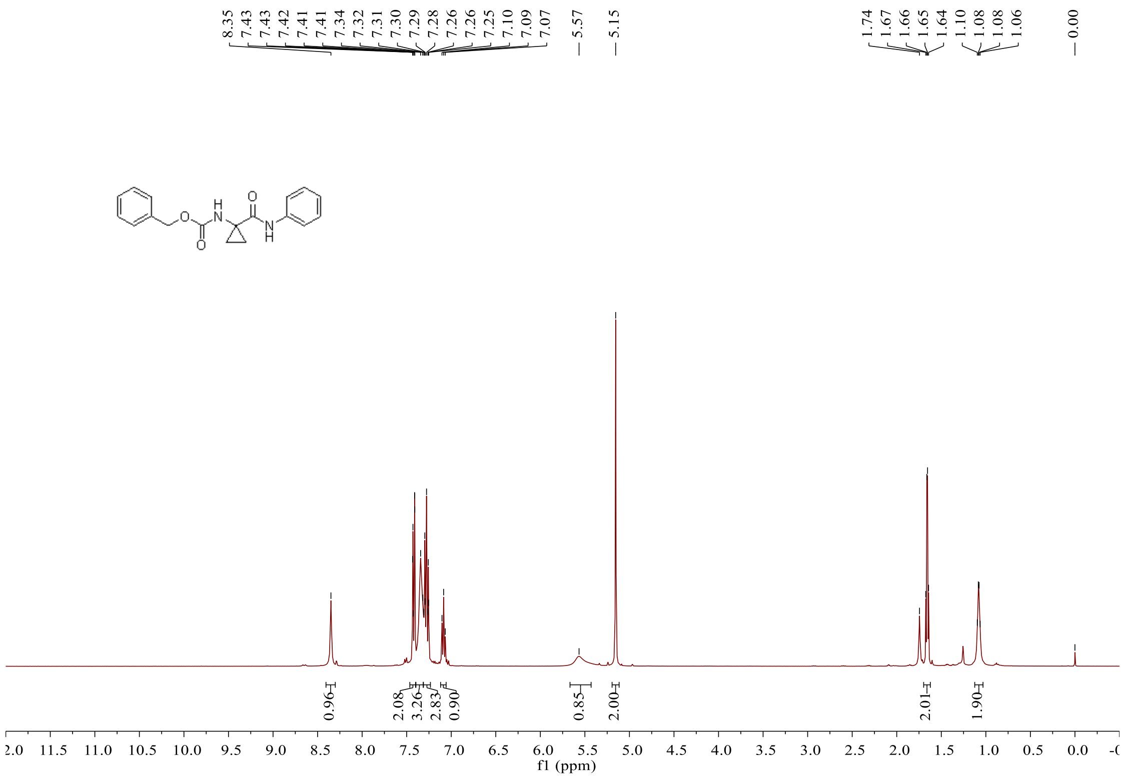

Supplementary Figure 171. ^{1}H NMR spectrum for compound **3bg**

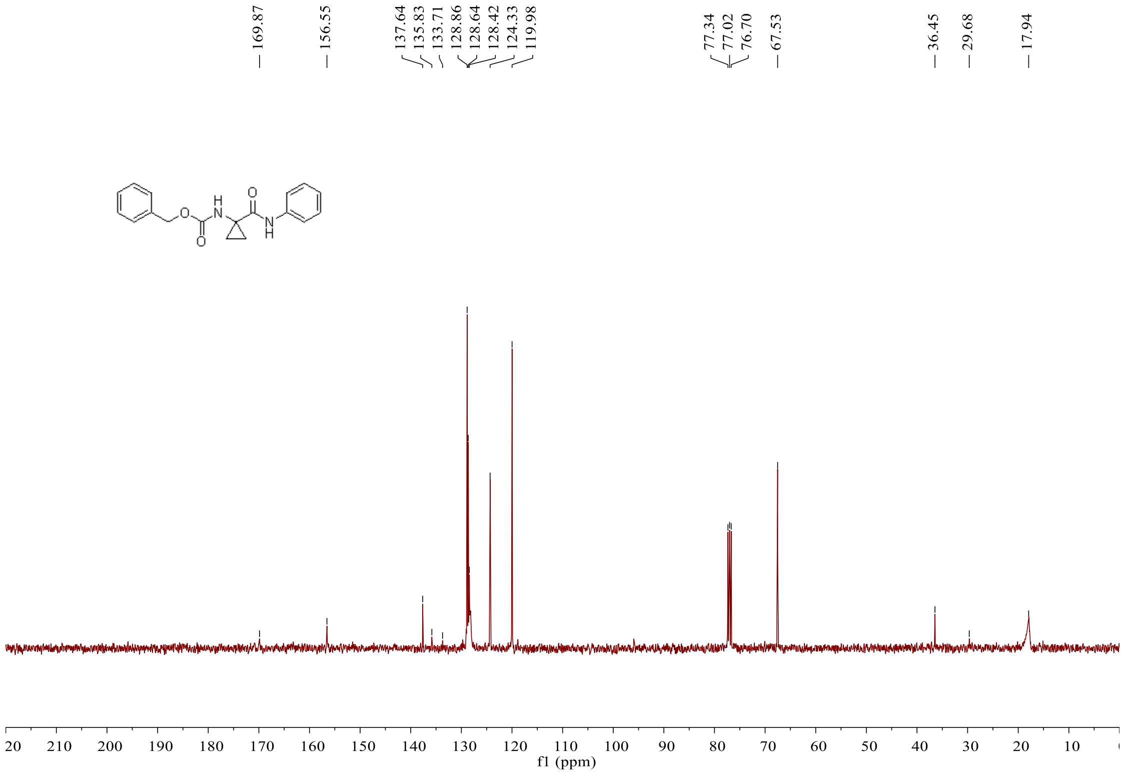

Supplementary Figure 172. ^{13}C NMR spectrum for compound **3bg**

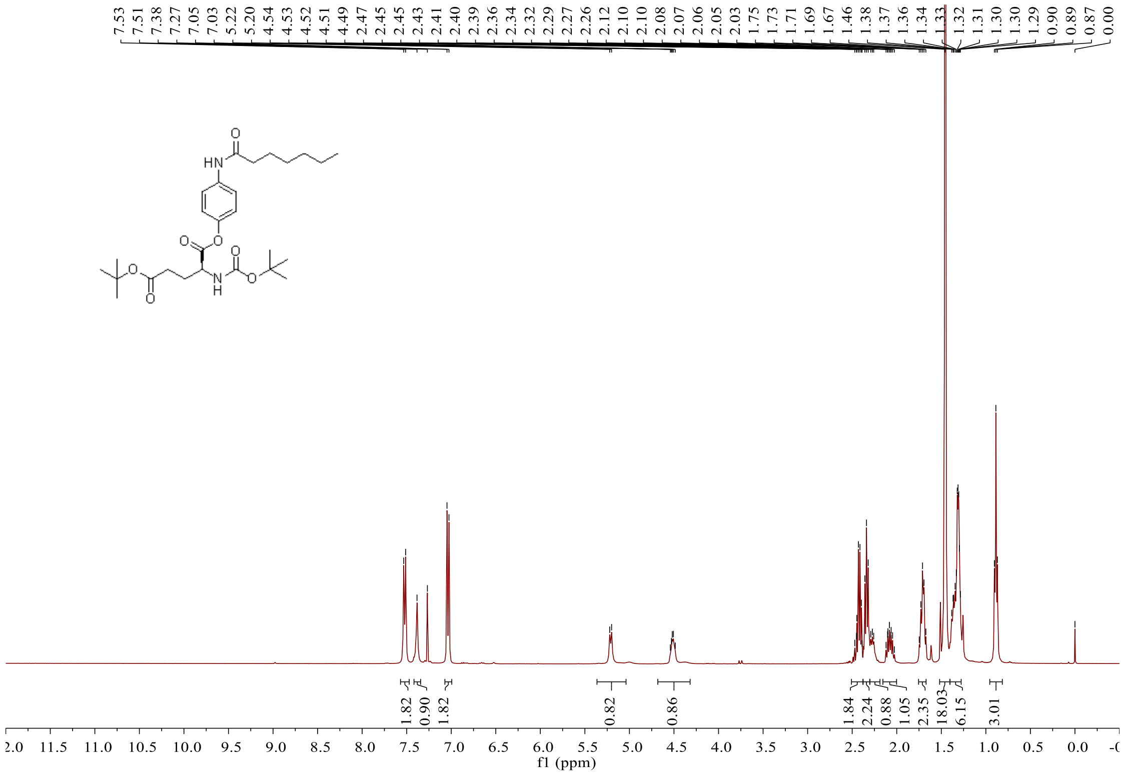

Supplementary Figure 173. ^{1}H NMR spectrum for compound **3bh**

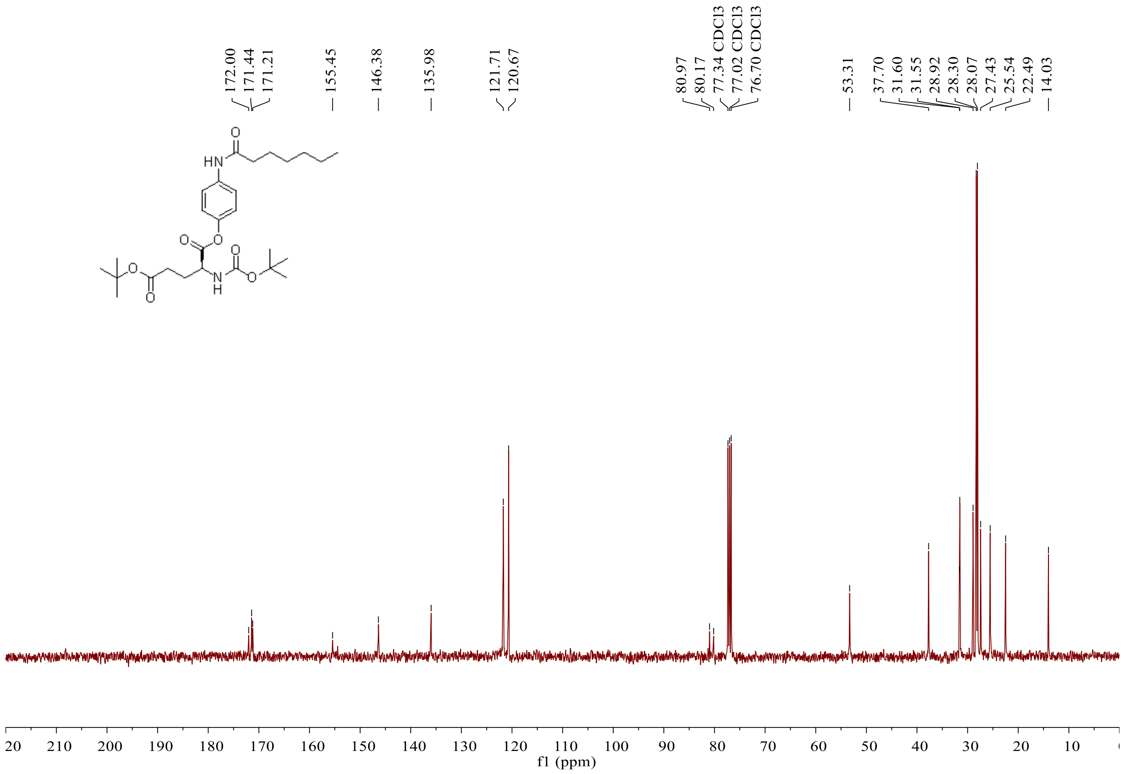

Supplementary Figure 174. ^{13}C NMR spectrum for compound **3bh**

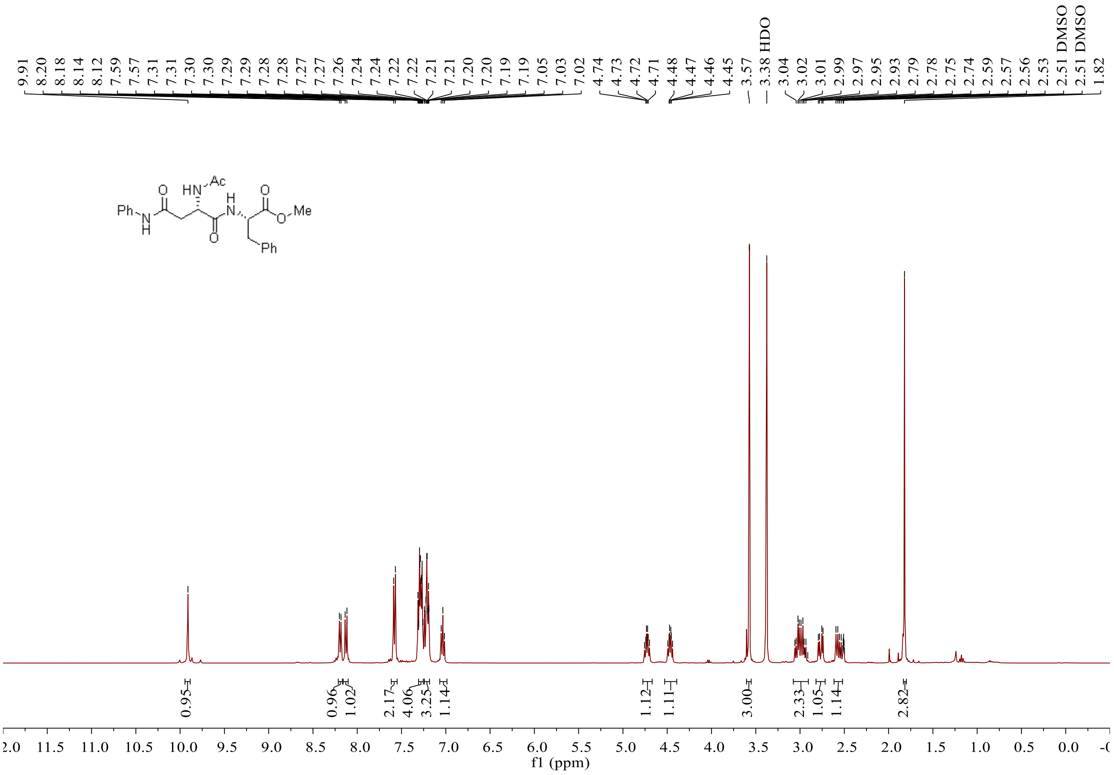

Supplementary Figure 175. ^{1}H NMR spectrum for compound **3bi**

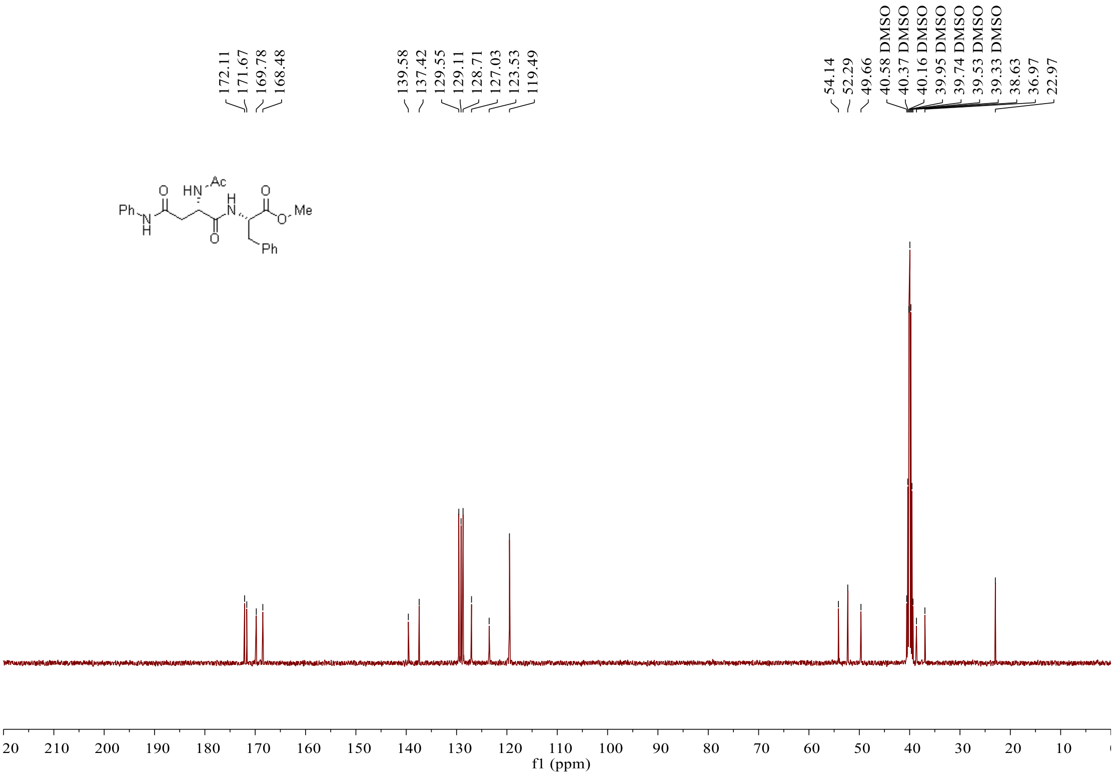

Supplementary Figure 176. ^{13}C NMR spectrum for compound **3bi**

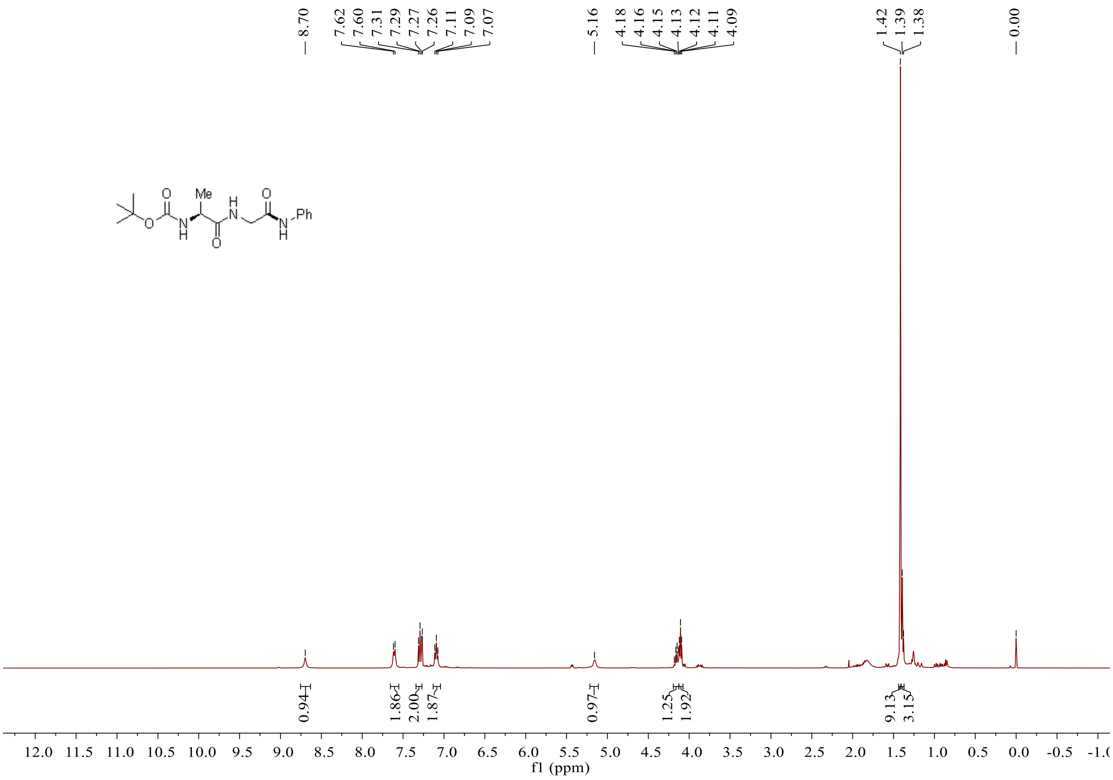

Supplementary Figure 177. ^{1}H NMR spectrum for compound **3bj**

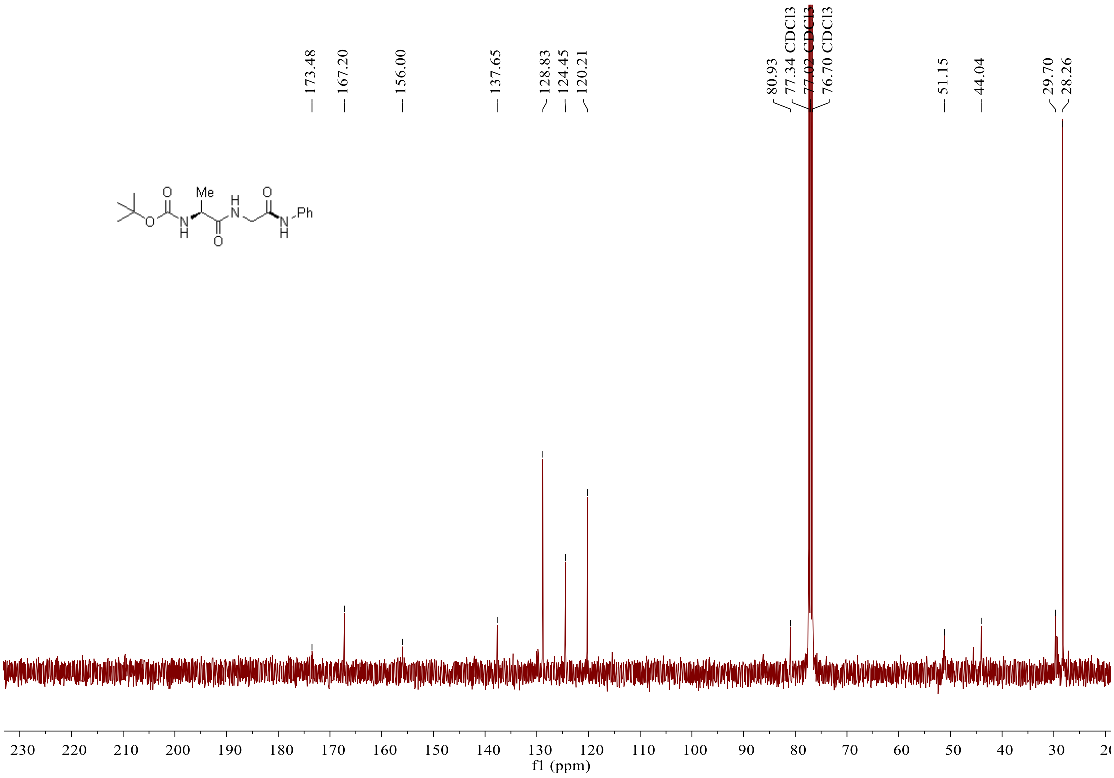

Supplementary Figure 178. ^{13}C NMR spectrum for compound **3bj**

7. HPLC spectra

Racemic form of 3cc

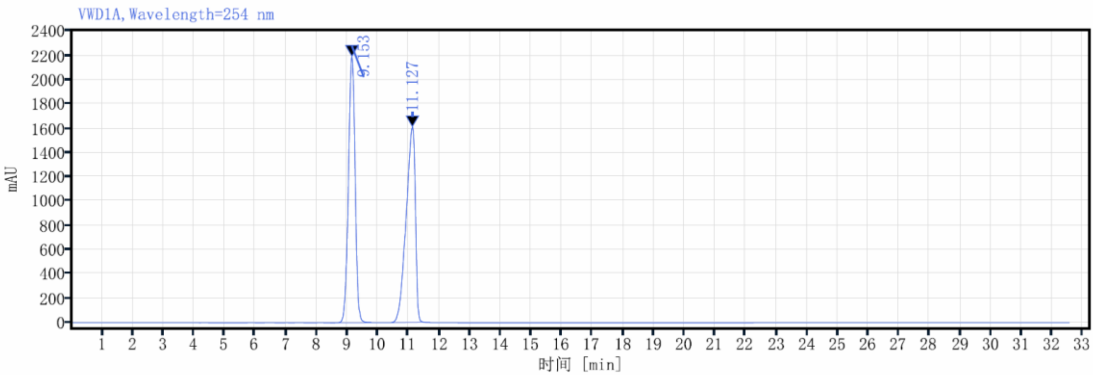

信号:	VWD1A, Wavelength=254 nm					
保留时间 [min]	类型	峰宽 [min]	峰面积	峰高	峰面积%	名称
9.153	BB	1.83	33322.63	2191.84	49.69	
11.127	BBA	1.15	33744.90	1607.68	50.31	
		总和	67067.53			

chiral HPLC analysis under the conditions as follows: Chiralcel IA column, ^{i}PrOH/^{n}hexane = 10/90, flow rate = 1.0 mL/min, λ = 254 nm.

Product of 3cc

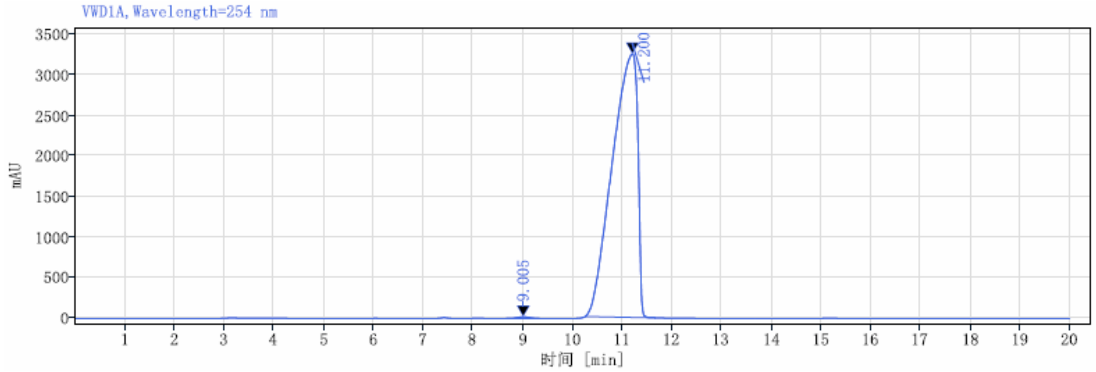

信号:	VWD1A, Wavelength=254 nm					
保留时间 [min]	类型	峰宽 [min]	峰面积	峰高	峰面积%	名称
9.005	VV	0.99	321.62	19.35	0.28	
11.200	MM m	1.40	115684.09	3252.01	99.72	
		总和	116005.71			

chiral HPLC analysis under the conditions as follows: Chiralcel IA column, ^{i}PrOH/^{n}hexane = 10/90, flow rate = 1.0 mL/min, λ = 254 nm.

Racemic form of 3dd

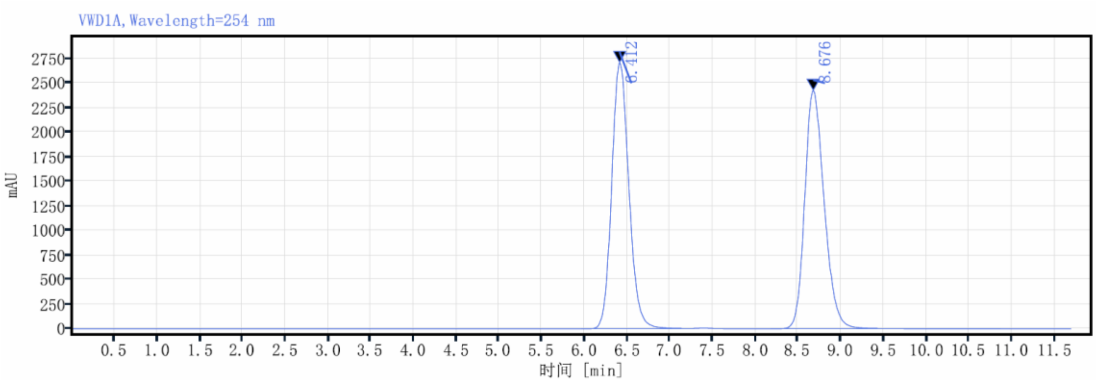

信号: VWD1A, Wavelength=254 nm

保留时间 [min]	类型	峰宽 [min]	峰面积	峰高	峰面积%	名称
6.412	BV	1.25	38034.63	2714.33	49.63	
8.676	VB	2.68	38599.96	2430.04	50.37	
		**总和**	**76634.58**			

chiral HPLC analysis under the conditions as follows: Chiralcel IA column, ^{i}PrOH/^{n}hexane = 10/90, flow rate = 1.0 mL/min, λ = 254 nm.

Product 3dd

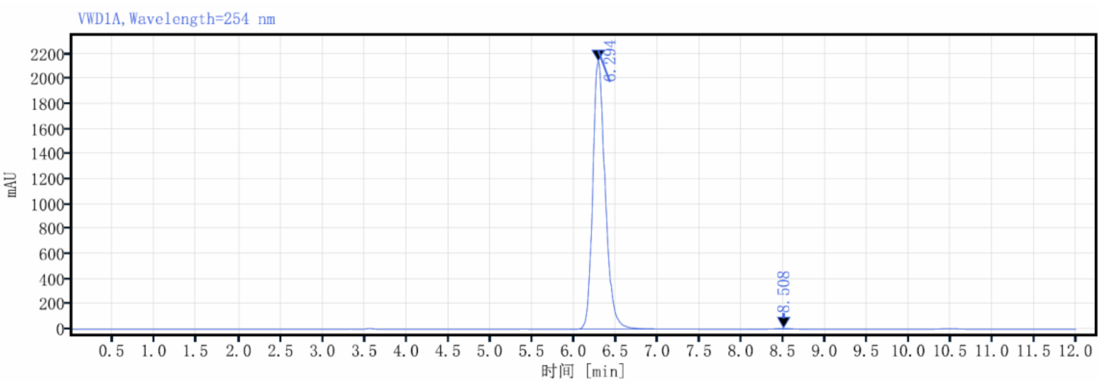

信号: VWD1A, Wavelength=254 nm

保留时间 [min]	类型	峰宽 [min]	峰面积	峰高	峰面积%	名称
6.294	BV	1.16	22333.75	2142.18	99.56	
8.508	BV	0.72	98.68	7.07	0.44	
		**总和**	**22432.43**			

chiral HPLC analysis under the conditions as follows: Chiralcel IA column, ^{i}PrOH/^{n}hexane = 10/90, flow rate = 1.0 mL/min, λ = 254 nm.

8. The crystallographic data

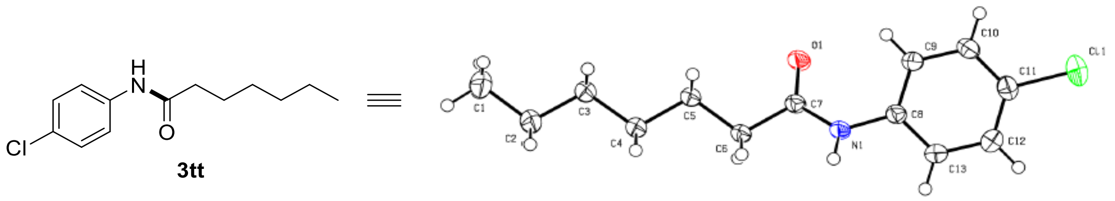

3tt

Identification code	CCDC 2055473
Empirical formula	C13 H18 Cl N O
Formula weight	239.73
Temperature	296.15
Wavelength	0.71073
Crystal system, space group	Triclinic, P -1
	a = 5.0161(5) Å alpha = 102.723(4)
Unit cell dimensions	b = 8.7042(10) Å beta = 93.318(3)
	c = 15.6268(15) Å gamma = 106.329(4)
Volume	633.43(12) Å ^{3}
Z, Calculated density	2, 1.257 g/cm^{3}
Absorpt coefficient	0.281 mm^{-1}
F(000)	256
Limiting indices	-7<=h<=7, -12<=k<=12, -20<=l<=22
Data / parameters	3800 / 146
Goodness-of-fit	1.039
Final R indices [I>2\s(I)]	R1 = 0.0443, wR2 = 0.1284
Cl1-C11 1.7357(12)	N1-H1 0.8600
O1-C7 1.2268(12)	N1-C7 1.3566(13)
N1-C8 1.4130(13)	C1-H1A 0.9600
C1-H1B 0.9600	C1-H1C 0.9600
C1-C2 1.5217(19)	C2-H2A 0.9700
C2-H2B 0.9700	C2-C3 1.5155(16)
C3-H3A 0.9700	C3-H3B 0.9700
C3-C4 1.5187(16)	C4-H4A 0.9700
C4-H4B 0.9700	C4-C5 1.5180(15)
C5-H5A 0.9700	C5-H5B 0.9700
C5-C6 1.5206(15)	C6-H6A 0.9700
C6-H6B 0.9700	C6-C7 1.5113(14)
C8-C9 1.3918(14)	C8-C13 1.3893(16)
C9-H9 0.9300	C9-C10 1.3852(16)
C10-H10 0.9300	C10-C11 1.3796(18)
C11-C12 1.3805(18)	C12-H12 0.9300
C12-C13 1.3857(17)	C13-H13 0.9300

Supplementary Reference

1. Nykaza T. V., Cooper J. C., Radosevich A. T. Anti-1,2,2,3,4,4-hexamethylphosphetane 1-oxide. Organic Synth **2019**, *96*, 418-435.
2. Nykaza T. V., Li G., Yang J., Luzung M. R., Radosevich A. T. P(III)/P(V) = O catalyzed cascade synthesis of N-functionalized azaheterocycles. Angew Chem Int Ed Engl **2020**, *59*, 4505-4510.
3. Nykaza T. V., Harrison T. S., Ghosh A., Putnik R. A., Radosevich A. T. A biphilic phosphetane catalyzes N-N bond-forming cadogan heterocyclization via P(III)/P(V) horizontal lineo redox cycling. J Am Chem Soc **2017**, *139*, 6839-6842.
4. Zhu, Y.-P., Sergeyev, S., Franck, P., Orru, R. V. A., Maes, B. U. W. Amine activation: synthesis of N-(Hetero)arylamides from isothioureas and carboxylic acids. Org. Lett. **2016**, *18*, 4602– 4605.
5. Wang W., Morohoshi T., Ikeda T., Chen L. Inhibition of Lux quorum-sensing system by synthetic N-acyl-L-homoserine lactone analogous. Acta Biochim Biophys Sin, **2008**, *40*, 1023-1028.
6. Ghosh S., et al. Direct amide bond formation from carboxylic acids and amines using activated alumina balls as a new, convenient, clean, reusable and low cost heterogeneous catalyst. Green Chem., **2012**, *14*, 3220–3229.
